# Optimal human papillomavirus vaccination strategies to prevent cervical cancer in low-income and middle-income countries in the context of limited resources: a mathematical modelling analysis

**DOI:** 10.1016/S1473-3099(20)30860-4

**Published:** 2021-11

**Authors:** Mélanie Drolet, Jean-François Laprise, Dave Martin, Mark Jit, Élodie Bénard, Guillaume Gingras, Marie-Claude Boily, Michel Alary, Iacopo Baussano, Raymond Hutubessy, Marc Brisson

**Affiliations:** a Centre de recherche du CHU de Québec, Université Laval, Quebec City, QC, Canada; b Département de médecine sociale et preventive, Université Laval, Quebec City, QC, Canada; c Centre for Mathematical Modelling of Infectious Disease, London School of Hygiene & Tropical Medicine, London, UK; d Modelling and Economics Unit, Public Health England, London, UK; e School of Public Health, University of Hong Kong, Hong Kong Special Administrative Region, China; f MRC Centre for Global Infectious Disease Analysis, Department of Infectious Disease Epidemiology, Imperial College London, London, UK; g Institut national de santé publique du Québec, Québec City, QC, Canada; h International Agency for Research on Cancer, Lyon, France; i Department of Immunization, Vaccines and Biologicals, World Health Organization, Geneva, Switzerland

## Abstract

**Background:**

Introduction of human papillomavirus (HPV) vaccination has been slow in low-income and middle-income countries (LMICs) because of resource constraints and worldwide shortage of vaccine supplies. To help inform WHO recommendations, we modelled various HPV vaccination strategies to examine the optimal use of limited vaccine supplies and best allocation of scarce resources in LMICs in the context of the WHO global call to eliminate cervical cancer as a public health problem.

**Methods:**

In this mathematical modelling analysis, we developed HPV-ADVISE LMIC, a transmission-dynamic model of HPV infection and diseases calibrated to four LMICs: India, Vietnam, Uganda, and Nigeria. For different vaccination strategies that encompassed use of a nine-valent vaccine (or a two-valent or four-valent vaccine assuming high cross-protection), we estimated three outcomes: reduction in the age-standardised rate of cervical cancer, number of doses needed to prevent one case of cervical cancer (NNV; as a measure of efficiency), and the incremental cost-effectiveness ratio (ICER; in 2017 international $ per disability-adjusted life-year [DALY] averted). We examined different vaccination strategies by varying the ages of routine HPV vaccination and number of age cohorts vaccinated, the population targeted, and the number of doses used. In our base case, we assumed 100% lifetime protection against HPV-16, HPV-18, HPV-31, HPV-33, HPV-45, HPV-52, and HPV-58; vaccination coverage of 80%; and a time horizon of 100 years. For the cost-effectiveness analysis, we used a 3% discount rate. Elimination of cervical cancer was defined as an age-standardised incidence of less than four cases per 100 000 woman-years.

**Findings:**

We predicted that HPV vaccination could lead to cervical cancer elimination in Vietnam, India, and Nigeria, but not in Uganda. Compared with no vaccination, strategies that involved vaccinating girls aged 9–14 years with two doses were predicted to be the most efficient and cost-effective in all four LMICs. NNV ranged from 78 to 381 and ICER ranged from $28 per DALY averted to $1406 per DALY averted depending on the country. The most efficient and cost-effective strategies were routine vaccination of girls aged 14 years, with or without a later switch to routine vaccination of girls aged 9 years, and routine vaccination of girls aged 9 years with a 5-year extended interval between doses and a catch-up programme at age 14 years. Vaccinating boys (aged 9–14 years) or women aged 18 years or older resulted in substantially higher NNVs and ICERs.

**Interpretation:**

We identified two strategies that could maximise efforts to prevent cervical cancer in LMICs given constraints on vaccine supplies and costs and that would allow a maximum of LMICs to introduce HPV vaccination.

**Funding:**

World Health Organization, Canadian Institute of Health Research, Fonds de recherche du Québec–Santé, Compute Canada, PATH, and The Bill & Melinda Gates Foundation.

**Translations:**

For the French and Spanish translations of the abstract see Supplementary Materials section.

## Introduction

Globally, in 2020, an estimated 604 000 new cases of cervical cancer and 342 000 cervical cancer-related deaths occurred.^1^ Over 80% of cases of cervical cancer occur in low-income and middle-income countries (LMICs).^1^ These inequalities in the burden of cervical cancer are set to increase, with 88% of high-income countries (HICs) having introduced HPV vaccination in women and girls as of the end of 2019 compared with less than 40% of LMICs.^1^, ^2^, ^3^ Furthermore, as of the end of 2019, 44% of high-income countries also vaccinate boys, compared with only 5% of LMICs.^3^ The reasons for the lower uptake of HPV vaccination in LMICs, which have been exacerbated by the COVID-19 pandemic, include financial and human resource constraints,^4^ paucity of evidence on its population-level effectiveness and cost-effectiveness in LMICs,^5^ and, importantly, worldwide shortage of HPV vaccine supply.^6^

**Research in context**

**Evidence before this study**

Approximately 500 000 new cases of cervical cancer are diagnosed in low-income and middle-income countries (LMICs) each year. Human papillomavirus (HPV) vaccines are highly effective: the Papillomavirus Rapid Interface for Modelling and Economics (PRIME) model predicted that HPV vaccination of girls aged 12 years with two doses of the vaccine was cost-effective in 156 (87%) of 179 countries. However, less than 40% of LMICs have introduced HPV vaccination programmes. Key barriers to introduction include financial and human resource constraints, and, since 2019, a worldwide shortage of HPV vaccine supply that might last until 2024. These barriers might have been intensified by the COVID-19 pandemic. In parallel, the WHO director-general has issued a global call to eliminate cervical cancer as a public health problem, which will result in sustained efforts to achieve high vaccination coverage across LMICs.

**Added value of this study**

In this modelling analysis we identified two novel HPV vaccination strategies that should minimise the number of doses needed to prevent one case of cervical cancer and the cost per DALY averted: two-dose routine vaccination of girls aged 14 years with or without a later switch to routine vaccination of girls aged 9 years, and routine vaccination of girls aged 9 years with an extended interval of 5 years between doses and a catch-up programme for girls aged 14 years. These strategies would maximise prevention of cervical cancer with the fewest doses in the short term and at the lowest cost, which would allow a maximum of LMICs to introduce HPV vaccination and could reduce the effect of HPV vaccine supply shortage on efforts to eliminate cervical cancer.

**Implication of all the available evidence**

Our modelling results have directly informed WHO Strategic Advisory Group of Experts on Immunization's recommendation in October, 2019, to continue vaccination with a two-dose schedule; temporarily postpone vaccination of multiple-age cohorts, older age groups (≥15 years), and gender-neutral vaccination; and consider implementing strategies such as those identified in our study.

Because of the substantial burden and inequalities in the distribution of cervical cancer across the world, the WHO Director-General Tedros Adhanom Ghebreyesus made a global call for action towards the elimination of cervical cancer as a public health problem.^7^ This initiative would entail efforts to achieve high coverage of routine vaccination in girls, but could also include efforts to accelerate elimination by vaccinating multiple age cohorts of women, introducing gender-neutral vaccination (ie, vaccination of both boys and girls), and increasing uptake of cervical cancer screening. In the context of the call for elimination of cervical cancer and limited resources, HPV vaccination policy decisions in LMICs will probably require trade-offs between two potentially conflicting perspectives: maximising population-level impact (eg, to reach elimination of cervical cancer) versus optimising vaccination efficiency and return on investment (eg, to minimise the number of doses needed to prevent one case of cancer and to minimise the cost-effectiveness ratio).

The main HPV vaccination policy questions being examined in LMICs by their health authorities and by WHO are as follows: what are the ages and the number of age cohorts that should be vaccinated? Should only girls or girls and boys be vaccinated? And how many and when should doses be given per vaccinee?^6^ To date, results from modelling studies have predicted that routine and multiple-age cohort HPV vaccination of girls is likely to be highly cost-effective in most LMICs.^8^, ^9^ However, most models for LMICs have not included realistic country-specific representation of sexual behaviour and natural history of HPV infection and cervical cancer and have not been designed to examine other, more complex policy questions (eg, adding vaccination of boys and different strategies among girls aged 9–14 years). Moreover, to our knowledge, no comprehensive modelling analysis has investigated the optimal HPV vaccination strategies in terms of efficiency and return on investment for any part of the world while simultaneously comparing different ages at vaccination, multiple age-cohort vaccination strategies, girls-only versus gender-neutral vaccination, and the interval between doses.

In this Article, we present the modelling results that were presented to the WHO Strategic Advisory Group of Experts on Immunization (SAGE) in October, 2019, to help inform their global HPV vaccination policy recommendations.^10^ The objective of the modelling study was to identify optimal HPV vaccination strategies in the context of elimination of cervical cancer, vaccine shortage, and resource constraints. We used a transmission-dynamic mathematical model to estimate the population-level effect, efficiency, and cost-effectiveness of 27 HPV vaccination strategies varying the age of routine vaccination (9 years or 14 years), number of age cohorts vaccinated, the population targeted (girls only or girls and boys), and the interval between doses.

## Methods

### Study design and countries

In this mathematical modelling analysis, we modelled the impact of HPV vaccination in four countries: India, Vietnam, Uganda, and Nigeria. We selected these countries because of differences between their populations in sexual behaviour, HPV prevalence, and cervical cancer burden. For example, according to Demographic Health Survey data, the mean number of lifetime sexual partners for men was 1·4 in Vietnam, 1·9 in India, 4·1 in Nigeria, and 6·6 in Uganda; the estimated HPV prevalence in a 2010 meta-analaysis^11^ was less than 10% in south (India) and southeast (Vietnam) Asia compared with more than 20% in sub-Saharan Africa (Nigeria and Uganda); and the age-standardised incidence of cervical cancer in 2020 was estimated to be less than 20 cases per 100 000 woman-years in Vietnam and India compared with more than 30 per 100 000 woman-years in Nigeria and Uganda.^12^ Furthermore, sufficient data were available to calibrate and validate the models to these countries (data sources are in appendix 3 [p 8] and online).

### Vaccination scenarios

For the four countries, we first compared seven HPV vaccination scenarios with two doses, starting with routine vaccination of girls aged 9 years. We then added the vaccination of multiple-aged cohorts of girls and women aged 9–14 years, 9–18 years, or 9–25 years within the first year of the programme. Finally, we added the vaccination of boys aged 9–14 years to the previous multiple-aged cohort vaccination scenarios of girls and women (appendix 4 p 2). Because of the shortage of HPV vaccines^4^, ^13^ and the WHO recommendation to vaccinate girls aged 9–14 years,^14^ we then examined seven strategies that could potentially optimise two-dose vaccination of girls aged 9–14 years (appendix 4 [pp 2, 25–26]). These strategies involved increasing the interval between doses (from the currently recommended 6-month interval to 5 years), delaying the age of routine vaccination to 14 years (with or without a subsequent switch to routine vaccination at age 9 years), implementing catch-up campaigns for the first 5 years of the programme, and various other combinations (appendix 4 p 2). In sensitivity analysis, we examined the same multiple-aged cohort vaccination scenarios (9–14 years, 9–18, years, 9–25 years) for girls only or girls and boys, but giving only one dose to girls and boys aged 9–14 years. These vaccination strategies were developed in collaboration with the WHO HPV Working Group to ensure they were relevant for SAGE recommendations. In our base case, we assumed 80% vaccination coverage.

### Model structure

To examine HPV vaccination policy questions in LMIC settings we developed HPV-ADVISE LMIC, an individual-based, transmission-dynamic model of multiple types of HPV infection and disease (appendix 3 pp 1–5). HPV-ADVISE LMIC has the same underlying structure for all LMICs and the same basic model structure as previous versions of HPV-ADVISE for HICs,^15^, ^16^ with modifications to capture differences in sexual behaviour between LMICs and HICs and to examine elimination of cervical cancer. The main difference between HPV-ADVISE LMIC and HPV-ADVISE for HICs is that we included female sex workers and their clients in HPV-ADVISE LMIC because the proportion of men who declare they have paid for sex is higher in some LMICs than in HICs,^17^ male clients of female sex workers have been shown to be bridge populations for sexually transmitted infections,^18^, ^19^ and female sex workers, as a core group for HPV transmission, might have a role in whether elimination of cervical cancer can be achieved.^20^

In HPV-ADVISE LMIC, individuals are attributed one of four mutually exclusive levels of sexual activity: level 0, including women and men who are married and remain married to a single partner throughout their life; level 1, including women who get married and then divorce or whose partner has a concurrent partner during their marriage (excluding female sex workers) and men who get married and then divorce or have a concurrent partnership during their marriage (excluding female sex workers); level 2, including women and men who never get married; and level 3, including female sex workers and men who pay for sex. Notably, men who pay for sex can also have concurrent long-term partnerships with level 0–1 women. These definitions of levels of sexual activity were chosen because they are good markers of sexual behaviour and risk of HPV infection in these countries, and valid data sources are available (appendix 3 p 21). The model includes stable (long-term) and casual partnerships. Partnership formation and dissolution are based on age-specific, gender-specific, and sexual activity-specific rates of partner acquisition and separation and mixing patterns. HPV transmission depends on sexual behaviour, per sex act probability of transmission, and natural history of infection (determined by the duration of infectiousness and natural immunity). 18 HPV types, including all types in the nine-valent vaccine, were modelled individually and independently (assuming no synergy or competition). Each HPV type has its own natural history parameters in terms of transmission, persistence, clearance, and disease progression to cervical cancer. After clearance, individuals might develop type-specific natural immunity. The natural history of cervical cancer is represented by the following type-specific health states: susceptible to HPV infection; infected; immune; cervical intraepithelial neoplasia of grade 1 (CIN1), grade 2 (CIN2), and grade 3 (CIN3); and three stages of cervical cancer.

### Model parameters

We parametrised and calibrated the model to each country separately. We estimated the parameter values determining sexual behaviour, HPV transmission, and the natural history of HPV infection and cervical cancer by calibrating the model to country-specific data (appendix 3 pp 6–51). For each country, we identified 50 parameter sets that simultaneously fit highly stratified country-specific sexual behaviour and HPV epidemiological data (appendix 4 pp 27–31), taken from published articles, specific studies, and international population-based datasets (appendix 3 p 8). These 50 parameter sets represent uncertainty in model parameters and variability in sexual behaviour and HPV epidemiology within a country. Additionally, we validated the model to verify whether the model fit data that were not used during model calibration (appendix 3 pp 52–56).

We obtained age-specific and country-specific mortality rates from the WHO Global Health Observatory data repository^21^ and country-specific screening parameters from the Institut Català d'Oncologia Information Centre on HPV and Cancer.^22^, ^23^, ^24^, ^25^ Notably, most women (aged ≥18 years) from India, Vietnam, Uganda, and Nigeria have never been screened for cervical cancer (91–100% of women).^22^, ^23^, ^24^, ^25^

We assumed that the HPV vaccines were prophylactic and do not affect the natural history of HPV infection or disease among individuals infected by a vaccine type before vaccination. In the model, vaccine efficacy is specific to each of the 18 HPV types modelled. In our base case, we assumed that two doses of HPV vaccine provide 100% efficacy against infection with HPV-16, HPV-18, HPV-31, HPV-33, HPV-45, HPV-52, and HPV-58 (representing either a nine-valent vaccine or a two-valent or four-valent vaccine with very high cross-protection) and that duration of protection is lifelong. In sensitivity analyses, we investigated one-dose vaccination strategies, in which we assumed that one dose of vaccine could provide an efficacy against infection of 95% or 85% with a duration of protection of 30 or 20 years. We also varied vaccine efficacy against cross-protective types for the two-valent and four-valent vaccines (high^26^ or none).

We used two different vaccine prices per dose: $4·60 per dose (based on Gavi, the Vaccine Alliance prices) and $7·50 per dose (1·5 × Gavi price) in 2017 international $ (INT$). The vaccine delivery and operational costs were country specific and based on estimates from the Papillomavirus Rapid Interface for Modelling and Economics (PRIME) model.^8^ Treatment costs for cervical cancer were based on HPV pilot studies and from analyses using WHO-CHOICE methodologies.^27^, ^28^ Costs were inflated to 2017 INT$ (INT$1 would buy in the cited country a similar amount of goods and services US$1 would buy in the USA;^29^ appendix 4 p 4).

### Outcomes

We had three main outcomes (appendix 4 p 24). First, to examine the potential for elimination of cervical cancer, we used the absolute reduction of the age-standardised incidence of cervical cancer over time as our main population-level impact outcome. We used age-standardised incidence below four cases per 100 000 woman-years as the threshold for elimination of cervical cancer because this is the working definition used by WHO.^30^ Our secondary population-level impact outcomes were the relative reduction in age-standardised incidence of cervical cancer and prevalence of HPV-16 and HPV-18 (appendix 4 pp 6–7). Second, to examine the optimal use of limited vaccine supplies, we used the number of doses needed to prevent one case of cervical cancer (NNV) as our efficiency outcome. Finally, to examine optimal return on investment we used an incremental cost-effectiveness analysis. Our main cost-effectiveness outcome was the incremental cost-effectiveness ratio (ICER) in costs per disability-adjusted life-year (DALY) averted.

### Analysis

For age-standardisation of the incidence of cervical cancer, we used the age structure of the 2015 world female population aged 0–99 years.^31^ We calculated the effectiveness of vaccination strategies as the pre-vaccination incidence minus the incidence at equilibrium divided by the pre-vaccination incidence. We estimated the NNV by dividing the total number of doses given in a population over time by the number of cases of cervical cancer prevented over the same period. We calculated incremental NNVs as the additional number of doses needed to prevent an additional case of cervical cancer. For the first step in our cost-effectiveness analysis, we incrementally compared different strategies varying the number of cohorts, age, and population targeted (ie, we gradually increased the number of individuals vaccinated by either vaccinating additional cohorts of girls or by adding the vaccination of boys; appendix 4 p 2). For the second step, to identify optimal vaccination strategies of girls aged 9–14 years (appendix 4 pp 2, 25–26), we compared all strategies with no vaccination. We calculated ICERs as the additional cost per additional DALY saved. For reference, we used two country-specific cost-effectiveness thresholds, represented by the countries' gross domestic product (GDP) per capita and 0·5 × GDP per capita (India: $7056 and $3528; Vietnam: $6776 and $3388; Uganda: $1864 and $932; and Nigeria: $5861 and $2930).^32^ We did the economic analysis from the health-care payer perspective, including all direct medical costs. We discounted costs and outcomes at 3% per year following WHO recommendation.

We modelled a 100-year time-horizon to capture both short-term and long-term benefits of HPV vaccination on the incidence of cervical cancer and associated mortality (ie, the time horizon was set at a point in time when all countries are at equilibrium, meaning that incidence and prevalence measures are constant over time).

We present all model predictions with the 10th and 90th percentiles (80% uncertainty interval) of the model predictions taken from the distribution of 1000 simulation results for each country (50 parameter sets × 20 simulations per parameter set), and the mean of the ten parameter sets that provide the best fit (using weighted least squares) to the estimated incidence of cervical cancer from Globocan 2020.^12^ The HPV-ADVISE LMIC model was implemented in C++ (version 11).

### Role of the funding source

WHO contributed to the study design, data collection, data analysis, data interpretation, and writing of the report. The Canadian Institute of Health Research, Fonds de recherche du Québec - Santé, The Bill & Melinda Gates Foundation, and Compute Canada had no role in the study design, data collection, data analysis, data interpretation, or writing of the report.

## Results

**Figure 1:**
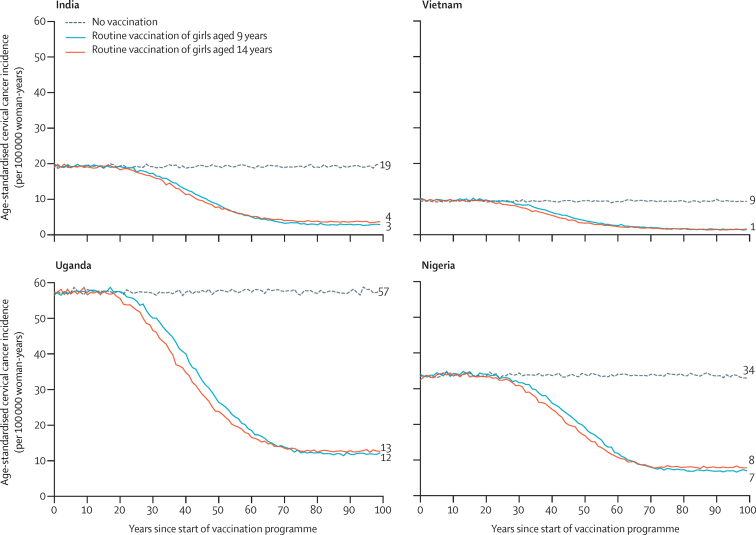
Predicted population-level impact of two-dose routine vaccination of girls aged 9 years *vs* aged 14 years in four low-income and middle-income countries Predicted data are the mean of the ten best fit parameter sets to Globocan 2020 estimated incidences of cervical cancer. Numbers to the right of the graphs indicate the incidence at equilibrium. Predictions were made under the base case assumptions.

All described analyses model vaccination with two doses of HPV vaccine, unless otherwise indicated. Under base case assumptions, we predicted that routine vaccination of girls only, with 80% coverage, would lead to substantial decreases in the incidence of cervical cancer (figure 1; appendix 4 pp 5–7). At equilibrium, routine vaccination of girls aged 9 years is predicted to reduce the age-standardised incidence per 100 000 woman-years of cervical cancer from 19 to three cases in India (ie, 85% effectiveness), from nine cases to one case in Vietnam (ie, 84% effectiveness), from 57 to 12 cases in Uganda (79% effectiveness), and from 34 to seven cases in Nigeria (79% effectiveness). Routine vaccination of girls aged 14 years (figure 1; appendix 4 p 5) would accelerate the decrease in incidence compared with vaccinating girls aged 9 years because they are closer to becoming sexually active and becoming exposed to HPV but is expected to produce a slightly lower decrease in age-standardised incidence in the long term (figure 1). The difference in incidence at equilibrium between routine HPV vaccination of girls aged 9 years and those aged 14 years is predicted to vary between less than one case per 100 000 woman-years in Vietnam (difference in effectiveness of <1%) and one case per 100 000 woman-years in the other LMICs (difference in effectiveness of 1–4%), due to differences in the proportion of girls who are infected before age 14 years.

**Figure 2:**
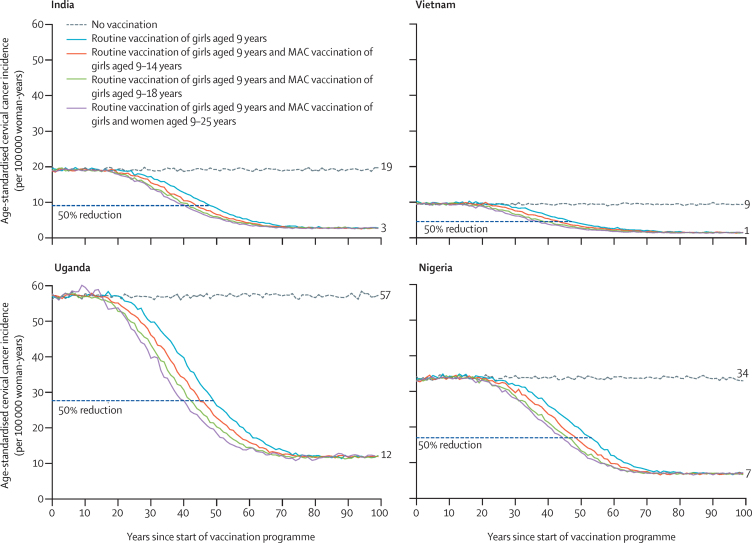
Predicted population-level impact of two-dose routine vaccination of girls aged 9 years, with MAC vaccination in the first year of the programme Girls older than 14 years are always given three doses. Predicted data are the mean of the ten best fit parameter sets to Globocan 2020 estimated incidences of cervical cancer. Numbers to the right of the graphs indicate the incidence at equilibrium. Predictions were made under the base case assumptions. MAC=multiple-age cohort.

Our model predicts that routine vaccination of girls aged 9 years, with vaccination of girls aged 9–14 years in the first year of the programme as part of a multiple-aged cohort vaccination programme combines the advantages of the long-term benefit of routine vaccination of girls aged 9 years and the faster decreases in incidence that were seen with routine vaccination of girls aged 14 years (figure 2). Vaccination of additional cohorts of girls or young women up to age 18 or 25 years would further accelerate decreases in the incidence of cervical cancer (figure 2; appendix 4 p 5) and therefore prevent a larger cumulative number of cases over time. However, in the long term, the strategy of vaccinating multiple older-age cohorts has no effect on the incidence of cervical cancer or whether elimination can be reached—ie, long-term incidence of cervical cancer is only affected by the routine strategy that is implemented (Figure 1, Figure 2).

**Figure 3:**
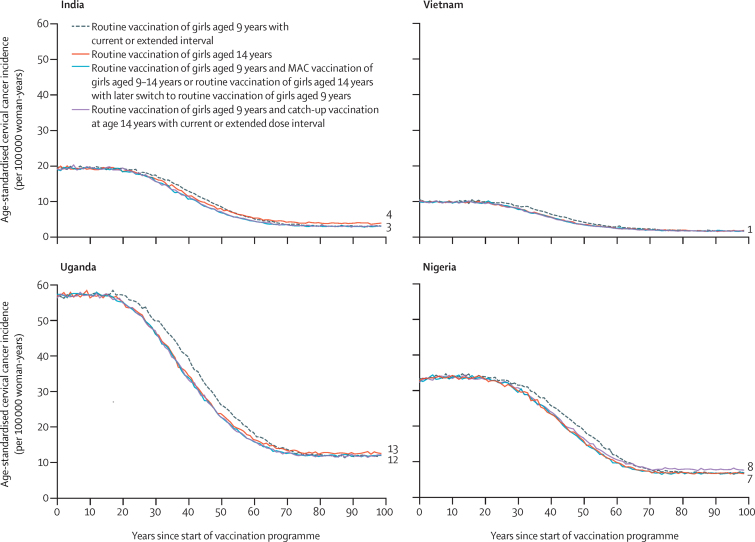
Predicted population-level impact of different two-dose HPV vaccination strategies targeting girls aged 9–14 years For the extended interval strategy, the first dose is given at age 9 years and the second dose at age 14 years. Predicted data are the mean of the ten best fit parameter sets to Globocan 2020 estimated incidences of cervical cancer. Numbers to the right of the graphs indicate the incidence at equilibrium. Predictions were made under the base case assumptions. MAC=multiple-age cohort.

Our model predicts very similar population-level effectiveness with the different vaccination strategies of girls aged 9–14 years (figure 3; appendix 4 pp 2, 25–26). The post-vaccination dynamics in age-standardised incidence of cervical cancer are predicted to be identical for the strategy of routine vaccination of girls aged 9 years using the current two-dose interval and that of routine vaccination of girls aged 9 years using the extended interval (ie, first dose at age 9 years and second dose at age 14 years), if the first dose and second dose have the same vaccine efficacy or vaccination coverage (figure 3; appendix 4 pp 25–26). The dynamics are also very similar for the following strategies: routine vaccination of girls aged 9 years with vaccination of girls aged 9–14 years in the first year of the programme, routine vaccination of girls aged 9 years with an extended interval of 5 years between doses and a catch-up campaign for girls aged 14 years, or routine vaccination of girls aged 14 years with a later switch to routine vaccination of girls aged 9 years (figure 3; appendix 4 pp 25–26).

**Figure 4:**
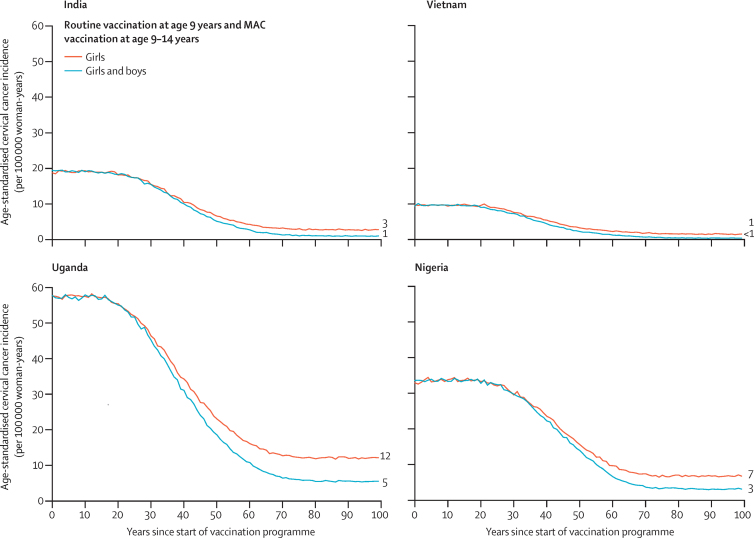
Predicted population-level impact of two-dose routine vaccination of girls *vs* girls and boys aged 9–14, with MAC vaccination in the first year of the programme Predicted data are the mean of the ten best fit parameter sets to Globocan 2020 estimated incidences of cervical cancer. Numbers to the right of the graphs indicate the incidence at equilibrium. Predictions were made under the base case assumptions. MAC=multiple-age cohort.

Vaccinating boys in addition to girls with two doses of vaccine both accelerates the decrease in age-standardised incidence of cervical cancer and further decreases the incidence at equilibrium (figure 4; appendix 4 pp 5–7). In the long term, gender-neutral vaccination with 80% vaccination coverage is predicted to decrease the age-standardised incidence per 100 000 woman-years of cervical cancer from 19 cases to one case in India (ie, 95% effectiveness), from nine cases to fewer than one case in Vietnam (96% effectiveness), from 57 to five cases in Uganda (91% effectiveness), and from 34 to three cases in Nigeria (90% effectiveness). Finally, at 80% vaccination coverage, gender-neutral vaccination with vaccination of boys and girls aged 9–14 years in the first year of the programme leads to a 90–96% reduction in HPV-16 and HPV-18 prevalence (*vs* 50–87% with girls-only vaccination with multiple-aged cohort vaccination of girls aged 9–14 years; appendix 4 pp 6–7).

**Figure 5:**
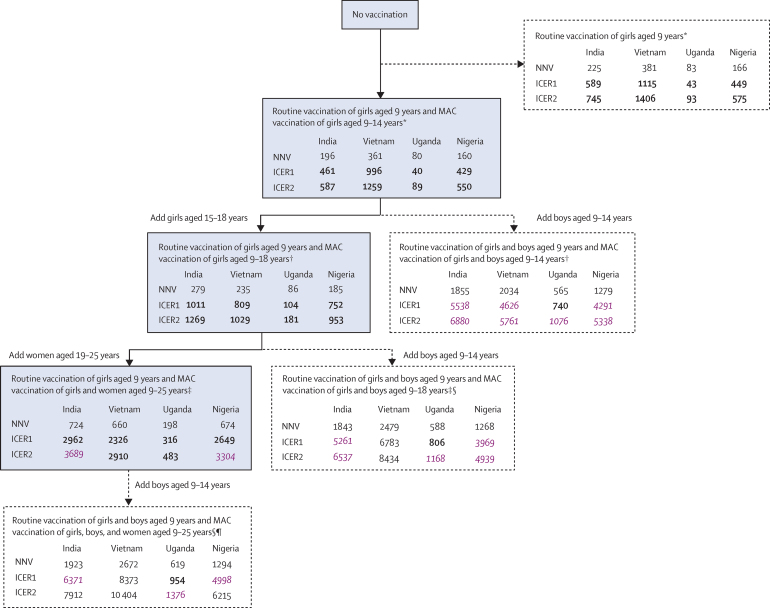
Predicted incremental efficiency and cost-effectiveness of different two-dose vaccination strategies varying the number of cohorts, age, and population targeted (girls only *vs* girls and boys) Strategies in the blue boxes are those that are incrementally more efficient and cost-effective when vaccinating additional cohorts of individuals (ie, the cost-effectiveness frontier). Dotted arrows and boxes indicate dominated strategies or extended dominated strategies (ie, strategies that were not the most efficient or cost-effective up the chain). ICERs that are bold are below 0·5 × GDP per capita (appendix 4 p 4). ICERs that are purple and italic are above 0·5 × GDP per capita but below GDP per capita. ICER1 is the scenario in which cost per vaccine dose, including administration costs, is INT$4·60. ICER2 is the scenario in which cost per vaccine dose, including administration costs, is INT$7·50. Routine vaccination occurs constantly, whereas MAC vaccination occurs in the first year of the vaccination programme only. Predictions were made under the base case assumptions. GDP=gross domestic product. ICER=incremental cost-effectiveness ratio per disability-adjusted life-year averted. INT$=international dollar for 2017. MAC=multiple age cohort. NNV=number of doses needed to prevent one case of cervical cancer. *The comparator was no vaccination. †The comparator was the strategy of routine vaccination of girls aged 9 years and MAC vaccination of girls aged 9–14 years. ‡The comparator was the strategy of routine vaccination of girls aged 9 years and MAC vaccination of girls aged 9–18 years. §For MAC vaccination of girls and boys, age of girls and women is as indicated and boys are aged 9–14 years in all instances. ¶The comparator was the strategy of routine vaccination of girls aged 9 years and MAC vaccination of girls and women aged 9–25 years.

Our model predicted that, compared with no vaccination, routine vaccination of girls aged 9 years, with additional vaccination of girls aged 9–14 years in the first year of the programme, was the most efficient and cost-effective vaccination strategy in all four countries, when varying simultaneously the number of cohorts, age of cohorts, and population targeted for vaccination. This strategy extendedly dominated routine vaccination of girls aged 9 years only (ie, was more costly but had lower ICER; figure 5; appendix 4 pp 8–9, 17–20). When examining the incremental efficiency and cost-effectiveness of vaccinating more individuals, either by increasing the number of age cohorts of girls vaccinated in the first year of introducing the vaccination programme (aged 15–18 years) or by vaccinating boys aged 9–14 years, our model predicted that vaccinating supplementary cohorts of girls would be optimal in all countries (figure 5). Compared with vaccinating girls aged 9–14 years, the NNV and incremental cost-effectiveness of extending vaccination to girls aged 15–18 years were similar (figure 5), and dominated vaccinating boys aged 9–14 years. Compared with vaccinating girls aged 9–18 years, vaccinating women up to age 25 years was estimated to be more efficient and cost-effective than adding vaccination of boys. Finally, strategies that involved vaccinating boys were the least efficient and cost-effective, with NNVs to prevent one case of cancer being six to eight times higher than when vaccinating girls and women up to age 25 years. However, ICERs remained below the GDP per capita threshold for all strategies.

**Figure 6:**
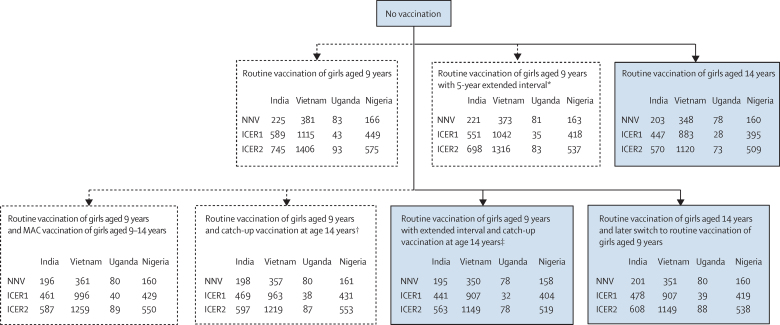
Predicted efficiency and cost-effectiveness of different two-dose HPV vaccination strategies targeting girls aged 9–14 years (all strategies are compared with no vaccination) Strategies in the blue boxes are those that are incrementally more efficient and cost-effective when vaccinating additional cohorts of girls. Dotted arrows and boxes indicate dominated strategies or extended dominated strategies (ie, strategies that were not the most efficient or cost-effective up the chain). All ICERs in this figure are below 0·5 GDP per capita (appendix 4 p 4). ICER1 is the scenario in which cost per vaccine dose, including administration costs, is INT$4·60. ICER2 is the scenario in which cost per vaccine dose, including administration costs, is INT$7·50. Routine vaccination occurs constantly, whereas MAC vaccination occurs in the first year of the vaccination programme only. Predictions were made under the base case assumptions. GDP=gross domestic product. ICER=incremental cost-effectiveness ratio per disability-adjusted life-year averted. INT$=international dollar for 2017. MAC=multiple age cohort. NNV=number of doses needed to prevent one case of cervical cancer. *First dose at age 9 years and second dose at age 14 years (assuming the same coverage). †Two doses at age 9 years and a 5-year catch-up at age 14 years. ‡First dose at age 9 years and second dose at age 14 years (assuming the same coverage) plus a 5-year catch-up vaccination of girls aged 14 years.

When examining different vaccination strategies of girls aged 9–14 years, our model predicted that, compared with no vaccination, all vaccination strategies were highly efficient and cost-effective and led to very similar NNVs and ICERs (figure 6; appendix 4 pp 10–11, 17–20). Nonetheless, our model predicted that routine vaccination of girls aged 14 years, with or without a later switch to routine vaccination of girls aged 9 years, and routine vaccination of girls aged 9 years with an extended interval between doses and catch-up vaccination at age 14 years were the most efficient and cost-effective strategies.

Sensitivity analysis examining population-level impact, the NNV to prevent one case of cancer, and cost-effectiveness of HPV vaccination strategies for different numbers of doses and vaccines used are presented in appendix 4 (pp 12–16, 32–35). Compared with no vaccination, one-dose vaccination of girls aged 9–14 years is highly efficient and cost-effective. When assuming one-dose vaccine-type efficacy is greater than 85% and duration of protection is lifelong, vaccinating older cohorts of girls, women, and boys would be more efficient and cost-effective than giving a second dose to girls aged 9–14 years in all countries (appendix 4 pp 14–16, 34–35). However, if one dose has a shorter duration of protection (eg, 20 years), then the strategy of giving a second dose to girls aged 9–14 years and the strategy of adding the vaccination of older girls or women (with three doses for older girls and women) would be equally efficient and cost-effective as each other (appendix 4 pp 12–13, 34–35).

Compared with no vaccination, our model predicts very similar ICERs for the different vaccines (appendix 4 pp 21–22). Furthermore, the findings of the most efficient and cost-effective strategies do not change according to the vaccine that is used (data not shown). Finally, if the two-valent or four-valent vaccines have no cross-protection against non-vaccine types, the use of the nine-valent vaccine costing $2·90 more than the two-valent or four-valent vaccine would be cost-effective (ICERs between $430 per DALY averted for Uganda and $3875 per DALY averted for India; appendix 4 p 22). However, if the two-valent or four-valent vaccines have high cross-protection, ICERs are higher for Vietnam, Uganda, and Nigeria than ICERs that assume no cross-protection with the two-valent or four-valent vaccines (appendix 4 p 22).

## Discussion

Of all HPV vaccination strategies examined with two doses, those that involved vaccinating girls aged 9–14 years are predicted to be the most efficient and cost-effective in the four LMICs examined. Among these strategies, routine vaccination of girls aged 14 years with or without a later switch to routine vaccination of girls aged 9 years and routine vaccination of girls aged 9 years with an extended interval of 5 years between doses and a catch-up programme for girls aged 14 years are predicted to be the most efficient and cost-effective strategies. Compared with no vaccination, these strategies produce NNVs between 78 and 351 and ICERs between $28 per DALY averted and $1149 per DALY averted depending on the country and vaccine price. Vaccination strategies that included boys or older cohorts of women were substantially less efficient and cost-effective than vaccination strategies that included girls aged 9–18 years only. Finally, HPV vaccination of girls only and boys and girls with high coverage could lead to elimination of cervical cancer (ie, less than four cases per 100 000 woman-years) in countries with incidences of cervical cancer between those of Vietnam (age-standardised incidence of cervical cancer of nine cases per 100 000 woman-years) and Nigeria (34 cases per 100 000 woman-years), but not in countries with an incidence similar to, or higher than, that of Uganda (57 cases per 100 000 woman-years), despite such countries potentially achieving substantial reductions in the incidence of cervical cancer. In countries like Uganda, adding cervical screening to female or gender-neutral vaccination will likely be required to reach elimination. Indeed, one modelling study suggested that the addition of twice lifetime HPV screening at ages 35 years and 45 years to HPV vaccination is needed for elimination of cervical cancer in countries with high incidences.^33^ WHO has created the WHO Global Cervical Cancer Elimination Modelling Consortium to identify the most efficient and cost-effective combinations of HPV vaccination, cervical screening, and treatment likely to lead to elimination.^34^, ^35^

Our findings have important implications for HPV vaccination policy decisions in LMICs in the context of limited supplies of vaccine. Our results suggest that the most efficient HPV vaccination strategies, as measured by NNV, are those that target girls aged 9–14 years. By contrast, vaccinating older girls and women or boys is much less efficient, with NNVs that are up to ten times higher than when vaccinating girls aged 9–14 years. Partly based on these results, in October, 2019, SAGE recommended that countries should temporarily postpone the implementation of gender-neutral vaccination and vaccination of girls and women in older age groups (≥15 years), at least until HPV vaccine supply shortages are resolved.^10^ Additionally, SAGE recommended that multiple-age cohort vaccination of girls aged 9–14 years, which requires a large number of doses during the first year of the programme, should also be postponed to alleviate the demand for vaccine doses in the coming years. The WHO recommended alternative strategies are variations on the optimal strategies identified in our analysis—ie, routine two-dose vaccination of girls aged 13 or 14 years, with or without a later switch to routine vaccination in younger girls (ie, aged 9 or 10 years) and routine vaccination at age 9 or 10 years with an extended interval of 3–5 years between doses combined with catch-up programme for girls aged 14 years. Given that the recommended vaccination strategies have very similar NNVs and ICERs, small changes in vaccination coverage or age of sexual debut could lead to changes in the ranking of the optimal strategy. Hence, the choice of strategy should be country-specific and will need to consider issues of programmatic feasibility and acceptability and the potential to obtain the greatest vaccination coverage before girls become sexually active.

Our results suggest that routine vaccination of girls aged 13 or 14 years, with or without a later switch to routine vaccination at an earlier age (ie, 9 or 10 years), would allow countries to retain the accelerated impact of vaccinating multiple-age cohorts while restricting the number of doses required in the first years after programme implementation. Compared with routine vaccination of girls aged 9 years, routine vaccination of girls aged 14 years has two main advantages: it accelerates health benefits by targeting girls closer to becoming sexually active, and it targets girls just before they age out of the 9–14 years age category (these girls would be missed with routine vaccination at younger ages). However, the population-level effectiveness of routine vaccination of girls aged 13 or 14 years (*vs* routine vaccination of girls aged 9 years or multiple-age cohort vaccination of girls aged 9–14 years) will depend on the proportion of girls who are sexually active before the age of vaccination and whether sufficiently high vaccination coverage can be achieved. We found that the long-term population-level effectiveness of routine vaccination of girls aged 14 years would be 3–4% percentage points lower than routine vaccination of girls aged 9 years in countries such as Nigeria and India, which have a relatively high proportion of girls aged 14 years who are sexually active (15–25%).^36^, ^37^ Notably, even in countries with a high proportion of girls who are sexually active at a relatively young age, the difference in estimated population-level effectiveness between strategies remains small probably because of herd immunity from vaccination (ie, reducing the risk of infection among unvaccinated girls who are sexually active by reducing the circulation of HPV in this population). In countries where school attendance substantially decreases with older age, vaccinating girls at age 13 years (or the oldest age at which school attendance is achieved) rather than at age 14 years could be considered to improve coverage. Importantly, routine vaccination of girls aged 13 or 14 years with a later switch to routine vaccination at age 9 or 10 years would probably be the optimal strategy if vaccination coverage is not as high as expected or a high proportion of girls are sexually active before age 14 years, or both. Such a strategy would allow a country to benefit from the short-term advantages of vaccinating older girls (accelerated benefits and minimising the number of missed vaccine cohorts) and long-term benefits of vaccinating younger girls (potentially increased coverage and vaccination before girls become sexually active).

Routine vaccination of girls aged 9 years with a 5-year extended interval between doses combined with a catch-up programme for girls aged 14 years was predicted to provide the same accelerated benefits and long-term population-level effectiveness as multiple age-cohort vaccination of girls aged 9–14 years in the first year of the programme, but with fewer vaccine doses required in the short term and lower NNVs and ICERs. An important advantage of this extended-interval vaccination strategy is that it offers countries the opportunity to not give a second dose if the results from single-dose vaccine efficacy randomised clinical trials are positive.^38^ Indeed, post-hoc analysis of randomised trials and post-vaccination surveillance studies suggest that single-dose HPV vaccination could provide protection against HPV infections and related diseases.^39^ The vaccine efficacy and duration of protection of one dose of HPV vaccine is unknown, but results of randomised trials are expected in the next 2–5 years. If a second dose is not deemed to be required in the next 5 years, our results predict that countries that introduce extended-interval dosing programmes will have implemented the most efficient and cost-effective vaccination strategy, and saved substantial medical costs. However, if two doses of HPV vaccine remain necessary, maintaining high vaccination coverage for the second dose at age 13 or 14 years will be important. If the extended-interval vaccination strategy also includes catch-up vaccination at age 14 years, countries can have time to implement programmes to increase vaccination coverage in that age group. Additionally, although not included in our model assumptions, an extended interval would allow girls who missed their first dose at age 9 years to be vaccinated at age 14 years. Furthermore, little is known about the vaccine efficacy of 3–5 year extended-interval schedules, and such a strategy would be considered off-label use of the vaccine. To our knowledge, the only study that has examined this interval found that extended intervals resulted in similar levels of geometric mean IgG antibody titres when the second dose was given 6 months or 3–8 years after the first dose.^40^ Finally, WHO recommended that countries consider routine vaccination of girls aged 9 years with a 3–5 year extended interval between doses (with a catch-up programme at age 14 years) after careful consideration of its programmatic challenges, and with a clear and well planned communication strategy.^10^

Our study has five major strengths. First, to our knowledge, this is the first comprehensive modelling analysis to simultaneously compare HPV vaccination strategies with different ages at vaccination, numbers of age cohorts targeted, gender targeted, numbers of doses, and intervals between doses. Second, our model was calibrated to LMIC-specific sexual activity and epidemiological data. We selected two Asian and two African countries, representing different profiles in terms of sexual activity and HPV-related burden, for which good-quality data were available for us to calibrate and validate our model. Even though these countries are very different in terms of number of lifetime partners and incidence of cervical cancer, the optimal HPV vaccination strategies in terms of efficiency and cost-effectiveness were consistent. Hence, our results are probably generalisable to most LMICs, except potentially LMICs in the Middle East and north Africa, which have a lower incidence of cervical cancer than other LMICs. Third, our model predictions are based on 50 parameter sets, capturing uncertainty in key parameters and within-country variability in sexual activity and HPV and cervical cancer epidemiology. Fourth, our model predictions, which suggested that all vaccination strategies for girls aged 9–14 years we investigated are highly efficient and cost-effective, are robust when using a different number of doses (one dose *vs* two dose) or type of vaccine (two-valent, four-valent, or nine-valent). Finally, we developed these vaccination strategies for girls aged 9–14 years in collaboration with the WHO HPV Working group to ensure that our modelling results were responsive to the needs of policy makers.

Our study also has some limitations. First, although we considered seven different vaccination strategies of girls aged 9–14 years, we did not model all possible combinations of ages at vaccination and vaccination coverage. For this reason, our results should be considered as general principles guiding HPV vaccination policy decisions in the different countries. For example, the most efficient strategies are those initially vaccinating the oldest age cohort before they became sexually active (through routine vaccination or catch-up to accelerate health benefits) and long-term routine vaccination at a younger age (to maximise coverage and minimise the likelihood of vaccinating previously infected girls). Second, our model does not account for the effect of HIV, which increases the likelihood of acquisition of HPV and disease progression, or the effect of HIV treatment, which might attenuate the effects of HIV on HPV acquisition and disease progression. Hence, the effect of HPV vaccination on the incidence of cervical cancer might be overestimated in settings with a high prevalence of HIV or might mean that specific prevention strategies might be required for people living with HIV to enable elimination of cervical cancer. These questions are being examined as part of the WHO Cervical Cancer Elimination Modeling Consortium.^30^

In summary, HPV vaccination strategies that target girls aged 9–14 years are the most optimal for use of scarce resources. In the context of limited supply of HPV vaccines and the COVID-19 pandemic, two-dose routine vaccination of girls aged 13 or 14 years, with or without a later switch to routine vaccination at an earlier age and routine vaccination at age 9 or 10 years with an extended interval of 3–5 years between doses combined with a programme of catch-up vaccination of girls aged 14 years are the strategies that would minimise the number of doses required in the short term for maximum prevention of cervical cancer. These strategies would allow the maximum number of countries to introduce HPV vaccination and could reduce the effect of vaccine supply shortage on efforts to eliminate cervical cancer.

## Data sharing

No individual participant-level data were used in this study. Descriptions of the model structure, the parameters included in the model, and the empirical data used for calibration and validation are available in appendix 3.

## Declaration of interests

We declare no competing interests.

## Supplementary Materials

French translation of the abstract

Spanish translation of the abstract
