# Supplementary material for: Optimal human papillomavirus vaccination strategies to prevent cervical cancer in low-income and middle-income countries in the context of limited resources: a mathematical modelling analysis
Source: Lancet Infect Dis. 2021 Nov;21(11):1598–610. doi: 10.1016/S1473-3099(20)30860-4 (PMC8554391; doi:10.1016/S1473-3099(20)30860-4)
Supplement: French translation of the abstract [file mmc1.pdf]

THE LANCET
Infectious Diseases

Supplementary appendix 1

This translation in French was submitted by the authors and we reproduce it as supplied. It has not been peer reviewed. *The Lancet*'s editorial processes have only been applied to the original in English, which should serve as reference for this manuscript.

Cette traduction en français a été proposée par les auteurs et nous l'avons reproduite telle quelle. Elle n'a pas été examinée par des pairs. Les processus éditoriaux du *Lancet* n'ont été appliqués qu'à l'original en anglais et c'est cette version qui doit servir de référence pour ce manuscrit.

Supplement to: Drolet M, Laprise J-F, Martin D, et al. Optimal human papillomavirus vaccination strategies to prevent cervical cancer in low-income and middle-income countries in the context of limited resources: a mathematical modelling analysis. *Lancet Infect Dis* 2021; published online July 7. https://doi.org/10.1016/S1473-3099(20)30860-4.

Appendix 1

Stratégies optimales de vaccination contre les virus du papillome humain pour prévenir le cancer du col de l'utérus dans les pays à revenu faible et intermédiaire dans un contexte de ressources limitées : une analyse de modélisation mathématique

Mise en contexte L'introduction de la vaccination contre les virus du papillome humain (VPH) est ralentie dans les pays à revenu faible et intermédiaire (PRFI) en raison des contraintes de ressources et de la pénurie mondiale de vaccins contre les VPH. Afin de guider les recommandations de l'OMS, nous avons modélisé différentes stratégies de vaccination pour examiner l'utilisation optimale des doses de vaccins et la meilleure allocation de ressources limitées dans les PRFI, dans le contexte de l'appel mondial de l'OMS pour éliminer le cancer du col de l'utérus comme problème de santé publique.

Méthodologie Pour cette analyse de modélisation mathématique, nous avons développé HPV-ADVISE LMIC, un modèle dynamique de transmission de l'infection aux VPH et des maladies liées aux VPH. Le modèle a été calibré à quatre PRFI : l'Inde, le Vietnam, l'Ouganda et le Nigéria. Pour différentes stratégies de vaccination avec le vaccin nonavalent (ou les vaccins quadrivalent et bivalent en faisant l'hypothèse d'une protection croisée élevée), nous avons estimé trois issues principales : 1) la réduction du taux de cancer du col de l'utérus standardisé pour l'âge, 2) l'efficience en utilisant le nombre nécessaire de personnes à vacciner (NNV) pour prévenir un cas de cancer du col de l'utérus, défini comme étant le nombre de dose à administrer pour prévenir un cas de cancer du col de l'utérus, et 3) le ratio coût-efficacité incrémental (RCEI, en dollars international 2017 par année de vie corrigée de l'incapacité (AVCI) prévenue). Pour les différentes stratégies, nous avons fait varier l'âge de la vaccination de routine, le nombre de cohortes et l'âge des cohortes ciblées, la population ciblée (filles seulement, garçons et filles) et le nombre de doses utilisées. Pour notre scénario de base, nous avons fait les hypothèses d'une protection vaccinale à vie de 100% contre les VPH-16, VPH-18, VPH-31, VPH-33, VPH-45, VPH-52 et VPH-58, d'une couverture vaccinale de 80% et d'un horizon temporel de 100 ans. Pour l'analyse coût-efficacité, nous avons utilisé un taux d'actualisation de 3%. L'élimination du cancer du col de l'utérus était définie comme un taux de cancer du col de l'utérus standardisé pour l'âge de moins de quatre cas par 100 000 femmes-années.

Résultats Notre modèle a prédit que la vaccination contre les VPH pourrait mener à l'élimination du cancer du col de l'utérus au Vietnam, en Inde et au Nigéria, mais pas en Ouganda. En comparaison avec l'absence de vaccination, les stratégies de vaccination des filles de 9 à 14 ans avec deux doses étaient les plus efficientes et coût-efficaces dans les quatre PRFI selon les prédictions de notre modèle. Les NNV pour prévenir un cas de cancer de col de l'utérus variaient de 78 à 381 et les RCEI variaient de 28 $ à 1406 $ par AVCI prévenue, dépendamment du pays. Les stratégies les plus efficientes et coût-efficaces étaient la vaccination de routine des filles de 14 ans, avec ou sans changement éventuel à la vaccination de routine des filles de 9 ans et la vaccination de routine des filles de 9 ans, avec un calendrier étendu de 5 ans entre les 2 doses, combinée à une campagne de rattrapage des filles de 14 ans. La vaccination des garçons (âgées de 9 à 14 ans) ou des femmes de 18 ans ou plus produisaient des NNV et des RCEI substantiellement plus élevés.

Interprétation Nous avons identifié deux stratégies qui pourraient maximiser les efforts de prévention du cancer du col de l'utérus dans les PRFI, en considérant les contraintes liées à la pénurie et au coût des vaccins, et qui permettraient à un maximum de PRFI d'introduire la vaccination contre les VPH.
