# Supplementary material for: Optimal human papillomavirus vaccination strategies to prevent cervical cancer in low-income and middle-income countries in the context of limited resources: a mathematical modelling analysis
Source: Lancet Infect Dis. 2021 Nov;21(11):1598–610. doi: 10.1016/S1473-3099(20)30860-4 (PMC8554391; doi:10.1016/S1473-3099(20)30860-4)
Supplement: Spanish translation of the abstract [file mmc2.pdf]

THE LANCET
Infectious Diseases

Supplementary appendix 2

This translation in Spanish was submitted by the authors and we reproduce it as supplied. It has not been peer reviewed. *The Lancet*'s editorial processes have only been applied to the original in English, which should serve as reference for this manuscript.

Los autores nos proporcionaron esta traducción al español y la reproducimos tal como nos fue entregada. No la hemos revisado. Los procesos editoriales de *The Lancet* se han aplicado únicamente al original en inglés, que debe servir de referencia para este manuscrito.

Supplement to: Drolet M, Laprise J-F, Martin D, et al. Optimal human papillomavirus vaccination strategies to prevent cervical cancer in low-income and middle-income countries in the context of limited resources: a mathematical modelling analysis. *Lancet Infect Dis* 2021; published online July 7. https://doi.org/10.1016/S1473-3099(20)30860-4.

Appendix 2

Estrategias óptimas de vacunación contra el virus del papiloma humano para prevenir el cáncer cervicouterino en países de bajos y medianos ingresos en contexto de recursos limitados: un análisis de modelación matemática

Resumen

Antecedentes La introducción de la vacunación contra el virus del papiloma humano (VPH) ha sido lenta en los países de bajos y medianos ingresos (PBMIs) debido a las limitaciones de recursos y a la escasez mundial de suministros de vacunas. Con el objetivo de aportar información a las recomendaciones de la OMS, modelamos varias estrategias de vacunación contra el VPH, con el fin de examinar el uso óptimo de los suministros de vacunas limitados y mejorar la distribución de los recursos escasos en los PBMIs en el contexto del llamado mundial de la OMS para la eliminación del cáncer cervicouterino como un problema de salud pública.

Métodos En este análisis de modelación matemática, desarrollamos HPV-ADVISE-LMIC, un modelo de transmisión dinámica de la infección y enfermedad por VPH, calibrado para cuatro PBMIs: India, Vietnam, Uganda y Nigeria. Para las estrategias de vacunación diferentes que abarcan el uso de la vacuna nonavalente (o vacunas bivalente y tetravalente con un supuesto de protección cruzada alta), estimamos tres resultados: la reducción en la tasa de cáncer cervicouterino estandarizada por edad; la eficiencia, utilizando la medida del número necesario de personas a vacunar (NNV) para evitar un caso de cáncer cervicouterino, definido como el número necesario de dosis para evitar un caso de cáncer cervicouterino; y la razón incremental de costo-efectividad (RICE; en 2017 $ [INT$] internacional por año de vida ajustado por discapacidad [AVAD] evitado). Las diferentes estrategias variaron según la edad de la vacunación de rutina contra el VPH, el número de cohortes de edades vacunadas, la población objetivo (solo niñas o niños y niñas), y el número de dosis utilizadas. En nuestro caso de base, asumimos una protección de por vida del 100% contra HPV-16, HPV-18, HPV-31, HPV-33, HPV-45, HPV-52 y HPV-58; una cobertura de vacunación del 80%; y una perspectiva temporal de 100 años. Para el análisis de costo-efectividad, usamos una tasa de descuento del 3%. La eliminación del cáncer cervicouterino se definió como una tasa de incidencia estandarizada por edad de menos de cuatro casos por 100 000 mujeres-año.

Resultados Se proyecta que la vacunación rutinaria contra el VPH conduciría a la eliminación del cáncer cervicouterino en Vietnam, India y Nigeria, pero no en Uganda. Comparado con la ausencia de vacunación, se proyecta que las estrategias de vacunación con un régimen de dos dosis que incluyen a las niñas de 9 a 14 años serían las más eficientes y costo-efectivas en los cuatro PBMIs. El NNV para evitar un caso de cáncer cervicouterino varió entre 78 y 381 y la RICE osciló entre $28 y $1406 por AVAD evitado, según el país. Las estrategias más eficientes y costo-efectivas fueron la vacunación rutinaria de las niñas de 14 años, con o sin un cambio posterior a la vacunación rutinaria de las niñas de 9 años, y la vacunación rutinaria de las niñas de 9 años con un intervalo extendido de 5 años entre las dosis y un programa de vacunación de rescate para niñas de 14 años. Se obtuvieron NNVs y RICEs sustancialmente más altos vacunando niños (de 9 a 14 años) o mujeres de 18 años o mayores.

Interpretación Se identificaron dos estrategias que podrían maximizar los esfuerzos para prevenir el cáncer cervicouterino dadas las limitaciones en el suministro y el costo de las vacunas, y esto permitiría que un mayor número de PBMIs introduzcan la vacunación contra el VPH.
