# Supplementary material for: Optimal human papillomavirus vaccination strategies to prevent cervical cancer in low-income and middle-income countries in the context of limited resources: a mathematical modelling analysis
Source: Lancet Infect Dis. 2021 Nov;21(11):1598–610. doi: 10.1016/S1473-3099(20)30860-4 (PMC8554391; doi:10.1016/S1473-3099(20)30860-4)
Supplement: Supplementary appendix 3 [file mmc3.pdf]

THE LANCET
Infectious Diseases

Supplementary appendix 3

This appendix formed part of the original submission and has been peer reviewed. We post it as supplied by the authors.

Supplement to: Drolet M, Laprise J-F, Martin D, et al. Optimal human papillomavirus vaccination strategies to prevent cervical cancer in low-income and middle-income countries in the context of limited resources: a mathematical modelling analysis. *Lancet Infect Dis* 2021; published online July 7. https://doi.org/10.1016/S1473-3099(20)30860-4.

TECHNICAL APPENDIX
HPV-ADVISE LMIC

Brisson, Marc
Laprise, Jean-François
Martin, Dave
Drolet, Mélanie
Van de Velde, Nicolas
Boily, Marie-Claude

© Marc Brisson, Marie-Claude Boily, 2017-2020.

Table of content

TABLE OF CONTENT II

LIST OF FIGURES III

LIST OF TABLES IV

1 MODEL STRUCTURE 1

1.1 Demography 1

1.2 Sexual behavior and HPV Transmission 1
1.2.1 Sexual activity levels 1
1.2.2 Partnership formation and separation process 1
1.2.3 Contact/Network structure 2

1.3 Natural History of HPV-related diseases 4
1.3.1 Cervical cancer 4

1.4 Screening 5
1.4.1 Screening behavior levels 5
1.4.2 Screening performance for the detection of cervical lesions 5

2 MODEL PARAMETERIZATION 6

2.1 Calibration procedure 6

2.2 Parameters 9
2.2.1 Demographic parameters 13
2.2.2 Sexual Behavior Parameters 13
2.2.3 Biological Parameters 35
2.2.4 Screening Parameters 44

2.3 Model fit 47
2.3.1 Fit to sexual behavior data 47
2.3.2 Fit to HPV prevalence data 48
2.3.3 Fit to cervical cancer incidence data 50
2.3.4 Fit to HPV types distribution in cervical cancers 51

2.4 Model validation 52

2.5 Target definition 56

REFERENCES 57

List of figures

Figure A1. Flow diagram of the natural history of HPV infection and cervical cancer in the absence of screening 5
Figure A2. Sexual activity level distribution - Posterior distributions 22
Figure A3. Proportion of females who ever had sex by age and sexual activity level - Posterior distributions 24
Figure A4. Number of new partners in the past 12 months. 28
Figure A5. Proportion of contacts that lead to stable partnerships - Posterior distributions 30
Figure A6. Stable partnership separation rates - Posterior distributions 31
Figure A7. Number of sex acts per week in stable partnerships - Posterior distributions 31
Figure A8. Number of sex acts per casual partnership - Posterior distributions 32
Figure A9. Assortative degree of the mixing between levels of sexual activity - Posterior distributions33
Figure A10. Estimated age mixing by age and sexual activity level for each modeled country. 34
Figure A11. Per-act transmission probabilities - Posterior distributions 36
Figure A12. HPV-16 clearance rates - Posterior distributions 37
Figure A13. Relative clearance rates compared to HPV-16 - Posterior distributions 37
Figure A14. Probabilities of developing lifelong natural immunity following clearance of infection - Posterior distributions 38
Figure A15. Proportion of regressing CIN1 that clear HPV infection - Posterior distribution 39
Figure A16. Progression rates from infected to CIN1 - Posterior distribution 39
Figure A17. Clearance rates from CIN1 - Posterior distribution 40
Figure A18. Progression rates from CIN1 to CIN2 - Posterior distribution 40
Figure A19. Regression rates from CIN2 to CIN1 - Posterior distribution 41
Figure A20. Clearance rates from CIN2 - Posterior distribution 41
Figure A21. Progression rates from CIN2 to CIN3 - Posterior distribution 42
Figure A22. Regression rates from CIN3 to CIN2 - Posterior distribution 42
Figure A23. Progression rates from CIN3 to CC1 - Posterior distribution 43
Figure A24. Fit to proportion of sexually active women 47
Figure A25. Fit to HPV-16 and 18 prevalence among sexually active females 48
Figure A26. Fit to high risk HPV prevalence among sexually active females 49
Figure A27. Fit to incidence of cervical cancer 50
Figure A28. Fit to proportion of cervical cancers caused by HPV-types 16, 16/18, cross-protective types (31, 33, 45, 52, and 58), and not cross-protective (35, 39, 51, 56, 59, 66, 68, 73, and 82) 51
Figure A29. Proportion of sexually active males 52
Figure A30. Distribution of the number of partners in the past 12 months 53
Figure A31. Mean lifetime number of partners among sexually active females 55
Figure A32. Mean age of clients of female sex workers (FSW) 56

List of tables

Table A1. Description of calibration data 8
Table A2. List of model parameters 9
Table A3. List of studies used to model sexual behavior 14
Table A4. Data sources for sexual activity levels 21
Table A5. Data sources for onset of sexual activity (% ever sex females) 23
Table A6. Data sources for partner acquisition rates 26
Table A7. Data sources for proportion of new partnerships that lead to stable partnerships in females and males 29
Table A8. Data sources for separation rates 29
Table A9. Data sources for age mixing by age and sexual activity levels 33
Table A10. Proportion of women screened for cervical cancer and onset of cervical cancer screening in each modeled country 44
Table A11. Probabilities of detecting a neoplastic state by cytology 45
Table A12. Probabilities of diagnosing a neoplastic state by colposcopy/biopsy 46
Table A13. Parameters for the management of women with a first or repeated abnormal cytology result, according to the severity of the result 46

1 Model structure

The HPV-ADVISE LMIC platform structure is very similar to HPV-ADVISE Canada (Van de Velde 2010[1], Van de Velde 2012[2], Brisson 2013[3], HPV-ADVISE Canada Technical Appendix[4]) and HPV-ADVISE US (Brisson 2014[5], HPV-ADVISE US Technical Appendix[6]). HPV-ADVISE LMIC platform currently models 5 countries separately: India, Vietnam, Uganda, Nigeria, and Benin. Because of the potential impact of female sex workers (FSW) as a core group and their clients as a bridge population on HPV dynamics, we have included these groups in HPV-ADVISE LMIC.

1.1 Demography

The populations modeled represent the heterosexual population specific to each modeled country. We assume open stable populations. Ten-year-old individuals enter the population (with a 1:1 male to female ratio) at a rate chosen to balance country- and age-specific death rates. The equilibrium age distribution of the population is found by running the demographic model (i.e. model without HPV infection) prior to calibration. Individuals younger than 10 years old are not included in the model because they have a very low prevalence of sexually acquired HPV infection. See details on demographic parameters in Section 2.2.1.

1.2 Sexual behavior and HPV Transmission

1.2.1 Sexual activity levels

Upon entry in the simulated population, 10-year-olds are assigned to 1 of 4 levels of sexual activity from low (L0) to high (L3). See Section 2.2.2 for the definition of each level and for the posterior distributions of the fractions of individuals assigned to each level. 10-year-old girls are assumed to begin sexual activity at a rate that depends on their age and level of sexual activity. A specific partner acquisition rate (i.e., number of new partner acquisitions per year) is then attributed to each sexual activity level by age (see Section 2.2.2 for details).

1.2.2 Partnership formation and separation process

The model is based on a stochastic pair formation and separation process, which represents the underlying structure of the sexual contact pattern. We model sequential monogamous stable and casual (instantaneous) partnerships, as well as casual sexual partnerships between FSW and men in stable partnerships or single. The partnership formation and separation process is driven by females. Each woman has an associated age and level of sexual activity specific rate of either forming a new partnership if they are single, or separating if they are currently involved in a stable partnership. When a new partnership is formed, the male partner is selected according to an age and level of sexual activity specific mixing matrix, which reflects the preferences of a woman to form partnerships with

men given their respective age and level of sexual activity (see section 1.2.3 for details on the mixing matrices). All newly formed partnerships have an age and level of sexual activity specific probability of being stable (see details in Section 2.2.2).

The partnership formation rates of single females is derived from the partner acquisition rates and the age and level of sexual activity specific proportions of stable partnerships taking into account the proportions of individuals not available for partnership formation as follows:

$$\boldsymbol{\zeta_l(a) = \frac{\theta_{g,l}(a)}{\left(1-\Psi_l(a)\right)}} \tag{1.1}$$

$\zeta_l(a)$:	partnership formation rates of single females
$\theta_{g,l}(a)$:	partner acquisition rates
$\Psi_l(a)$:	proportion of stable partnerships
g:	gender
a:	age
l:	sexual activity level

1.2.3 Contact/Network structure

Mixing by sexual activity level

The sexual activity mixing matrix defines the probability that an individual of given gender and level of sexual activity forms a partnership with someone of the opposite gender with a given level of sexual activity. The matrix is computed as follows (Boily 1991[7]):

$$\Gamma_{l,l',g} = \frac{W_{l,l',g}\sum_{a'}\left\{N_{l',g'}(a')\cdot\theta_{g',l'}(a')\right\}}{\sum_{l'}\left\{W_{l,l',g}\sum_{a'}\left[N_{l',g'}(a')\cdot\theta_{g',l'}(a')\right]\right\}} \tag{1.2}$$

$\Gamma_{l,l',g}$:	sexual activity level mixing matrix
$N_{l,g}(a)$:	number of individuals of gender g, sexual activity level l and age group a
$\theta_{g,l}(a)$:	mean rate of sexual partner acquisition for gender g, sexual activity level l and age group a

$W_{l,l',g}$: weights corresponding to the preference of an individual of gender g and sexual activity level l for someone of the opposite gender with sexual activity level l' (preference matrix)

g: gender

a: age group of individual of gender g

a': age group of opposite gender partner

l: sexual activity level of individual of gender g

l': sexual activity level of opposite gender partner

Detailed data on each element of the mixing matrix by degree is rarely available and therefore, the preference matrix $W_{l,l',g}$ is often summarized by an assortative degree parameter (See Section 2.2.2) (Boily 1991[7]). The preference matrix is therefore defined as follows:

		**Males**			
		L0	L1	L2	L3
**Females**	L0	κ	1	1	κ
	L1	1	κ	1	κ
	L2	1	1	κ	κ
	L3	0	0	0	κ

(1.3)

κ: assortative degree parameter (where $\kappa > 1$ represents assortative mixing, $\kappa = 1$ is proportionate mixing and $\kappa < 1$, disassortative mixing)

We have 4 levels of sexual activity; the 4th level (L3) represents women who are sex workers (FSW), and men who are their clients (L3 men can concurrently be in partnership with a FSW and a L0, L1 or L2 woman). By having such categories, we can directly parameterize the percent of the female population that are FSW, and percent of the male population that are clients based on observed data.

Mixing by age

The age mixing matrix is specific to each country. The mixing matrix, $\Lambda_{a,a',l,g}$, defines the probability that an individual of given gender (g), age group (a) and sexual activity level (l) forms a partnership with someone of opposite gender (g') of a given age (a'). This age mixing matrix is thus level of sexual activity-specific and was derived from observed data as explained in Section 2.2.2.

Global mixing matrix

The global mixing matrix is computed for females only, because the partnership formation and dissolution process is driven by females. The global matrix is computed as the Hadamard (element-wise) product of the mixing matrix by sexual activity level and the mixing matrix by age weighted by the male age-specific partner acquisition rates:

$$\Omega_{al,a'l'} = \Gamma_{l,l',g=1} \cdot \Theta_{a',g=2} \cdot \Lambda_{a,a',l,g=1} \tag{1.4}$$

$\Omega_{al,a'l'}$:	global mixing matrix
$\Gamma_{l,l',g}$:	sexual activity level mixing matrix
$\Theta_{a',g=2}$:	Male partner mean acquisition rates
$\Lambda_{a,a',l,g}$:	age mixing matrix
g:	gender (1=females, 2=males)
a:	age group of individual of gender g
a':	age group of opposite gender partner
l:	sexual activity level of individual of gender g
l':	sexual activity level of opposite gender partner

1.3 Natural History of HPV-related diseases

1.3.1 Cervical cancer

HPV-ADVISE LMIC models the following 18 HPV genotypes individually and independently: 16, 18, 6, 11, 31, 33, 45, 52, 58, 35, 39, 51, 56, 59, 66, 68, 73, and 82. Natural history is assumed to be the same for all countries. That is, we assume that infection with a given genotype does not protect against infection or alter disease progression with the other genotypes (i.e. no partial or mutual exclusion). Our model reproduces progression/clearance through different clinical cytological classifications (e.g., CIN1 to CIN3), and the course of underlying HPV infection progression/clearance to CIN3 based on duration of infection and HPV-type. The infection status (susceptible, infected, and immune) of each individual is type-specific and, therefore, an individual can be infected with multiple genotypes at the same time. This assumption is particularly important as co-infections occur frequently (Koutsky 1992[8], Nobbenhuis 1999[9], Rousseau 2001[10], Thomas 2000[11], Winer 2003[12], Woodman 2001[13]). Infected women can either clear the infection and return to immune/susceptible status or remain infected (Infected 1-4, see Figure A1) and progress in the model to more severe stages of cervical intraepithelial lesions of grade 1 (CIN1), 2 (CIN2) or 3 (CIN3), and invasive cervical cancer

(CC) of stage 1 (localized), stage 2 (regional) or stage 3 (distant). Women with CIN may also regress to a less severe stage or clear the infection and directly return to susceptible/immune status (Figure A1). For transmission probabilities and clearance, progression and regression rates see Section 2.2.3.

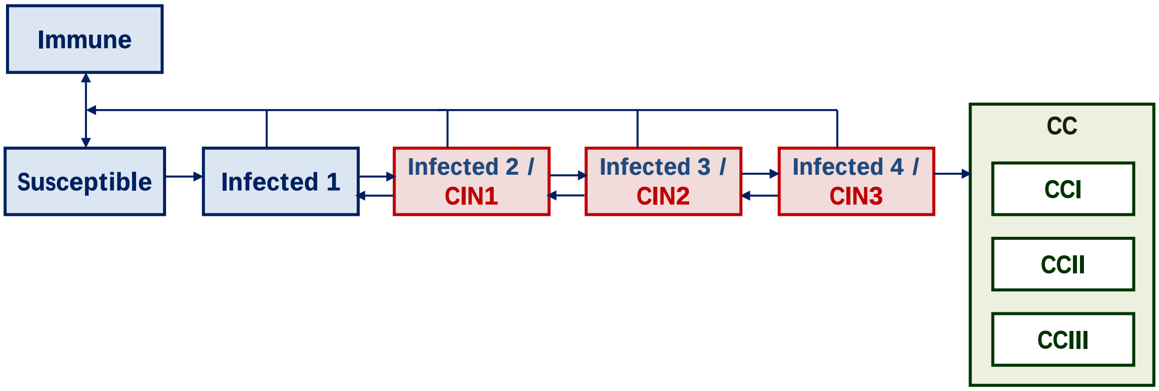

Figure A1. Flow diagram of the natural history of HPV infection and cervical cancer in the absence of screening
The mutually exclusive compartments represent the different HPV epidemiological states. Arrows represent the possible HPV-type, age, and gender specific transitions between these states for each individual.

1.4 Screening

1.4.1 Screening behavior levels

Each country has their own screening behavior. Upon entry in the simulated population, 10-year-old females are assigned a level of screening behavior based on the interval between two routine screening tests. Screening behavior is country specific. The levels of screening behavior range from a short interval between two routine screening tests ($S = 0$) to never being screened ($S = 4$). Please see Section 2.2.4 for the distribution of women assigned to each level of screening behavior.

Different screening methods (ex., Pap or HPV testing) can be attributed to each woman (if screening is available in the population). CC screening initiation is determined by an age-specific rate (which is function of a woman's screening behavior). A screening interval is then attributed to each screening behavior level (see Section 2.2.4 for details).

1.4.2 Screening performance for the detection of cervical lesions

Depending on their true health states (Figure A1) and on screening technology, women are given probabilities of being diagnosed with different results. See Section 2.2.4 for the health state-specific probabilities and references for parameter values.

2 Model Parameterization

A calibration procedure is used to identify multiple parameter sets that simultaneously fit highly-stratified sexual behavior and natural history data. Table A1 in Section 2.1 presents the data sources used for calibration targets and Table A2 in Section 2.2 lists all the model parameters that have been derived through calibration. Section 2.2 describes the prior range calculations and the posterior parameter sets for each parameter.

We identified country-specific prior range of parameters and calibration data through a 4-step process. First, we extracted country-specific data from the Statcompiler of the DHS program to obtain standardized indicators of sexual activity across the 5 LMIC (mainly from the Demographic and Health Survey (DHS) and the AIDS Indicator Survey (AIS), two major population-based surveys). Second, we searched the web for other country-specific population-based surveys about sexual activity. Third, we had access to crude data from several studies conducted by our collaborators and, for the purposes of our mathematical modeling, we obtained a re-analysis of their data using our specific age groups (sexual activity and HPV prevalence). Finally, we performed specific literature searches for sexual activity parameters (mainly regarding female sex work) and HPV prevalence. We searched Medline and EMBASE using a combination of Medical Subject Heading (MeSH) terms, title or abstract word, without restrictions on the language of publication. For example, for HPV prevalence literature search, we used: “Prevalence” or “Prevalence study” AND “Papillomaviridae” or “Papillomavirus infections” or “HPV infection” or “Human papillomavirus infection or “HPV” AND “the specific country”.

2.1 Calibration procedure

The general calibration approach has been described extensively in prior publications (Van de Velde 2007[14], Van de Velde 2010[1], Brisson 2013[3], Brisson 2014[5]): 1) prior distributions are defined for each of the calibrated model parameters (Table A2) (min.–max. values for each parameter are derived from the literature); 2) thousands of different combinations of parameter values are drawn from the prior distributions using Latin Hypercube sampling; 3) parameter sets are qualified as producing a “good fit”, and can be included in the posterior parameter sets, if the associated model predictions fall simultaneously within the pre-specified targets (ranges) of the observed sexual behavior and natural history data described in Table A1; 4) posterior parameter sets are cross-validated by comparing model predictions with observed epidemiological data not used during the fitting procedure. For HPV-ADVISE LMIC predictions, we selected 50 posterior parameter sets (for each country) for model predictions.

Section 2.3 shows examples of model fit to behavior and epidemiological data using the 50 posterior parameter sets for each of the 5 modeled countries. Section 2.4 compares model results obtained

using the 50 posterior parameter sets to observed data not used in the calibration procedure (model validation). Finally, Section 2.5 explains how targets were defined.

Table A1. Description of calibration data

	Stratification	India Ref	India Targets Points	Vietnam Ref	Vietnam Targets Points	Uganda Ref	Uganda Targets Points	Nigeria Ref	Nigeria Targets Points	Benin Ref	Benin Targets Points
**Sexual Behavior**											
Percent that ever had sexual intercourse (women)*	Age (15, 18, 20, 22, 25, [25-49]yrs); Sexual Activity Levels ($l \in \{0, 1, 2, 3\}$)	15,16	32	17	12	18	12	19	26	20-24	32
**Natural history**											
Prevalence of HPV-16/18[¶,#]	Age ([20-24], …, [50-54]yrs)	25,£	14	26,27,£	14	28-30	6	£	14	26,31,32,£	14
Prevalence of HR-HPV[¶,#]	Age ([20-24], …, [50-54]yrs)	33,34,£	14	26,35-38,£	14	29,39-43	12	44-48,£	14	26,32,45,46,49-51,£	14
HPV types distribution in CC[#]	HPV-16, 18, HRC, HRNC	52-58	8	59	8	60-65	8	61,64,66-69	8	50,61,64,66-68,70	8
Incidence of CC	Age ([40-44], …, [60-64], [65+]yrs)	71,72	12	71-73	12	71,72	12	71	12	71	12
**Total number of data points**			80		60		52		74		80

HR=High oncogenic Risk types; HRC=HR Cross-protective types: 31, 33, 45, 52, 58; HRNC=HR Non Cross-protective types: 35, 39, 51, 56, 59, 66, 68, 73, 82; CC=Cervical Cancer.

*. Given uncertainty and scarcity of data, we weighted the point estimates with a factor reflecting differences due to study designs to obtain wider prior ranges (e.g., by comparing GPS Benin[24] vs. DHS Benin 2[23]).

¶. Among sexually active individuals.

#. When country-specific data were scarce, we also used regional data.

£. IARC prevalence data provided by Dr. Iacopo Baussano.

2.2 Parameters

Table A2. List of model parameters

Parameters	Stratification	Data sources & Parameter values
**Demography (Section 2.2.1)**		
Sex ratio at birth	none	
Mortality rates[¶] (per person-year)	Age (a = [10-14], …, [95-99], [100+]yrs);<br>Gender ($g \in \{1, 2\}$)	Section 2.2.1
**Sexual Behavior (section 2.2.2)**		
Proportion of individuals in sexual activity levels	Sexual Activity Levels ($l \in \{0, 1, 2, 3\}$);<br>Gender ($g \in \{1, 2\}$)	Table A4/<br>Figure A2
Onset of sexual activity	Age (10, …, 40yrs);<br>Sexual Activity Levels ($l \in \{0, 1, 2, 3\}$)<br>Gender ($g \in \{1, 2\}$)	Table A5/
Partner acquisition rates (per person-year)	Age (10,… 17, [20-24], …, [45-49], [50-59], [60-69], [70+]yrs);<br>Sexual Activity Levels ($l \in \{0, 1, 2, 3\}$)<br>Gender ($g \in \{1, 2\}$)	Table A6/<br>Figure A4
Separation rates for stable partnerships (per partnership-year)	Age ([10-14], …, [45-49], [50-59], [60-69], [70+]yrs);<br>Sexual Activity Levels ($l = 1$)	Table A8/<br>Figure A6
Proportion of individuals in stable partnerships	Age (10, …, 39, [40+]yrs);<br>Sexual Activity Levels ($l \in \{0, 1, 2, 3\}$)	Table A7
Proportion of partnerships that lead to stable partnerships	Age ([10-14], [15+]yrs);<br>Sexual Activity Levels ($l \in \{0, 1, 2, 3\}$)	Table A7
Contact rates in stable partnerships (per week)	None	Figure A7
Number of contacts per casual partnership	None	Figure A8
Assortative degree for sexual activity matrix	none	Figure A9

	Age matrix (probabilities of one age group to form a partnership with any other age group)	Age ([10-14], …, [65+]yrs);<br>Sexual Activity Levels ($l \in \{0, 1, 2\}$);<br>Gender ($g \in \{1, 2\}$)	Table A9/<br>Figure A10
**Natural history (Section 2.2.3)**			
	Transmission probability for HPV-16 (per act)	Gender ($g \in \{1, 2\}$)	Figure A11
	Relative rate of transmission (vs HPV-16)	HPV-18, HRC, HRNC	Figure A11
	Clearance rate of infection with HPV-16 (per person-year)	Age ([15-65]yrs‡);<br>Gender ($g \in \{1, 2\}$)	Figure A12
	Relative rate of clearance from infection (vs HPV-16)	HPV-18, HRC, HRNC	Figure A13
	Probability of developing lifelong natural immunity	Gender ($g \in \{1, 2\}$)	Figure A14
	Proportion of regressing CIN1 that clears the infection	None	Figure A15
	Progression rates from infection with HPV-16 to CIN1 (per person-year)	None	Figure A16
	Relative rate of progression from infection to CIN1 (vs HPV-16)	HPV-18, HRC, HRNC	Figure A16
	Clearance rate from CIN1 with HPV-16 (per person-year)	None	Figure A17
	Relative rate of clearance from CIN1 (vs HPV-16)	HPV-18, HR	Figure A17
	Progression rates from CIN1 with HPV-16 to CIN2 (per person-year)	None	Figure A18
	Relative rate of progression from CIN1 to CIN2 (vs HPV-16)	HPV-18, HRC, HRNC	Figure A18
	Regression rate from CIN2 with HPV-16 to CIN1 (per person-year)	None	Figure A19

Relative rate of regression from CIN2 to CIN1 (vs HPV-16)	HPV-18, HR	Figure A19
Clearance rates from CIN2 with HPV-16 (per person-year)	None	Figure A20
Relative clearance rate from CIN2 (vs HPV-16)	HPV-18, HR	Figure A20
Progression rates from CIN2 with HPV-16 to CIN3 (per person-year)	None	Figure A21
Relative rate of progression from CIN2 to CIN3 (vs HPV-16)	HPV-18, HRC, HRNC	Figure A21
Regression rate from CIN3 to CIN2 (per person-year)	None	Figure A22
Progression rate from CIN3 with HPV-16 to CC (per person-year)	None	Figure A23
Relative progression rate from CIN3 to CC (per person-year, vs. HPV-16)	HPV-18, HRC, HRNC	Figure A23
Mortality rates from CC (per person-year)	None	Section 2.2.3, page 43
**Screening (Section 2.2.4)**		
Proportion of individuals in screening behavior levels	Screening Behavior Levels ($S \in \{0, 1, 2, 3, 4\}$)	Table A10
Age distribution of first screening test	Age (18, …, 35, [39+]yrs)	Table A10
Screening rates (per person-year)	Age ([10-14], …,[45-49], [50-59], [60-69], [70+]yrs); Screening behavior levels ($S \in \{0, 1, 2, 3, 4\}$); Previous screening results	Table A10
Probability of detecting cervical lesions by cytology	Severity of lesion (Normal, CIN1, CIN2, CIN3, CC)	Table A11
Probability of diagnosing neoplastic states by colposcopy/biopsy	Severity of lesion (Normal, CIN1, CIN2, CIN3, CC)	Table A12

Management of women with abnormal results	None	Table A13
Probability of CIN treatment success	None	Section 2.2.4, page 46
Probability of clearing the infection after CIN treatment success	None	Section 2.2.4, page 46

HR=All high oncogenic Risk types; HRC=HR Cross-protective: 31, 33, 45, 52, 58; HRNC=HR Non Cross-protective: 35, 39, 51, 56, 59, 66, 68, 73, 82; CIN=Cervical Intraepithelial Neoplasia; CC=Cervical Cancer.
¶. Stationary population
ǂ. Linear trend based on values sampled at 15 and 65 years old

2.2.1 Demographic parameters

Each modeled country's gender- and age-specific mortality rates are taken from the Global Health Observatory data repository[74].

2.2.2 Sexual Behavior Parameters

Prior ranges for the sexual behavior parameters are based on the 4-step process of study identification previously described. Table A3 lists all the studies that were used to model sexual behavior (which parameters are informed by the different studies is detailed in subsequent tables).

It has been shown that the most influential sexual behavior parameters in HPV model predictions of vaccination impact are those determining heterogeneity in sexual activity (e.g., including different sexual risk groups and mixing between these groups) (Brisson 2016[75]). Hence, particular effort was placed on properly stratifying the population according to meaningful sexual behavior risk groups (sexual activity levels) and quantifying age-specific mixing.

Proportion of individuals in sexual activity levels. The population is stratified into 4 levels sexual activity. For females, heterogeneity in sexual behavior was modeled according to marital status and sex work, as these data are available and are good markers of sexual activity and risk of HPV infection in low and middle income countries (ex., India: Franceschi 2005[76]; Vietnam: Hernandez 2008[77], Pham 2003[38]; Uganda: Mitchell 2014[40], Safaeian 2008[43]; Nigeria: Clarke 2011[47], Ezechi 2014[44]). The 4 mutually exclusive sexual activity levels are thus L0=women who get married and remain married to a single partner throughout their lifetime, L1=women who get married and then divorce or whose partner has a concurrent partner during their marriage (excluding sexual intercourse with a sex worker), L2=women who never marry, L3=women who are sex workers during their lifetime. For men, L0=men who get married to a single partner and remain married throughout their lifetime, L1=men who get married and who divorce or who have a concurrent partner during their marriage (excluding sexual intercourse with a sex worker), L2=men who never marry, L3=men who pay for sex (these men can be in concurrent partnerships with L0-L2 women). To calculate the distribution of women and men in the level of sexual activity groups, we used the data described in Table A4.

The prior range of proportions of individuals in each sexual activity level was determined by using the minimum and maximum values found in the literature (see Table A4 for data sources). Proportions of individuals sampled within these prior ranges were rescaled to ensure that the 4 proportions sum to 1. Figure A2 shows the posterior parameter sets obtained through model calibration for the proportion of individuals in the sexual activity levels.

Table A3. List of studies used to model sexual behavior

Country	Abbreviation	Study	Region	Population	Sample size	Study type	References
**India**	NFHS-3 India	India national family health survey, 2005-06	National	General population of women aged 15-49 and men aged men age 15-54	198,754 (124,385 women and 74,369 men)	Population-based survey	[15]
	India Census	2001 census of India	National	General population of India	779.1 million (376.2 million females and 402.9 million males)	Census	[78]
	GPS India	Comparative analysis of data from 3 general population surveys to understand heterogeneity in HIV risk	Belgaum (northern), Bellary (mid-state) and Mysore (southern) districts of Karnataka state, south India	General population of women and men aged 15-49	13,026 (6,476 women and 6,550 men)	Population-based surveys	[16]
	Lowndes et al.	Polling Booth Surveys: A novel approach for reducing social desirability bias in HIV-related behavioural surveys in resource-poor settings	Mysore and Belgaum districts, Karnataka state, south India	General population of women and men aged 15-49	14,391 (7,555 women and 6,836 men)	Population-based surveys	[79]
	Gaffey et al.	Male use of female sex work in India: A nationally representative behavioural survey	7 northeastern states of India (Arunachal Pradesh, Manipur, Meghalaya, Mizoram, Nagaland, Sikkim and Tripura)	Men aged 15-49 years	31,040 men	Population-based surveys	[80]
	Mishra et al.	Exploring the population-level impact of antiretroviral treatment: the influence of baseline intervention context	Belgaum, Mysore, and Shimoga districts in Karnataka state, south India	FSW and clients	NA (multiple data sources)	Review, mathematical modeling of HIV transmission	[81]

Country	Abbreviation	Study	Region	Population	Sample size	Study type	References
	Vandepitte et al. 1	Estimates of the number of female sex workers in different regions of the world	Sub-Saharan Africa and Asia	FSW, age 15-49 years	NA (multiple data sources)	Meta-analysis, specific population groups	82
**Vietnam**	Vietnam Census	1989 census of Vietnam	National	Women and men, age ≥13 years	42.1 million individuals (22.3 million females, 19.8 million males)	Census	83
	VPAIS	Vietnam population and AIDS indicator survey, 2005 (DHS)	251 urban and rural clusters in 4 provinces (Ha Noi, Ho Chi Minh City, Hai Phong, Quang Ninh)	Women and men, age 15-49 years	13,996 (7,289 women and 6,707 men)	Population-based survey	17
	PCFPS	The 1/4/2011 Vietnam population change and family planning survey	6 Regions of Vietnam*	Women and men, all ages	399,685 households	Population-based survey	84
	VPHC	The 2009 Vietnam population and housing census	6 Regions of Vietnam*	Women and men, all ages	3.7 million households	Population-based survey	85
	MICS 1	Multiple indicator cluster survey, 2011	6 Regions of Vietnam*	Women, age 15-49 years	11,663 women	Population-based survey	86
	MICS 2	Multiple indicator cluster survey, 2014	6 Regions of Vietnam*	Women, age 15-49 years	9,827 women	Population-based survey	87
	SAVY	Survey and assessment of Vietnamese youth, 2003	6 Regions of Vietnam*	Young women and men, age 14-25 years	7,584 (3,831 women, 3,753 men)	Population-based survey	88
	SAVY2	Survey and assessment of Vietnamese youth round 2, 2010	6 Regions of Vietnam*	Young women and men, age 14-25 years	10,030 (4,924 women, 5,106 men)	Population-based survey	89
	IBBS	HIV/STI integrated biological and behavioral durveillance in Vietnam 2005-2006	7 provinces and cities (Hanoi, Hai Phong, Quang Ninh, Da Nang, Ho Chi Minh City, Can Tho, An Giang)	HIV/STI high risk women and men: injecting drug user (IDU; age 18+), karaoke-based sex workers (KSW; age 18+	2,032 IDU (men), 1,959 KSW (women), 1,588 SSW (women), and 790 MSM	High-risk populations survey	90

Country	Abbreviation	Study	Region	Population	Sample size	Study type	References
				years), street-based sex workers (SSW; age 18+ years) and men who have sex with men (MSM; age 15+ years)			
	VTWG	Vietnam Technical Working Group: Vietnam HIV/AIDS estimates and projections, 2007-2012	6 Regions of Vietnam*	General population of women and men, and HIV/STI high risk women and men: injecting drug user (IDU; age 15+ years), FSW (FSW; age 15+ years), male clients of FSW (age 15+ years) and men who have sex with men (MSM; age 15+ years)	Estimates : 25.1-27.1 million males, 27.1-27.2 million females; 111,233-273,579 IDU, 1.4-2.9 million male clients of FSW, 160,544-481,431 MSM	Population-based and high-risk populations survey	91
	Bui et al.	Cross-sectional study of sexual behavior and knowledge about HIV among urban, rural, and minority residents in Viet Nam	3 districts of Quang Ninh province (Binh Lieu, Yen Hung and Ha Long)	Women and men, age 15-45	630 (311 women and 319 men)	Study among general population	92
	Ghuman et al.	Continuity and change in premarital sex in Vietnam	Northern Vietnam (Red River Delta) and southern Vietnam (Ho Chi Minh City and surroundings)	Women and men, all ages (restricted to women younger than 40 at marriage and men whose wife was younger than 40 at marriage)	2,592 (1,296 women and 1,296 men)	Study among general population	93
	Knodel et al.	Marital sexual behavior and aging in Vietnam in comparative perspective	Northern Vietnam (Red River Delta) and southern Vietnam (Ho Chi Minh City and surroundings)	Women and men, all ages (restricted to women younger than 40 at marriage and men whose wife was younger than 40 at marriage)	2,592 (1,296 women and 1,296 men)	Study among general population	94
	Le et al. 1	Correlates of HIV infection among	10 provinces in northern, central	FSW, age ≥ 18	5,298 FSW (2,530 street-	Specific population	95

Country	Abbreviation	Study	Region	Population	Sample size	Study type	References
		street-based and venue-based sex workers in Vietnam.	and southern regions		based sex workers and 2,768 venue-based sex workers)	groups	
	Vandepitte et al. 1	Estimates of the number of female sex workers in different regions of the world	Sub-Saharan Africa and Asia	FSW, age 15-49 years	NA (multiple data sources)	Meta-analysis, specific population groups	82
	Le et al. 2	Correlates of HIV infection among female sex workers in Vietnam: Injection drug use remains a key risk factor	10 provinces in northern, central and southern regions	FSW, age ≥18 years	5,298	Specific population groups	96
	Tran et al. 1	condom use and its correlates among female sex workers in Hanoi, Vietnam	Hanoi	FSW, age 16-56 years	400	Specific population groups	97
**Uganda**	DHS Uganda	Uganda demographic and health survey 2016	15 regions and 3 special areas of Uganda	General population of women aged 15-49 and men aged 15-54	23,842 individuals (18,506 women and 5,336 men)	Population-based survey	18
	Uganda Census	2002 census of Uganda	National	General population of Uganda	15.9 million individuals (8.2 million women and 7.7 million men) ≥10+yrs	Census	98
	Todd et al.	Reported number of sexual partners: comparison of data from four African longitudinal studies	Zimbabwe, Uganda and South Africa	Women and men 15+ years	NA (multiple study populations)	Population-based surveys	99
	HIV/AIDS KMCC	Sex workers and HIV/AIDS in Uganda: Synthesis of information and evidence to inform the response	National	FSW	NA (multiple study populations)	Comprehensive literature review	100

Country	Abbreviation	Study	Region	Population	Sample size	Study type	References
	HIV/AIDS Uganda	The HIV and AIDS Uganda country progress report 2014	66 districts Uganda	NA (Multiple data sources: Government of Uganda documents bilateral and UN organisations' documents and also from studies)		Report	101
	Muldoon et al.	A systematic review of the clinical and social epidemiological research among sex workers in Uganda.	Kampala, Gulu, northern Uganda, Fishing village in southwestern Uganda, Trading town in southwestern Uganda	FSW	NA (multiple study populations)	Systematic review	102
	Pickering et al.	Sexual networks in Uganda: casual and commercial sex in a trading town	Trading town on the trans-Africa highway	FSW and males that were potential clients of FSW	86 (48 FSW and 38 men)	High-risk populations	103
	Nagaddya et al.	Understanding the dynamics and practices of female sex workers with both circumcised and non-circumcised men in Makindye Division	Makindye Division- Kampala	Women and men 15+ years	314 FSW	High-risk populations	104
	Vandepitte et al. 2	HIV and other sexually transmitted infections in a cohort of women involved in high-risk sexual behavior in Kampala, Uganda	Kampala, Uganda.	FSW, age ≥18 years (15-17 years also eligible if catering for their own livelihood, being pregnant, or already having children)	1,027 FSW	High-risk populations	105
	Kelly et al.	Age differences in sexual partners and risk of HIV-1 infection in rural Uganda	56 communities of rural Rakai District,Uganda	Women 15-29 years	6,177 women	Randomized community-based trial	106
**Nigeria**	DHS Nigeria	Nigeria demographic and health survey 2013	National	General population of women aged 15-49 and men aged 15-49	56,307 individuals (38,948 women and 17,359 men)	Population-based survey	19

Country	Abbreviation	Study	Region	Population	Sample size	Study type	References
	Nigeria Census	1991 census of Nigeria	National	Women and men, age ≥10 years	60.1 million (29.8 million women, 30.3 million men)	Census	[107]
	Ibisomi et al.	Is age difference between partners associated with contraceptive use among married couples in Nigeria?	National	General population of women aged 15-49 and men aged 15-59	13,104 (6,552 sexually active women and their partners)	Population-based survey	[108]
	Vandepitte et al. 1	Estimates of the number of female sex workers in different regions of the world	Sub-Saharan Africa, Asia	FSW, age 15-49 years	NA (multiple data sources)	Meta-analysis, specific population groups	[82]
	Ikpeazu et al.	An appraisal of female sex work in Nigeria - Implications for designing and scaling up HIV prevention programmes	Anambra, Benue, Cross River, Federal Capital Territory (FCT), Lagos, Nasarawa, and Ondo	FSW and secondary key informants	17,266 secondary key informants and 5,732 FSWs	High-risk populations	[109]
	Eluwa et al.	Sexual risk behaviors and HIV among female sex workers in Nigeria	6 Nigerian states Anambra (south east), Cross River and Edo (south south), Federal Capital Territory (north central), Kano (north west), and Lagos (south west)	FSW	5,860 FSW	High-risk populations	[110]
	Stephenson et al.	Community environments shaping transactional sex among sexually active men in Malawi, Nigeria, and Tanzania	Malawi, Nigeria, and Tanzania	Male clients of FSW, age 15-59 years (Nigeria)	1,799 males clients of FSW (Nigeria)	High-risk populations	[111]

Country	Abbreviation	Study	Region	Population	Sample size	Study type	References
	Wellings et al.	Sexual behaviour in context: a global perspective	59 countries	General population of women and men	NA (multiple data sources)	Systematic review of population-based surveys	[112]
**Benin**	DHS Benin 1	Benin demographic and health survey 2001	National	Women aged 15-49 and men aged 20-64	8,928 individuals (6,219 women and 2,709 men)	Population-based survey	[21]
	DHS Benin 2	Benin demographic and health survey 2011	National	Women aged 15-49 and men aged 15-64	21,779 individuals (16,599 women and 5,180 men)	Population-based survey	[23]
	GPS Benin	Assessment of HIV-related risky behaviour: a comparative study of face-to-face interviews and polling booth surveys in the general population of Cotonou, Benin.	Cotonou, Benin	Women aged 15-49 years and men aged 15-64 years	3,675 (1,889 women and 1,786 men)	Population-based survey	[24]
	Vandepitte et al. 1	Estimates of the number of female sex workers in different regions of the world	Sub-Saharan Africa, Asia	FSW, age 15-49 years	NA (multiple data sources)	Meta-analysis, specific population groups	[82]
	Ahoyo et al.	Female sex workers in Benin, 2002. Behavioural survey and HIV and other STI screening	Benin (Cotonou, Porto-Novo, Abomey-Bohicon, Parakou)	Self-identified FSW, age ≥15 years	723 FSW (474 in Cotonou, 128 in Porto-Novo, 42 in Abomey-Bohicon, and 79 in Parakou)	High-risk populations	[113]

IDU=Injection Drugs User; FSW=Female Sex Worker; KSW=Karaoke-based Sex Worker; SSW=Street-based Sex Worker; MSM=Men who have Sex with Men; LDTD=Long Distance Truck Driver

*. 6 regions of Vietnam: Red River Delta, Northern Midlands and Mountain areas, North Central area and Central Coastal area, Central Highlands, South East, Mekong River Delta

Table A4. Data sources for sexual activity levels

Parameters	India	Vietnam	Uganda	Nigeria	Benin
**% individuals by marital statuses** (ever married, never married, divorced/separated)	India Census[78]	Vietnam Census[83]<br>VPAIS[17]<br>PCFPS[84]<br>VPHC[85]	DHS Uganda[18]<br>Uganda Census[98]	DHS Nigeria[19]<br>Nigeria Census[107]	DHS Benin 1[21]<br>DHS Benin 2[23]<br>GPS Benin[24]
**% men report a concurrent partnership**[¥] (lifetime)	GPS India[16]<br>Lowndes et al.[79]<br>Gaffey et al.[80]	VPAIS[17]<br>SAVY[88,*]	DHS Uganda[18]	DHS Nigeria[19]	DHS Benin 2[23]<br>GPS Benin[24]
**% Female Sex Workers** (lifetime)	Mishra et al.[81]<br>GPS India[16]<br>Vandepitte et al. 1[82]	VPAIS[17,&]<br>Vandepitte et al. 1[82]<br>VTWG[91]<br>Bui et al.[92]	HIV/AIDS KMCC[100]<br>HIV/AIDS Uganda[101]	Vandepitte et al. 1[82]<br>Ikpeazu et al.[109]	Vandepitte et al. 1[82]
**% Males Ever Paid for sex** (lifetime)	GPS India[16]<br>Lowndes et al.[79]	VPAIS[17]<br>Bui et al.[92]<br>SAVY[88]<br>Duong 2008[114]	DHS Uganda[18]	DHS Nigeria[19]<br>Stephenson et al.[111]	DHS Benin 2[23]<br>GPS Benin[24]

¥. Not including sex with a female sex worker

*. Given uncertainty and scarcity of data, we weighted the point estimates with a factor reflecting differences due to study designs (we used GPS India[16] and Lowndes et al.[79] for minimum and maximum estimates, respectively) to obtain wider prior ranges.

&. Vietnam and India percentage of female sex workers from GPS India 2011[16] and VPAIS[17] are both estimated to 0.2%, using similar methodologies. Using a different methodology, Mishra et al.[81] estimated a population sex worker prevalence of 1.2% in India. We thus used 1.2% as the maximum value in our prior for Vietnam to obtain wider prior ranges taking into account data uncertainty.

Figure A2. Sexual activity level distribution - Posterior distributions

Sexual activity level distribution in females and males for India, Vietnam, Uganda, Nigeria, and Benin. Box plots represent the medians, and 10th, 25th, 75th, and 90th percentiles of the posterior parameter sets.

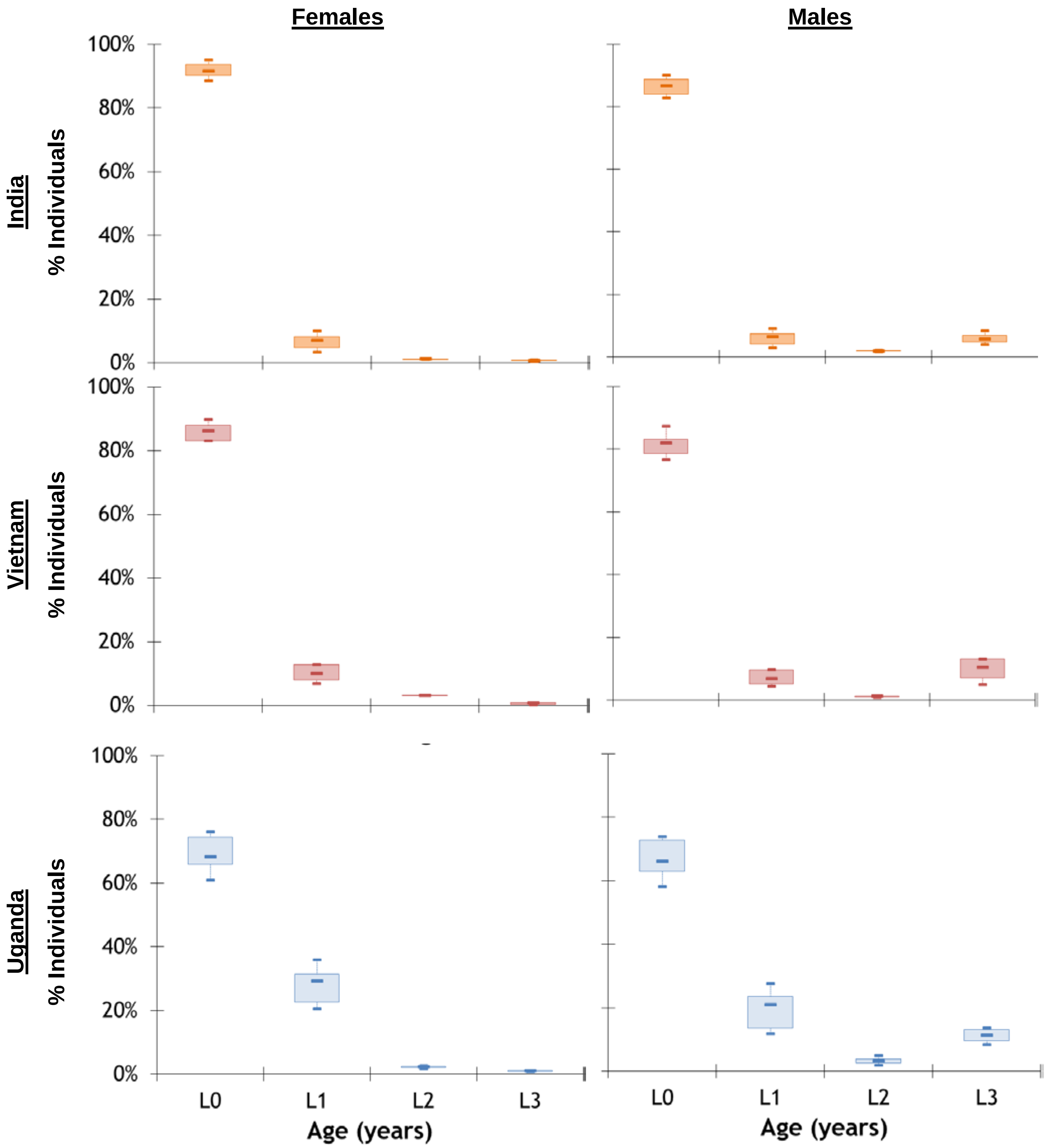

(Figure continued on next page…)

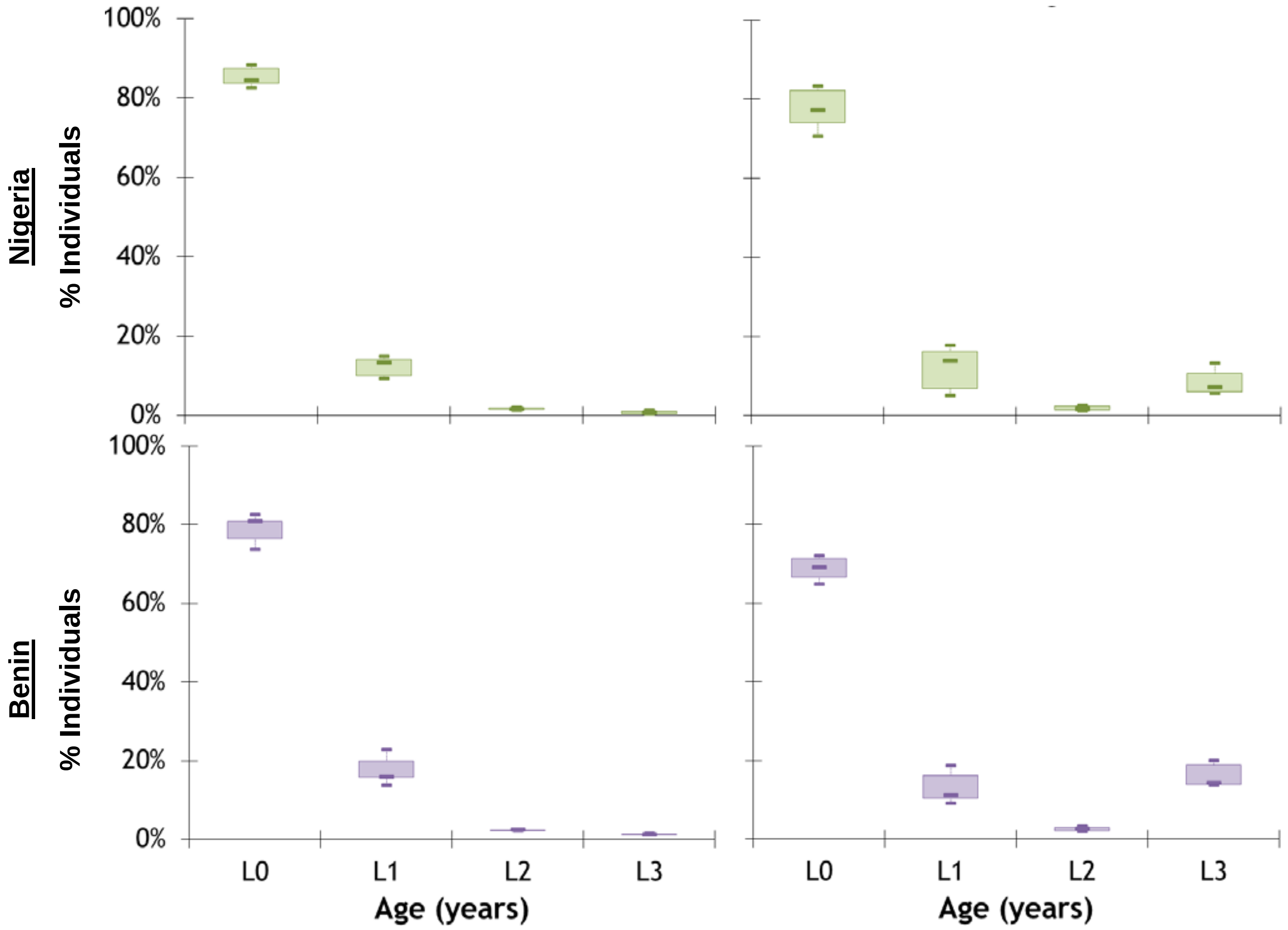

Onset of sexual activity. The prior ranges for the rates of onset of sexual activity in females are based on data on the percentage of girls who ever had sex stratified by age and level of sexual activity (see Table A5 for data sources). Figure A3 shows the percentage of females who ever had sex by level of sexual activity calculated from the posterior rates of onset of sexual activity for each modeled country. Onset of sexual activity in males occurs through female partners choice.

Table A5. Data sources for onset of sexual activity (% ever sex females)

Level of sexual activity	India	Vietnam	Uganda	Nigeria*	Benin
**L0-1** (ever married / consensual unions, divorced / separated)	NFHS-3 India[15] GPS India[16]	VPAIS[17]	DHS Uganda[18]	DHS Nigeria[19]	DHS Benin 2[23] GPS Benin[24]
**L2** (Never married)	NFHS-3 India[15] GPS India[16]	VPAIS[17,*]	DHS Uganda[18]	DHS Nigeria[19]	DHS Benin 2[23] GPS Benin[24]
**L3** (Sex workers)	GPS India[16]	VPAIS[17]	DHS Uganda[18]	DHS Nigeria[19]	DHS Benin 2[23] GPS Benin[24]

*. Given uncertainty and scarcity of data, we weighted the point estimates with a factor reflecting differences due to study designs (e.g., using Lowndes et al.[79], or comparing GPS Benin[24] vs DHS Benin 2[23]) to obtain wider prior ranges.

Figure A3. Proportion of females who ever had sex by age and sexual activity level - Posterior distributions

Proportion of females who ever had sex for each modeled country by age and sexual activity level among females who will have sex in their life. (There is also a proportion of females who will never have sex in their life (not shown): 1.2%, 3.3%, 0.3%, 0.3% and 0.4% in India, Vietnam, Uganda, Nigeria, and Benin, respectively[15,17-19,23]. These females were categorized in level L2 (females who never marry) in the model.) Box plots represent the median, and 10th, 25th, 75th, and 90th percentiles of the 50 posterior parameter sets.

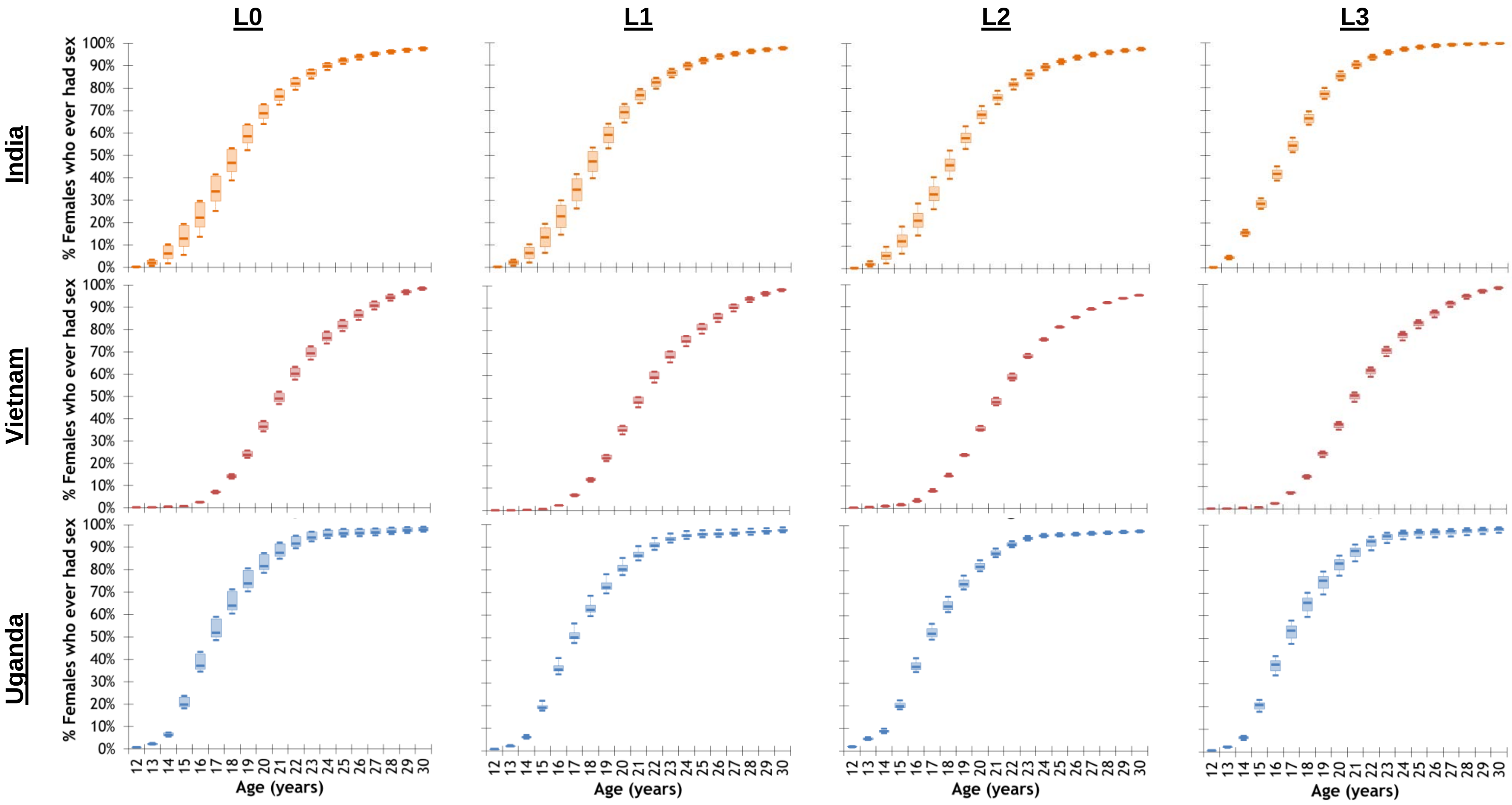

(Figure continued on next page…)

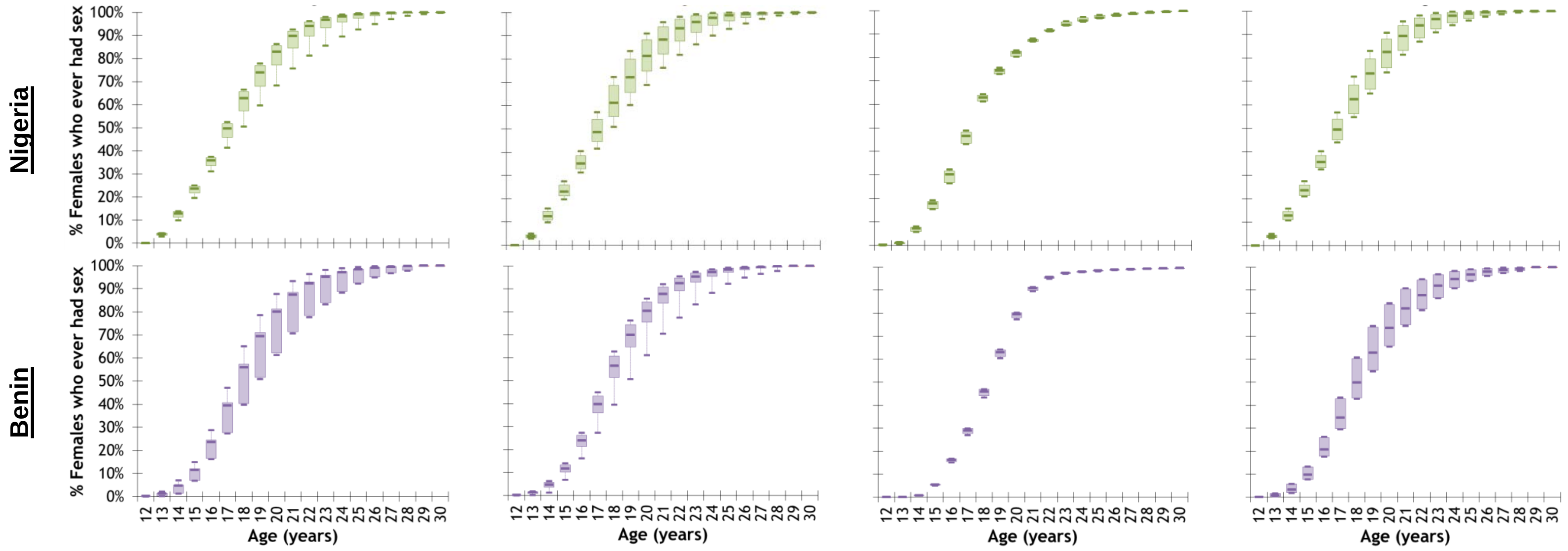

Nigeria
Benin
% Females who ever had sex
100%
90%
80%
70%
60%
50%
40%
30%
20%
10%
0%
12 13 14 15 16 17 18 19 20 21 22 23 24 25 26 27 28 29 30
Age (years)

Partner acquisition rates. The rate of partner acquisition is the rate of new sexual partner acquisition amongst individuals who are sexually active (i.e. number of new partners per year). The data sources from which prior ranges for the partner acquisition rates for women and men by sexual activity level and age were derived are detailed in Table A6. For women and men in the L0 category, the rate of partner acquisition is the rate of new partners among those who have premarital sex or those widowed, as by definition these individuals do not separate. If they have no premarital sex, the rate at which these individuals enter partnership is determined by the rate of onset of sexual activity. Since partnership formation is driven by females, the acquisition rates of males are taken into account through age-dependent weights applied on the female demand (mixing matrix) (see section 1.2.3). Given the uncertainty around some estimates due to small sample size in some age groups, the prior ranges were calculated by multiplying the minimum and maximum values (or point estimates) by 75% and 125%. The model samples different partner acquisition rates for females and males within the prior range for each prior parameter set. Figure A4 shows the mean number of new partners among sexually active females stratified by age and sex level resulting from the 50 posterior parameter sets obtained through calibration for each modeled country. As will be shown in section 2.4 (Model validation), the posterior parameter values produce predictions that reproduce well the observed data for the lifetime number of partners and the number of partners in the past 12 months (see Figure A30 and Figure A31).

Table A6. Data sources for partner acquisition rates

	India	Vietnam	Uganda	Nigeria	Benin
**Females**					
Lifetime number of partners					
All, stratified by marital status			DHS Uganda[18]	DHS Nigeria[19,£]	GPS Benin[24,&,*]
Number of partners in the last year					
Widowed/Separated	GPS India[16]	VPAIS[17]	Todd et al.[99] DHS Uganda[18]		
Never married (sexually active)	GPS India[16] Lowndes et al.[79]	MICS 1[86] VPAIS[17] SAVY[88] SAVY2[89]	Todd et al.[99] DHS Uganda[18]		
Sex worker clients/year	Mishra et al.[81]	Tran et al. 1[97] Le et al. 1[95] IBBS[90]	Muldoon et al.[102] Pickering et al.[103] Nagaddya et al.[104] Vandepitte et al. 2[105]	Eluwa et al.[110] Ikpeazu et al.[109]	Ahoyo et al.[113]

**Males**					
Lifetime number of partners					
All, stratified by marital status				DHS Nigeria[19,£]	GPS Benin[24,*]
Number of partners in the last year					
<u>Widowed/Separated</u>	GPS India[16]<br>Gaffey et al.[80]	VPAIS[17]	Todd et al.[99]<br>DHS Uganda[18]		
<u>Never married</u> (sexually active)	GPS India[16]<br>Lowndes et al.[79]<br>Gaffey et al.[80]	VPAIS[17]<br>SAVY[88]<br>SAVY2[89]	Todd et al.[99]<br>DHS Uganda[18]		

£. Due to lack of data for Nigeria, we used the change rates estimated for Benin on which we applied a scale factor. The scale factor was calculated by comparing lifetime number of partners from Nigeria (DHS Nigeria[19]) and Benin (DHS Benin 2[23]; GPS Benin[24]);
&. Stratified by age
*. We used the number of partners in the past 12 months stratified by age in Uganda and Zimbabwe from Todd et al.[99] to model change rate decrease in older ages for all countries.

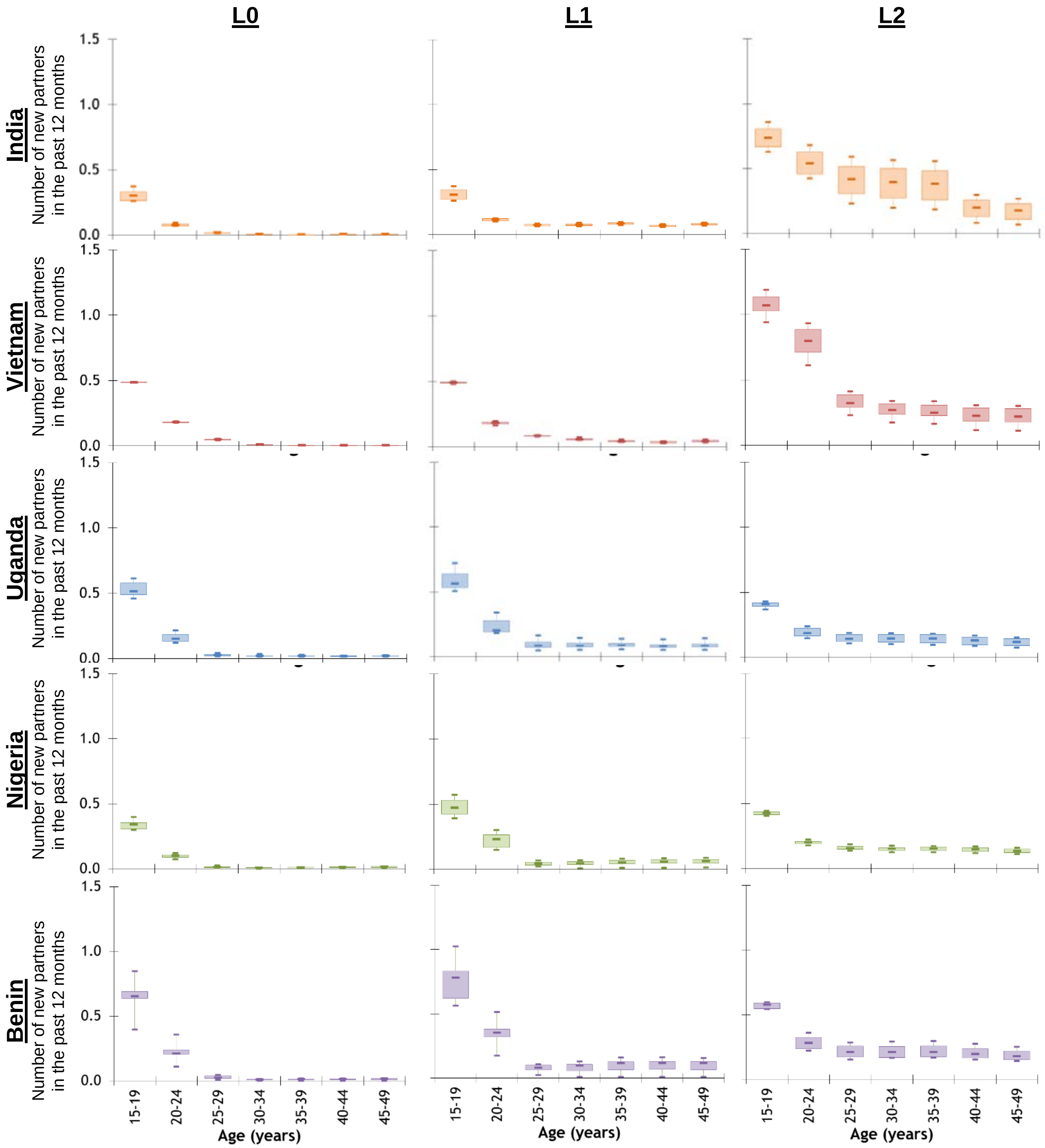

Figure A4. Number of new partners in the past 12 months.
Mean number of new partners among sexually active females in the past 12 months. Box plots represent the median, and 10th, 25th, 75th, and 90th percentiles of the 50 posterior parameter sets.

Proportion of new partnerships that lead to stable partnerships. By definition, L0 women get (and remain) married throughout their lifetime, and L1 women marry and then divorce. We estimate the proportion of new partnerships that lead to casual or stable partnerships among women who eventually marry based on the proportion of women having pre-marital sex. Data sources are shown in Table A7. We assumed that L2 (women who never marry) and L3 (FSW) women only have casual partnerships. Although it is known that FSW can also be married, such relationships would only marginally contribute to the overall HPV transmission dynamics. Figure A5 shows the posterior distribution for the proportion of contacts that lead to stable partnerships.

Table A7. Data sources for proportion of new partnerships that lead to stable partnerships in females and males

	India	Vietnam	Uganda	Nigeria	Benin
**% Premarital sex** (All, spouse, other than spouse)	GPS India[16] Lowndes et al.[79]	Ghuman et al.[93] SAVY[88] SAVY2[89] Bui et al.[92]	DHS Uganda[18]	DHS Nigeria[19]	DHS Benin 2[23] GPS Benin[24]

Stable partnership separation rates. The data sources used to estimate the prior range of the rates of separation amongst stable partnerships are shown in Table A8. L0 women do not separate, and therefore the rate of separation is 0 per partnership-year. For L1, we estimated the average divorce rate among those who will eventually divorce. L2-L3 women are assumed to only have casual partnerships and therefore do not have separation rates. The prior ranges for L1 women were calculated by taking the minimum and maximum of the estimates found in the literature. See Figure A6 for the posterior separation rates for each country.

Table A8. Data sources for separation rates

	India	Vietnam	Uganda	Nigeria	Benin
**% individuals by age and marital statuses** (All, married, consensual union, divorced/separated)	NFHS-3 India[15] India Census 2001[78]	Vietnam Census[83] PCFPS[84] SAVY[88]	DHS Uganda[18] Uganda Census[98]	DHS Nigeria[19] Nigeria Census[107]	DHS Benin 1[21] GPS Benin[24]

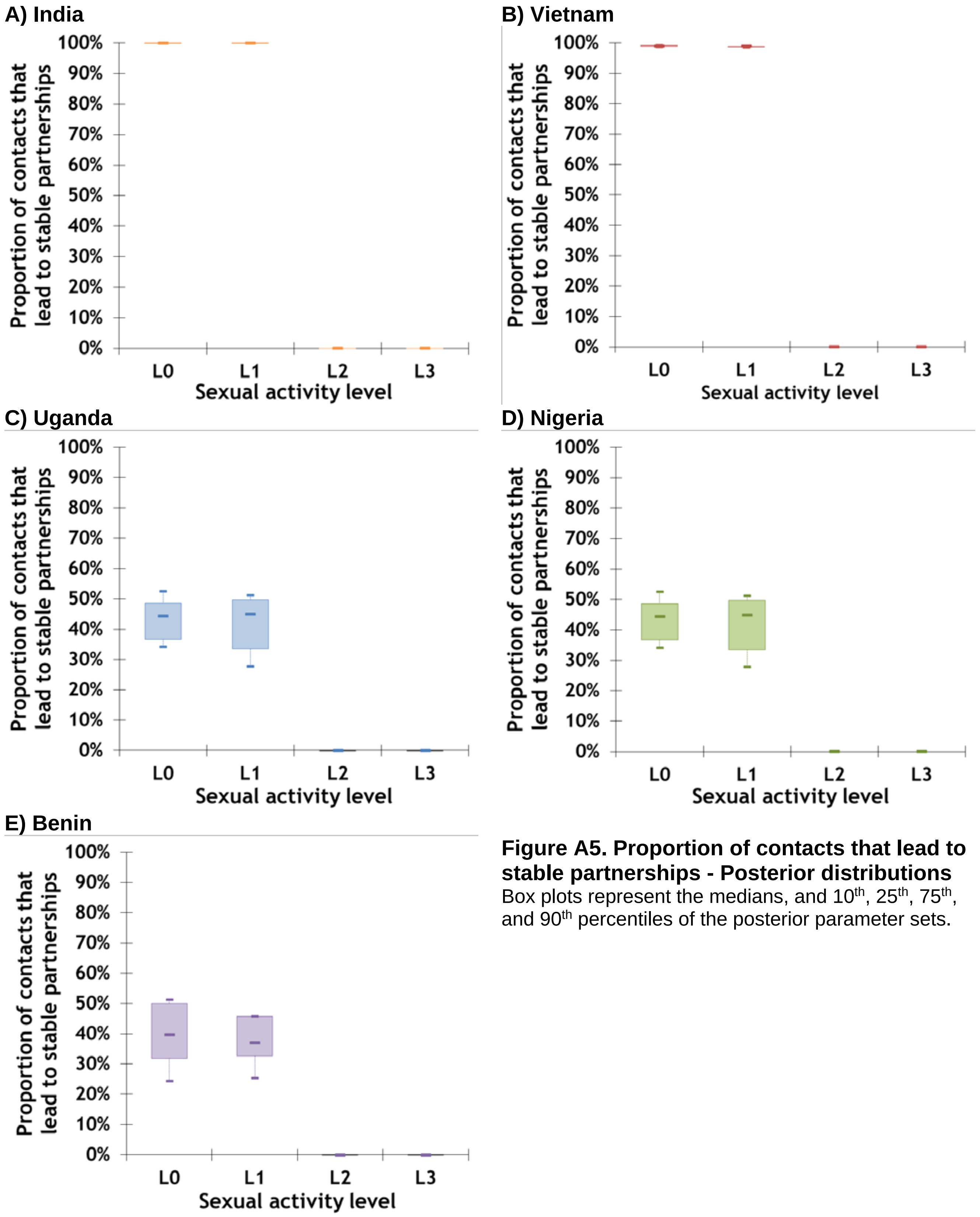

Figure A5. Proportion of contacts that lead to stable partnerships - Posterior distributions
Box plots represent the medians, and 10th, 25th, 75th, and 90th percentiles of the posterior parameter sets.

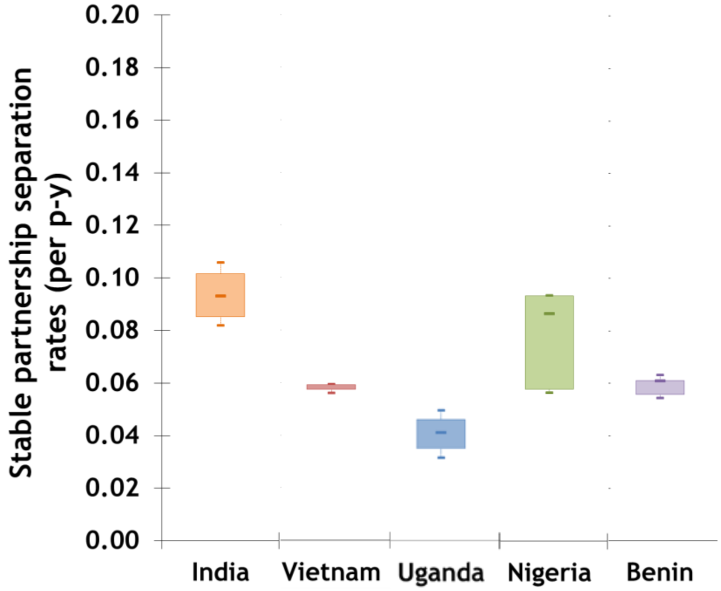

Figure A6. Stable partnership separation rates - Posterior distributions
Box plots represent the medians, and 10th, 25th, 75th, and 90th percentiles of the posterior parameter sets.

Frequency of sex acts in stable partnerships. Due to scarcity of data on the average frequency of sex acts during the course of a stable partnership, we assumed the frequency to be between 1.5 and 4 for all countries except for Vietnam. For Vietnam, we assumed the frequency to be between 1 and 1.75, based on Knodel et al.[94], a marital sexual behavior survey from Vietnam. Figure A7 represents the posterior distribution for the weekly frequency of sex acts in a stable relationship for each country.

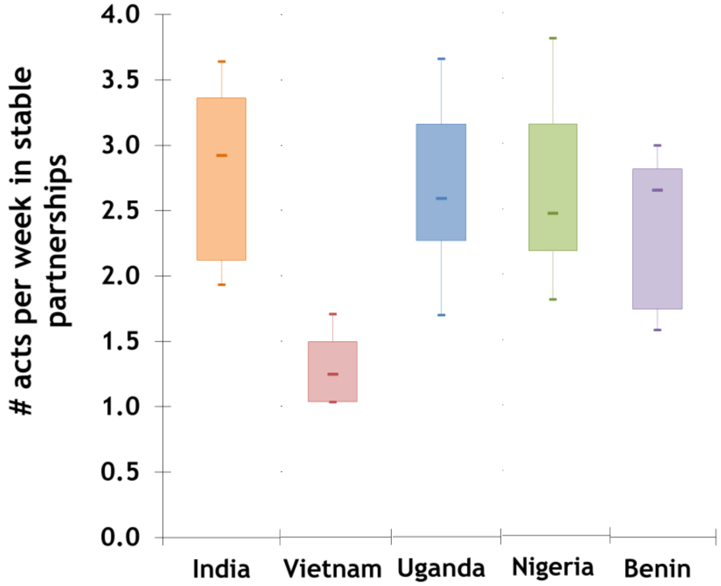

Figure A7. Number of sex acts per week in stable partnerships - Posterior distributions
Box plot represents the medians, and 10th, 25th, 75th, and 90th percentiles of the posterior parameter sets.

Number of sex acts per casual partnership. Casual partnerships are assumed instantaneous (Section 1.2.2). Due to the scarcity of data on the average frequency of sex acts per casual partnership, we assumed the value to be between 1.5 and 4.0. Figure A8 represents the posterior distribution for the number of sex acts per casual partnership.

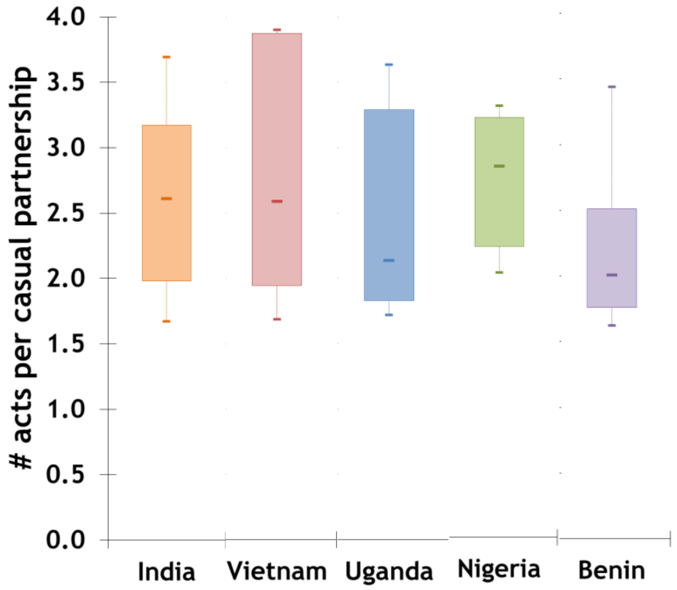

Figure A8. Number of sex acts per casual partnership - Posterior distributions
Box plots represent the medians, and 10th, 25th, 75th, and 90th percentiles of the posterior parameter sets.

Assortative degree of mixing by level of sexual activity. Refer to Section 1.2.3 for the definition of the mixing matrices. In particular, Equations (1.2) and (1.3) define the mixing by level of sexual activity $\Gamma_{l,l',g}$ and the assortative degree κ, respectively. See Figure A9 for the posterior distribution of the assortative degree.

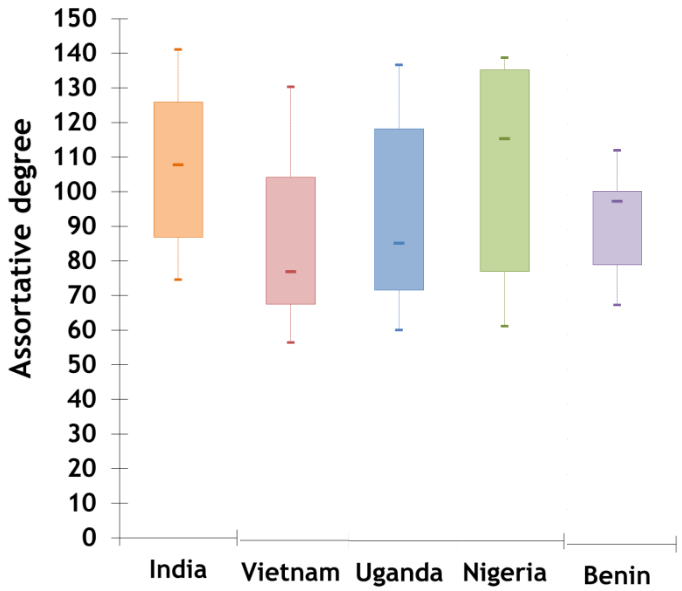

Figure A9. Assortative degree of the mixing between levels of sexual activity - Posterior distributions
Box plots represent the medians, and 10th, 25th, 75th, and 90th percentiles of the posterior parameter sets.

Age mixing matrix. The data sources used to estimate the age mixing matrices for married (L0 and L1) and unmarried (L2) women are detailed in Table A9. The estimated age mixing by sexual activity levels for each modeled country is shown in Figure A10.

Table A9. Data sources for age mixing by age and sexual activity levels

	India	Vietnam	Uganda	Nigeria	Benin
L0-1	GPS India[16]	SAVY[88]<br>MICS 1[86]<br>MICS 2[87]<br>VPAIS[17]	DHS Uganda[18]<br>Kelly et al.[106]	DHS Nigeria[19]<br>Wellings et al.[112]<br>Ibisomi et al.[108]	GPS Benin[24]
L2	GPS India[16]	SAVY[88]<br>MICS 1[86]<br>MICS 2[87]<br>VPAIS[17]	DHS Uganda[18]<br>Kelly et al.[106]	DHS Nigeria[19]	GPS Benin[24]

Figure A10. Estimated age mixing by age and sexual activity level for each modeled country.
(L0-L1=Women that marry; L2=Women that never marry.)

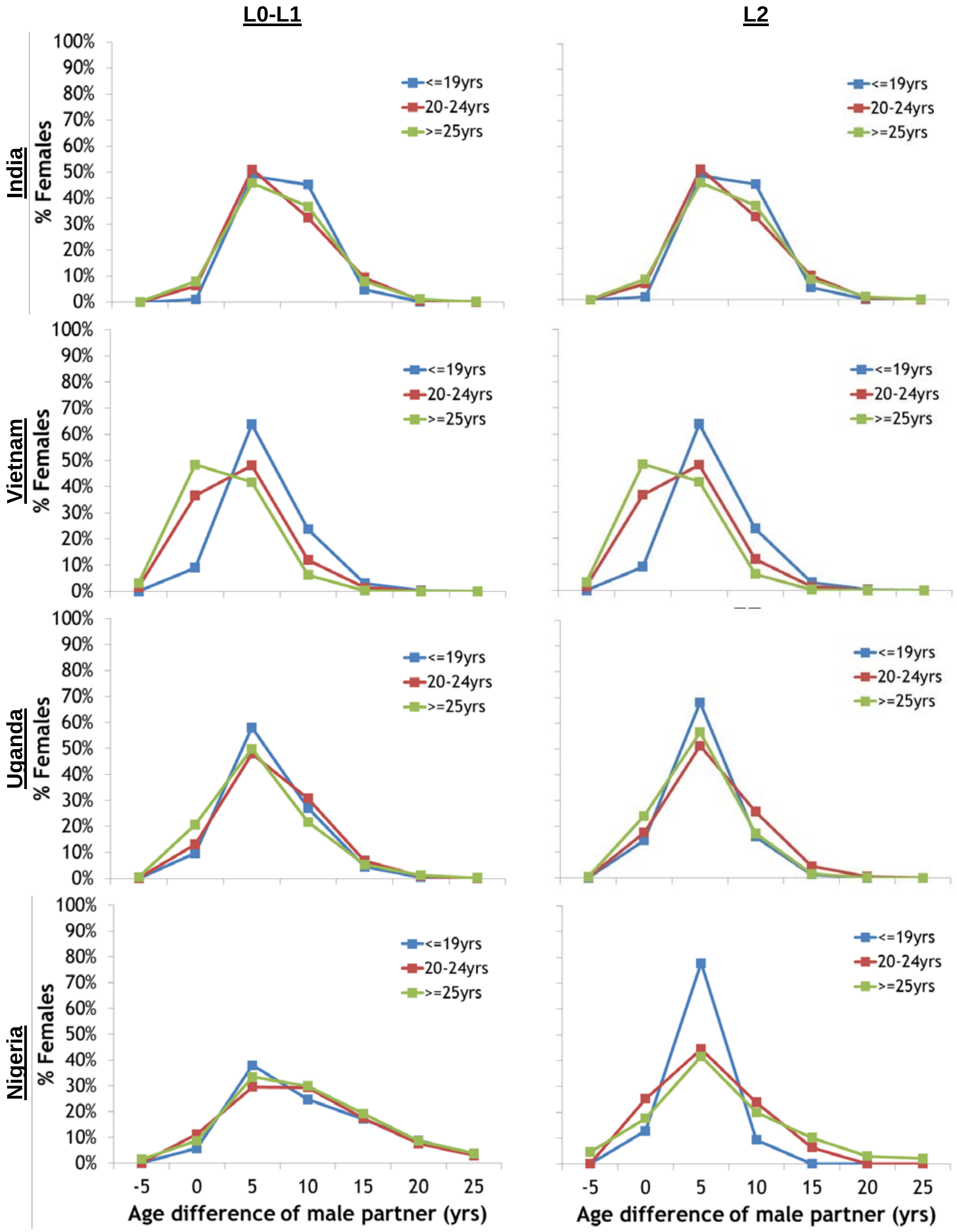

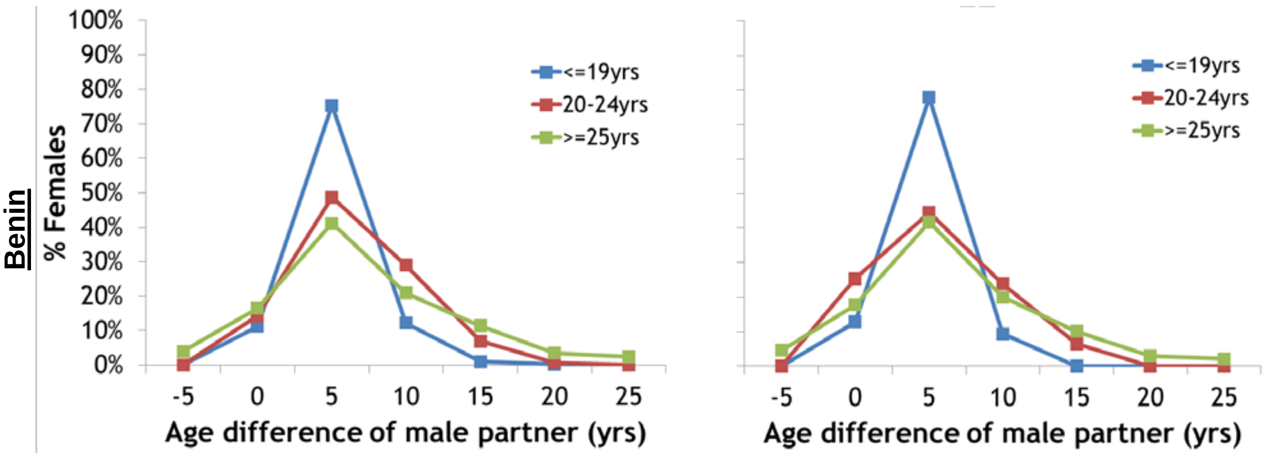

2.2.3 Biological Parameters

See HPV-ADVISE Canada Technical Appendix[4] for a detailed description of the calculation of the biological parameter priors including data sources. All modeled LMICs use the same biological parameter priors.

Per-act transmission probability. In our model, we allocated different per-act transmission probabilities to types HPV-16, 18, cross-protective and non cross-protective high-risk types (Cross: 31, 33, 45, 52, and 58; Not Cross: 35, 39, 51, 56, 59, 66, 68, 73, and 82). Furthermore, we allow male-to-female and female-to-male transmission probabilities to be different. Figure A11 shows the posterior per-act transmission probabilities by HPV-types.

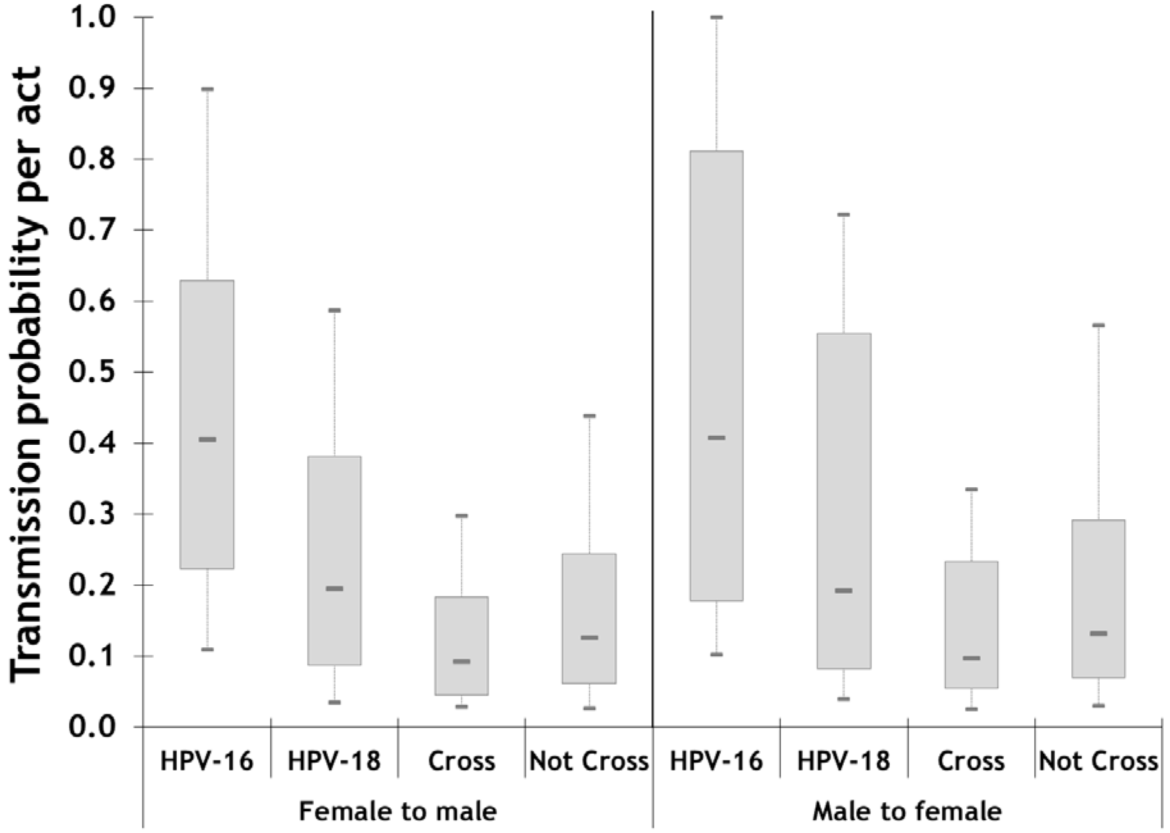

Figure A11. Per-act transmission probabilities - Posterior distributions
Per-act transmission probabilities posterior distributions by HPV types. Box plots represent the medians, and 10th, 25th, 75th, and 90th percentiles of the posterior parameter sets. Cross: high-risk cross-protective types 31, 33, 45, 52, and 58; Not Cross: high-risk non cross-protective types 35, 39, 51, 56, 59, 66, 68, 73, and 82.

Clearance rates. To allow clearance to be age dependent whilst keeping the number of parameters to a minimum, we modeled age-specific clearance rates using a linear trend. For female and male clearance rates, we sample two points from the uniform distribution of HPV-16 clearance. These values are attributed to the first and last age groups, and clearance rates for the intermediate age groups are inferred from the linear trend joining the two values. The HPV-16 clearance rates serve as reference rates. Clearance rates for HPV-18, cross-protective and non cross-protective high-risk types are obtained by multiplying the HPV-16 rates with the sampled relative rates.

Of note, the posterior parameter values for the clearance rates are allowed to be different for females and males.

Of note, even though the high risk types labeled as cross-protective have the same clearance rates, it is important to understand that they are modeled individually and not as a group of types. Figure A12 shows the posterior HPV-16 clearance rates for females and males, and Figure A13 shows the posterior distribution of the relative clearance rates compared to HPV-16.

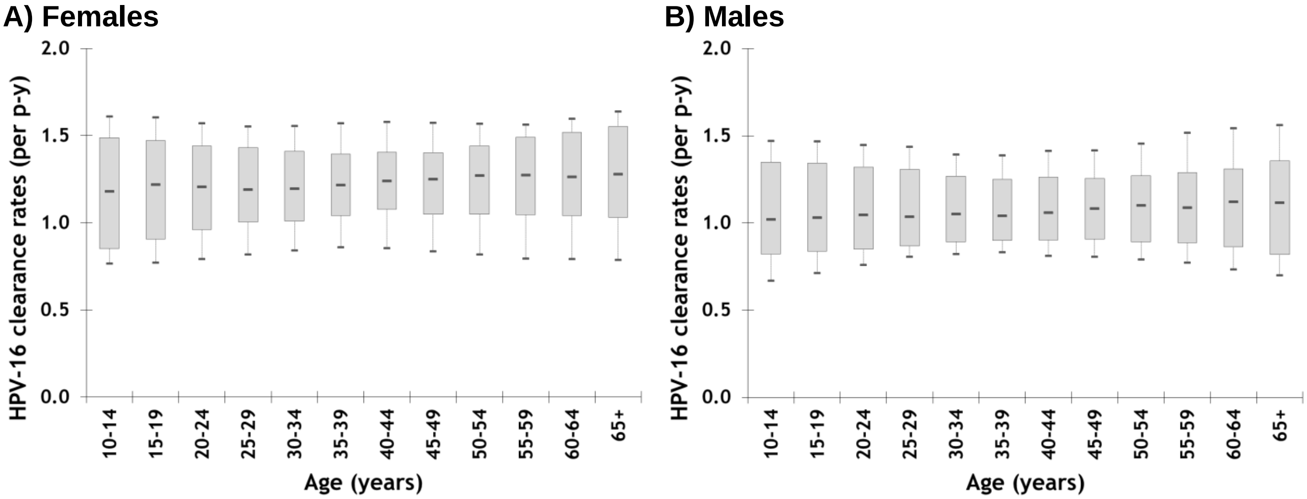

Figure A12. HPV-16 clearance rates - Posterior distributions
HPV-16 clearance rates for A) females and B) males. Box plots represent the medians, and 10th, 25th, 75th, and 90th percentiles of the posterior parameter sets.

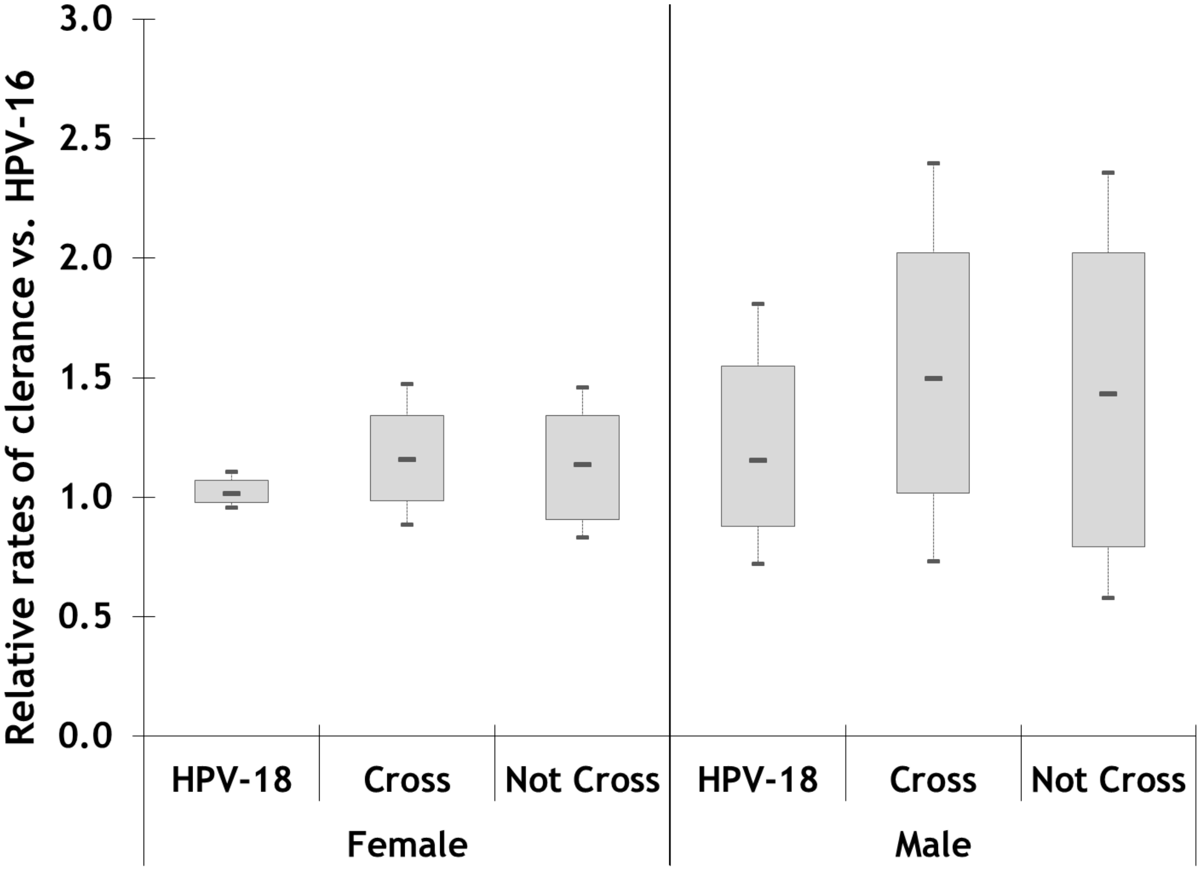

Figure A13. Relative clearance rates compared to HPV-16 - Posterior distributions
Relative clearance rates (vs. HPV-16) posterior distribution. Box plots represent the medians, and 10th, 25th, 75th, and 90th percentiles of the posterior parameter sets. Cross: high-risk cross-protective types 31, 33, 45, 52, and 58; Not Cross: high-risk non cross-protective types 35, 39, 51, 56, 59, 66, 68, 73, and 82.

Probability of developing lifelong natural immunity. Compared to HPV-ADVISE Canada[4], we modified our priors for the probability of developing lifelong natural immunity following infection to take into account a recent meta-analysis by Beachler 2016[115]. We set male probability to null. See Figure A14 for posterior distributions.

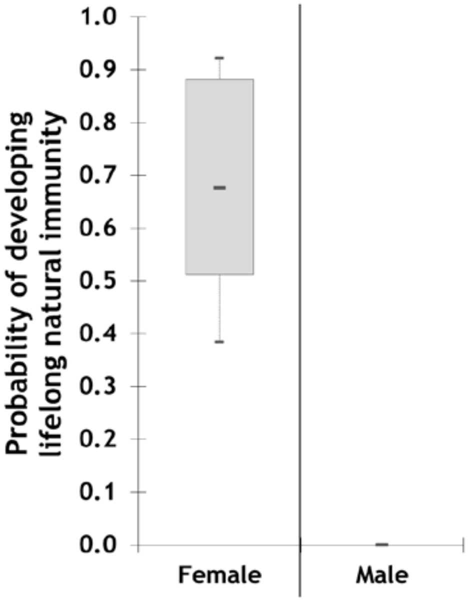

Figure A14. Probabilities of developing lifelong natural immunity following clearance of infection - Posterior distributions
Box plots represent the medians, and 10th, 25th, 75th, and 90th percentiles of the posterior parameter sets.

Progression, regression and clearance rates for cervical intraepithelial lesions. The estimation of the prior ranges for the progression, regression and clearance rates for cervical intraepithelial lesions are detailed in HPV-ADVISE Canada[4]. However, in the HPV-ADVISE version for LMICs, we allow the progression from CIN3 to cancer to be type-specific.

Figure A15 to Figure A23 represent the posterior parameter sets for the natural history parameters.

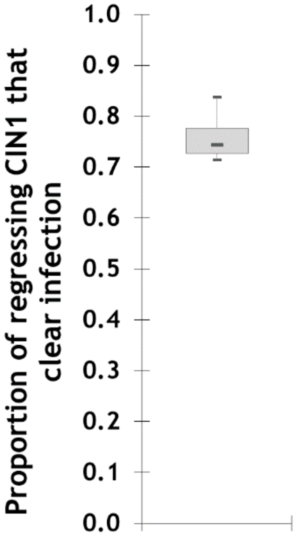

Figure A15. Proportion of regressing CIN1 that clear HPV infection - Posterior distribution
Box plot represents the median, and 10th, 25th, 75th, and 90th percentiles of the posterior parameter sets. Women regressing from CIN1 can either return to the infected or susceptible state.

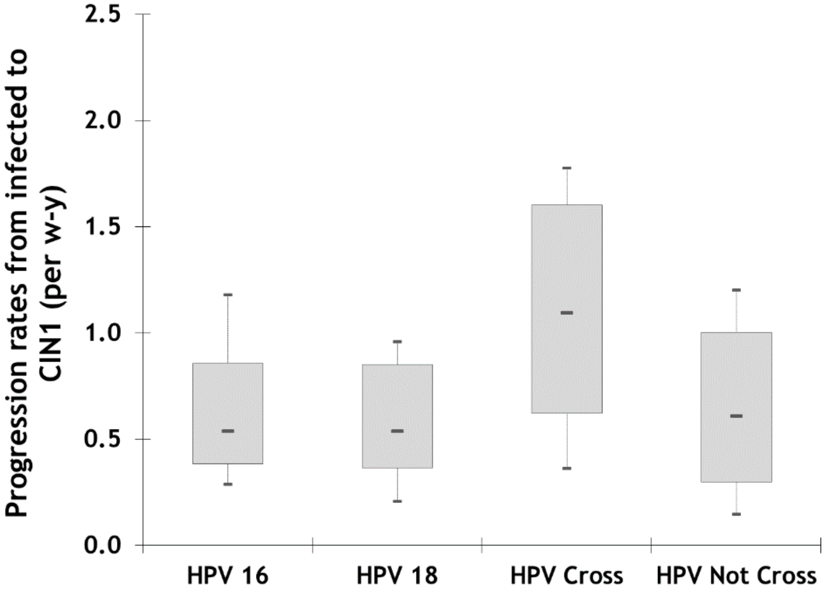

Figure A16. Progression rates from infected to CIN1 - Posterior distribution
Box plots represent the medians, and 10th, 25th, 75th, and 90th percentiles of the posterior parameter sets.

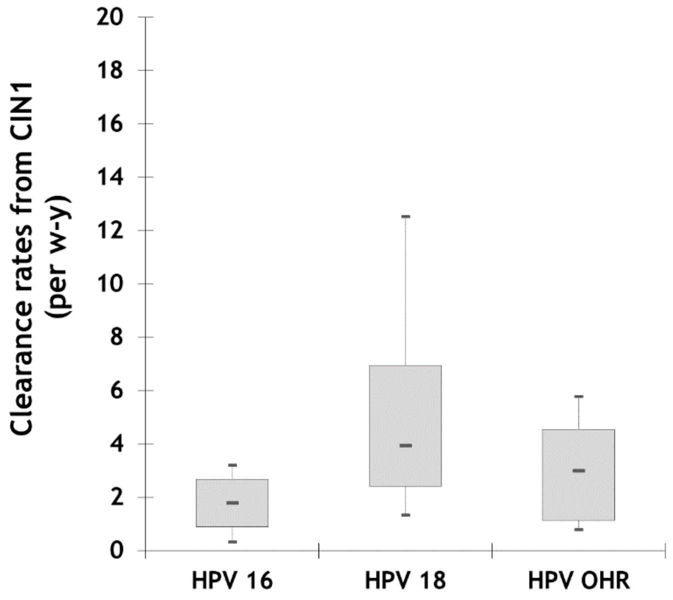

Figure A17. Clearance rates from CIN1 - Posterior distribution
Box plots represent the medians, and 10th, 25th, 75th, and 90th percentiles of the posterior parameter sets.

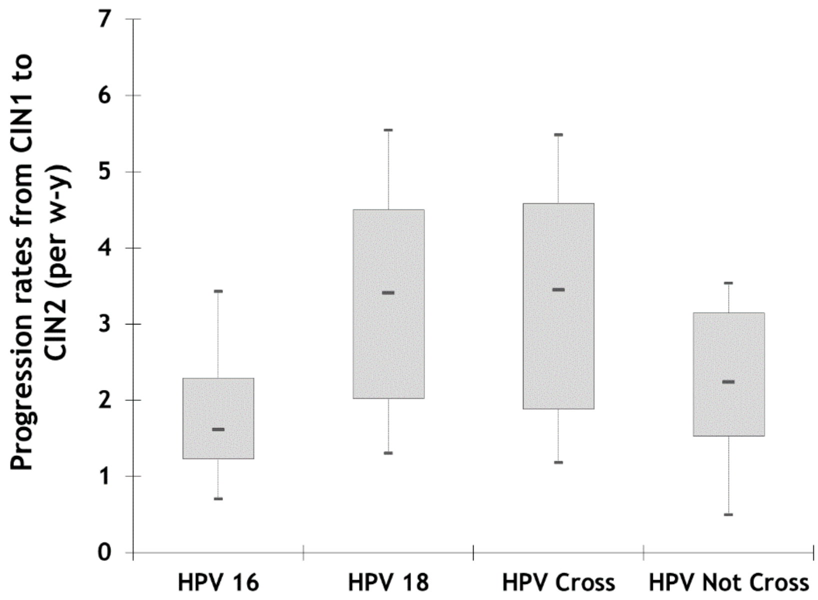

Figure A18. Progression rates from CIN1 to CIN2 - Posterior distribution
Box plots represent the medians, and 10th, 25th, 75th, and 90th percentiles of the posterior parameter sets.

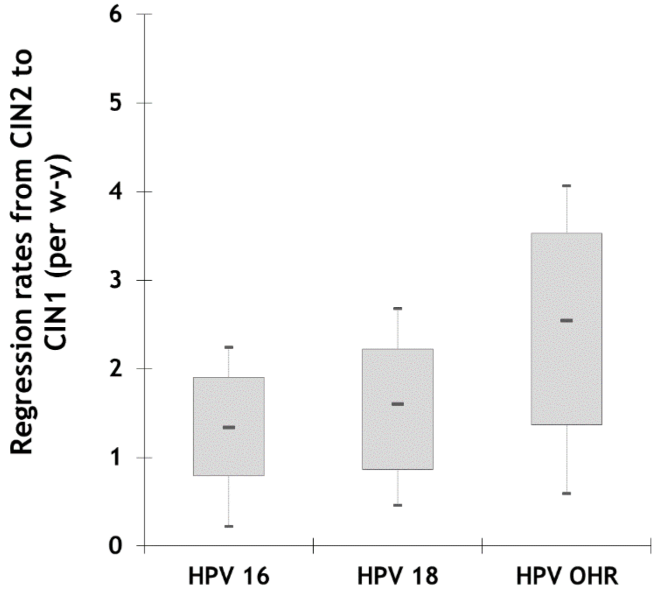

Figure A19. Regression rates from CIN2 to CIN1 - Posterior distribution
Box plots represent the medians, and 10th, 25th, 75th, and 90th percentiles of the posterior parameter sets.

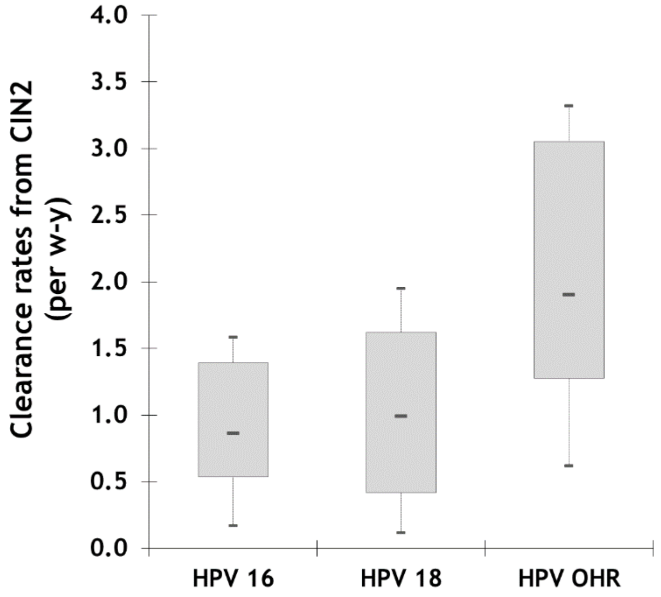

Figure A20. Clearance rates from CIN2 - Posterior distribution
Box plots represent the medians, and 10th, 25th, 75th, and 90th percentiles of the posterior parameter sets.

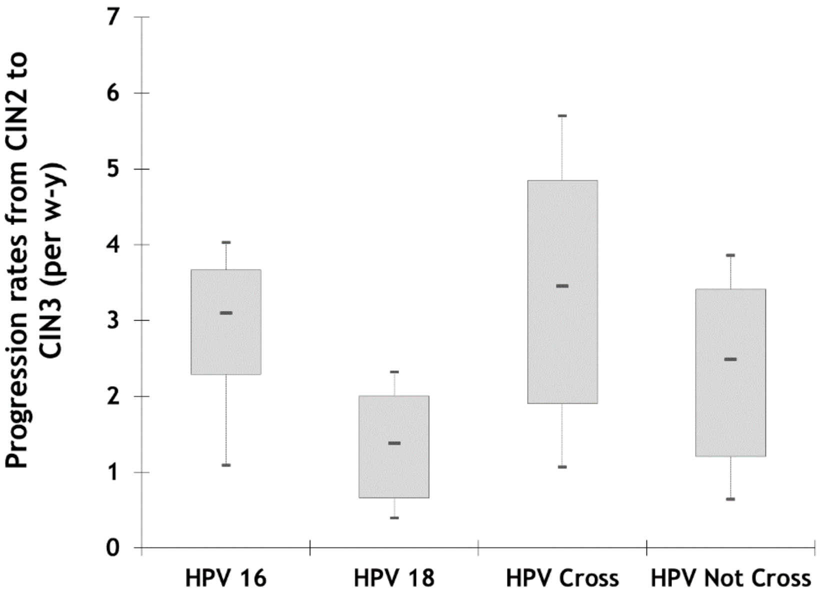

Figure A21. Progression rates from CIN2 to CIN3 - Posterior distribution
Box plots represent the medians, and 10th, 25th, 75th, and 90th percentiles of the posterior parameter sets.

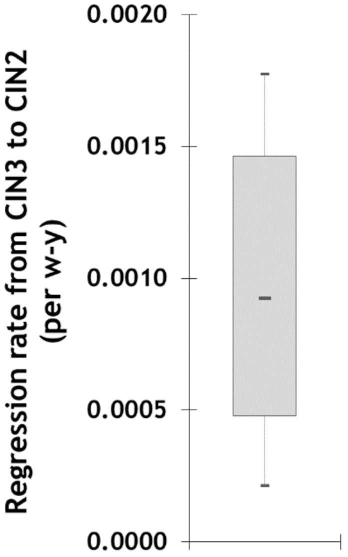

Figure A22. Regression rates from CIN3 to CIN2 - Posterior distribution
Box plots represent the medians, and 10th, 25th, 75th, and 90th percentiles of the posterior parameter sets.

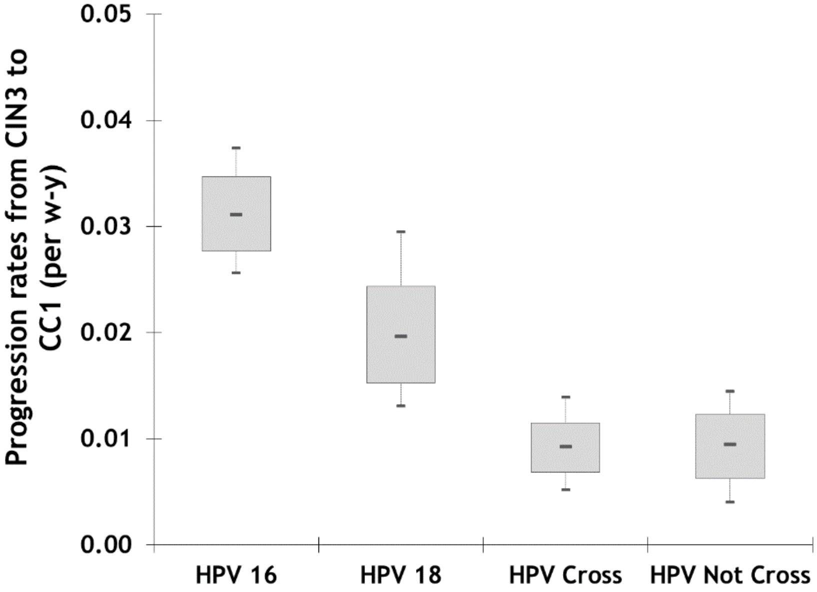

Figure A23. Progression rates from CIN3 to CC1 - Posterior distribution
Box plots represent the medians, and 10th, 25th, 75th, and 90th percentiles of the posterior parameter sets.

Symptoms and mortality in cervical cancer. We used previously published estimates of the probability of developing symptoms from Myers 2000[116]. Country- and age-specific mortality rates for cervical cancer were taken from GLOBOCAN[71].

2.2.4 Screening Parameters

Proportion of women in screening behavior levels and Onset of cervical cancer screening. The proportion of women in screening behavior levels and the age at onset of cervical cancer screening are shown in Table A10.

Table A10. Proportion of women screened for cervical cancer and onset of cervical cancer screening in each modeled country

	India	Vietnam	Uganda	Nigeria	Benin
Proportion of women that are screened for cervical cancer	5%<br><br>Data sources:<br>ICO India[117,£]	9%<br><br>Data sources:<br>ICO Vietnam[59]<br>Gakidou et al.[118]<br>Hoang et al.[119]<br>WHS Vietnam[120]	5%<br><br>Data source:<br>Ndejjo et al.[121]	9%<br><br>Data source:<br>ICO Nigeria[122]	0%<br><br>Data source:<br>ICO Benin[123]<br>Gakidou et al.[118]
Age at onset of cervical cancer screening	25 years<br><br>Data source:<br>ICO India[117]	25 years<br><br>Data source:<br>ICO Vietnam[59]	35 years<br><br>Data source:<br>ICO Uganda[124]	35 years<br><br>Data source:<br>ICO Nigeria[122]	NA

£. Personnal communication, Dr. Partha Basu, IARC.

Screening performance for the detection of infection and cervical lesions. In LMICs, the main test currently used for primary screening is either the Pap test, or visual inspection of the cervix with acetic acid (VIA; Domingo et al.[125], ICO reports[59,117,122,124]). We have previously estimated screening performance for the Canadian and US versions of HPV-ADVISE. The parameters for the probabilities of detecting women in each neoplastic state by cervical cytology (Table A11) were estimated using the data of two systematic reviews on psychometric performance of CC screening with cytology (Nanda et al.[126], and Arbyn et al.[127]). More specifically, in Nanda et al.[126], we used data collected in low HPV prevalence settings and corrected for verification bias whereas in Arbyn et al.[127], we used data presented for conventional cytology. We complemented these data with information from two studies presenting the specific cytological result obtained by women diagnosed with an invasive cancer (Martin-Hirsch et al. 2007[128], and Wright et al.[129]). Given uncertainty around the estimates of sensitivity and specificity, we used the 95% confidence intervals provided in the papers to obtain a range of probabilities. When confidence intervals were unavailable, we varied the point estimate by ±10%. Given the very different context for screening in LMICs, we examined whether sensitivity and specificity estimates for cytology corresponded with those from Nanda et al.[126] and Arbyn et al.[127] and found they were in the same range. For visual inspection, specificity may be slightly lower than cytology, but sensitivity is similar (hence, if a proportion of tests are visual inspection rather than cytology, this would have very little impact on our model predictions). Finally, we used the same

probabilities of diagnosing a neoplastic state by colposcopy/biopsy as in HPV-ADVISE Canada and US (see Table A12).

Table A11. Probabilities of detecting a neoplastic state by cytology

	Cytology results					
**Health States**	**Normal %**	**ASC-US %**	**LSIL %**	**HSIL/ASC-H+ %**	**Cancer %**	**Total %**
**Normal**	98.0	1.5	1.0	0.45	0.05	100.0
	(95.0-99.0)	(1.0-2.0)	(0.5-1.5)	(0.0-1.0)	(0.0-0.5)	
**CIN1**	41.0	12.0	29.0	18.0	0.0	100.0
	(37.0-45.0)	(10.5-14.5)	(26.5-40.5)	(8.0-18.0)	(0.0-0.0)	
**CIN2/3**	20.0	5.0	20.0	53.0	2.0	100.0
	(18.0-22.0)	(3.0-7.0)	(18.0-22.0)	(48.0-54.0)	(1.0-3.0)	
**Cancer**	0.0	6.0	9.0	54.0	31.0	100.0
	(0.0-2.0)	(2.0-9.0)	(3.0-12.0)	(50.0-60.0)	(27.0-35.0)	

ASC-US=Atypical Squamous Cells of Undetermined Significance; LSIL=Low grade Squamous Intraepithelial Lesion; HSIL= High grade Squamous Intraepithelial Lesion ASC-H+=Atypical Squamous Cells - cannot exclude HSIL; CIN=Cervical Intraepithelial Neoplasia

Parameters for the probabilities of confirming the neoplastic state by colposcopy/biopsy are shown in Table A12. They were estimated using the data from several articles assessing the success of colposcopy at diagnosing CIN or the inter-/intra-observer agreement in CIN diagnosis (Gage et al.[130], Chase et al.[131], Mitchell et al.[132], Da Fomo et al.[133], Cai et al.[134]. Given that sensitivity estimates of colposcopy/biopsy to diagnose CIN highly depends on the number and location of biopsies taken (Gage et al.[130]), we considered a wide range of probabilities to account for different biopsy practices.

Table A12. Probabilities of diagnosing a neoplastic state by colposcopy/biopsy

Health States	Colposcopy/biopsy results: Normal %	CIN1 %	CIN2 %	CIN3 %	Cancer %	Total %
**Normal**	88.0 (65-100)	7.0 (0-28)	3.0 (0-5)	2.0 (0-2)	0.0 (0-0)	100.0
**CIN1**	22.0 (10-38)	62.0 (57-90)	15.0 (0-3)	1.0 (0-2)	0.0 (0-0)	100.0
**CIN2**	10.0 (5-19)	10.0 (5-13)	47.0 (52-85)	35.0 (0-16)	0.0 (0-0)	100.0
**CIN3**	10.0 (1-19)	10.0 (3-13)	16.0 (6-16)	56.0 (42-81)	10.0 (0-10)	100.0
**Cancer**	0 (0-0.5)	0.0 (0-2)	0.0 (0-2.5)	5.0 (0-5)	95.0 (90-100)	100.0

CIN=Cervical Intraepithelial Neoplasia

Management of women with abnormal results. Based on Goldie et al.[135], we assumed 15% loss to follow-up after an abnormal result in routine screening. Table A13 shows the parameters for the management of women by screening results. Based on a Cochrane systematic review on the efficacy of seven alternative surgical treatments for CIN (Martin-Hirsch et al. 2009[136]), we assumed that treatment fails for 5% of women (the health state of these women remains unchanged after treatment). Using data from Kreimer et al.[137], we assumed that 80% of women clear both the lesion and the infection after treatment and 15% clear the lesion but remain HPV infected. We assumed the following screening strategy: repeat cytology for low grade lesions, colposcopy and possible biopsy for high grade lesions, and treatment of precancerous lesions or invasive cancer.

Table A13. Parameters for the management of women with a first or repeated abnormal cytology result, according to the severity of the result

	**First abnormal result**				**Repeat abnormal result**			
**Follow-up**	ASC-US	LSIL	HSIL/ ASC-H	Cancer	ASC-US	LSIL	HSIL/ ASC-H	Cancer
Lost to follow-up	15%	15%	15%	15%	0.0%	0.0%	0.0%	0.0%
Repeat cytology	85.0%	85.0%	0.0%	0.0%	0.0%	0.0%	0.0%	0.0%
Colposcopy/biopsy	0%	0%	85%	85%	100%	100%	100%	100%

ASC-US=Atypical Squamous Cells of Undetermined Significance; LSIL=Low grade Squamous Intraepithelial Lesion; HSIL= High grade Squamous Intraepithelial Lesion ASC-H+=Atypical Squamous Cells - cannot exclude HSIL.

2.3 Model fit

Please see Table A1 for details on the calibration data used to fit the model (stratifications, references and number of data points), and Section 2.5 for target definitions. Figure A24 to Figure A28 illustrate the model fit to sexual behavior, HPV prevalence, incidence of CC, and HPV types distribution in CC, respectively.

2.3.1 Fit to sexual behavior data

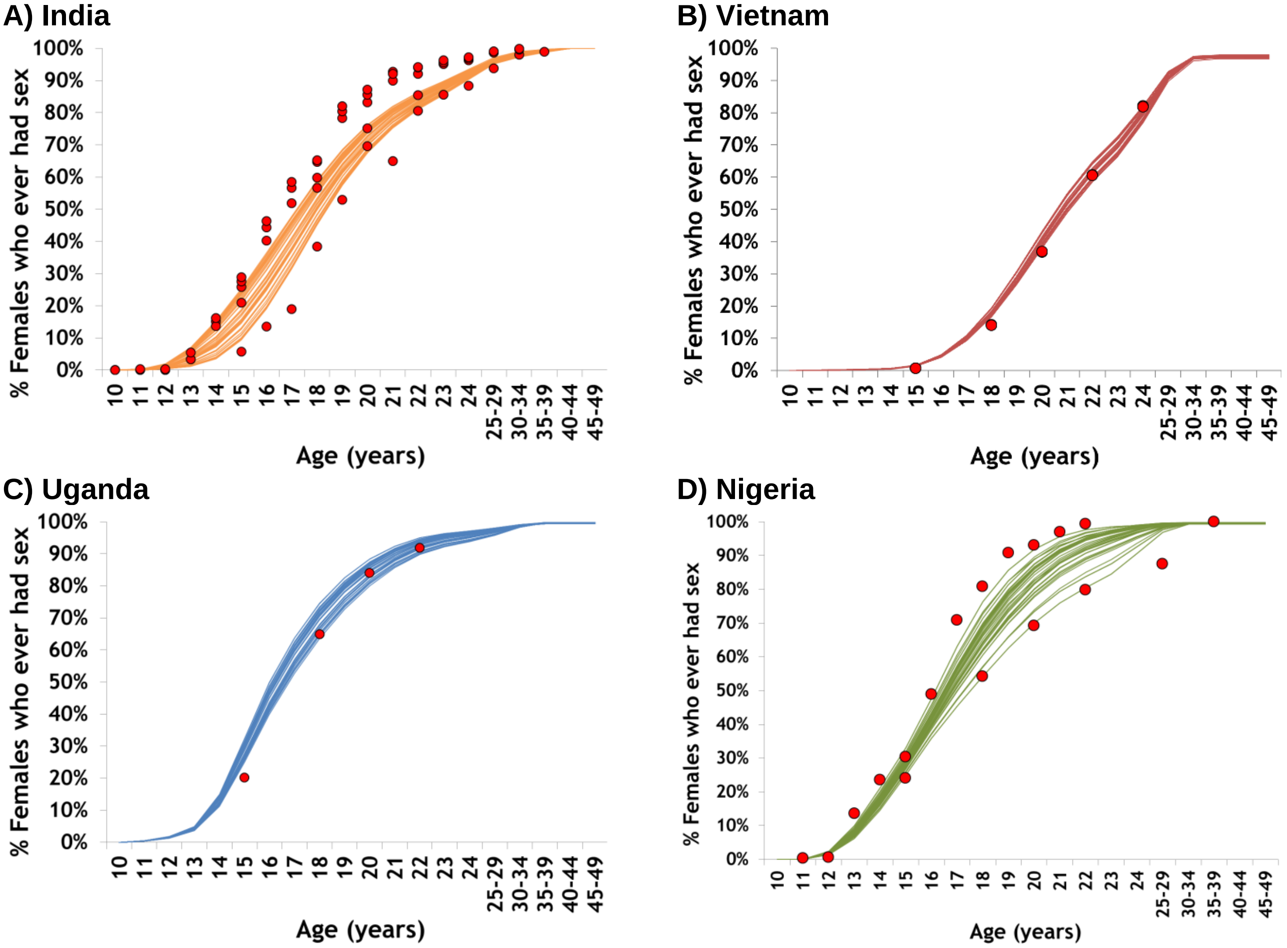

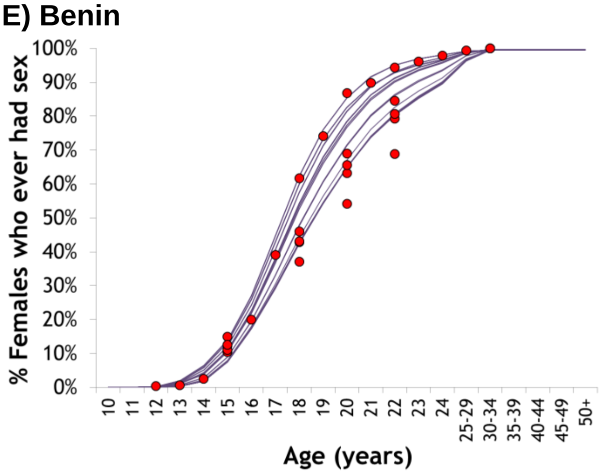

Figure A24. Fit to proportion of sexually active women

Proportion of sexually active women for A) India, B) Vietnam, C) Uganda, D) Nigeria, and E) Benin. Solid lines represent the model predictions generated by the posterior parameter sets. Red dots represent observed data (India: GPS India[16], NFHS-3 India[15] ; Vietnam: VPAIS[17]; Uganda: DHS Uganda[18]; Nigeria: DHS Nigeria[19]; Benin: DHS Benin 1996[20], DHS Benin 1[21], DHS Benin 2006[22], DHS Benin 2[23], GPS Benin[24]).

2.3.2 Fit to HPV prevalence data

Figure A25 and Figure A26 show the fits to prevalence data.

A) India

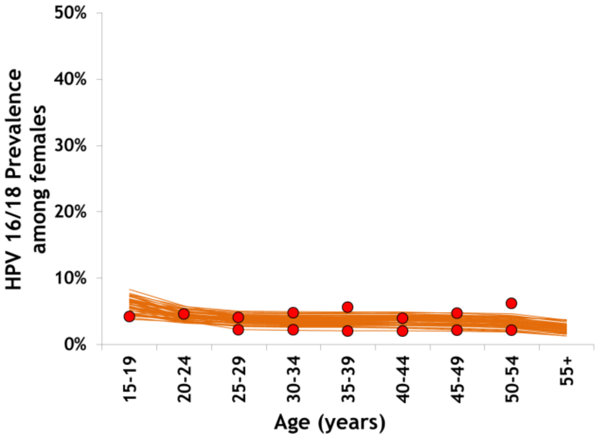

B) Vietnam

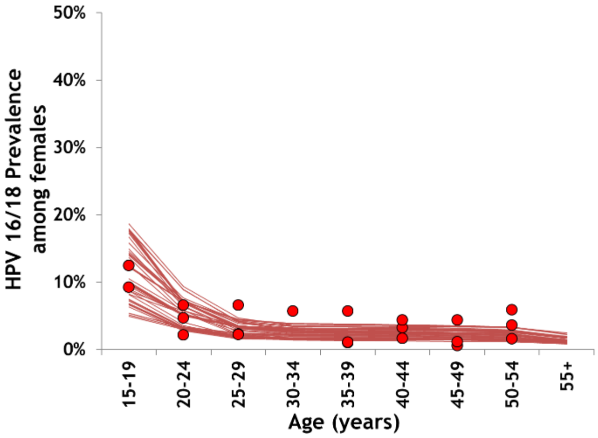

C) Uganda

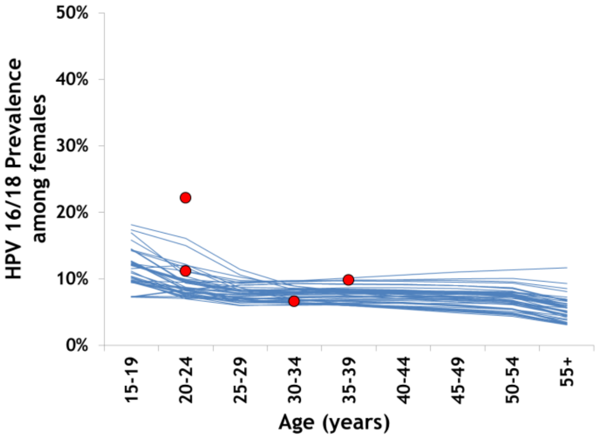

D) Nigeria

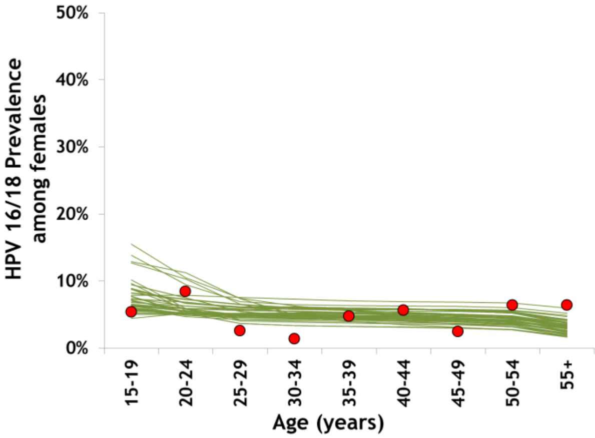

E) Benin

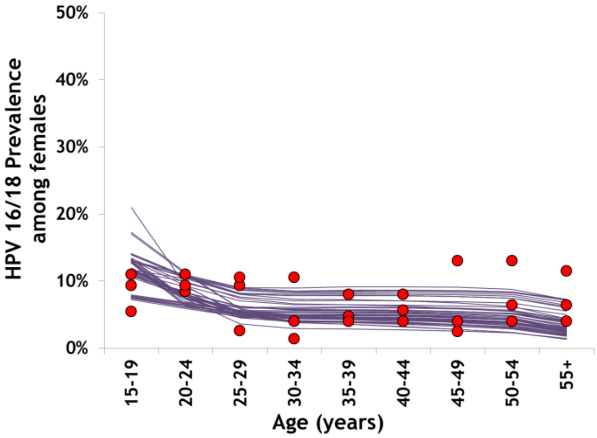

Figure A25. Fit to HPV-16 and 18 prevalence among sexually active females

Fit to prevalence of HPV-16 and 18 infections among sexually active females for A) India, B) Vietnam, C) Uganda, D) Nigeria, and E) Benin. Solid lines represent the model predictions generated by the posterior parameter sets. For model predictions, we assumed a specificity of 99.7% for the HPV-test. Red dots represent the observed prevalence data (data sources for India: Dutta et al.[25], IARC prevalence data provided by Dr. Iacopo Baussano; Vietnam: Vu et al. 2013[27] and IARC prevalence data provided by Dr. Iacopo Baussano; Uganda: Banura et al.[28], Moses et al.[29], Kumakech et al.[30]; Nigeria: IARC prevalence data for Nigeria provided by Dr. Iacopo Baussano; Benin (due to lack of data for Benin, we used regional data): Ouedraogo et al.[31], Keita et al.[32], IARC prevalence data provided by Dr. Iacopo Baussano).

A) India

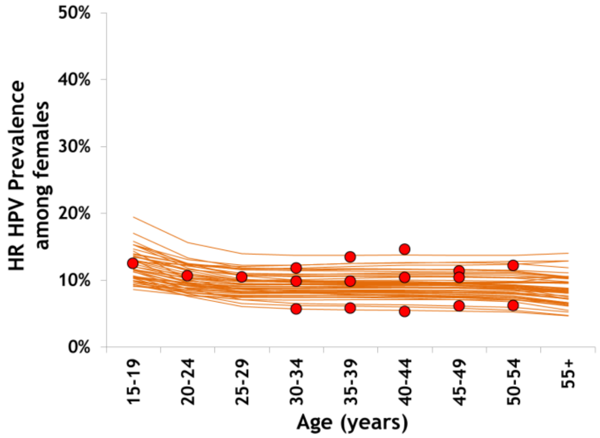

B) Vietnam

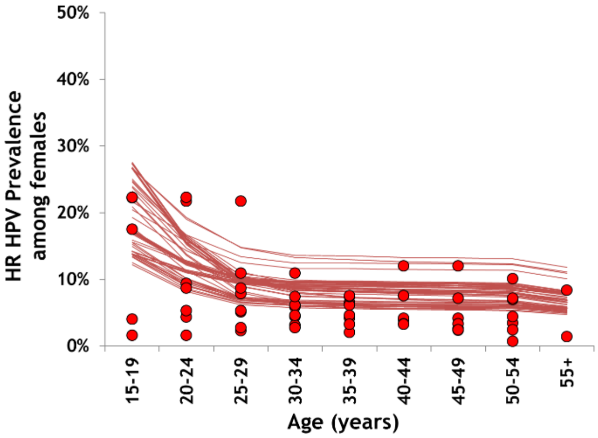

C) Uganda

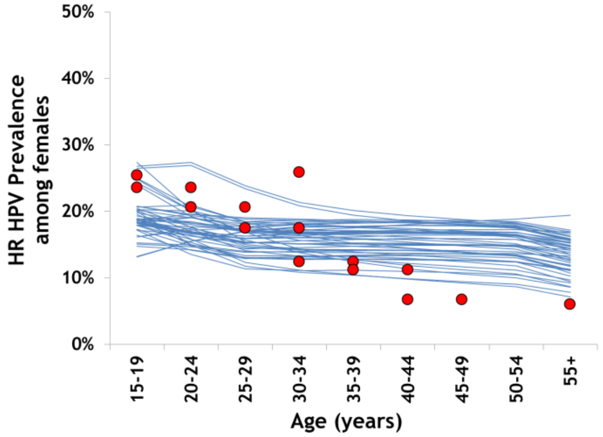

D) Nigeria

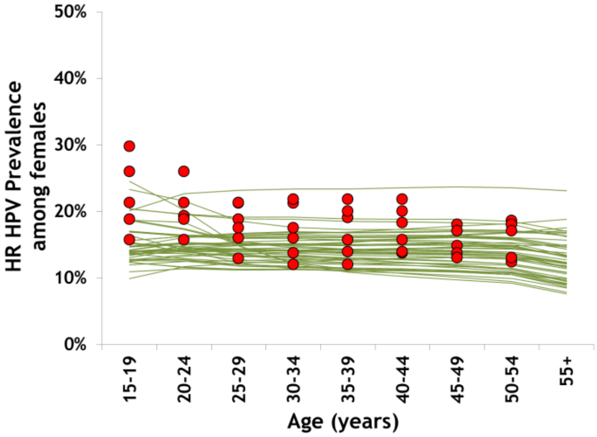

E) Benin

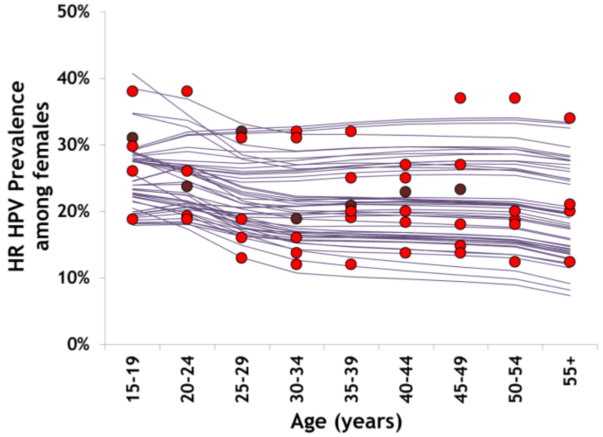

Figure A26. Fit to high risk HPV prevalence among sexually active females

Fit to prevalence of high risk HPV types infections among sexually active females for A) India, B) Vietnam, C) Uganda, D) Nigeria, and E) Benin. Solid lines represent the model predictions generated by the posterior parameter sets. For model predictions, we assumed a specificity of 99.7% for the HPV-test. Red dots represent the observed prevalence data (data sources for India: Sauvaget et al.[33], Basu et al.[34], and IARC prevalence data provided by Dr. Iacopo Baussano; Vietnam: Tran et al.[35], Vu et al. 2012[36], Van et al.[37], Anh et al.[38], and IARC prevalence data provided by Dr. Iacopo Baussano; Uganda: Asiimwe et al.[39], Mitchell et al.[40], Serwadda et al.[41], Safaeian et al. 2007[42], Safaeian et al. 2008[43], and Moses et al.[29]; Nigeria: Ezechi et al.[44], Gage et al.[45], Thomas et al.[46], Clarke et al.[47], Adebamowo et al.[48], and IARC prevalence data for Nigeria provided by Dr. Iacopo Baussano; Benin (due to scarcity of data for Benin, we also used regional data): Piras et al.[49] (Benin; shown in dark red on the graph), Jaquet et al.[51], Thomas et al.[46], Keita et al.[32], Gage et al.[45], and IARC prevalence data provided by Dr. Iacopo Baussano).

2.3.3 Fit to cervical cancer incidence data

Figure A27 show the fit to cervical cancer incidence data.

A) India

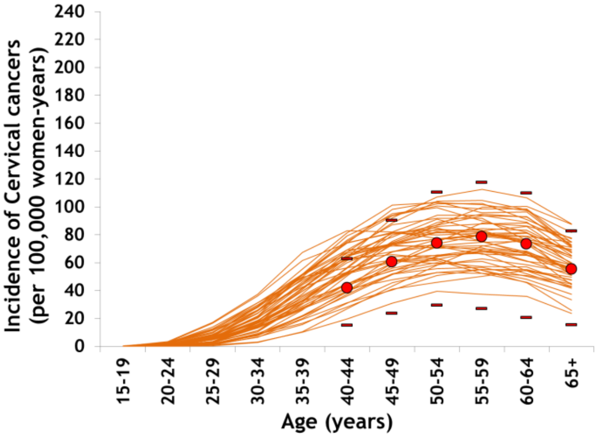

B) Vietnam

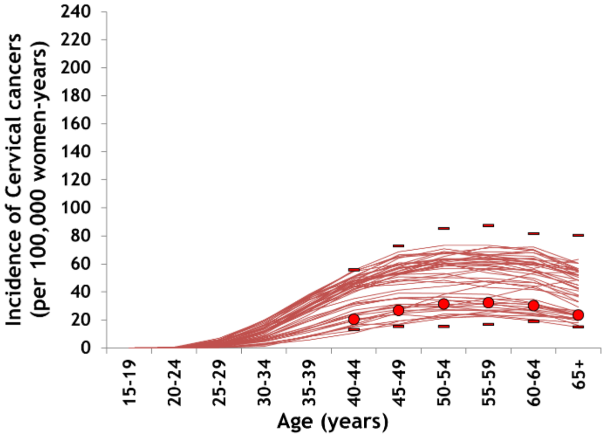

C) Uganda

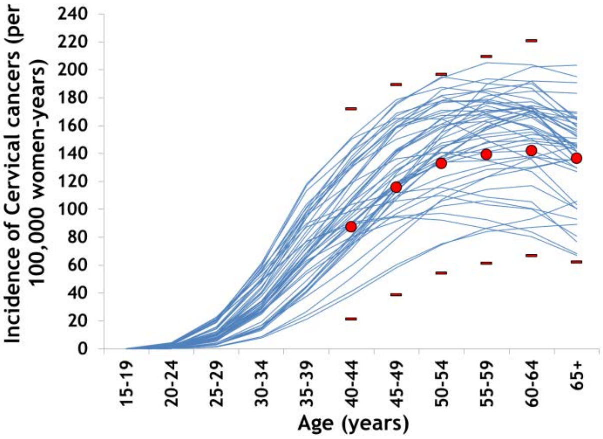

D) Nigeria

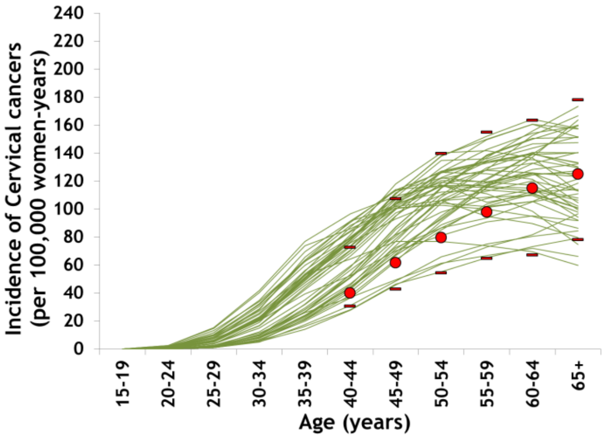

E) Benin

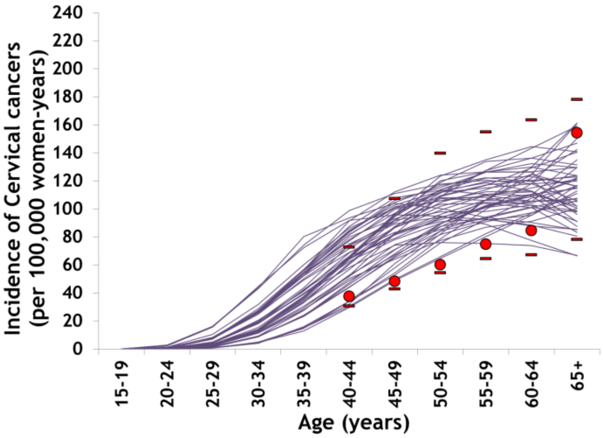

Figure A27. Fit to incidence of cervical cancer
Solid lines represent the model predictions generated by the posterior parameter sets for A) India, B) Vietnam, C) Uganda, D) Nigeria, and E) Benin. Red dots represent the observed data for each country from Globocan[71]. Red bars represent the variability of cervical cancer observed incidence for each world region and within each modeled country (India: Globocan[71]; Parkin et al.[72]; Vietnam: Globocan[71], Parkin et al.[72]; Uganda: Globocan[71], Parkin et al.[72]; Nigeria: Globocan[71]; Benin: Globocan[71])

2.3.4 Fit to HPV types distribution in cervical cancers

Figure A28 shows the model fit to the proportions of CC that are caused by the different HPV types for each modeled country.

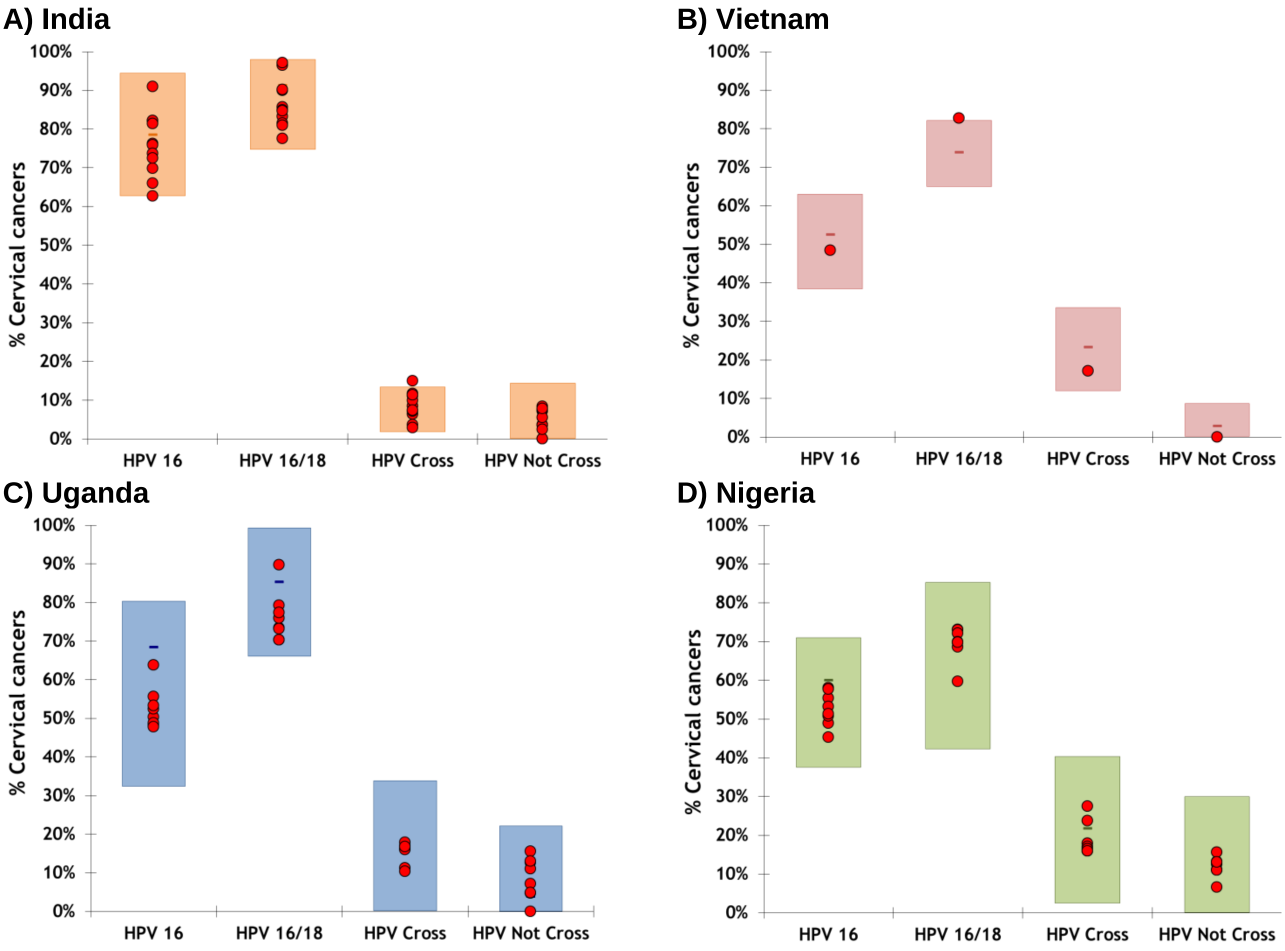

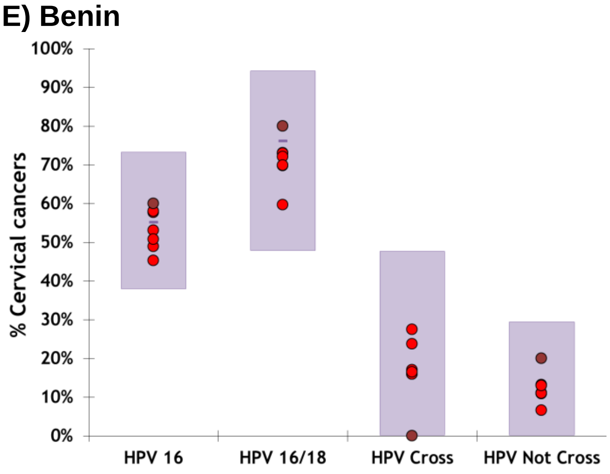

Figure A28. Fit to proportion of cervical cancers caused by HPV-types 16, 16/18, cross-protective types (31, 33, 45, 52, and 58), and not cross-protective (35, 39, 51, 56, 59, 66, 68, 73, and 82) Box plots represent the means, minimums and maximums of the model predictions generated by the posterior parameter sets for A) India, B) Vietnam, C) Uganda, D) Nigeria, and E) Benin. Red dots represent the observed data (data sources for India: Serrano et al.[65], Franceschi et al.[56], Munirajan et al.[57], Sowjanya et al.[58], Pillai et al.[53], Deodhar et al.[54], Srivastava et al.[55]; Vietnam: ICO Vietnam[59]; Uganda: Smith et al[60], Ndiaye et al.[61], Odida et al. 2008[62], Odida et al. 2011[63], Guan et al.[64], Serrano et al.[65]; Nigeria: Lin et al.[66], Bayo et al.[67], Denny et al.[68], Ndiaye et al.[61]; Benin (due to scarcity of data for Benin, we also used regional data): Bosch et al.[70] (Benin; shown in dark red on the graph), Lin et al.[66], Bayo et al.[67], Guan et al.[64], de Sanjosé et al.[50], Denny et al.[68], Ndiaye et al.[61]). Note: Multiple HPV infections in cervical cancers were added to single types in accordance with their relative weights among single type infections in cervical cancers.

2.4 Model validation

Model fit was cross-validated by comparing model predictions using the posterior parameter sets with observed data not used during the fitting procedure. Figure A29 to Figure A32 illustrate the proportion of males who ever had sex by age, the number of partners in the past 12 months in females, the mean lifetime number of partners among sexually active females and males, and the mean age of FSW male clients, respectively.

A) India

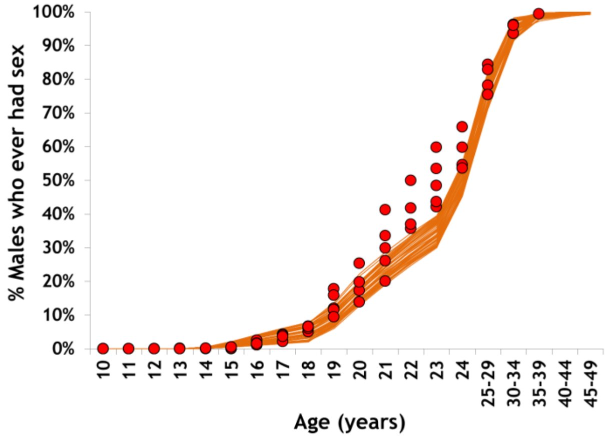

B) Vietnam

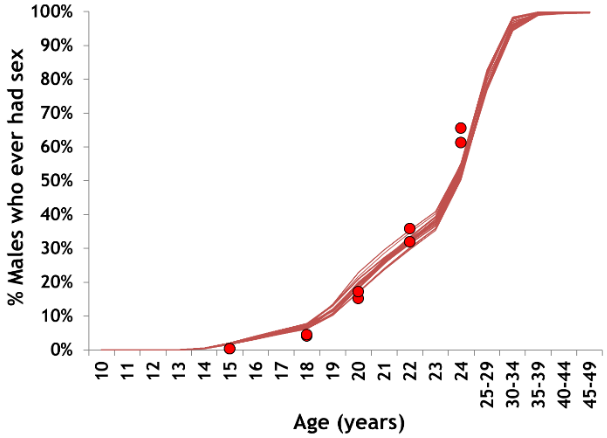

C) Uganda

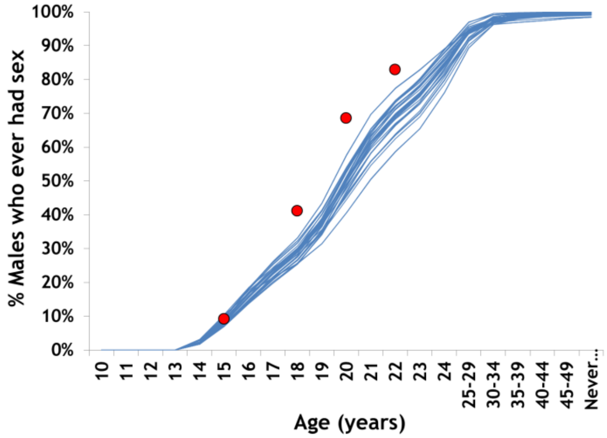

D) Nigeria

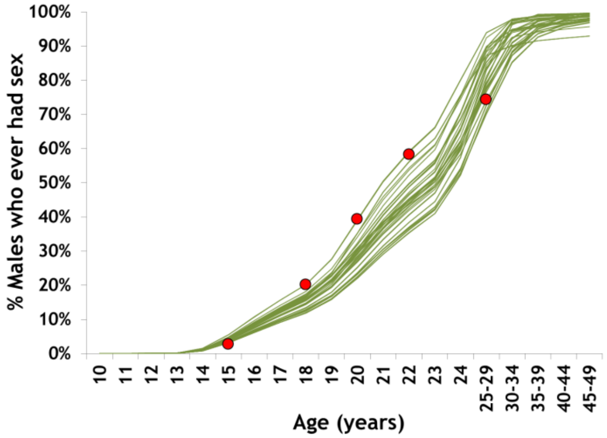

E) Benin

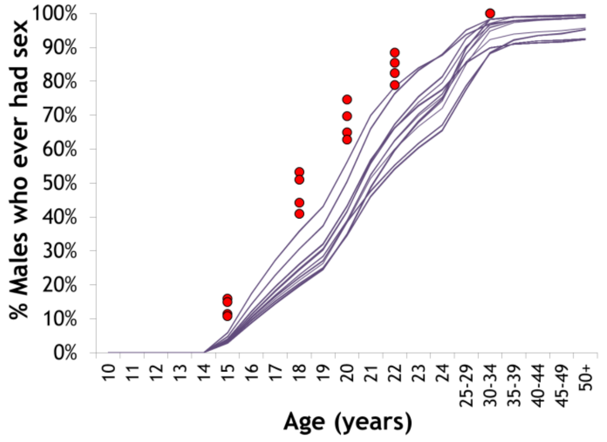

Figure A29. Proportion of sexually active males

Solid lines represent the model predictions generated by the posterior parameter sets for A) India, B) Vietnam, C) Uganda, D) Nigeria, and E) Benin. Red dots represent the observed data (data sources for India: GPS India[16], NFHS-3 India[15]; Vietnam: VPAIS[17]; Uganda: DHS Uganda[18]; Nigeria: DHS Nigeria[19]; Benin: DHS Benin 1996[20], DHS Benin 1[21], DHS Benin 2006[22], DHS Benin 2[23])

Figure A30. Distribution of the number of partners in the past 12 months
Number of partners in past 12 months in sexually active females and males aged 15-24 years in A-B) India, C-D) Vietnam, E-F) Uganda, and G-H) Nigeria. Box plots represent the medians, and 10th, 25th, 75th, and 90th percentiles of the model predictions generated by the posterior parameter sets. Red dots represent the observed data (data source for India: GPS India[16]; Vietnam: SAVY[88]; Uganda: UAIS[138]; Nigeria: no data; Benin: no data).

A) India females

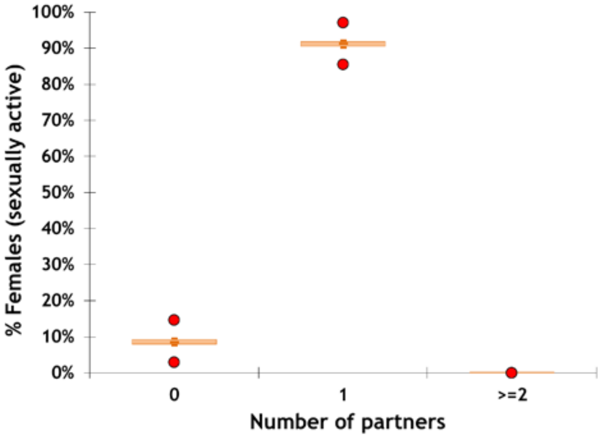

B) India males

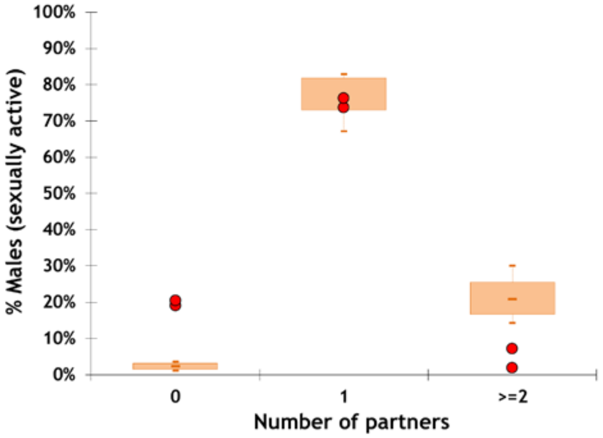

C) Vietnam females

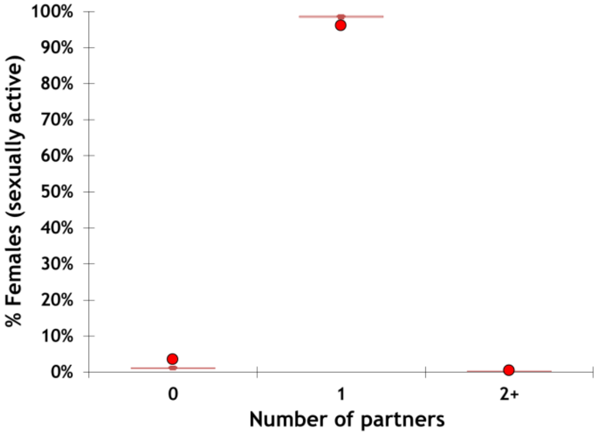

D) Vietnam males

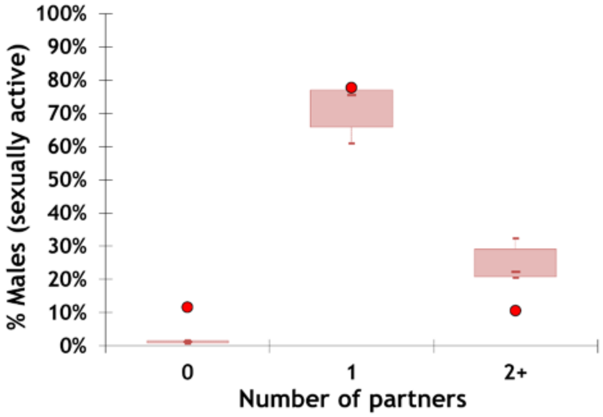

E) Uganda females

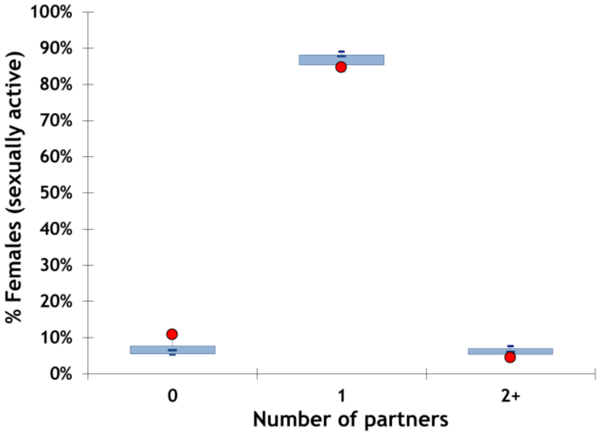

F) Uganda males

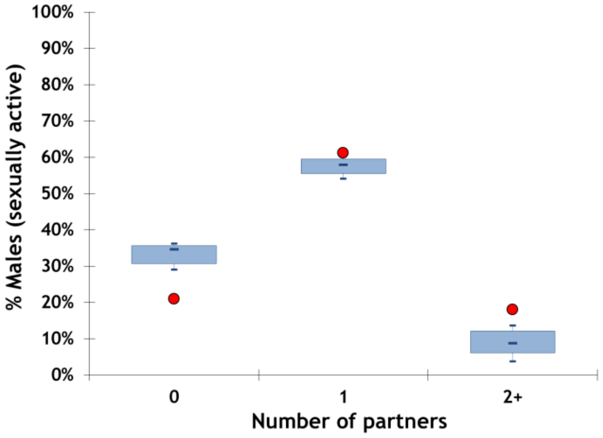

(Figure continued on next page…)

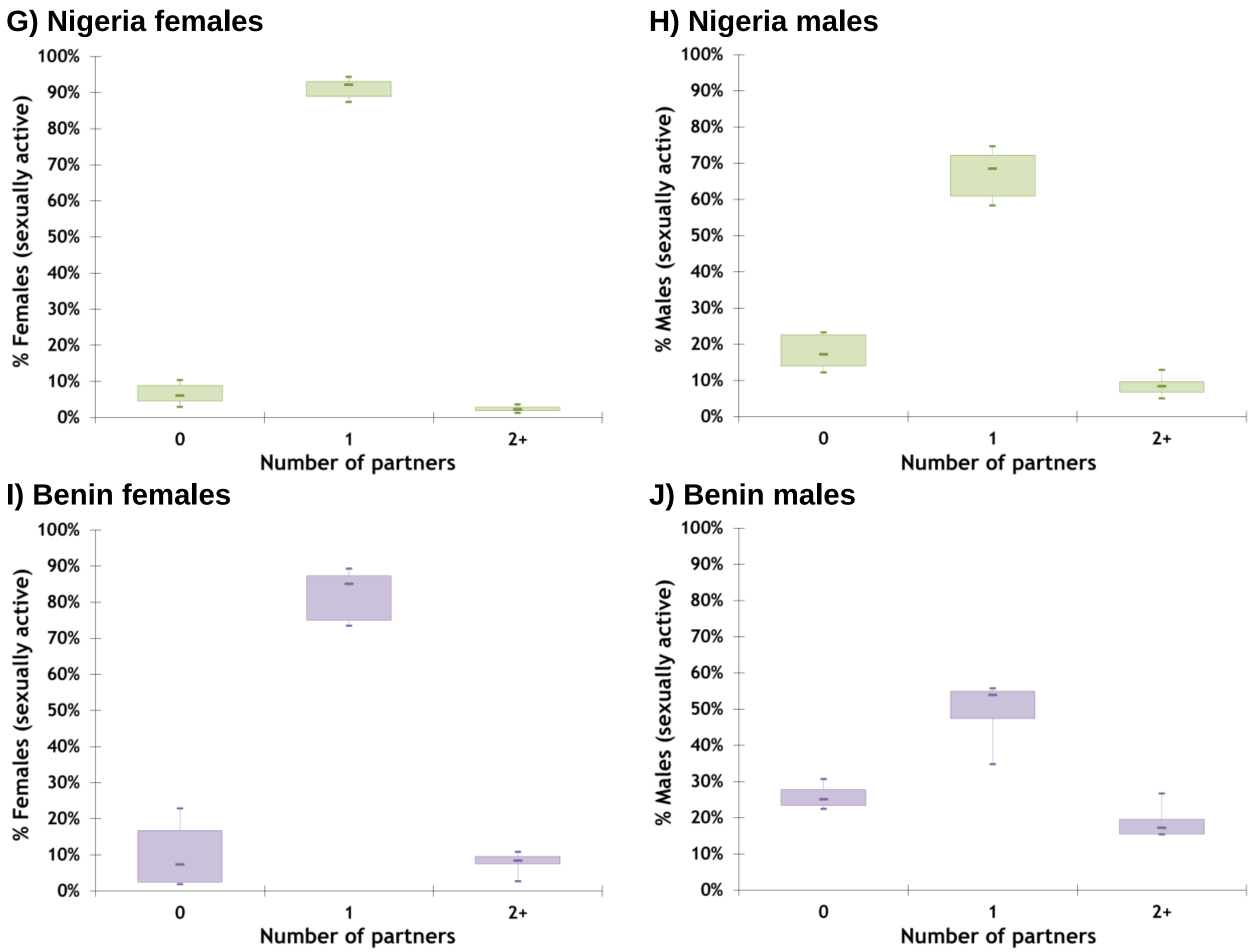
G) Nigeria females
H) Nigeria males
I) Benin females
J) Benin males
% Females (sexually active)
% Males (sexually active)
100%
90%
80%
70%
60%
50%
40%
30%
20%
10%
0%
0
1
2+
Number of partners

A) India

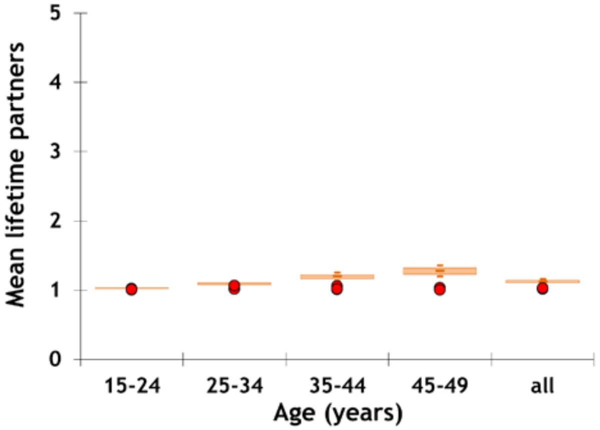

B) Vietnam

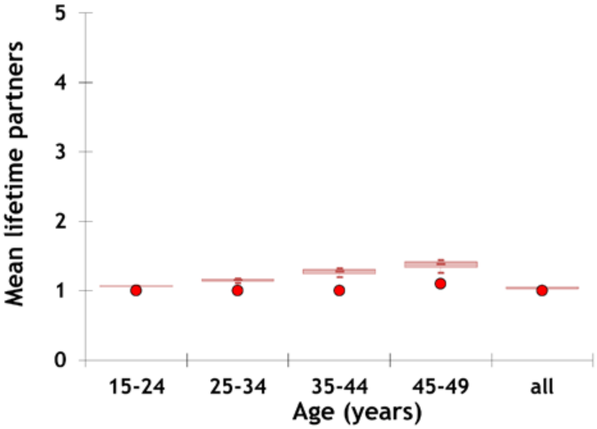

C) Uganda

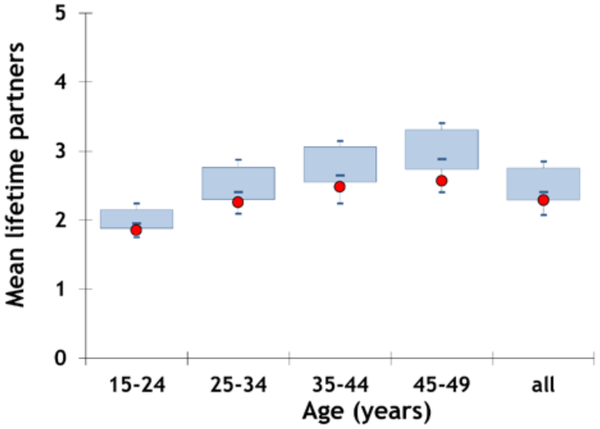

D) Nigeria

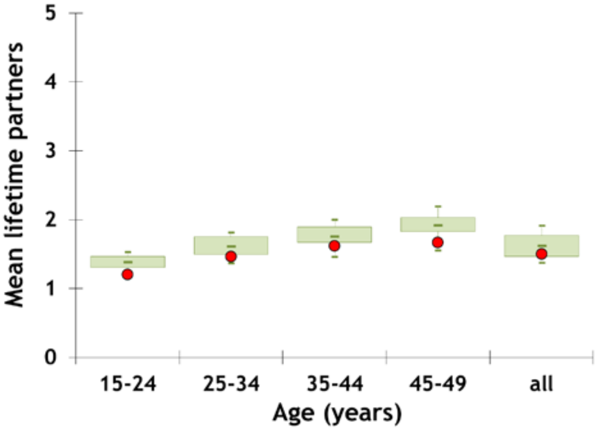

E) Benin

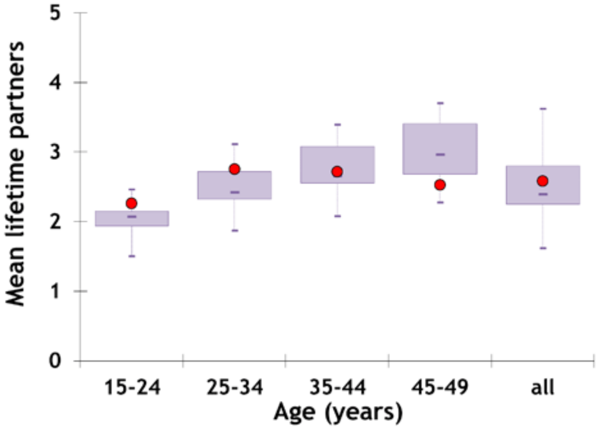

Figure A31. Mean lifetime number of partners among sexually active females
Box plots represent the medians, and 10th, 25th, 75th, and 90th percentiles of the model predictions generated by the posterior parameter sets for A) India, B) Vietnam, C) Uganda, D) Nigeria, and E) Benin. Red dots represent the observed data (data sources for <u>India</u>: GPS India[16]; <u>Vietnam</u>: VPAIS[17]; <u>Uganda</u>: DHS Uganda[18]; <u>Nigeria</u>: DHS Nigeria[19]; <u>Benin</u>: GPS Benin[24]).

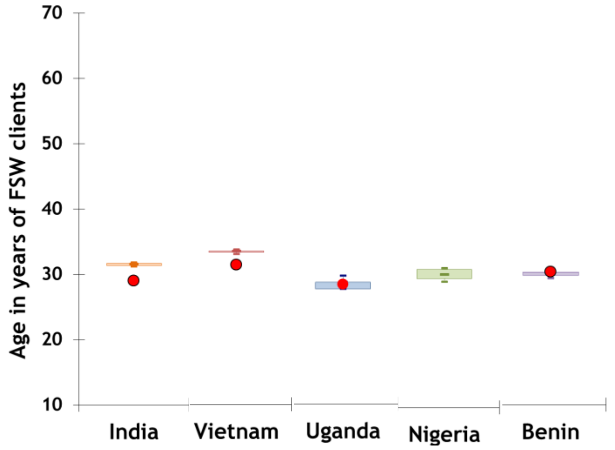

Figure A32. Mean age of clients of female sex workers (FSW)
Box plots represent the medians, and 10th, 25th, 75th, and 90th percentiles of the model predictions generated by the posterior parameter sets for A) India, B) Vietnam, C) Uganda, D) Nigeria, and E) Benin. Red dots represent the mean and 95% confidence Interval of FSW clients' age in the observed data (data sources for India: Suryawanshi et al.[139]; Vietnam: Nguyen et al.[140]; Uganda: UAIS[138]; Nigeria: no data; Benin: GPS Benin[24]).

2.5 Target definition

A prior parameter set is qualified as producing a ''good fit'', and included as a posterior parameter set, if the associated model predictions fall simultaneously within pre-specified targets (ranges) of the sexual behavior and epidemiological data defined in Table A1.

The lower and upper bounds of the target ranges are built as follows:

$$\text{Lower bound} = \min(O_{i,g,a,l}) - \xi_l$$
$$\text{Upper bound} = \max(O_{i,g,a,l}) + \xi_l \qquad (2.6)$$
$$\xi_l = f \cdot \max_{i,a}(O_{i,a,l})$$

O: specific data point

ξ: half-interval of target range

$\min/\max(\cdot)$: minimum/maximum value of all data sources for a specific data point $O_{i,g,a,l}$

$\max_{i,a}(\cdot)$: maximum value over all ages and data sources

f: takes values between 10% and 50% depending on the target to reflect data uncertainty.

i: data source

g: gender

a: age group of individual of gender g

l: sexual activity level of individual of gender g

This target definition allows for taking into account data uncertainty in the calibration procedure.

References

1. Van de Velde N, Brisson M, Boily MC. Understanding differences in predictions of HPV vaccine effectiveness: A comparative model-based analysis. *Vaccine.* 2010;28(33):5473-5484.
2. Van de Velde N, Boily MC, Drolet M, et al. Population-level impact of the bivalent, quadrivalent, and nonavalent human papillomavirus vaccines: a model-based analysis. *J Natl Cancer Inst.* 2012;104(22):1712-1723.
3. Brisson M, Laprise JF, Drolet M, et al. Comparative cost-effectiveness of the quadrivalent and bivalent human papillomavirus vaccines: a transmission-dynamic modeling study. *Vaccine.* 2013;31(37):3863-3871.
4. Brisson M, Van de Velde N, Drolet M, Laprise J-F, Boily M-C. Technical Appendix HPV-ADVISE Canada, 2012. Available at: http://www.marc-brisson.net/HPVadviseCEA.pdf. 2012.
5. Brisson M, Laprise JF, Chesson HW, et al. Health and Economic Impact of Switching from a 4-Valent to a 9-Valent HPV Vaccination Program in the United States. *J Natl Cancer Inst.* 2016;108(1).
6. Brisson M, Laprise JF, Drolet M, Van de Velde N, Boily MC. Technical Appendix HPV-ADVISE CDC, 2014. Available at http://marc-brisson.net/HPVadvise-US.pdf.
7. Boily MC, Anderson RM. Sexual contact patterns between men and women and the spread of HIV-1 in urban centres in Africa. *IMA J Math Appl Med Biol.* 1991;8(4):221-247.
8. Koutsky LA, Holmes KK, Critchlow CW, et al. A cohort study of the risk of cervical intraepithelial neoplasia grade 2 or 3 in relation to papillomavirus infection. *N Engl J Med.* 1992;327(18):1272-1278.
9. Nobbenhuis MA, Walboomers JM, Helmerhorst TJ, et al. Relation of human papillomavirus status to cervical lesions and consequences for cervical-cancer screening: a prospective study. *Lancet.* 1999;354(9172):20-25.
10. Rousseau MC, Pereira JS, Prado JC, Villa LL, Rohan TE, Franco EL. Cervical coinfection with human papillomavirus (HPV) types as a predictor of acquisition and persistence of HPV infection. *J Infect Dis.* 2001;184(12):1508-1517.
11. Thomas KK, Hughes JP, Kuypers JM, et al. Concurrent and sequential acquisition of different genital human papillomavirus types. *J Infect Dis.* 2000;182(4):1097-1102.
12. Winer RL, Lee SK, Hughes JP, Adam DE, Kiviat NB, Koutsky LA. Genital human papillomavirus infection: incidence and risk factors in a cohort of female university students. *Am J Epidemiol.* 2003;157(3):218-226.
13. Woodman CB, Collins S, Winter H, et al. Natural history of cervical human papillomavirus infection in young women: a longitudinal cohort study. *Lancet.* 2001;357(9271):1831-1836.
14. Van de Velde N, Brisson M, Boily MC. Modeling human papillomavirus vaccine effectiveness: quantifying the impact of parameter uncertainty. *Am J Epidemiol.* 2007;165(7):762-775.
15. International Institute for Population Sciences (IIPS) and Macro International. *National Family Health Survey (NFHS-3), 2005–06: India: Volume I. Mumbai: IIPS.* 2007.
16. Banandur P, Rajaram SP, Mahagaonkar SB, et al. Heterogeneity of the HIV epidemic in the general population of Karnataka state, south India. *BMC Public Health.* 2011;11 Suppl 6:S13.
17. General Statistics Office of Vietnam (GSO), National Institute of Hygiene and Epidemiology (NIHE) [Vietnam], ORC Macro. *Vietnam Population and AIDS Indicator Survey (VPAIS) 2005.* Calverton, Maryland, USA: GSO, NIHE, and ORC Macro; May 2006.
18. Uganda Bureau of Statistics (UBOS) and ICF. *Uganda Demographic and Health Survey 2016.* Kampala, Uganda and Rockville, Maryland, USA: UBOS and ICF2018.
19. National Population Commission (NPC) [Nigeria] and ICF International. *Nigeria Demographic and Health Survey 2013.* Abuja, Nigeria, and Rockville, Maryland, USA: NPC and ICF International;2014.
20. Kodjogbé N, Mboup G, Tossou J, et al. *Enquête Démographique et de Santé, République de Bénin 1996.* Calverton, Maryland USA: Institut National de la Statistique et de l'Analyse Économique et Macro International Inc;1997.

21. Institut National de la Statistique et de l'Analyse Économique (INSAE) et ORC Macro. *Enquête Démographique et de Santé au Bénin 2001.* Calverton, Maryland, USA: Institut National de la Statistique et de l'Analyse Économique et ORC Macro;2002.
22. Institut National de la Statistique et de l'Analyse Économique (INSAE) [Bénin], Macro International Inc. *Enquête Démographique et de Santé (EDSB-III) - Bénin 2006.* Calverton, Maryland, USA: Institut National de la Statistique et de l'Analyse Économique et Macro International Inc;2007.
23. Institut National de la Statistique et de l'Analyse Économique (INSAE), ICF International. *Enquête Démographique et de Santé du Bénin 2011-2012. Calverton, Maryland, USA: INSAE and ICF International.* 2013.
24. Behanzin L, Diabate S, Minani I, et al. Assessment of HIV-related risky behaviour: a comparative study of face-to-face interviews and polling booth surveys in the general population of Cotonou, Benin. *Sex Transm Infect.* 2013;89(7):595-601.
25. Dutta S, Begum R, Mazumder Indra D, et al. Prevalence of human papillomavirus in women without cervical cancer: a population-based study in Eastern India. *Int J Gynecol Pathol.* 2012;31(2):178-183.
26. Franceschi S, Herrero R, Clifford GM, et al. Variations in the age-specific curves of human papillomavirus prevalence in women worldwide. *Int J Cancer.* 2006;119(11):2677-2684.
27. Vu LT, Bui D, Le HT. Prevalence of cervical infection with HPV type 16 and 18 in Vietnam: implications for vaccine campaign. *BMC Cancer.* 2013;13:53.
28. Banura C, Mirembe FM, Katahoire AR, Namujju PB, Mbonye AK, Wabwire FM. Epidemiology of HPV genotypes in Uganda and the role of the current preventive vaccines: A systematic review. *Infect Agent Cancer.* 2011;6(1):11.
29. Moses E, Pedersen HN, Mitchell SM, et al. Uptake of community-based, self-collected HPV testing vs. visual inspection with acetic acid for cervical cancer screening in Kampala, Uganda: preliminary results of a randomised controlled trial. *Trop Med Int Health.* 2015;20(10):1355-1367.
30. Kumakech E, Berggren V, Wabinga H, et al. Significantly Reduced Genoprevalence of Vaccine-Type HPV-16/18 Infections among Vaccinated Compared to Non-Vaccinated Young Women 5.5 Years after a Bivalent HPV-16/18 Vaccine (Cervarix(R)) Pilot Project in Uganda. *PLoS One.* 2016;11(8):e0160099.
31. Ouedraogo CM, Djigma FW, Bisseye C, et al. [Epidemiology, characterization of genotypes of human papillomavirus in a population of women in Ouagadougou]. *J Gynecol Obstet Biol Reprod (Paris).* 2011;40(7):633-638.
32. Keita N, Clifford GM, Koulibaly M, et al. HPV infection in women with and without cervical cancer in Conakry, Guinea. *Br J Cancer.* 2009;101(1):202-208.
33. Sauvaget C, Nene BM, Jayant K, et al. Prevalence and determinants of high-risk human papillomavirus infection in middle-aged Indian women. *Sex Transm Dis.* 2011;38(10):902-906.
34. Basu P, Mittal S, Bhaumik S, et al. Prevalence of high-risk human papillomavirus and cervical intraepithelial neoplasias in a previously unscreened population--a pooled analysis from three studies. *Int J Cancer.* 2013;132(7):1693-1699.
35. Tran LT, Tran LT, Bui TC, et al. Risk factors for high-risk and multi-type Human Papillomavirus infections among women in Ho Chi Minh City, Vietnam: a cross-sectional study. *BMC Womens Health.* 2015;15:16.
36. Vu LT, Bui D. Prevalence of cervical human papilloma virus infection among married women in Vietnam, 2011. *Asian Pac J Cancer Prev.* 2012;13(1):37-40.
37. Van SN, Khac MN, Dimberg J, Matussek A, Henningsson AJ. Prevalence of Cervical Infection and Genotype Distribution of Human Papilloma Virus Among Females in Da Nang, Vietnam. *Anticancer Res.* 2017;37(3):1243-1247.
38. Pham TH, Nguyen TH, Herrero R, et al. Human papillomavirus infection among women in South and North Vietnam. *Int J Cancer.* 2003;104(2):213-220.

39. Asiimwe S, Whalen CC, Tisch DJ, Tumwesigye E, Sethi AK. Prevalence and predictors of high-risk human papillomavirus infection in a population-based sample of women in rural Uganda. *Int J STD AIDS.* 2008;19(9):605-610.
40. Mitchell SM, Sekikubo M, Biryabarema C, et al. Factors associated with high-risk HPV positivity in a low-resource setting in sub-Saharan Africa. *Am J Obstet Gynecol.* 2014;210(1):81 e81-87.
41. Serwadda D, Wawer MJ, Shah KV, et al. Use of a hybrid capture assay of self-collected vaginal swabs in rural Uganda for detection of human papillomavirus. *J Infect Dis.* 1999;180(4):1316-1319.
42. Safaeian M, Kiddugavu M, Gravitt PE, et al. Comparability of self-collected vaginal swabs and physician-collected cervical swabs for detection of human papillomavirus infections in Rakai, Uganda. *Sex Transm Dis.* 2007;34(7):429-436.
43. Safaeian M, Kiddugavu M, Gravitt PE, et al. Prevalence and risk factors for carcinogenic human papillomavirus infections in rural Rakai, Uganda. *Sex Transm Infect.* 2008;84(4):306-311.
44. Ezechi OC, Ostergren PO, Nwaokorie FO, Ujah IA, Odberg Pettersson K. The burden, distribution and risk factors for cervical oncogenic human papilloma virus infection in HIV positive Nigerian women. *Virol J.* 2014;11:5.
45. Gage JC, Ajenifuja KO, Wentzensen NA, et al. The age-specific prevalence of human papillomavirus and risk of cytologic abnormalities in rural Nigeria: implications for screen-and-treat strategies. *Int J Cancer.* 2012;130(9):2111-2117.
46. Thomas JO, Herrero R, Omigbodun AA, et al. Prevalence of papillomavirus infection in women in Ibadan, Nigeria: a population-based study. *Br J Cancer.* 2004;90(3):638-645.
47. Clarke MA, Gage JC, Ajenifuja KO, et al. A population-based cross-sectional study of age-specific risk factors for high risk human papillomavirus prevalence in rural Nigeria. *Infect Agent Cancer.* 2011;6:12.
48. Adebamowo SN, Olawande O, Famooto A, et al. Persistent Low-Risk and High-Risk Human Papillomavirus Infections of the Uterine Cervix in HIV-Negative and HIV-Positive Women. *Front Public Health.* 2017;5:178.
49. Piras F, Piga M, De Montis A, et al. Prevalence of human papillomavirus infection in women in Benin, West Africa. *Virol J.* 2011;8:514.
50. de Sanjose S, Diaz M, Castellsague X, et al. Worldwide prevalence and genotype distribution of cervical human papillomavirus DNA in women with normal cytology: a meta-analysis. *Lancet Infect Dis.* 2007;7(7):453-459.
51. Jaquet A, Horo A, Charbonneau V, et al. Cervical human papillomavirus and HIV infection in women of child-bearing age in Abidjan, Cote d'Ivoire, 2010. *Br J Cancer.* 2012;107(3):556-563.
52. Serrano B, Alemany L, Ruiz PA, et al. Potential impact of a 9-valent HPV vaccine in HPV-related cervical disease in 4 emerging countries (Brazil, Mexico, India and China). *Cancer epidemiology.* 2014;38(6):748-756.
53. Pillai RM, Babu JM, Jissa VT, et al. Region-wise distribution of high-risk human papillomavirus types in squamous cell carcinomas of the cervix in India. *Int J Gynecol Cancer.* 2010;20(6):1046-1051.
54. Deodhar K, Gheit T, Vaccarella S, et al. Prevalence of human papillomavirus types in cervical lesions from women in rural Western India. *J Med Virol.* 2012;84(7):1054-1060.
55. Srivastava S, Shahi UP, Dibya A, Gupta S, Roy JK. Distribution of HPV Genotypes and Involvement of Risk Factors in Cervical Lesions and Invasive Cervical Cancer: A Study in an Indian Population. *Int J Mol Cell Med.* 2014;3(2):61-73.
56. Franceschi S, Rajkumar T, Vaccarella S, et al. Human papillomavirus and risk factors for cervical cancer in Chennai, India: a case-control study. *Int J Cancer.* 2003;107(1):127-133.
57. Munirajan AK, Kannan K, Bhuvarahamurthy V, et al. The status of human papillomavirus and tumor suppressor genes p53 and p16 in carcinomas of uterine cervix from India. *Gynecol Oncol.* 1998;69(3):205-209.

58. Sowjanya AP, Jain M, Poli UR, et al. Prevalence and distribution of high-risk human papilloma virus (HPV) types in invasive squamous cell carcinoma of the cervix and in normal women in Andhra Pradesh, India. *BMC Infect Dis.* 2005;5:116.
59. Bruni L, Barrionuevo-Rosas L, Albero G, et al. *ICO Information Centre on HPV and Cancer (HPV Information Centre). Human Papillomavirus and Related Diseases in Viet Nam. Summary Report 27 July 2017. Accessed November 2017.*
60. Smith JS, Lindsay L, Hoots B, et al. Human papillomavirus type distribution in invasive cervical cancer and high-grade cervical lesions: a meta-analysis update. *Int J Cancer.* 2007;121(3):621-632.
61. Ndiaye C, Alemany L, Ndiaye N, et al. Human papillomavirus distribution in invasive cervical carcinoma in sub-Saharan Africa: could HIV explain the differences? *Trop Med Int Health.* 2012;17(12):1432-1440.
62. Odida M, de Sanjose S, Quint W, Bosch XF, Klaustermeier J, Weiderpass E. Human Papillomavirus type distribution in invasive cervical cancer in Uganda. *BMC Infect Dis.* 2008;8:85.
63. Odida M, Sandin S, Mirembe F, Kleter B, Quint W, Weiderpass E. HPV types, HIV and invasive cervical carcinoma risk in Kampala, Uganda: a case-control study. *Infect Agent Cancer.* 2011;6(1):8.
64. Guan P, Howell-Jones R, Li N, et al. Human papillomavirus types in 115,789 HPV-positive women: A meta-analysis from cervical infection to cancer. *Int J Cancer.* 2012;131(10):2349-2359.
65. Serrano B, Alemany L, Tous S, et al. Potential impact of a nine-valent vaccine in human papillomavirus related cervical disease. *Infect Agent Cancer.* 2012;7(1):38.
66. Lin P, Koutsky LA, Critchlow CW, et al. HLA class II DR-DQ and increased risk of cervical cancer among Senegalese women. *Cancer Epidemiol Biomarkers Prev.* 2001;10(10):1037-1045.
67. Bayo S, Bosch FX, de Sanjose S, et al. Risk factors of invasive cervical cancer in Mali. *Int J Epidemiol.* 2002;31(1):202-209.
68. Denny L, Adewole I, Anorlu R, et al. Human papillomavirus prevalence and type distribution in invasive cervical cancer in sub-Saharan Africa. *Int J Cancer.* 2014;134(6):1389-1398.
69. de Sanjose S, Quint WG, Alemany L, et al. Human papillomavirus genotype attribution in invasive cervical cancer: a retrospective cross-sectional worldwide study. *Lancet Oncol.* 2010;11(11):1048-1056.
70. Bosch FX, Manos MM, Munoz N, et al. Prevalence of human papillomavirus in cervical cancer: a worldwide perspective. International biological study on cervical cancer (IBSCC) Study Group. *J Natl Cancer Inst.* 1995;87(11):796-802.
71. International Agency for Research on Cancer (IARC). Incidence/Mortality, Age-specific tables, Cervix Uteri. *GLOBOCAN 2012: Estimated Cancer Incidence, Mortality and Prevalence Worldwide in 2012* http://gco.iarc.fr/today/home. Accessed March, 2017.
72. Parkin DM, Whelan SL, Ferlay J, Teppo L, Thomas DB, Editors. *Cancer Incidence in Five Continents Vol. VIII.* 2002.
73. Vuong DA, Velasco-Garrido M, Lai TD, Busse R. Temporal trends of cancer incidence in Vietnam, 1993-2007. *Asian Pac J Cancer Prev.* 2010;11(3):739-745.
74. World Health Organization (WHO). Global Health Observatory data repository: Life tables by country. http://apps.who.int/gho/data/node.main.LIFECOUNTRY?lang=en. Accessed September, 2017.
75. Brisson M, Bénard E, Drolet M, et al. Population-level impact, herd immunity and elimination after HPV vaccination: a systematic review and meta-analysis of predictions of transmission-dynamic models. *Lancet Public Health.* 2016;1(1):e8-e17.
76. Franceschi S, Rajkumar R, Snijders PJ, et al. Papillomavirus infection in rural women in southern India. *Br J Cancer.* 2005;92(3):601-606.
77. Hernandez BY, Vu Nguyen T. Cervical human papillomavirus infection among female sex workers in southern Vietnam. *Infect Agent Cancer.* 2008;3:7.

78. United Nations Statistics Division (UNSD). Population by marital status, age, sex and urban/rural residence: India (2001). http://data.un.org/Data.aspx?d=POP&f=tableCode%3a23%3bcountryCode%3a356%3brefYear%3a2001&c=2,3,6,8,10,12,14,16,17,18&s=_countryEnglishNameOrderBy:asc,refYear:desc,areaCode:asc&v=1. Accessed November, 2017.
79. Lowndes CM, Jayachandran AA, Banandur P, et al. Polling booth surveys: a novel approach for reducing social desirability bias in HIV-related behavioural surveys in resource-poor settings. *AIDS and behavior.* 2012;16(4):1054-1062.
80. Gaffey MF, Venkatesh S, Dhingra N, et al. Male Use of Female Sex Work in India: A Nationally Representative Behavioural Survey. *PLoS One.* 2011;6(7):e22704.
81. Mishra S, Mountain E, Pickles M, et al. Exploring the population-level impact of antiretroviral treatment: the influence of baseline intervention context. *AIDS.* 2014;28 Suppl 1:S61-72.
82. Vandepitte J, Lyerla R, Dallabetta G, Crabbe F, Alary M, Buve A. Estimates of the number of female sex workers in different regions of the world. *Sex Transm Infect.* 2006;82 Suppl 3:iii18-25.
83. United nations Statistics Division (UNSD). Population by marital status, age, sex and urban/rural residence: Viet Nam (1989). http://data.un.org/Data.aspx?q=viet+nam+1989+marital&d=POP&f=tableCode:23;countryCode:704;refYear:1989&c=2,3,6,8,10,12,14,16,17,18&s=_countryEnglishNameOrderBy:asc,refYear:desc,areaCode:asc&v=1. Accessed November, 2017.
84. General Statistics Office of Vietnam (GSO), United Nations Population Fund (UNFPA). *The 1/4/2011 Population Change and Family Planning Survey: Major Findings.* Hanoi, Vietnam 2011.
85. Central Population and Housing Census Steering Committee. *The 2009 Vietnam Population and Housing Census: Major Findings.* Hanoi, Vietnam2010.
86. General Statistics Office of Vietnam (GSO), UNICEF. *Viet Nam Multiple Indicator Cluster Survey (MICS) 2011. Final Report.* Hanoi, Vietnam;2011.
87. General Statistics Office of Vietnam (GSO), UNICEF. *Viet Nam Multiple Indicator Cluster Survey (MICS) 2014. Final Report.* Hanoi, Vietnam;2015.
88. Vietnam Ministry of Health and General Statistics Office of Vietnam (GSO). *Survey and Assessment of Vietnamese Youth (SAVY), 2003. ICPSR24387-v1. Ann Arbor, MI: Inter-university Consortium for Political and Social Research [distributor], 2009-01-13. https://doi.org/10.3886/ICPSR24387.v1.* 2003.
89. Vietnam Ministry of Health, General Statistics Office of Vietnam (GSO), World Health Organization, UNICEF. *Survey Assessment of Vietnamese Youth Round 2. (SAVY 2).* Hanoi, Vietnam;2010.
90. Vietnam Ministry of Health. *Results from the HIV/STI Integrated Biological and Behavioral Surveillance (IBBS) in Vietnam 2005-2006.* 2006.
91. Vietnam Technical Working Group on HIV Estimates and Projections (VTWG). *Vietnam HIV/AIDS Estimates and Projections, 2007-2012.* Hanoi, Vietnam;2009.
92. Bui TD, Pham CK, Pham TH, et al. Cross-sectional study of sexual behaviour and knowledge about HIV among urban, rural, and minority residents in Viet Nam. *Bull World Health Organ.* 2001;79(1):15-21.
93. Ghuman S, Loi VM, Huy VT, Knodel J. Continuity and change in premarital sex in Vietnam. *Int Fam Plan Perspect.* 2006;32(4):166-174.
94. Knodel J, Huy VT, Loi VM, Ghuman S. *Marital Sexual Behavior and Aging in Vietnam in Comparative Perspective - Report 05-583.* Population Studies Center, University of Michigan Institute for Social Research;2005.
95. Le TT, Nguyen QC, Tran HT, Schwandt M, Lim HJ. Correlates of HIV infection among street-based and venue-based sex workers in Vietnam. *Int J STD AIDS.* 2016;27(12):1093-1103.
96. Le LV, Nguyen TA, Tran HV, et al. Correlates of HIV infection among female sex workers in Vietnam: injection drug use remains a key risk factor. *Drug Alcohol Depend.* 2015;150:46-53.

97. Tran TN, Detels R, Lan HP. Condom use and its correlates among female sex workers in Hanoi, Vietnam. *AIDS and behavior.* 2006;10(2):159-167.
98. United nations Statistics Division (UNSD). Population by marital status, age, sex and urban/rural residence: Uganda (2002). http://data.un.org/Data.aspx?q=viet+nam+1989+marital&d=POP&f=tableCode%3a23%3bcountryCode%3a800&c=2,3,6,8,10,12,14,16,17,18&s=_countryEnglishNameOrderBy:asc,refYear:desc,areaCode:asc&v=1. Accessed November, 2017.
99. Todd J, Cremin I, McGrath N, et al. Reported number of sexual partners: comparison of data from four African longitudinal studies. *Sex Transm Infect.* 2009;85 Suppl 1:i72-80.
100. HIV/AIDS Knowledge Management and Communication Capacity (KMCC). *Sex Workers and HIV/AIDS in Uganda: Synthesis of Information and Evidence to Inform the Response.* 2014.
101. Uganda AIDS Commission. *The HIV and AIDS Uganda country progress report 2014.* 2015.
102. Muldoon KA. A systematic review of the clinical and social epidemiological research among sex workers in Uganda. *BMC Public Health.* 2015;15:1226.
103. Pickering H, Okongo M, Nnalusiba B, Bwanika K, Whitworth J. Sexual networks in Uganda: casual and commercial sex in a trading town. *AIDS Care.* 1997;9(2):199-207.
104. Nagaddya T, Wooding N, Nabankema E. *Understanding the dynamics and practices of female sex workers with both circumcised and non-circumcised men in Makindye Division.* International Health Sciences University;2014.
105. Vandepitte J, Bukenya J, Weiss HA, et al. HIV and other sexually transmitted infections in a cohort of women involved in high-risk sexual behavior in Kampala, Uganda. *Sex Transm Dis.* 2011;38(4):316-323.
106. Kelly RJ, Gray RH, Sewankambo NK, et al. Age differences in sexual partners and risk of HIV-1 infection in rural Uganda. *J Acquir Immune Defic Syndr.* 2003;32(4):446-451.
107. United Nations Statistics Division (UNSD). Population by marital status, age, sex and urban/rural residence: Nigeria (1991). http://data.un.org/Data.aspx?d=POP&f=tableCode%3a23%3bcountryCode%3a566%3brefYear%3a1991&c=2,3,6,8,10,12,14,16,17,18&s=_countryEnglishNameOrderBy:asc,refYear:desc,areaCode:asc&v=1. Accessed November, 2017.
108. Ibisomi L. Is age difference between partners associated with contraceptive use among married couples in Nigeria? *Int Perspect Sex Reprod Health.* 2014;40(1):39-45.
109. Ikpeazu A, Momah-Haruna A, Madu Mari B, et al. An appraisal of female sex work in Nigeria--implications for designing and scaling up HIV prevention programmes. *PLoS One.* 2014;9(8):e103619.
110. Eluwa GI, Strathdee SA, Adebajo SB, Ahonsi B, Azeez A, Anyanti J. Sexual risk behaviors and HIV among female sex workers in Nigeria. *J Acquir Immune Defic Syndr.* 2012;61(4):507-514.
111. Stephenson R, Winter A, Elfstrom M. Community environments shaping transactional sex among sexually active men in Malawi, Nigeria, and Tanzania. *AIDS Care.* 2013;25(6):784-792.
112. Wellings K, Collumbien M, Slaymaker E, et al. Sexual behaviour in context: a global perspective. *Lancet.* 2006;368(9548):1706-1728.
113. Ahoyo AB, Alary M, Meda H, et al. [Female sex workers in Benin, 2002. Behavioural survey and HIV and other STI screening]. *Sante.* 2007;17(3):143-151.
114. Duong CT, Nguyen TH, Hoang TT, et al. Sexual risk and bridging behaviors among young people in Hai Phong, Vietnam. *AIDS and behavior.* 2008;12(4):643-651.
115. Beachler DC, Jenkins G, Safaeian M, Kreimer AR, Wentzensen N. Natural Acquired Immunity Against Subsequent Genital Human Papillomavirus Infection: A Systematic Review and Meta-analysis. *J Infect Dis.* 2016;213(9):1444-1454.
116. Myers ER, McCrory DC, Nanda K, Bastian L, Matchar DB. Mathematical model for the natural history of human papillomavirus infection and cervical carcinogenesis. *Am J Epidemiol.* 2000;151(12):1158-1171.
117. Bruni L, Barrionuevo-Rosas L, Albero G, et al. *ICO Information Centre on HPV and Cancer (HPV Information Centre). Human Papillomavirus and Related Diseases in India. Summary Report 27 July 2017. Accessed November 2017.*

118. Gakidou E, Nordhagen S, Obermeyer Z. Coverage of cervical cancer screening in 57 countries: low average levels and large inequalities. *PLoS Med.* 2008;5(6):e132.
119. Hoang A, Nguyen CQ, Duong CD. Youth experiences in accessing sexual healthcare services in Vietnam. *Cult Health Sex.* 2018;20(5):545-559.
120. World Health Organization (WHO). *World Health Survey Results - Report of Vietnam (2003).* http://apps.who.int/healthinfo/systems/surveydata/index.php/catalog/92. Accessed March, 2018.
121. Ndejjo R, Mukama T, Musabyimana A, Musoke D. Uptake of Cervical Cancer Screening and Associated Factors among Women in Rural Uganda: A Cross Sectional Study. *PLoS One.* 2016;11(2):e0149696.
122. Bruni L, Barrionuevo-Rosas L, Albero G, et al. *ICO/IARC Information Centre on HPV and Cancer (HPV Information Centre). Human Papillomavirus and Related Diseases in Nigeria. Summary Report 27 July 2017. Accessed May 2018.*
123. Bruni L, Barrionuevo-Rosas L, Albero G, et al. *ICO Information Centre on HPV and Cancer (HPV Information Centre). Human Papillomavirus and Related Diseases in Benin. Summary Report 27 July 2017. Accessed January 2018.*
124. Bruni L, Albero G, Serrano B, et al. *ICO/IARC Information Centre on HPV and Cancer (HPV Information Centre). Human Papillomavirus and Related Diseases in Uganda. Summary Report 10 December 2018. Accessed March 2019.*
125. Domingo EJ, Noviani R, Noor MR, et al. Epidemiology and prevention of cervical cancer in Indonesia, Malaysia, the Philippines, Thailand and Vietnam. *Vaccine.* 2008;26 Suppl 12:M71-79.
126. Nanda K, McCrory DC, Myers ER, et al. Accuracy of the Papanicolaou test in screening for and follow-up of cervical cytologic abnormalities: a systematic review. *Ann Intern Med.* 2000;132(10):810-819.
127. Arbyn M, Bergeron C, Klinkhamer P, Martin-Hirsch P, Siebers AG, Bulten J. Liquid compared with conventional cervical cytology: a systematic review and meta-analysis. *Obstet Gynecol.* 2008;111(1):167-177.
128. Martin-Hirsch P, Rash B, Martin A, Standaert B. Management of women with abnormal cervical cytology: treatment patterns and associated costs in England and Wales. *Bjog.* 2007;114(4):408-415.
129. Wright TC, Jr., Denny L, Kuhn L, Pollack A, Lorincz A. HPV DNA testing of self-collected vaginal samples compared with cytologic screening to detect cervical cancer. *Jama.* 2000;283(1):81-86.
130. Gage JC, Hanson VW, Abbey K, et al. Number of cervical biopsies and sensitivity of colposcopy. *Obstet Gynecol.* 2006;108(2):264-272.
131. Chase DM, Kalouyan M, DiSaia PJ. Colposcopy to evaluate abnormal cervical cytology in 2008. *Am J Obstet Gynecol.* 2009;200(5):472-480.
132. Mitchell MF, Schottenfeld D, Tortolero-Luna G, Cantor SB, Richards-Kortum R. Colposcopy for the diagnosis of squamous intraepithelial lesions: a meta-analysis. *Obstet Gynecol.* 1998;91(4):626-631.
133. Da Forno PD, Holbrook MR, Nunns D, Shaw PA. Long-term follow-up of patients following negative colposcopy: a new gold standard and its implications for cervical screening. *Cytopathology.* 2003;14(5):281-286.
134. Cai B, Ronnett BM, Stoler M, et al. Longitudinal evaluation of interobserver and intraobserver agreement of cervical intraepithelial neoplasia diagnosis among an experienced panel of gynecologic pathologists. *Am J Surg Pathol.* 2007;31(12):1854-1860.
135. Goldie SJ, Gaffikin L, Goldhaber-Fiebert JD, et al. Cost-effectiveness of cervical-cancer screening in five developing countries. *N Engl J Med.* 2005;353(20):2158-2168.
136. Martin-Hirsch PP, Paraskevaidis E, Bryant A, Dickinson HO, Keep SL. Surgery for cervical intraepithelial neoplasia. *Cochrane Database Syst Rev.* 2009;6:CD001318.

137. Kreimer AR, Katki HA, Schiffman M, Wheeler CM, Castle PE. Viral determinants of human papillomavirus persistence following loop electrical excision procedure treatment for cervical intraepithelial neoplasia grade 2 or 3. *Cancer Epidemiol Biomarkers Prev.* 2007;16(1):11-16.
138. Uganda Ministry of Health. *Uganda AIDS Indicator Survey (UAIS).* 2011.
139. Suryawanshi D, Bhatnagar T, Deshpande S, Zhou W, Singh P, Collumbien M. Diversity among clients of female sex workers in India: comparing risk profiles and intervention impact by site of solicitation. implications for the vulnerability of less visible female sex workers. *PLoS One.* 2013;8(9):e73470.
140. Nguyen NT, Nguyen HT, Trinh HQ, Mills SJ, Detels R. Clients of female sex workers as a bridging population in Vietnam. *AIDS and behavior.* 2009;13(5):881-891.
