# Supplementary material for: Optimal human papillomavirus vaccination strategies to prevent cervical cancer in low-income and middle-income countries in the context of limited resources: a mathematical modelling analysis
Source: Lancet Infect Dis. 2021 Nov;21(11):1598–610. doi: 10.1016/S1473-3099(20)30860-4 (PMC8554391; doi:10.1016/S1473-3099(20)30860-4)
Supplement: Supplementary appendix 4 [file mmc4.pdf]

THE LANCET
Infectious Diseases

Supplementary appendix 4

This appendix formed part of the original submission and has been peer reviewed. We post it as supplied by the authors.

Supplement to: Drolet M, Laprise J-F, Martin D, et al. Optimal human papillomavirus vaccination strategies to prevent cervical cancer in low-income and middle-income countries in the context of limited resources: a mathematical modelling analysis. *Lancet Infect Dis* 2021; published online July 7. https://doi.org/10.1016/S1473-3099(20)30860-4.

Supplementary appendix

- Table S1. HPV vaccination strategies ... 2
- Table S2. Economic parameters ... 4
- Table S3. Number of years following HPV vaccination to reach a 50% decline in age-standardized cervical cancer incidence (vs no vaccination) ... 5
- Table S4. Relative reduction at equilibrium in age-standardized incidence of cervical cancer and prevalence of HPV16/18 infection for the different HPV vaccination strategies (vs no vaccination) ... 6
- Table S5. Incremental efficiency and cost-effectiveness of different 2-dose HPV vaccination strategies varying the number of cohorts, age, and population (girls-only or girls & boys) targeted ... 8
- Table S6. Efficiency and cost-effectiveness of 2-dose HPV vaccination strategies to optimize the vaccination of girls aged between 9 and 14 years ... 10
- Table S7. Incremental efficiency and cost-effectiveness of different 1-dose HPV vaccination strategies varying the number of cohorts, age, and population (girls-only or girls & boys) targeted, assuming 1-dose duration of protection of 20 years ... 12
- Table S8. Incremental efficiency and cost-effectiveness of different 1-dose HPV vaccination strategies varying the number of cohorts, age, and population (girls-only or girls & boys) targeted, assuming 1-dose efficacy of 85% ... 14
- Table S9. Number needed to vaccinate (doses) to prevent 1 cervical cancer (vs no vaccination, undiscounted) ... 17
- Table S10. Number needed to vaccinate (vaccinees) to prevent 1 cervical cancer (vs no vaccination, undiscounted) ... 18
- Table S11. Cost-effectiveness ($/DALY-averted) of the different HPV vaccination strategies (vs no vaccination) ... 19
- Table S12. Sensitivity analysis: vaccine used ... 21
- Table S13. Advantages of the different HPV vaccination strategies of girls aged between 9 to 14 years old ... 23

- Figure S1. Framework used to examine optimal HPV vaccination strategies: Policy questions, goals, analyses and outcomes ... 24
- Figure S2. Description of the different HPV vaccination strategies to optimize the vaccination of girls aged 9 to 14 years old ... 25
- Figure S3. Examples of model fit to country-specific epidemiological data: Fit to proportion of sexually active women ... 27
- Figure S4. Examples of model fit to country-specific epidemiological data: Fit to HPV16/18 prevalence among sexually active females ... 28
- Figure S5. Examples of model fit to country-specific epidemiological data: Fit to high-risk HPV prevalence among sexually active females ... 29
- Figure S6. Examples of model fit to country-specific epidemiological data: Fit to age-specific cervical cancer incidence ... 30
- Figure S7. Examples of model fit to country-specific epidemiological data: Fit to proportion of cervical cancers caused by high risk HPV types (HPV16; HPV16/18; cross-protective types HPV31/33/45/52/58; and non-cross-protective types HPV35/39/51/56/59/66/68/73/82) ... 31
- Figure S8. Impact of 1 dose duration of protection and vaccine efficacy on the population-level impact of routine vaccination of girls aged 9 years with multi-cohort vaccination (MAC) ... 32
- Figure S9. Incremental efficiency and cost-effectiveness of different 1-dose HPV vaccination strategies varying the number of cohorts, age, and population (girls-only or girls & boys) targeted ... 34

- CHEERS checklist ... 38

Table S1. HPV vaccination strategies

A) 2 doses: Variation of the number of cohorts, age and population (Girls-only or Girls & Boys) targeted

	Routine vaccination		Additional cohorts targeted			Dose interval	
Strategies	9 yrs old	14 yrs old	MAC 9-14 yrs old	MAC 9-18 yrs old	MAC 9-25 yrs old	Current*	Extended¶
**Girls-only**							
1. Routine 9 yrs old	x					x	
2a. Routine 9 yrs old & MAC 9-14 yrs old	x		x (First yr of program)			x	
3a. Routine 9 yrs old & MAC 9-18 yrs old	x			x (First yr of program)		x	
4a. Routine 9 yrs old & MAC 9-25 yrs old	x				x (First yr of program)	x	
**Girls & Boys**							
2b. Routine 9 yrs old & MAC 9-14 yrs old ¥	x		x (First yr of program)			x	
3b. Routine 9 yrs old & MAC 9-18 yrs old ¥	x			x (First yr of program)		x	
4b. Routine 9 yrs old & MAC 9-25 yrs old ¥	x				x (First yr of program)	x	

B) 2 doses: Optimization of vaccination of girls aged 9 to 14 years old

	Routine vaccination		Additional cohorts targeted		Dose interval	
Girls-only Strategies	9 yrs old	14 yrs old	MAC 9-14 yrs old	Catch-up 14 yrs old	Current[1]	Extended[2]
1. Routine 9 yrs old	x				x	
2. Routine 9 yrs old 5-yr extended interval	x					x
3. Routine 14 yrs old		x			x	
4. Routine 9 yrs old & MAC 9-14 yrs old	x		x (First yr of program)		x	
5. Routine 9 yrs old & Catch-up 14 yrs old	x			x (First 5 yrs of program)	x	
6. Routine 9 yrs old 5-yr extended interval & 14-yr-old catch-up	x			x (First 5 yrs of program)		x
7. Routine 14 yrs old & later switch to Routine 9 yrs old	x (After 5 years)	x (First 10 yrs of program)			x	

C) 1 dose: Variation of the number of cohorts, age and population (Girls-only or Girls & Boys) targeted

	Routine vaccination		Additional cohorts targeted		
Strategies	9 yrs old	14 yrs old	MAC 9-14 yrs old	MAC 9-18 yrs old	MAC 9-25 yrs old
**Girls-only**					
2c. Routine 9 yrs old & MAC 9-14 yrs old	x		x (First yr of program)		
3c. Routine 9 yrs old & MAC 9-18 yrs old	x			x (First yr of program)	
4c. Routine 9 yrs old & MAC 9-25 yrs old	x				x (First yr of program)
**Girls & Boys**					
2d. Routine 9 yrs old & MAC 9-14 yrs old ¥	x		x (First yr of program)		
3d. Routine 9 yrs old & MAC 9-18 yrs old ¥	x			x (First yr of program)	
4d. Routine 9 yrs old & MAC 9-25 yrs old ¥	x				x (First yr of program)

MAC: Multiple age cohort vaccination

* Currently recommended 2-dose schedule

¶ First dose at 9 years old, second dose at 14 years old

¥ Girls & boys MAC: Girls age as indicated and boys always ≤ 14 yrs

Table S2. Economic parameters

	India	Vietnam	Uganda	Nigeria
**Cost-effectiveness thresholds** (i$2017)*				
GDP per capita	7,056	6,776	1,864	5,861
0.5 x GDP per capita	3,528	3,388	932	2,930
**Costs (i$2017)**				
HPV vaccine cost per dose				
2/4-valent (base case; max GAVI)	4.60	4.60	4.60	4.60
9-valent				
- Equal price assumption	4.60	4.60	4.60	4.60
- 1.5 x 2/4-valent GAVI price assumption	7.50	7.50	7.50	7.50
Vaccine delivery cost per dose£	7.50	7.50	2.50	7.50
CC treatment (unadjusted for GDP)£,¶	687.94	1066.08	899.10	737.14
**DALYs£**				
Health outcomes				
All CC cases (diagnosis/treatment)	0.08	0.08	0.08	0.08
Non-fatal CC cases (sequelae)	0.68	0.44	0.68	0.68
Fatal CC cases (final phases)	0.78	0.78	0.78	0.78
Death	Remaining LE#	Remaining LE#	Remaining LE#	Remaining LE#
**Mortality**				
Probability of death from CC (%)‡	55.0	46.9	58.2	58.5

GDP=Gross Domestic Product; i$=international dollars; 2/4-valent=bivalent or quadrivalent HPV vaccines; 9-valent=nonavalent HPV vaccine; GAVI=Global Alliance for Vaccines and Immunization; USD=United States Dollars; DALY= Disability-Adjusted Life-Year; CC=Cervical Cancer; LE=Life Expectancy;

* We use 0.5x and 1x GDP per capita as cost-effectiveness thresholds. Data from World Bank, September 2018 (https://data.worldbank.org/)

£ Data from PRIME (http://primetool.org) provided by Mark Jit, London School of Hygiene & Tropical Medicine, UK

¶ Inflated from i$2011 to i$2017 using World Bank data, September 2018 (https://data.worldbank.org/)

§ Personal communication, September 18, 2018, Raymond Hutubessy, World Health Organization

Remaining life expectancy is country-, gender- and age-specific

‡ Data from GLOBOCAN 2012: [Probability of death from CC] = [Crude death rate] / [Crude CC incidence]

Table S3. Number of years following HPV vaccination to reach a 50% decline in age-standardized cervical cancer incidence (vs no vaccination)

Vaccination Strategies[α]	India<br>Mean nb of years (80% UI)	Vietnam<br>Mean nb of years (80% UI)	Uganda<br>Mean nb of years (80% UI)	Nigeria<br>Mean nb of years (80% UI)
**2 doses: Variation of the number of cohorts, age, and population targeted**				
**2 doses: Girls-only Routine vaccination, No MAC**				
1. Routine 9 yrs old	48 (45-48*)	47 (47-47)	49 (47-49*)	53 (50-53*)
**2 doses: Girls-only Routine vaccination with MAC**				
2a. Routine 9 yrs old with MAC 9-14 yrs old	44 (41-44*)	43 (42-43)	45 (43-45*)	49 (47-49*)
3a. Routine 9 yrs old with MAC 9-18 yrs old[γ]	42 (39-42*)	38 (39-39)	41 (40-41)	46 (45-46*)
4a. Routine 9 yrs old with MAC 9-25 yrs old[γ]	39 (37-39)	37 (36-37)	40 (38-40*)	45 (42-45)
**2 doses: Girls&Boys Routine vaccination with MAC[†]**				
2b. Routine 9 yrs old with MAC 9-14 yrs old	42 (40-42*)	39 (38-39)	42 (40-42*)	47 (45-47*)
3b. Routine 9 yrs old with MAC 9-18 yrs old[γ]	39 (38-39)	37 (36-37)	40 (38-40*)	45 (42-45*)
4b. Routine 9 yrs old with MAC 9-25 yrs old[γ]	38 (36-38*)	34 (34-35)	38 (37-38*)	43 (41-43*)
**2 doses: Girls-only optimisation of 9-14 years-old vaccination**				
1. Routine 9 yrs old, No MAC	48 (45-48*)	47 (47-47)	49 (47-49*)	53 (50-53*)
2. Routine 9 yrs old, 5-yr extended interval[¥], No MAC	48 (45-48*)	47 (47-47)	49 (47-49*)	53 (50-53*)
3. Routine 14 yrs old, No MAC	44 (43-45)	43 (42-43)	45 (43-45*)	50 (47-50)
4. Routine 9 yrs old with MAC 9-14 yrs old	44 (41-44*)	43 (42-43)	45 (43-45*)	49 (47-49*)
5. Routine 9 yrs old with 14-yr-old catch-up	44 (43-44*)	43 (42-43)	46 (43-46*)	49 (46-49*)
6. Routine 9 yrs old, 5-yr extended interval[¥] with 14-yr-old catch-up	44 (43-44*)	43 (42-43)	46 (43-46*)	49 (46-49*)
7. Routine 14 yrs old & later switch to Routine 9 yrs old	43 (41-44)	43 (42-43*)	45 (42-45*)	49 (46-49*)
**1 dose: Girls-only Routine vaccination with MAC, Vaccine duration = 20 years**				
2c. Routine 9 yrs old with MAC 9-14 yrs old	67 (53-X)	X (X-X)	X (87-X)	60 (55-X)
3c. Routine 9 yrs old with MAC 9-18 yrs old[γ]	52 (45-X)	X (X-X)	X (56-X)	52 (49-X)
4c. Routine 9 yrs old with MAC 9-25 yrs old[γ]	45 (43-X)	X (X-X)	54 (44-54*)	50 (45-X)
**1 dose: Girls&Boys Routine vaccination with MAC[†], Vaccine duration = 20 years**				
2d. Routine 9 yrs old with MAC 9-14 yrs old	47 (43-47)	48 (48-X)	53 (44-53*)	53 (47-54)
3d. Routine 9 yrs old with MAC 9-18 yrs old[γ]	43 (41-43*)	39 (39-46)	44 (41-44*)	49 (45-50)
4d. Routine 9 yrs old with MAC 9-25 yrs old[γ]	39 (39-39)	36 (36-39)	41 (38-41)	46 (43-46)
**1 dose: Girls-only Routine vaccination with MAC, Vaccine efficacy = 85%**				
2c. Routine 9 yrs old with MAC 9-14 yrs old	47 (44-47)	45 (44-47)	50 (47-50*)	53 (49-53)
3c. Routine 9 yrs old with MAC 9-18 yrs old[γ]	44 (43-44)	43 (40-43)	46 (44-46*)	49 (47-50)
4c. Routine 9 yrs old with MAC 9-25 yrs old[γ]	42 (39-42*)	39 (39-40)	43 (40-44)	48 (45-48)
**1 dose: Girls&Boys Routine vaccination with MAC[†], Vaccine efficacy = 85%**				
2d. Routine 9 yrs old with MAC 9-14 yrs old	44 (41-44*)	40 (40-41)	44 (41-44*)	49 (46-49*)
3d. Routine 9 yrs old with MAC 9-18 yrs old[γ]	41 (39-41*)	38 (38-38)	41 (40-41*)	46 (44-46*)
4d. Routine 9 yrs old with MAC 9-25 yrs old[γ]	39 (37-39*)	36 (34-36)	40 (38-40*)	45 (42-45*)

MAC: Multiple age cohort vaccination

Predictions: Mean of the 10 best fit parameter sets to Globocan 2020 (80% UI: 10th-90th percentiles of the 50 parameter sets); Base case: 2 doses, Vaccine efficacy against HPV16/18/31/33/45/52/58 =100%, Vaccine duration=Lifetime, Vaccination coverage=80%

[α] Vaccination strategies are described in Table S1

* Maximum value is the mean of the 10 best fit parameter sets to Globocan 2020

[γ] Girls >14 years old always receive 3 doses

[¥] Extended interval: 1st dose at 9 years old and 2nd dose at 14 years old

[†] Girls&Boys MAC: Girls age as indicated and boys always ≤ 14 yrs old

Table S4. Relative reduction at equilibrium in the age-standardized incidence of cervical cancer and prevalence of HPV16/18 infection for the different HPV vaccination strategies (vs no vaccination)

Vaccination Strategies [α] ---- vs no vaccination	India			Vietnam			Uganda			Nigeria		
	Females		Males	Females		Males	Females		Males	Females		Males
	Cervical cancer Mean	HPV 16/18 Mean	HPV 16/18 Mean	Cervical cancer Mean	HPV 16/18 Mean	HPV 16/18 Mean	Cervical cancer Mean	HPV 16/18 Mean	HPV 16/18 Mean	Cervical cancer Mean	HPV 16/18 Mean	HPV 16/18 Mean
**2 doses: Variation of the number of cohorts, age, and population targeted**												
**2 doses: Girls-only Routine vaccination, No MAC**												
1. Routine 9 yrs old	85%	86%	76%	84%	82%	59%	79%	84%	54%	79%	83%	50%
**2 doses: Girls-only Routine vaccination with MAC**												
2a. Routine 9 yrs old with MAC 9-14 yrs old	86%	87%	76%	85%	83%	59%	79%	84%	53%	79%	83%	50%
3a. Routine 9 yrs old with MAC 9-18 yrs old [γ]	86%	87%	77%	85%	83%	61%	79%	84%	54%	80%	83%	50%
4a. Routine 9 yrs old with MAC 9-25 yrs old [γ]	85%	87%	76%	84%	83%	60%	80%	84%	53%	79%	83%	50%
**2 doses: Girls&Boys Routine vaccination with MAC[†]**												
2b. Routine 9 yrs old with MAC 9-14 yrs old	95%	96%	96%	96%	96%	92%	91%	96%	91%	90%	96%	90%
3b. Routine 9 yrs old with MAC 9-18 yrs old [γ]	95%	97%	96%	96%	96%	92%	91%	96%	91%	90%	96%	90%
4b. Routine 9 yrs old with MAC 9-25 yrs old [γ]	95%	97%	96%	96%	96%	93%	91%	96%	91%	91%	95%	90%
**2 doses: Girls-only optimisation of 9-14 years-old vaccination**												
1. Routine 9 yrs old, No MAC	85%	86%	76%	84%	82%	59%	79%	84%	54%	79%	83%	50%
2. Routine 9 yrs old, 5-yr extended interval[¥], No MAC	85%	86%	76%	84%	82%	59%	79%	84%	54%	79%	83%	50%
3. Routine 14 yrs old, No MAC	81%	83%	70%	84%	82%	58%	78%	83%	51%	76%	80%	44%
4. Routine 9 yrs old with MAC 9-14 yrs old	86%	87%	76%	85%	83%	59%	79%	84%	53%	79%	83%	50%
5. Routine 9 yrs old with 14-yr-old catch-up	85%	87%	76%	84%	83%	60%	79%	84%	54%	79%	83%	50%
6. Routine 9 yrs old, 5-yr extended interval[¥] with 14-yr-old catch-up	85%	87%	76%	84%	83%	60%	79%	84%	54%	79%	83%	50%
7. Routine 14 yrs old & later switch to Routine 9 yrs old	85%	86%	76%	85%	83%	60%	79%	84%	53%	79%	83%	51%
**1 dose: Girls-only Routine vaccination with MAC, Vaccine duration = 20 years**												
2c. Routine 9 yrs old with MAC 9-14 yrs old	50%	42%	32%	33%	21%	8%	45%	40%	22%	53%	47%	21%
3c. Routine 9 yrs old with MAC 9-18 yrs old [γ]	50%	42%	32%	33%	21%	9%	46%	40%	21%	54%	47%	22%
4c. Routine 9 yrs old with MAC 9-25 yrs old [γ]	48%	42%	32%	31%	19%	5%	46%	40%	22%	53%	47%	22%

Vaccination Strategies [α] vs no vaccination	India			Vietnam			Uganda			Nigeria		
	Females		Males	Females		Males	Females		Males	Females		Males
	Cervical cancer	HPV 16/18	HPV 16/18	Cervical cancer	HPV 16/18	HPV 16/18	Cervical cancer	HPV 16/18	HPV 16/18	Cervical cancer	HPV 16/18	HPV 16/18
	Mean	Mean	Mean	Mean	Mean	Mean	Mean	Mean	Mean	Mean	Mean	Mean
**1 dose: Girls&Boys Routine vaccination with MAC[†], Vaccine duration = 20 years**												
2d. Routine 9 yrs old with MAC 9-14 yrs old	60%	52%	51%	49%	32%	35%	55%	49%	46%	62%	55%	45%
3d. Routine 9 yrs old with MAC 9-18 yrs old [γ]	60%	52%	51%	49%	33%	36%	56%	49%	46%	62%	54%	45%
4d. Routine 9 yrs old with MAC 9-25 yrs old [γ]	61%	52%	51%	47%	30%	34%	56%	49%	46%	61%	54%	45%
**1 dose: Girls-only Routine vaccination with MAC, Vaccine efficacy = 85%**												
2c. Routine 9 yrs old with MAC 9-14 yrs old	74%	75%	62%	73%	70%	45%	68%	72%	41%	68%	70%	37%
3c. Routine 9 yrs old with MAC 9-18 yrs old [γ]	73%	74%	62%	72%	71%	45%	68%	72%	41%	67%	70%	37%
4c. Routine 9 yrs old with MAC 9-25 yrs old [γ]	74%	74%	62%	72%	70%	46%	68%	72%	41%	68%	70%	38%
**1 dose: Girls&Boys Routine vaccination with MAC[†], Vaccine efficacy = 85%**												
2d. Routine 9 yrs old with MAC 9-14 yrs old	89%	90%	88%	90%	89%	81%	85%	90%	81%	84%	89%	79%
3d. Routine 9 yrs old with MAC 9-18 yrs old [γ]	89%	90%	88%	90%	89%	81%	85%	90%	81%	84%	89%	79%
4d. Routine 9 yrs old with MAC 9-25 yrs old [γ]	89%	90%	88%	89%	89%	81%	85%	90%	81%	84%	89%	79%

MAC: Multiple age cohort vaccination

Predictions: Mean of the 10 best fit parameter sets to Globocan 2020

Base case: 2 doses, Vaccine efficacy against HPV16/18/31/33/45/52/58 =100%, Vaccine duration=Lifetime, Vaccination coverage=80%

[α] Vaccination strategies are described in Table S1

[γ] Girls >14 years old always receive 3 doses

[¥] Extended interval: First dose at 9 years old and second dose at 14 years old

[†] Girls&Boys MAC: Girls age as indicated and boys always ≤ 14 yrs old

Table S5. Incremental efficiency and cost-effectiveness of 2-dose HPV vaccination strategies varying the number of cohorts, age, and population targeted (girls-only or girls & boys)

		Efficiency (undiscounted)			Cost-effectiveness (discounted)		
		Number of cases averted (thousands)	Additional number of doses (millions)	NNV	DALYs averted (thousands)	Additional cost (millions)	ICER
Vaccination strategies [α]	Comparator for NNV and ICER	Mean (80%UI)	Mean (80%UI)	Mean (80%UI)	Mean (80%UI)	Mean (80%UI)	Mean (80%UI)
**INDIA**							
**Vaccine cost per dose[¶]: $4.60**							
1. Girls Routine 9 yrs	No vaccination	6,069 (6,299-10,779)	1,365 (1,365-1,369)	225 (127-225*)	8,041 (8,466-15,703)	4,732 (4,277-4,732*)	Extended dominated
2a. Girls Routine 9 yrs & MAC 9-14 yrs	No vaccination	7,223 (7,572-12,533)	1,419 (1,418-1,423)	196 (113-196*)	11,203 (11,083-21,243)	5,163 (4,589-5,163*)	461 (219-465)
2b. Girls&Boys Routine 9 yrs & MAC 9-14 yrs	vs. 2a	765 (739-1,613)	1,419 (1,418-1,423)	1,855 (881-1,920)	1,051 (467-3,177)	5,823 (5,703-5,859)	Extended dominated
3a. Girls Routine 9 yrs & MAC 9-18 yrs	vs. 2a	287 (235-693)	80 (80-80)	279 (115-339)	884 (453-2,584)	894 (784-939)	1,011 (303-2,100)
3b. Girls&Boys Routine 9 yrs & MAC 9-18 yrs [γ]	vs. 3a	770 (609-1,368)	1,419 (1,418-1,423)	1,843 (1,040-2,333)	1,106 (592-2,121)	5,819 (5,765-5,859)	Extended dominated
4a. Girls Routine 9 yrs & MAC 9-25 yrs	vs. 3a	192 (138-475)	139 (139-139)	724 (290-962)	548 (174-1,694)	1,622 (1,545-1,643)	2,962 (914-9,682)
4b. Girls&Boys Routine 9 yrs & MAC 9-25 yrs [γ]	vs. 4a	738 (567-1,513)	1,419 (1,418-1,423)	1,923 (940-2,507)	916 (501-2,508)	5,835 (5,742-5,866)	6,371 (2,292-X)
**Vaccine cost per dose[¶]: $7.50**							
1. Girls Routine 9 yrs	No vaccination	6,069 (6,299-10,779)	1,365 (1,365-1,369)	225 (127-225*)	8,041 (8,466-15,703)	5,990 (5,539-5,990*)	Extended dominated
2a. Girls Routine 9 yrs & MAC 9-14 yrs	No vaccination	7,223 (7,572-12,533)	1,419 (1,418-1,423)	196 (113-196*)	11,203 (11,083-21,243)	6,573 (6,000-6,573*)	587 (286-593)
2b. Girls&Boys Routine 9 yrs & MAC 9-14 yrs	vs. 2a	765 (739-1,613)	1,419 (1,418-1,423)	1,855 (881-1,920)	1,051 (467-3,177)	7,234 (7,118-7,270)	Extended dominated
3a. Girls Routine 9 yrs & MAC 9-18 yrs	vs. 2a	287 (235-693)	80 (80-80)	279 (115-339)	884 (453-2,584)	1,122 (1,012-1,167)	1,269 (392-2,611)
3b. Girls&Boys Routine 9 yrs & MAC 9-18 yrs [γ]	vs. 3a	770 (609-1,368)	1,419 (1,418-1,423)	1,843 (1,040-2,333)	1,106 (592-2,121)	7,231 (7,177-7,273)	Extended dominated
4a. Girls Routine 9 yrs & MAC 9-25 yrs	vs. 3a	192 (138-475)	139 (139-139)	724 (290-962)	548 (174-1,694)	2,020 (1,943-2,041)	3,689 (1,149-12,029)
4b. Girls&Boys Routine 9 yrs & MAC 9-25 yrs [γ]	vs. 4a	738 (567-1,513)	1,419 (1,418-1,423)	1,923 (940-2,507)	916 (501-2,508)	7,246 (7,152-7,279)	7,912 (2,857-X)
**VIETNAM**							
**Vaccine cost per dose[¶]: $4.60**							
1. Girls Routine 9 yrs	No vaccination	254 (265-716)	97 (97-97)	381 (135-381*)	307 (336-1,031)	342 (268-342*)	Extended dominated
2a. Girls Routine 9 yrs & MAC 9-14 yrs	No vaccination	279 (298-763)	101 (101-101)	361 (132-361*)	383 (433-1,145)	381 (304-381*)	996 (266-996*) [§]
2b. Girls&Boys Routine 9 yrs & MAC 9-14 yrs	vs. 2a	49 (47-129)	101 (100-101)	2,034 (779-2,145)	89 (43-251)	410 (393-416)	Extended dominated
3a. Girls Routine 9 yrs & MAC 9-18 yrs	vs. 2a	25 (12-68)	6 (6-6)	235 (85-288)	76 (3-229)	62 (44-70)	809 (191-X)
3b. Girls&Boys Routine 9 yrs & MAC 9-18 yrs [γ]	vs. 3a	41 (41-109)	101 (100-101)	2,479 (921-2,479*)	61 (33-202)	413 (398-418)	Extended dominated
4a. Girls Routine 9 yrs & MAC 9-25 yrs	vs. 3a	15 (10-41)	10 (10-10)	660 (250-898)	50 (6-144)	116 (106-121)	2,326 (731-20,128)
4b. Girls&Boys Routine 9 yrs & MAC 9-25 yrs [γ]	vs. 4a	38 (41-119)	101 (100-101)	2,672 (846-2,672*)	49 (29-184)	414 (400-416)	8,373 (2,179-15,561)
**Vaccine cost per dose[¶]: $7.50**							
1. Girls Routine 9 yrs	No vaccination	254 (265-716)	97 (97-97)	381 (135-381*)	307 (336-1,031)	432 (358-432*)	Extended dominated
2a. Girls Routine 9 yrs & MAC 9-14 yrs	No vaccination	279 (298-763)	101 (101-101)	361 (132-361*)	383 (433-1,145)	482 (405-482*)	1,259 (354 - 1,259*) [§]
2b. Girls&Boys Routine 9 yrs & MAC 9-14 yrs	vs. 2a	49 (47-129)	101 (100-101)	2,034 (779-2,145)	89 (43-251)	510 (493-516)	Extended dominated
3a. Girls Routine 9 yrs & MAC 9-18 yrs	vs. 2a	25 (12-68)	6 (6-6)	235 (85-288)	76 (3-229)	78 (60-86)	1,029 (264-X)
3b. Girls&Boys Routine 9 yrs & MAC 9-18 yrs [γ]	vs. 3a	41 (41-109)	101 (100-101)	2,479 (921-2,479*)	61 (33-202)	513 (499-519)	Extended dominated
4a. Girls Routine 9 yrs & MAC 9-25 yrs	vs. 3a	15 (10-41)	10 (10-10)	660 (250-898)	50 (6-144)	145 (135-150)	2,910 (932-24,961)
4b. Girls&Boys Routine 9 yrs & MAC 9-25 yrs [γ]	vs. 4a	38 (41-119)	101 (100-101)	2,672 (846-2,672*)	49 (29-184)	514 (500-517)	10,404 (2,727-19,320)
**UGANDA**							
**Vaccine cost per dose[¶]: $4.60**							
1. Girls Routine 9 yrs	No vaccination	460 (360-585)	38 (38-38)	83 (65-105)	694 (503-984)	30 (9-44)	Extended dominated
2a. Girls Routine 9 yrs & MAC 9-14 yrs	No vaccination	494 (386-628)	39 (39-39)	80 (63-102)	795 (620-1,110)	32 (10-44)	40 (8-71)

Vaccination strategies [α]	Comparator for NNV and ICER	Efficiency (undiscounted): Number of cases averted (thousands) Mean (80%UI)	Efficiency (undiscounted): Additional number of doses (millions) Mean (80%UI)	Efficiency (undiscounted): NNV Mean (80%UI)	Cost-effectiveness (discounted): DALYs averted (thousands) Mean (80%UI)	Cost-effectiveness (discounted): Additional cost (millions) Mean (80%UI)	Cost-effectiveness (discounted): ICER Mean (80%UI)
2b. Girls&Boys Routine 9 yrs & MAC 9-14 yrs	vs. 2a	70 (52-97)	39 (39-39)	565 (408-754)	117 (59-194)	87 (80-91)	Extended dominated
3a. Girls Routine 9 yrs & MAC 9-18 yrs	vs. 2a	26 (16-36)	2 (2-2)	86 (62-135)	84 (47-116)	9 (6-12)	104 (48-247)
3b. Girls&Boys Routine 9 yrs & MAC 9-18 yrs [γ]	vs. 3a	67 (50-89)	39 (39-39)	588 (442-781)	108 (62-155)	87 (84-91)	Extended dominated
4a. Girls Routine 9 yrs & MAC 9-25 yrs	vs. 3a	20 (7-25)	4 (4-4)	198 (158-539)	67 (16-90)	21 (19-26)	316 (210-1,584)
4b. Girls&Boys Routine 9 yrs & MAC 9-25 yrs [γ]	vs. 4a	64 (50-89)	39 (39-39)	619 (442-783)	93 (52-157)	89 (84-92)	954 (535-1,789)
**Vaccine cost per dose[¶]: $7.50**							
1. Girls Routine 9 yrs	No vaccination	460 (360-585)	38 (38-38)	83 (65-105)	694 (503-984)	65 (44-79)	Extended dominated
2a. Girls Routine 9 yrs & MAC 9-14 yrs	No vaccination	494 (386-628)	39 (39-39)	80 (63-102)	795 (620-1,110)	71 (49-83)	89 (43-134)
2b. Girls&Boys Routine 9 yrs & MAC 9-14 yrs	vs. 2a	70 (52-97)	39 (39-39)	565 (408-754)	117 (59-194)	126 (119-131)	Extended dominated
3a. Girls Routine 9 yrs & MAC 9-18 yrs	vs. 2a	26 (16-36)	2 (2-2)	86 (62-135)	84 (47-116)	15 (12-18)	181 (103-384)
3b. Girls&Boys Routine 9 yrs & MAC 9-18 yrs [γ]	vs. 3a	67 (50-89)	39 (39-39)	588 (442-781)	108 (62-155)	127 (123-130)	Extended dominated
4a. Girls Routine 9 yrs & MAC 9-25 yrs	vs. 3a	20 (7-25)	4 (4-4)	198 (158-539)	67 (16-90)	32 (30-37)	483 (333-2,261)
4b. Girls&Boys Routine 9 yrs & MAC 9-25 yrs [γ]	vs. 4a	64 (50-89)	39 (39-39)	619 (442-783)	93 (52-157)	128 (123-131)	1,376 (786-2,550)
**NIGERIA**							
**Vaccine cost per dose[¶]: $4.60**							
1. Girls Routine 9 yrs	No vaccination	1,254 (1,063-1,921)	209 (208-209)	166 (109-196)	1,523 (1,354-2,826)	684 (603-697)	Extended dominated
2a. Girls Routine 9 yrs & MAC 9-14 yrs	No vaccination	1,358 (1,162-2,045)	217 (217-218)	160 (106-187)	1,780 (1,477-3,215)	763 (673-789)	429 (207-534)
2b. Girls&Boys Routine 9 yrs & MAC 9-14 yrs	vs. 2a	170 (138-324)	217 (217-218)	1,279 (672-1,580)	207 (166-503)	887 (866-891)	Extended dominated
3a. Girls Routine 9 yrs & MAC 9-18 yrs	vs. 2a	67 (41-101)	12 (12-12)	185 (123-300)	177 (115-324)	133 (122-140)	752 (376-1,214)
3b. Girls&Boys Routine 9 yrs & MAC 9-18 yrs [γ]	vs. 3a	171 (152-314)	217 (217-218)	1,268 (691-1,421)	223 (169-502)	886 (866-891)	Extended dominated
4a. Girls Routine 9 yrs & MAC 9-25 yrs	vs. 3a	32 (13-71)	21 (21-21)	674 (302-1,624)	93 (6-234)	246 (233-252)	2,649 (995-46,733)
4b. Girls&Boys Routine 9 yrs & MAC 9-25 yrs [γ]	vs. 4a	168 (150-304)	217 (217-218)	1,294 (716-1,450)	178 (150-485)	889 (869-891)	4,998 (1,793-5,917)
**Vaccine cost per dose[¶]: $7.50**							
1. Girls Routine 9 yrs	No vaccination	1,254 (1,063-1,921)	209 (208-209)	166 (109-196)	1,523 (1,354-2,826)	876 (796-890)	Extended dominated
2a. Girls Routine 9 yrs & MAC 9-14 yrs	No vaccination	1,358 (1,162-2,045)	217 (217-218)	160 (106-187)	1,780 (1,477-3,215)	979 (890-1,005)	550 (274-680)
2b. Girls&Boys Routine 9 yrs & MAC 9-14 yrs	vs. 2a	170 (138-324)	217 (217-218)	1,279 (672-1,580)	207 (166-503)	1,103 (1,083-1,107)	Extended dominated
3a. Girls Routine 9 yrs & MAC 9-18 yrs	vs. 2a	67 (41-101)	12 (12-12)	185 (123-300)	177 (115-324)	168 (158-176)	953 (486-1,524)
3b. Girls&Boys Routine 9 yrs & MAC 9-18 yrs [γ]	vs. 3a	171 (152-314)	217 (217-218)	1,268 (691-1,421)	223 (169-502)	1,102 (1,082-1,107)	Extended dominated
4a. Girls Routine 9 yrs & MAC 9-25 yrs	vs. 3a	32 (13-71)	21 (21-21)	674 (302-1,624)	93 (6-234)	306 (293-313)	3,304 (1,254-57,939)
4b. Girls&Boys Routine 9 yrs & MAC 9-25 yrs [γ]	vs. 4a	168 (150-304)	217 (217-218)	1,294 (716-1,450)	178 (150-485)	1,105 (1,085-1,107)	6,215 (2,240-7,356)

MAC: Multiple age cohort vaccination ; DALY: Disability-adjusted life-years ; ICER: Incremental cost-effectiveness ratio ; UI: Uncertainty interval ; NNV: Number of doses needed to prevent 1 case of cervical cancer; X: >1million $/daly

Predictions: Mean of the 10 best fit parameter sets to Globocan 2020 (80% UI: 10th-90th percentiles of the 50 parameter sets)

Base case: 2 doses, Vaccine efficacy against HPV16/18/31/33/45/52/58 =100%, Vaccine duration=Lifetime, Vaccination coverage=80%

[α] Vaccination strategies are described in Table S1

[¶] Vaccine cost per dose including administration costs

[γ] Girls & boys MAC: Girls age as indicated and boys always ≤ 14 yrs

[*] Maximum value is the mean of the 10 best fit parameter sets to Globocan 2020

[§] 26 / 50 parameter sets were extended dominated for this strategy

Table S6. Efficiency and cost-effectiveness of 2-dose HPV vaccination strategies to optimize the vaccination of girls aged between 9 and 14 years

Vaccination strategies [α]	Comparator for NNV and ICER	Efficiency (undiscounted): Number of cases averted (thousands) Mean (80%UI)	Efficiency (undiscounted): Additional number of doses (millions) Mean (80%UI)	Efficiency (undiscounted): NNV Mean (80%UI)	Cost-effectiveness (discounted): DALYs averted (thousands) Mean (80%UI)	Cost-effectiveness (discounted): Additional cost (millions) Mean (80%UI)	Cost-effectiveness (discounted): ICER Mean (80%UI)
**INDIA**							
**Vaccine cost per dose[¶]: $4.60**							
1. Girls Routine 9 yrs	No vaccination	6,069 (6,299-10,779)	1,365 (1,365-1,369)	225 (127-225*)	8,041 (8,466-15,703)	4,732 (4,277-4,732*)	589 (274-589*)
2. Girls Routine 9 yrs 5-yr extended interval [γ]	No vaccination	6,069 (6,299-10,779)	1,339 (1,339-1,342)	221 (125-221*)	8,041 (8,466-15,703)	4,428 (3,971-4,428*)	551 (254-551*)
3. Girls Routine 14 yrs	No vaccination	6,741 (6,979-12,045)	1,365 (1,365-1,369)	203 (114-203*)	10,259 (10,569-19,477)	4,588 (4,040-4,588*)	447 (208-447*)
4. Girls Routine 9 yrs & MAC 9-14 yrs	No vaccination	7,223 (7,572-12,533)	1,419 (1,418-1,423)	196 (113-196*)	11,203 (11,083-21,243)	5,163 (4,589-5,163*)	461 (219-465)
5. Girls Routine 9 yrs & 14-yr-old catch-up[¥]	No vaccination	7,154 (7,424-12,528)	1,419 (1,419-1,423)	198 (113-198*)	10,981 (11,310-21,033)	5,152 (4,615-5,152*)	469 (220-469*)
6. Girls Routine 9 yrs 5-yr extended interval & 14-yr-old catch-up[α]	No vaccination	7,154 (7,424-12,528)	1,392 (1,392-1,396)	195 (111-195*)	10,981 (11,310-21,033)	4,848 (4,310-4,848*)	441 (206-441*)
7. Girls Routine 14 yrs & later switch to Routine 9 yrs	No vaccination	7,060 (7,307-12,279)	1,419 (1,419-1,423)	201 (116-201*)	10,645 (11,517-20,991)	5,092 (4,531-5,092*)	478 (217-478*)
**Vaccine cost per dose[¶]: $7.50**							
1. Girls Routine 9 yrs	No vaccination	6,069 (6,299-10,779)	1,365 (1,365-1,369)	225 (127-225*)	8,041 (8,466-15,703)	5,990 (5,539-5,990*)	745 (354-745*)
2. Girls Routine 9 yrs 5-yr extended interval [γ]	No vaccination	6,069 (6,299-10,779)	1,339 (1,339-1,342)	221 (125-221*)	8,041 (8,466-15,703)	5,613 (5,161-5,613*)	698 (330-698*)
3. Girls Routine 14 yrs	No vaccination	6,741 (6,979-12,045)	1,365 (1,365-1,369)	203 (114-203*)	10,259 (10,569-19,477)	5,846 (5,302-5,846*)	570 (272-570*)
4. Girls Routine 9 yrs & MAC 9-14 yrs	No vaccination	7,223 (7,572-12,533)	1,419 (1,418-1,423)	196 (113-196*)	11,203 (11,083-21,243)	6,573 (6,000-6,573*)	587 (286-593)
5. Girls Routine 9 yrs & 14-yr-old catch-up[¥]	No vaccination	7,154 (7,424-12,528)	1,419 (1,419-1,423)	198 (113-198*)	10,981 (11,310-21,033)	6,556 (6,020-6,556*)	597 (287-597*)
6. Girls Routine 9 yrs 5-yr extended interval & 14-yr-old catch-up[α]	No vaccination	7,154 (7,424-12,528)	1,392 (1,392-1,396)	195 (111-195*)	10,981 (11,310-21,033)	6,179 (5,642-6,179*)	563 (269-563*)
7. Girls Routine 14 yrs & later switch to Routine 9 yrs	No vaccination	7,060 (7,307-12,279)	1,419 (1,419-1,423)	201 (116-201*)	10,645 (11,517-20,991)	6,476 (5,918-6,476*)	608 (283-608*)
**VIETNAM**							
**Vaccine cost per dose[¶]: $4.60**							
1. Girls Routine 9 yrs	No vaccination	254 (265-716)	97 (97-97)	381 (135-381*)	307 (336-1,031)	342 (268-342*)	1,115 (259-1,115*)
2. Girls Routine 9 yrs 5-yr extended interval [γ]	No vaccination	254 (265-716)	95 (95-95)	373 (133-373*)	307 (336-1,031)	320 (246-320*)	1,042 (238-1,042*)
3. Girls Routine 14 yrs	No vaccination	278 (291-769)	97 (97-97)	348 (126-348*)	379 (398-1,166)	334 (254-334*)	883 (217-883*)
4. Girls Routine 9 yrs & MAC 9-14 yrs	No vaccination	279 (298-763)	101 (101-101)	361 (132-361*)	383 (433-1,145)	381 (304-381*)	996 (266-996*)
5. Girls Routine 9 yrs & 14-yr-old catch-up[¥]	No vaccination	282 (286-754)	101 (101-101)	357 (134-357*)	392 (390-1,161)	378 (300-379)	963 (259-969)
6. Girls Routine 9 yrs 5-yr extended interval & 14-yr-old catch-up[α]	No vaccination	282 (286-754)	99 (99-99)	350 (131-350*)	392 (390-1,161)	355 (278-357)	907 (239-912)
7. Girls Routine 14 yrs & later switch to Routine 9 yrs	No vaccination	287 (293-757)	101 (101-101)	351 (133-351*)	408 (428-1,159)	370 (295-370*)	907 (254-907*)
**Vaccine cost per dose[¶]: $7.50**							
1. Girls Routine 9 yrs	No vaccination	254 (265-716)	97 (97-97)	381 (135-381*)	307 (336-1,031)	432 (358-432*)	1,406 (346-1,406*)
2. Girls Routine 9 yrs 5-yr extended interval [γ]	No vaccination	254 (265-716)	95 (95-95)	373 (133-373*)	307 (336-1,031)	404 (330-404*)	1,316 (319-1,316*)
3. Girls Routine 14 yrs	No vaccination	278 (291-769)	97 (97-97)	348 (126-348*)	379 (398-1,166)	424 (344-424*)	1,120 (294-1,120*)
4. Girls Routine 9 yrs & MAC 9-14 yrs	No vaccination	279 (298-763)	101 (101-101)	361 (132-361*)	383 (433-1,145)	482 (405-482*)	1,259 (354-1,259*)
5. Girls Routine 9 yrs & 14-yr-old catch-up[¥]	No vaccination	282 (286-754)	101 (101-101)	357 (134-357*)	392 (390-1,161)	478 (401-479)	1,219 (345-1,225)
6. Girls Routine 9 yrs 5-yr extended interval & 14-yr-old catch-up[α]	No vaccination	282 (286-754)	99 (99-99)	350 (131-350*)	392 (390-1,161)	450 (373-452)	1,149 (321-1,155)
7. Girls Routine 14 yrs & later switch to Routine 9 yrs	No vaccination	287 (293-757)	101 (101-101)	351 (133-351*)	408 (428-1,159)	468 (394-468*)	1,149 (339-1,149*)
**UGANDA**							
**Vaccine cost per dose[¶]: $4.60**							
1. Girls Routine 9 yrs	No vaccination	460 (360-585)	38 (38-38)	83 (65-105)	694 (503-984)	30 (9-44)	43 (9-88)
2. Girls Routine 9 yrs 5-yr extended interval [γ]	No vaccination	460 (360-585)	37 (37-37)	81 (64-103)	694 (503-984)	25 (4-39)	35 (4-78)
3. Girls Routine 14 yrs	No vaccination	486 (378-619)	38 (38-38)	78 (61-100)	783 (586-1,062)	22 (2-36)	28 (2-62)

		Efficiency (undiscounted)			Cost-effectiveness (discounted)		
		Number of cases averted (thousands)	Additional number of doses (millions)	NNV	DALYs averted (thousands)	Additional cost (millions)	ICER
**Vaccination strategies** [α]	**Comparator for NNV and ICER**	**Mean (80%UI)**	**Mean (80%UI)**	**Mean (80%UI)**	**Mean (80%UI)**	**Mean (80%UI)**	**Mean (80%UI)**
4. Girls Routine 9 yrs & MAC 9-14 yrs	No vaccination	494 (386-628)	39 (39-39)	80 (63-102)	795 (620-1,110)	32 (10-44)	40 (8-71)
5. Girls Routine 9 yrs & 14-yr-old catch-up[¥]	No vaccination	495 (383-627)	39 (39-39)	80 (63-103)	802 (609-1,115)	31 (9-45)	38 (8-74)
6. Girls Routine 9 yrs 5-yr extended interval & 14-yr-old catch-up[α]	No vaccination	495 (383-627)	39 (39-39)	78 (62-101)	802 (609-1,115)	26 (4-40)	32 (4-66)
7. Girls Routine 14 yrs & later switch to Routine 9 yrs	No vaccination	492 (379-622)	39 (39-39)	80 (63-104)	787 (608-1,110)	31 (7-44)	39 (6-71)
**Vaccine cost per dose[¶]: $7.50**							
1. Girls Routine 9 yrs	No vaccination	460 (360-585)	38 (38-38)	83 (65-105)	694 (503-984)	65 (44-79)	93 (44-158)
2. Girls Routine 9 yrs 5-yr extended interval [γ]	No vaccination	460 (360-585)	37 (37-37)	81 (64-103)	694 (503-984)	57 (37-72)	83 (37-144)
3. Girls Routine 14 yrs	No vaccination	486 (378-619)	38 (38-38)	78 (61-100)	783 (586-1,062)	57 (37-71)	73 (36-121)
4. Girls Routine 9 yrs & MAC 9-14 yrs	No vaccination	494 (386-628)	39 (39-39)	80 (63-102)	795 (620-1,110)	71 (49-83)	89 (43-134)
5. Girls Routine 9 yrs & 14-yr-old catch-up[¥]	No vaccination	495 (383-627)	39 (39-39)	80 (63-103)	802 (609-1,115)	70 (48-84)	87 (43-138)
6. Girls Routine 9 yrs 5-yr extended interval & 14-yr-old catch-up[α]	No vaccination	495 (383-627)	39 (39-39)	78 (62-101)	802 (609-1,115)	63 (41-77)	78 (36-127)
7. Girls Routine 14 yrs & later switch to Routine 9 yrs	No vaccination	492 (379-622)	39 (39-39)	80 (63-104)	787 (608-1,110)	69 (45-83)	88 (41-134)
**NIGERIA**							
**Vaccine cost per dose[¶]: $4.60**							
1. Girls Routine 9 yrs	No vaccination	1,254 (1,063-1,921)	209 (208-209)	166 (109-196)	1,523 (1,354-2,826)	684 (603-697)	449 (214-515)
2. Girls Routine 9 yrs 5-yr extended interval [γ]	No vaccination	1,254 (1,063-1,921)	205 (204-205)	163 (106-192)	1,523 (1,354-2,826)	637 (555-650)	418 (197-480)
3. Girls Routine 14 yrs	No vaccination	1,301 (1,178-1,953)	209 (208-209)	160 (107-178)	1,692 (1,517-2,990)	669 (589-684)	395 (198-452)
4. Girls Routine 9 yrs & MAC 9-14 yrs	No vaccination	1,358 (1,162-2,045)	217 (217-218)	160 (106-187)	1,780 (1,477-3,215)	763 (673-789)	429 (207-534)
5. Girls Routine 9 yrs & 14-yr-old catch-up[¥]	No vaccination	1,351 (1,152-2,032)	217 (217-217)	161 (107-188)	1,763 (1,553-3,211)	759 (673-782)	431 (210-501)
6. Girls Routine 9 yrs 5-yr extended interval & 14-yr-old catch-up[α]	No vaccination	1,351 (1,152-2,032)	213 (213-213)	158 (105-185)	1,763 (1,553-3,211)	712 (626-735)	404 (195-470)
7. Girls Routine 14 yrs & later switch to Routine 9 yrs	No vaccination	1,354 (1,168-2,031)	217 (217-217)	160 (107-186)	1,777 (1,466-3,201)	745 (657-770)	419 (206-525)
**Vaccine cost per dose[¶]: $7.50**							
1. Girls Routine 9 yrs	No vaccination	1,254 (1,063-1,921)	209 (208-209)	166 (109-196)	1,523 (1,354-2,826)	876 (796-890)	575 (283-658)
2. Girls Routine 9 yrs 5-yr extended interval [γ]	No vaccination	1,254 (1,063-1,921)	205 (204-205)	163 (106-192)	1,523 (1,354-2,826)	818 (737-831)	537 (262-614)
3. Girls Routine 14 yrs	No vaccination	1,301 (1,178-1,953)	209 (208-209)	160 (107-178)	1,692 (1,517-2,990)	861 (782-876)	509 (262-579)
4. Girls Routine 9 yrs & MAC 9-14 yrs	No vaccination	1,358 (1,162-2,045)	217 (217-218)	160 (106-187)	1,780 (1,477-3,215)	979 (890-1,005)	550 (274-680)
5. Girls Routine 9 yrs & 14-yr-old catch-up[¥]	No vaccination	1,351 (1,152-2,032)	217 (217-217)	161 (107-188)	1,763 (1,553-3,211)	974 (888-997)	553 (277-639)
6. Girls Routine 9 yrs 5-yr extended interval & 14-yr-old catch-up[α]	No vaccination	1,351 (1,152-2,032)	213 (213-213)	158 (105-185)	1,763 (1,553-3,211)	916 (829-938)	519 (259-601)
7. Girls Routine 14 yrs & later switch to Routine 9 yrs	No vaccination	1,354 (1,168-2,031)	217 (217-217)	160 (107-186)	1,777 (1,466-3,201)	957 (869-982)	538 (272-670)

MAC: Multiple age cohort vaccination; DALY: Disability-adjusted life-years ; ICER: Incremental cost-effectiveness ratio ; UI: Uncertainty interval ; NNV: Number of doses needed to prevent 1 case of cervical cancer

Base case: 2 doses, Vaccine efficacy against HPV16/18/31/33/45/52/58 =100%, Vaccine duration=Lifetime, Vaccination coverage=80%

Predictions: Mean of the 10 best fit parameter sets to Globocan 2020 (80% UI: 10th-90th percentiles of the 50 parameter sets)

[α] Vaccination strategies are described in Table S1

[¶] Vaccine cost per dose including administration costs

[γ] 1 dose at 9 yrs and 1 dose at 14 yrs (assuming the same coverage)

[¥] 2 doses at 9 yrs and a 5 yrs catch-up at 14 yr-olds

[α] 1 dose at 9 yrs and 1 dose at 14 yrs (assuming the same coverage) + a 5 yrs catch-up of 14-yr-olds

[*] Maximum value is the mean of the 10 best fit parameter sets to Globocan 2020

Table S7. Incremental efficiency and cost-effectiveness of different 1-dose HPV vaccination strategies varying the number of cohorts, age, and population (girls-only or girls & boys) targeted, assuming 1-dose duration of protection of 20 years

		Efficiency (undiscounted)						Cost-effectiveness (discounted)					
		Number of cases averted (thousands)		Additional number of doses (millions)		NNV		DALYs averted (thousands)		Additional cost (millions)		ICER	
Vaccination strategies [α]	Comparator for NNV and ICER	Mean	(80%UI)	Mean	(80%UI)	Mean	(80%UI)	Mean	(80%UI)	Mean	(80%UI)	Mean	(80%UI)
**INDIA**													
**Vaccine cost per dose[¶]: $4.60**													
2c. Girls Routine 1 dose 9 yrs & MAC 9-14 yrs	No vaccination	4,269	(4,204-7,368)	709	(709-711)	166	(97-169)	7,806	(7,743-14,945)	2,470	(2,094-2,507)	316	(142-319)
2a. Girls Routine 2 doses 9 yrs & MAC 9-14 yrs	vs. 2c	2,953	(2,886-5,439)	709	(709-711)	240	(131-246)	3,396	(3,308-7,230)	2,693	(2,437-2,701)	793	(337-813)
3c. Girls Routine 1 dose 9 yrs & MAC 9-18 yrs [γ]	vs. 2c	312	(108-600)	80	(80-80)	256	(133-745)	821	(213-2,159)	898	(811-941)	Extended Dominated	
2d. Girls&Boys Routine 1 dose 9 yrs & MAC 9-14 yrs[¥]	vs. 2c	885	(829-1,707)	710	(709-711)	802	(417-855)	1,340	(1,269-3,655)	2,861	(2,722-2,874)	Extended Dominated	
**Vaccine cost per dose[¶]: $7.50**													
2c. Girls Routine 1 dose 9 yrs & MAC 9-14 yrs	No vaccination	4,269	(4,204-7,368)	709	(709-711)	166	(97-169)	7,806	(7,743-14,945)	3,175	(2,800-3,211)	407	(189-410)
2a. Girls Routine 2 doses 9 yrs & MAC 9-14 yrs	vs. 2c	2,953	(2,886-5,439)	709	(709-711)	240	(131-246)	3,396	(3,308-7,230)	3,398	(3,144-3,407)	1,000	(435-1,026)
3c. Girls Routine 1 dose 9 yrs & MAC 9-18 yrs [γ]	vs. 2c	312	(108-600)	80	(80-80)	256	(133-745)	821	(213-2,159)	1,126	(1,038-1,171)	Extended Dominated	
2d. Girls&Boys Routine 1 dose 9 yrs & MAC 9-14 yrs[¥]	vs. 2c	885	(829-1,707)	710	(709-711)	802	(417-855)	1,340	(1,269-3,655)	3,566	(3,428-3,581)	Extended Dominated	
**VIETNAM**													
**Vaccine cost per dose[¶]: $4.60**													
2c. Girls Routine 1 dose 9 yrs & MAC 9-14 yrs	No vaccination	105	(100-320)	50	(50-51)	480	(158-504)	189	(182-649)	193	(148-193)	Extended Dominated	
2a. Girls Routine 2 doses 9 yrs & MAC 9-14 yrs	vs. 2c	175	(181-454)	50	(50-51)	289	(111-289*)	194	(227-556)	188	(150-188*)	Extended Dominated	
3c. Girls Routine 1 dose 9 yrs & MAC 9-18 yrs [γ]	vs. 2c	23	(8-66)	6	(6-6)	253	(89-299)	67	(15-199)	63	(48-69)	943	(241-4,578)
2d. Girls&Boys Routine 1 dose 9 yrs & MAC 9-14 yrs[¥]	vs. 2c	55	(47-135)	50	(50-50)	916	(374-1,091)	105	(53-271)	199	(182-206)	Extended Dominated	
**Vaccine cost per dose[¶]: $7.50**													
2c. Girls Routine 1 dose 9 yrs & MAC 9-14 yrs	No vaccination	105	(100-320)	50	(50-51)	480	(158-504)	189	(182-649)	243	(199-244)	Extended Dominated	
2a. Girls Routine 2 doses 9 yrs & MAC 9-14 yrs	vs. 2c	175	(181-454)	50	(50-51)	289	(111-289*)	194	(227-556)	239	(201-239*)	Extended Dominated	
3c. Girls Routine 1 dose 9 yrs & MAC 9-18 yrs [γ]	vs. 2c	23	(8-66)	6	(6-6)	253	(89-299)	67	(15-199)	80	(65-86)	1,193	(325-5,684)
2d. Girls&Boys Routine 1 dose 9 yrs & MAC 9-14 yrs[¥]	vs. 2c	55	(47-135)	50	(50-50)	916	(374-1,091)	105	(53-271)	249	(233-257)	Extended Dominated	
**UGANDA**													
**Vaccine cost per dose[¶]: $4.60**													
2c. Girls Routine 1 dose 9 yrs & MAC 9-14 yrs	No vaccination	297	(230-387)	20	(20-20)	67	(51-86)	561	(424-778)	5	(-12-15)	9	(CS-37)
2a. Girls Routine 2 doses 9 yrs & MAC 9-14 yrs	vs. 2c	197	(134-270)	20	(20-20)	100	(73-246)	234	(161-335)	27	(18-34)	114	(53-205)
3c. Girls Routine 1 dose 9 yrs & MAC 9-18 yrs [γ]	vs. 2c	24	(13-39)	2	(2-2)	96	(58-179)	71	(38-122)	10	(5-12)	Extended Dominated	
2d. Girls&Boys Routine 1 dose 9 yrs & MAC 9-14 yrs[¥]	vs. 2c	61	(45-94)	20	(20-20)	323	(211-436)	105	(56-168)	40	(35-44)	Extended Dominated	
**Vaccine cost per dose[¶]: $7.50**													
2c. Girls Routine 1 dose 9 yrs & MAC 9-14 yrs	No vaccination	297	(230-387)	20	(20-20)	67	(51-86)	561	(424-778)	25	(8-35)	44	(10-82)
2a. Girls Routine 2 doses 9 yrs & MAC 9-14 yrs	vs. 2c	197	(134-270)	20	(20-20)	100	(73-246)	234	(161-335)	47	(37-53)	198	(112-327)
3c. Girls Routine 1 dose 9 yrs & MAC 9-18 yrs [γ]	vs. 2c	24	(13-39)	2	(2-2)	96	(58-179)	71	(38-122)	16	(12-19)	Extended Dominated	
2d. Girls&Boys Routine 1 dose 9 yrs & MAC 9-14 yrs[¥]	vs. 2c	61	(45-94)	20	(20-20)	323	(211-436)	105	(56-168)	60	(55-64)	Extended Dominated	
**NIGERIA**													
**Vaccine cost per dose[¶]: $4.60**													
2c. Girls Routine 1 dose 9 yrs & MAC 9-14 yrs	No vaccination	963	(820-1,421)	109	(108-109)	113	(77-132)	1,388	(1,119-2,516)	346	(279-367)	249	(110-330)
2a. Girls Routine 2 doses 9 yrs & MAC 9-14 yrs	vs. 2c	394	(349-691)	109	(108-109)	275	(157-275*)	392	(345-828)	417	(384-421)	Extended Dominated	

Vaccination strategies [α]	Comparator for NNV and ICER	Efficiency (undiscounted)			Cost-effectiveness (discounted)		
		Number of cases averted (thousands)	Additional number of doses (millions)	NNV	DALYs averted (thousands)	Additional cost (millions)	ICER
		Mean (80%UI)	Mean (80%UI)	Mean (80%UI)	Mean (80%UI)	Mean (80%UI)	Mean (80%UI)
3c. Girls Routine 1 dose 9 yrs & MAC 9-18 yrs [γ]	vs. 2c	67 (44-104)	12 (12-12)	186 (119-284)	160 (105-335)	135 (122-140)	843 (366-1,333)
2d. Girls&Boys Routine 1 dose 9 yrs & MAC 9-14 yrs[¥]	vs. 2c	140 (109-270)	109 (108-109)	773 (403-991)	196 (150-469)	437 (418-442)	Extended Dominated
**Vaccine cost per dose[¶]: $7.50**							
2c. Girls Routine 1 dose 9 yrs & MAC 9-14 yrs	No vaccination	963 (820-1,421)	109 (108-109)	113 (77-132)	1,388 (1,119-2,516)	454 (387-475)	327 (153-427)
2a. Girls Routine 2 doses 9 yrs & MAC 9-14 yrs	vs. 2c	394 (349-691)	109 (108-109)	275 (157-275*)	392 (345-828)	525 (492-529)	Extended Dominated
3c. Girls Routine 1 dose 9 yrs & MAC 9-18 yrs [γ]	vs. 2c	67 (44-104)	12 (12-12)	186 (119-284)	160 (105-335)	170 (158-175)	1,065 (472-1,669)
2d. Girls&Boys Routine 1 dose 9 yrs & MAC 9-14 yrs[¥]	vs. 2c	140 (109-270)	109 (108-109)	773 (403-991)	196 (150-469)	545 (526-550)	Extended Dominated

MAC: Multiple age cohort vaccination ; DALY: Disability-adjusted life-years ; ICER: Incremental cost-effectiveness ratio ; UI: Uncertainty interval ; NNV: Number of doses needed to prevent 1 case of cervical cancer

Base case: 2 doses, Vaccine efficacy against HPV16/18/31/33/45/52/58 =100%, Vaccine duration=Lifetime, Vaccination coverage=80%

Predictions: Mean of the 10 best fit parameter sets to Globocan 2020 (80% UI: 10th-90th percentiles of the 50 parameter sets)

[α] Vaccination strategies are described in Table S1

[¶] Vaccine cost per dose including administration costs

[γ] Girls >14 years always have 3 doses

[¥] Girls & boys MAC: Girls age as indicated and boys always ≤ 14 yrs

[*] Maximum value is the mean of the 10 best fit parameter sets to Globocan 2020

Table S8. Incremental efficiency and cost-effectiveness of different 1-dose HPV vaccination strategies varying the number of cohorts, age, and population (girls-only or girls & boys) targeted, assuming 1-dose efficacy of 85%

		Efficiency (undiscounted)			Cost-effectiveness (discounted)		
Vaccination strategies [α]	Comparator for NNV and ICER	Number of cases averted (thousands) Mean (80%UI)	Additional number of doses (millions) Mean (80%UI)	NNV Mean (80%UI)	DALYs averted (thousands) (vs. No vaccination) Mean (80%UI)	Additional cost (millions) (vs. No vaccination) Mean (80%UI)	ICER Mean (80%UI)
**INDIA**							
**Vaccine cost per dose[¶]: $4.60**							
2c. Girls Routine 1 dose 9 yrs & MAC 9-14 yrs	No vaccination	6,248 (6,570-10,805)	709 (709-711)	114 (66-114*)	9,777 (9,607-18,102)	2,314 (1,844-2,322)	237 (102-242)
2a. Girls Routine 2 doses 9 yrs & MAC 9-14 yrs	vs. 2c	974 (980-1,837)	709 (709-711)	728 (387-728*)	1,426 (846-3,573)	2,849 (2,729-2,884)	Extended Dominated
3c. Girls Routine 1 dose 9 yrs & MAC 9-18 yrs [γ]	vs. 2c	196 (133-600)	80 (80-80)	407 (130-494)	436 (47-2,221)	926 (812-953)	Extended Dominated
2d. Girls&Boys Routine 1 dose 9 yrs & MAC 9-14 yrs	vs. 2c	1,206 (1,103-2,352)	710 (709-711)	589 (302-644)	1,627 (1,202-4,209)	2,841 (2,670-2,867)	Extended Dominated
3a. Girls Routine 2 doses 9 yrs & MAC 9-18 yrs [γ]	vs. 3c	1,065 (1,070-1,919)	709 (709-711)	666 (371-666*)	1,873 (1,361-3,673)	2,817 (2,710-2,857)	Extended Dominated
4c.Girls Routine 1 dose 9 yrs & MAC 9-25 yrs [γ]	vs. 3c	232 (93-523)	139 (139-139)	599 (265-1,385)	789 (0-1,831)	1,606 (1,534-1,660)	Extended Dominated
3d. Girls&Boys Routine 1 dose 9 yrs & MAC 9-18 yrs[¥]	vs. 3c	1,299 (1,148-2,334)	710 (709-711)	546 (304-618)	2,156 (1,386-4,015)	2,805 (2,694-2,853)	1,301 (671-2,063)
3b.Girls&Boys Routine 2 doses 9 yrs & MAC 9-18 yrs[¥]	vs. 3d	536 (523-982)	1,419 (1,418-1,423)	2,646 (1,449-2,715)	823 (616-1,810)	5,831 (5,775-5,855)	Extended Dominated
4d. Girls&Boys Routine 1 doses 9 yrs & MAC 9-25 yrs[¥]	vs. 3d	192 (130-451)	139 (139-139)	726 (303-782)	534 (24-1,597)	1,621 (1,546-1,665)	3,037 (973-X)
**Vaccine cost per dose[¶]: $7.50**							
2c. Girls Routine 1 dose 9 yrs & MAC 9-14 yrs	No vaccination	6,248 (6,570-10,805)	709 (709-711)	114 (66-114*)	9,777 (9,607-18,102)	3,019 (2,551-3,027)	309 (141-316)
2a. Girls Routine 2 doses 9 yrs & MAC 9-14 yrs	vs. 2c	974 (980-1,837)	709 (709-711)	728 (387-728*)	1,426 (846-3,573)	3,554 (3,436-3,591)	Extended Dominated
3c. Girls Routine 1 dose 9 yrs & MAC 9-18 yrs [γ]	vs. 2c	196 (133-600)	80 (80-80)	407 (130-494)	436 (47-2,221)	1,155 (1,040-1,183)	Extended Dominated
2d. Girls&Boys Routine 1 dose 9 yrs & MAC 9-14 yrs	vs. 2c	1,206 (1,103-2,352)	710 (709-711)	589 (302-644)	1,627 (1,202-4,209)	3,547 (3,378-3,573)	Extended Dominated
3a. Girls Routine 2 doses 9 yrs & MAC 9-18 yrs [γ]	vs. 3c	1,065 (1,070-1,919)	709 (709-711)	666 (371-666*)	1,873 (1,361-3,673)	3,522 (3,417-3,563)	Extended Dominated
4c.Girls Routine 1 dose 9 yrs & MAC 9-25 yrs [γ]	vs. 3c	232 (93-523)	139 (139-139)	599 (265-1,385)	789 (0-1,831)	2,004 (1,931-2,057)	Extended Dominated
3d. Girls&Boys Routine 1 dose 9 yrs & MAC 9-18 yrs[¥]	vs. 3c	1,299 (1,148-2,334)	710 (709-711)	546 (304-618)	2,156 (1,386-4,015)	3,510 (3,401-3,559)	1,628 (847-2,571)
3b.Girls&Boys Routine 2 doses 9 yrs & MAC 9-18 yrs[¥]	vs. 3d	536 (523-982)	1,419 (1,418-1,423)	2,646 (1,449-2,715)	823 (616-1,810)	7,242 (7,186-7,266)	Extended Dominated
4d. Girls&Boys Routine 1 doses 9 yrs & MAC 9-25 yrs[¥]	vs. 3d	192 (130-451)	139 (139-139)	726 (303-782)	534 (24-1,597)	2,018 (1,943-2,062)	3,782 (1,223-X)
**VIETNAM**							
**Vaccine cost per dose[¶]: $4.60**							
2c. Girls Routine 1 dose 9 yrs & MAC 9-14 yrs	No vaccination	244 (258-653)	50 (50-51)	207 (77-207*)	344 (349-1,019)	174 (108-175)	508 (105-508*)
2a. Girls Routine 2 doses 9 yrs & MAC 9-14 yrs	vs. 2c	36 (37-113)	50 (50-51)	1,406 (446-1,406*)	39 (0-200)	206 (189-212)	Extended Dominated
3c. Girls Routine 1 dose 9 yrs & MAC 9-18 yrs [γ]	vs. 2c	22 (14-65)	6 (6-6)	266 (90-297)	70 (37-216)	63 (47-67)	894 (217-1,847)
2d. Girls&Boys Routine 1 dose 9 yrs & MAC 9-14 yrs	vs. 2c	66 (72-178)	50 (50-50)	762 (283-762*)	111 (96-313)	198 (177-200)	Extended Dominated
3a. Girls Routine 2 doses 9 yrs & MAC 9-18 yrs [γ]	vs. 3c	39 (31-126)	50 (50-51)	1,300 (399-1,636)	45 (1-234)	205 (186-212)	Extended Dominated
4c.Girls Routine 1 dose 9 yrs & MAC 9-25 yrs [γ]	vs. 3c	14 (0-47)	10 (10-10)	704 (214-2,143)	41 (0-173)	117 (103-125)	2,855 (592-X)
3d. Girls&Boys Routine 1 dose 9 yrs & MAC 9-18 yrs[¥]	vs. 3c	56 (51-165)	50 (50-50)	905 (304-990)	64 (30-288)	203 (180-208)	Extended Dominated
3b.Girls&Boys Routine 2 doses 9 yrs & MAC 9-18 yrs[¥]	vs. 3d	24 (19-72)	101 (101-101)	4,233 (1,395-4,873)	42 (0-168)	415 (401-423)	Extended Dominated
4d. Girls&Boys Routine 1 doses 9 yrs & MAC 9-25 yrs[¥]	vs. 3d	9 (3-49)	10 (10-10)	1,083 (204-1,270)	20 (0-169)	119 (102-124)	5,888 (603-X)
**Vaccine cost per dose[¶]: $7.50**							
2c. Girls Routine 1 dose 9 yrs & MAC 9-14 yrs	No vaccination	244 (258-653)	50 (50-51)	207 (77-207*)	344 (349-1,019)	225 (159-225)	655 (154-655*)
2a. Girls Routine 2 doses 9 yrs & MAC 9-14 yrs	vs. 2c	36 (37-113)	50 (50-51)	1,406 (446-1,406*)	39 (0-200)	257 (240-263)	Extended Dominated
3c. Girls Routine 1 dose 9 yrs & MAC 9-18 yrs [γ]	vs. 2c	22 (14-65)	6 (6-6)	266 (90-297)	70 (37-216)	79 (64-84)	1,133 (294-2,313)

		Efficiency (undiscounted)			Cost-effectiveness (discounted)		
		Number of cases averted (thousands)	Additional number of doses (millions)	NNV	DALYs averted (thousands) (vs. No vaccination)	Additional cost (millions) (vs. No vaccination)	ICER
**Vaccination strategies [α]**	**Comparator for NNV and ICER**	**Mean (80%UI)**	**Mean (80%UI)**	**Mean (80%UI)**	**Mean (80%UI)**	**Mean (80%UI)**	**Mean (80%UI)**
2d. Girls&Boys Routine 1 dose 9 yrs & MAC 9-14 yrs	vs. 2c	66 (72-178)	50 (50-50)	762 (283-762*)	111 (96-313)	248 (227-250)	Extended Dominated
3a. Girls Routine 2 doses 9 yrs & MAC 9-18 yrs [γ]	vs. 3c	39 (31-126)	50 (50-51)	1,300 (399-1,636)	45 (1-234)	256 (236-262)	Extended Dominated
4c.Girls Routine 1 dose 9 yrs & MAC 9-25 yrs [γ]	vs. 3c	14 (0-47)	10 (10-10)	704 (214-2,143)	41 (0-173)	146 (132-154)	3,567 (760-X)
3d. Girls&Boys Routine 1 dose 9 yrs & MAC 9-18 yrs[¥]	vs. 3c	56 (51-165)	50 (50-50)	905 (304-990)	64 (30-288)	253 (230-258)	Extended Dominated
3b.Girls&Boys Routine 2 doses 9 yrs & MAC 9-18 yrs[¥]	vs. 3d	24 (19-72)	101 (101-101)	4,233 (1,395-4,873)	42 (0-168)	516 (501-524)	Extended Dominated
4d. Girls&Boys Routine 1 doses 9 yrs & MAC 9-25 yrs[¥]	vs. 3d	9 (3-49)	10 (10-10)	1,083 (204-1,270)	20 (0-169)	148 (132-154)	7,323 (775-X)
**UGANDA**							
**Vaccine cost per dose[¶]: $4.60**							
2c. Girls Routine 1 dose 9 yrs & MAC 9-14 yrs	No vaccination	424 (327-544)	20 (20-20)	47 (36-60)	684 (533-952)	CS (CS-4)	CS (CS-7)
2a. Girls Routine 2 doses 9 yrs & MAC 9-14 yrs	vs. 2c	70 (52-92)	20 (20-20)	281 (216-381)	112 (74-163)	39 (35-42)	Extended Dominated
3c. Girls Routine 1 dose 9 yrs & MAC 9-18 yrs [γ]	vs. 2c	27 (13-35)	2 (2-2)	84 (65-178)	88 (36-122)	8 (5-13)	95 (45-363)
2d. Girls&Boys Routine 1 dose 9 yrs & MAC 9-14 yrs	vs. 2c	102 (78-132)	20 (20-20)	195 (150-254)	167 (100-232)	35 (30-40)	Extended Dominated
3a. Girls Routine 2 doses 9 yrs & MAC 9-18 yrs [γ]	vs. 3c	70 (56-94)	20 (20-20)	283 (211-351)	108 (78-165)	40 (34-42)	Extended Dominated
4c.Girls Routine 1 dose 9 yrs & MAC 9-25 yrs [γ]	vs. 3c	17 (9-37)	4 (4-4)	233 (105-438)	52 (22-139)	23 (15-25)	Extended Dominated
3d. Girls&Boys Routine 1 dose 9 yrs & MAC 9-18 yrs[¥]	vs. 3c	95 (82-130)	20 (20-20)	207 (152-241)	137 (109-227)	37 (30-39)	Extended Dominated
3b.Girls&Boys Routine 2 doses 9 yrs & MAC 9-18 yrs[¥]	vs. 3d	42 (25-50)	40 (39-40)	952 (786-1,593)	79 (33-108)	90 (87-94)	Extended Dominated
4d. Girls&Boys Routine 1 doses 9 yrs & MAC 9-25 yrs[¥]	vs. 3d	19 (7-28)	4 (4-4)	201 (140-565)	69 (9-101)	21 (18-26)	300 (180-2,889)
**Vaccine cost per dose[¶]: $7.50**							
2c. Girls Routine 1 dose 9 yrs & MAC 9-14 yrs	No vaccination	424 (327-544)	20 (20-20)	47 (36-60)	684 (533-952)	12 (CS-23)	18 (CS-43)
2a. Girls Routine 2 doses 9 yrs & MAC 9-14 yrs	vs. 2c	70 (52-92)	20 (20-20)	281 (216-381)	112 (74-163)	59 (55-62)	Extended Dominated
3c. Girls Routine 1 dose 9 yrs & MAC 9-18 yrs [γ]	vs. 2c	27 (13-35)	2 (2-2)	84 (65-178)	88 (36-122)	15 (12-19)	168 (98-545)
2d. Girls&Boys Routine 1 dose 9 yrs & MAC 9-14 yrs	vs. 2c	102 (78-132)	20 (20-20)	195 (150-254)	167 (100-232)	54 (49-60)	Extended Dominated
3a. Girls Routine 2 doses 9 yrs & MAC 9-18 yrs [γ]	vs. 3c	70 (56-94)	20 (20-20)	283 (211-351)	108 (78-165)	59 (54-61)	Extended Dominated
4c.Girls Routine 1 dose 9 yrs & MAC 9-25 yrs [γ]	vs. 3c	17 (9-37)	4 (4-4)	233 (105-438)	52 (22-139)	34 (26-36)	Extended Dominated
3d. Girls&Boys Routine 1 dose 9 yrs & MAC 9-18 yrs[¥]	vs. 3c	95 (82-130)	20 (20-20)	207 (152-241)	137 (109-227)	57 (50-59)	Extended Dominated
3b.Girls&Boys Routine 2 doses 9 yrs & MAC 9-18 yrs[¥]	vs. 3d	42 (25-50)	40 (39-40)	952 (786-1,593)	79 (33-108)	129 (127-133)	Extended Dominated
4d. Girls&Boys Routine 1 doses 9 yrs & MAC 9-25 yrs[¥]	vs. 3d	19 (7-28)	4 (4-4)	201 (140-565)	69 (9-101)	32 (29-38)	460 (290-4,105)
**NIGERIA**							
**Vaccine cost per dose[¶]: $4.60**							
2c. Girls Routine 1 dose 9 yrs & MAC 9-14 yrs	No vaccination	1,158 (975-1,748)	109 (108-109)	94 (62-111)	1,517 (1,231-2,841)	332 (250-356)	219 (89-289)
2a. Girls Routine 2 doses 9 yrs & MAC 9-14 yrs	vs. 2c	199 (167-321)	109 (108-109)	545 (338-652)	263 (218-496)	431 (414-435)	Extended Dominated
3c. Girls Routine 1 dose 9 yrs & MAC 9-18 yrs [γ]	vs. 2c	62 (45-105)	12 (12-12)	200 (117-275)	169 (112-359)	135 (120-141)	797 (336-1,233)
2d. Girls&Boys Routine 1 dose 9 yrs & MAC 9-14 yrs	vs. 2c	253 (227-429)	109 (108-109)	429 (253-477)	314 (265-657)	427 (403-431)	Extended Dominated
3a. Girls Routine 2 doses 9 yrs & MAC 9-18 yrs [γ]	vs. 3c	204 (167-308)	109 (108-109)	531 (353-649)	271 (216-506)	429 (411-436)	Extended Dominated
4c.Girls Routine 1 dose 9 yrs & MAC 9-25 yrs [γ]	vs. 3c	32 (23-65)	21 (21-21)	659 (327-915)	86 (21-221)	246 (235-252)	Extended Dominated
3d. Girls&Boys Routine 1 dose 9 yrs & MAC 9-18 yrs[¥]	vs. 3c	257 (204-430)	109 (108-109)	423 (252-532)	309 (237-629)	427 (406-434)	1,382 (646-1,822)
3b.Girls&Boys Routine 2 doses 9 yrs & MAC 9-18 yrs[¥]	vs. 3d	119 (99-195)	217 (217-217)	1,823 (1,116-2,202)	185 (124-401)	888 (874-894)	Extended Dominated
4d. Girls&Boys Routine 1 doses 9 yrs & MAC 9-25 yrs[¥]	vs. 3d	32 (18-71)	21 (21-21)	657 (298-975)	89 (49-259)	246 (235-249)	2,756 (906-6,716)

Vaccination strategies [α]	Comparator for NNV and ICER	Efficiency (undiscounted)			Cost-effectiveness (discounted)		
		Number of cases averted (thousands)	Additional number of doses (millions)	NNV	DALYs averted (thousands) (vs. No vaccination)	Additional cost (millions) (vs. No vaccination)	ICER
		Mean (80%UI)	Mean (80%UI)	Mean (80%UI)	Mean (80%UI)	Mean (80%UI)	Mean (80%UI)
**Vaccine cost per dose[¶]: $7.50**							
2c. Girls Routine 1 dose 9 yrs & MAC 9-14 yrs	No vaccination	1,158 (975-1,748)	109 (108-109)	94 (62-111)	1,517 (1,231-2,841)	440 (358-464)	290 (127-377)
2a. Girls Routine 2 doses 9 yrs & MAC 9-14 yrs	vs. 2c	199 (167-321)	109 (108-109)	545 (338-652)	263 (218-496)	539 (522-543)	Extended Dominated
3c. Girls Routine 1 dose 9 yrs & MAC 9-18 yrs [γ]	vs. 2c	62 (45-105)	12 (12-12)	200 (117-275)	169 (112-359)	170 (156-176)	1,006 (434-1,549)
2d. Girls&Boys Routine 1 dose 9 yrs & MAC 9-14 yrs	vs. 2c	253 (227-429)	109 (108-109)	429 (253-477)	314 (265-657)	536 (511-540)	Extended Dominated
3a. Girls Routine 2 doses 9 yrs & MAC 9-18 yrs [γ]	vs. 3c	204 (167-308)	109 (108-109)	531 (353-649)	271 (216-506)	538 (519-545)	Extended Dominated
4c.Girls Routine 1 dose 9 yrs & MAC 9-25 yrs [γ]	vs. 3c	32 (23-65)	21 (21-21)	659 (327-915)	86 (21-221)	307 (296-313)	Extended Dominated
3d. Girls&Boys Routine 1 dose 9 yrs & MAC 9-18 yrs[¥]	vs. 3c	257 (204-430)	109 (108-109)	423 (252-532)	309 (237-629)	535 (515-542)	1,732 (818-2,278)
3b.Girls&Boys Routine 2 doses 9 yrs & MAC 9-18 yrs[¥]	vs. 3d	119 (99-195)	217 (217-217)	1,823 (1,116-2,202)	185 (124-401)	1,105 (1,090-1,111)	Extended Dominated
4d. Girls&Boys Routine 1 doses 9 yrs & MAC 9-25 yrs[¥]	vs. 3d	32 (18-71)	21 (21-21)	657 (298-975)	89 (49-259)	306 (296-310)	3,437 (1,141-8,344)

MAC: Multiple age cohort vaccination ; DALY: Disability-adjusted life-years ; ICER: Incremental cost-effectiveness ratio ; UI: Uncertainty interval ; NNV: Number of doses needed to prevent 1 case of cervical cancer

Base case: 2 doses, Vaccine efficacy against HPV16/18/31/33/45/52/58 =100%, Vaccine duration=Lifetime, Vaccination coverage=80%

Predictions: Mean of the 10 best fit parameter sets to Globocan 2020 (80% UI: 10th-90th percentiles of the 50 parameter sets)

[α] Vaccination strategies are described in Table S1

[¶] Vaccine cost per dose including administration costs

[γ] Girls >14 years always have 3 doses

[¥] Girls & boys MAC: Girls age as indicated and boys always ≤ 14 yrs

[*] Maximum value is the mean of the 10 best fit parameter sets to Globocan 2020

Table S9. Number needed to vaccinate (doses) to prevent 1 cervical cancer compared (vs no vaccination, undiscounted)

	India	Vietnam	Uganda	Nigeria
**Vaccination Strategies** [α]	**Mean (80% UI)**	**Mean (80% UI)**	**Mean (80% UI)**	**Mean (80% UI)**
**2 doses: Variation of the number of cohorts, age, and population targeted**				
**2 doses: Girls-only Routine vaccination, No MAC**				
1. Routine 9 yrs old	225 (127-225*)	381 (135-381*)	83 (65-105)	166 (109-196)
**2 doses: Girls-only Routine vaccination with MAC**				
2a. Routine 9 yrs old with MAC 9-14 yrs old	196 (113-196*)	361 (132-361*)	80 (63-102)	160 (106-187)
3a. Routine 9 yrs old with MAC 9-18 yrs old [γ]	200 (114-200*)	350 (129-350*)	80 (63-104)	161 (108-187)
4a. Routine 9 yrs old with MAC 9-25 yrs old [γ]	213 (121-213*)	365 (139-365*)	85 (67-109)	172 (115-200)
**2 doses: Girls&Boys Routine vaccination with MAC** [†]				
2b. Routine 9 yrs old with MAC 9-14 yrs old	355 (205-355*)	612 (227-612*)	140 (109-178)	284 (185-318)
3b. Routine 9 yrs old with MAC 9-18 yrs old [γ]	352 (205-352*)	600 (221-600*)	138 (109-177)	280 (184-314)
4b. Routine 9 yrs old with MAC 9-25 yrs old [γ]	362 (207-362*)	608 (225-608*)	141 (111-180)	288 (191-320)
**2 doses: Girls-only optimisation of 9-14 years-old vaccination**				
1. Routine 9 yrs old, No MAC	225 (127-225*)	381 (135-381*)	83 (65-105)	166 (109-196)
2. Routine 9 yrs old, 5-yr extended interval [¥], No MAC	221 (125-221*)	373 (133-373*)	81 (64-103)	163 (106-192)
3. Routine 14 yrs old, No MAC	203 (114-203*)	348 (126-348*)	78 (61-100)	160 (107-178)
4. Routine 9 yrs old with MAC 9-14 yrs old	196 (113-196*)	361 (132-361*)	80 (63-102)	160 (106-187)
5. Routine 9 yrs old with 14-yr-old catch-up	198 (113-198*)	357 (134-357*)	80 (63-103)	161 (107-188)
6. Routine 9 yrs old, 5-yr extended interval [¥] with 14-yr-old catch-up	195 (111-195*)	350 (131-350*)	78 (62-101)	158 (105-185)
7. Routine 14 yrs old & later switch to Routine 9 yrs old	201 (116-201*)	351 (133-351*)	80 (63-104)	160 (107-186)
**1 dose: Girls-only Routine vaccination with MAC, Vaccine duration = 20 years**				
2c. Routine 9 yrs old with MAC 9-14 yrs old	166 (97-169)	480 (158-504)	67 (51-86)	113 (77-132)
3c. Routine 9 yrs old with MAC 9-18 yrs old [γ]	172 (103-175)	439 (152-439*)	69 (53-90)	117 (81-138)
4c. Routine 9 yrs old with MAC 9-25 yrs old [γ]	198 (115-198)	483 (161-492)	76 (61-100)	134 (91-158)
**1 dose: Girls&Boys Routine vaccination with MAC** [†]**, Vaccine duration = 20 years**				
2d. Routine 9 yrs old with MAC 9-14 yrs old	275 (161-275*)	630 (227-630*)	110 (84-138)	197 (134-227)
3d. Routine 9 yrs old with MAC 9-18 yrs old [γ]	276 (161-276*)	595 (215-595*)	109 (85-139)	197 (134-229)
4d. Routine 9 yrs old with MAC 9-25 yrs old [γ]	291 (169-291*)	641 (216-641*)	115 (91-146)	210 (141-240)
**1 dose: Girls-only Routine vaccination with MAC, Vaccine efficacy = 85%**				
2c. Routine 9 yrs old with MAC 9-14 yrs old	114 (66-114*)	207 (77-207*)	47 (36-60)	94 (62-111)
3c. Routine 9 yrs old with MAC 9-18 yrs old [γ]	122 (69-122*)	212 (80-212*)	49 (38-63)	99 (66-114)
4c. Routine 9 yrs old with MAC 9-25 yrs old [γ]	139 (79-139*)	237 (92-237*)	55 (43-72)	114 (76-131)
**1 dose: Girls&Boys Routine vaccination with MAC** [†]**, Vaccine efficacy = 85%**				
2d. Routine 9 yrs old with MAC 9-14 yrs old	190 (110-190*)	325 (122-325*)	75 (59-95)	154 (100-180)
3d. Routine 9 yrs old with MAC 9-18 yrs old [γ]	194 (113-194*)	332 (124-332*)	77 (59-97)	155 (103-175)
4d. Routine 9 yrs old with MAC 9-25 yrs old [γ]	206 (119-206*)	353 (129-353*)	81 (63-104)	166 (110-186)

MAC: Multiple age cohort vaccination

Predictions: Mean of the 10 best fit parameter sets to Globocan 2020 (80% UI: 10th-90th percentiles of the 50 parameter sets)

Base case: 2 doses, Vaccine efficacy against HPV16/18/31/33/45/52/58 =100%, Vaccine duration=Lifetime, Vaccination coverage=80%

[α] Vaccination strategies are described in Table S1

* Maximum value is the mean of the 10 best fit parameter sets to Globocan 2020

[γ] Girls >14 years old always receive 3 doses

[¥] Extended interval: First dose at 9 years old and second dose at 14 years old

[†] Girls&Boys MAC: Girls age as indicated and boys always ≤ 14 yrs old

Table S10. Number needed to vaccinate (vaccinees) to prevent 1 cervical cancer (vs no vaccination, undiscounted)

	India	Vietnam	Uganda	Nigeria
**Vaccination Strategies** [α]	**Mean (80% UI)**	**Mean (80% UI)**	**Mean (80% UI)**	**Mean (80% UI)**
**2 doses: Variation of the number of cohorts, age, and population targeted**				
**2 doses: Girls-only Routine vaccination, No MAC**				
1. Routine 9 yrs old	112 (64-112*)	190 (68-190*)	41 (32-53)	83 (54-98)
**2 doses: Girls-only Routine vaccination with MAC**				
2a. Routine 9 yrs old with MAC 9-14 yrs old	98 (57-98*)	180 (66-180*)	40 (31-51)	80 (53-94)
3a. Routine 9 yrs old with MAC 9-18 yrs old [γ]	98 (56-98*)	172 (63-172*)	39 (31-51)	79 (53-92)
4a. Routine 9 yrs old with MAC 9-25 yrs old [γ]	102 (58-102*)	175 (67-175*)	40 (32-53)	83 (55-96)
**2 doses: Girls&Boys Routine vaccination with MAC** [†]				
2b. Routine 9 yrs old with MAC 9-14 yrs old	178 (102-178*)	306 (113-306*)	70 (55-89)	142 (92-159)
3b. Routine 9 yrs old with MAC 9-18 yrs old [γ]	175 (102-175*)	297 (110-297*)	69 (54-87)	139 (91-155)
4b. Routine 9 yrs old with MAC 9-25 yrs old [γ]	177 (101-177*)	297 (110-297*)	69 (54-88)	141 (93-156)
**2 doses: Girls-only optimisation of 9-14 years-old vaccination**				
1. Routine 9 yrs old, No MAC	112 (64-112*)	190 (68-190*)	41 (32-53)	83 (54-98)
2. Routine 9 yrs old, 5-yr extended interval[¥], No MAC	110 (62-110*)	187 (66-187*)	40 (32-52)	82 (53-96)
3. Routine 14 yrs old, No MAC	101 (57-101*)	174 (63-174*)	39 (31-50)	80 (54-89)
4. Routine 9 yrs old with MAC 9-14 yrs old	98 (57-98*)	180 (66-180*)	40 (31-51)	80 (53-94)
5. Routine 9 yrs old with 14-yr-old catch-up	99 (57-99*)	178 (67-178*)	40 (31-51)	80 (53-94)
6. Routine 9 yrs old, 5-yr extended interval[¥] with 14-yr-old catch-up	99 (57-99*)	178 (67-178*)	40 (31-52)	81 (54-95)
7. Routine 14 yrs old & later switch to Routine 9 yrs old	100 (58-100*)	176 (67-176*)	40 (32-52)	80 (53-93)
**1 dose: Girls-only Routine vaccination with MAC, Vaccine duration = 20 years**				
2c. Routine 9 yrs old with MAC 9-14 yrs old	166 (97-169)	480 (158-504)	67 (51-86)	113 (77-132)
3c. Routine 9 yrs old with MAC 9-18 yrs old [γ]	161 (96-163)	409 (141-409*)	64 (49-83)	109 (75-129)
4c. Routine 9 yrs old with MAC 9-25 yrs old [γ]	167 (97-167)	405 (135-413)	64 (51-84)	113 (76-133)
**1 dose: Girls&Boys Routine vaccination with MAC[†], Vaccine duration = 20 years**				
2d. Routine 9 yrs old with MAC 9-14 yrs old	275 (161-275*)	630 (227-630*)	110 (84-138)	197 (134-227)
3d. Routine 9 yrs old with MAC 9-18 yrs old [γ]	266 (156-266)	573 (207-573*)	105 (82-134)	189 (130-221)
4d. Routine 9 yrs old with MAC 9-25 yrs old [γ]	265 (154-265*)	582 (196-582*)	105 (83-133)	191 (129-219)
**1 dose: Girls-only Routine vaccination with MACs, Vaccine efficacy = 85%**				
2c. Routine 9 yrs old with MAC 9-14 yrs old	114 (66-114*)	207 (77-207*)	47 (36-60)	94 (62-111)
3c. Routine 9 yrs old with MAC 9-18 yrs old [γ]	114 (64-114*)	197 (74-197*)	46 (36-59)	92 (61-106)
4c. Routine 9 yrs old with MAC 9-25 yrs old [γ]	117 (66-117*)	199 (77-199*)	47 (36-61)	96 (64-110)
**1 dose: Girls&Boys Routine vaccination with MACs[†], Vaccine efficacy = 85%**				
2d. Routine 9 yrs old with MAC 9-14 yrs old	190 (110-190*)	325 (122-325*)	75 (59-95)	154 (100-180)
3d. Routine 9 yrs old with MAC 9-18 yrs old [γ]	187 (109-187*)	319 (119-319*)	74 (57-94)	150 (99-169)
4d. Routine 9 yrs old with MAC 9-25 yrs old [γ]	188 (108-188*)	321 (118-321*)	74 (57-95)	151 (100-169)

MAC: Multiple age cohort vaccination
Predictions: Mean of the 10 best fit parameter sets to Globocan 2020 (80% UI: 10th-90th percentiles of the 50 parameter sets)
Base case: 2 doses, Vaccine efficacy against HPV16/18/31/33/45/52/58 =100%, Vaccine duration=Lifetime, Vaccination coverage=80%
[α] Vaccination strategies are described in Table S1
* Maximum value is the mean of the 10 best fit parameter sets to Globocan 2020
[γ] Girls >14 years old always receive 3 doses
[¥] Extended interval: First dose at 9 years old and second dose at 14 years old
[†] Girls&Boys MAC: Girls age as indicated and boys always ≤ 14 yrs old

Table S11. Cost-effectiveness ($/DALY-averted) of the different HPV vaccination strategies (vs no vaccination)

	India		Vietnam		Uganda		Nigeria	
	$4.60/dose	$7.50/dose	$4.60/dose	$7.50/dose	$4.60/dose	$7.50/dose	$4.60/dose	$7.50/dose
**Vaccination Strategies** [α]	**Mean (80% UI)**	**Mean (80% UI)**	**Mean (80% UI)**	**Mean (80% UI)**	**Mean (80% UI)**	**Mean (80% UI)**	**Mean (80% UI)**	**Mean (80% UI)**
**GDP/0.5 GDP per capita**	**$7,056 / $3,528**		**$6,776 / $3,388**		**$1,864 / $932**		**$5,861 / $2,930**	
**2 doses: Variation of the number of cohorts, age, and population targeted**								
**2 doses: Girls-only Routine vaccination, No MAC**								
1. Routine 9 yrs old	589 (274-589*)	745 (354-745*)	1,115 (259-1,115*)	1,406 (346-1,406*)	43 (9-88)	93 (44-158)	449 (214-515)	575 (283-658)
**2 doses: Girls-only Routine vaccination with MAC**								
2a. Routine 9 yrs old with MAC 9-14 yrs old	461 (219-465)	587 (286-593)	996 (266-996*)	1,259 (354-1,259*)	40 (8-71)	89 (43-134)	429 (207-534)	550 (274-680)
3a. Routine 9 yrs old with MAC 9-18 yrs old [γ]	501 (229-501*)	637 (299-637*)	965 (259-1,020)	1,221 (346-1,288)	46 (16-83)	98 (55-151)	458 (232-574)	586 (304-730)
4a. Routine 9 yrs old with MAC 9-25 yrs old [γ]	608 (286-608*)	769 (370-769*)	1,098 (322-1,098*)	1,386 (424-1,386*)	65 (30-109)	125 (76-188)	557(287-690)	709 (372-874)
**2 doses: Girls&Boys Routine vaccination with MAC** [†]								
2b. Routine 9 yrs old with MAC 9-14 yrs old	896 (425-896*)	1,127 (541-1,127*)	1,678 (512-1,678*)	2,105 (659-2,105*)	130 (71-187)	216 (133-298)	830 (417-950)	1,048 (534-1,197)
3b. Routine 9 yrs old with MAC 9-18 yrs old [γ]	900 (439-900*)	1,131 (558-1,131*)	1,646 (506-1,752)	2,065 (652-2,197)	130 (73-187)	216 (136-297)	817 (431-947)	1,032 (552-1,192)
4b. Routine 9 yrs old with MAC 9-25 yrs old [γ]	997 (493-997*)	1,252 (625-1,252*)	1,743 (551-1,810)	2,185 (708-2,270)	145 (90-208)	237 (158-328)	912 (471-1,008)	1,149 (601-1,269)
**2 doses: Girls-only optimisation of 9-14 years-old vaccination**								
1. Routine 9 yrs old, No MAC	589 (274-589*)	745 (354-745*)	1,115 (259-1,115*)	1,406 (346-1,406*)	43 (9-88)	93 (44-158)	449 (214-515)	575 (283-658)
2. Routine 9 yrs old, 5-yr extended interval[¥], No MAC	551 (254-551*)	698 (330-698*)	1,042 (238-1,042*)	1,316 (319-1,316*)	35 (4-78)	83 (37-144)	418 (197-480)	537 (262-614)
3. Routine 14 yrs old, No MAC	447 (208-447*)	570 (272-570*)	883 (217-883*)	1,120 (294-1,120*)	28 (2-62)	73 (36-121)	395 (198-452)	509 (262-579)
4. Routine 9 yrs old with MAC 9-14 yrs old	461 (219-465)	587 (286-593)	996 (266-996*)	1,259 (354-1,259*)	40 (8-71)	89 (43-134)	429 (207-534)	550 (274-680)
5. Routine 9 yrs old with 14-yr-old catch-up	469 (220-469*)	597 (287-597*)	963 (259-969)	1,219 (345-1,225)	38 (8-74)	87 (43-138)	431 (210-501)	553 (277-639)
6. Routine 9 yrs old, 5-yr extended interval[¥] with 14-yr-old catch-up	441 (206-441*)	563 (269-563*)	907 (239-912)	1,149 (321-1,155)	32 (4-66)	78 (36-127)	404 (195-470)	519 (259-601)
7. Routine 14 yrs old & later switch to Routine 9 yrs old	478 (217-478*)	608 (283-608*)	907 (254-907*)	1,149 (339-1,149*)	39 (6-71)	88 (41-134)	419 (206-525)	538 (272-670)
**1 dose: Girls-only Routine vaccination with MAC, Vaccine duration = 20 years**								
2c. Routine 9 yrs old with MAC 9-14 yrs old	316 (142-319)	407 (189-410)	1,018 (229-1,066)	1,284 (307-1,343)	9 (CS-37)	44 (10-82)	249 (110-330)	327 (153-427)
3c. Routine 9 yrs old with MAC 9-18 yrs old [γ]	390 (183-390*)	499 (240-499*)	998 (258-998*)	1,260 (344-1,260*)	24 (CS-50)	65 (26-100)	311 (149-405)	404 (201-520)
4c. Routine 9 yrs old with MAC 9-25 yrs old [γ]	553 (264-553*)	700 (340-700*)	1,334 (349-1,334)	1,677 (456-1,677)	51 (21-87)	104 (61-152)	444 (224-553)	569 (293-704)

Vaccination Strategies [α]	India $4.60/dose Mean (80% UI)	India $7.50/dose Mean (80% UI)	Vietnam $4.60/dose Mean (80% UI)	Vietnam $7.50/dose Mean (80% UI)	Uganda $4.60/dose Mean (80% UI)	Uganda $7.50/dose Mean (80% UI)	Nigeria $4.60/dose Mean (80% UI)	Nigeria $7.50/dose Mean (80% UI)
**GDP/0.5 GDP per capita**	**$7,056 / $3,528**		**$6,776 / $3,388**		**$1,864 / $932**		**$5,861 / $2,930**	
**1 dose: Girls&Boys Routine vaccination with MAC[†], Vaccine duration = 20 years**								
2d. Routine 9 yrs old with MAC 9-14 yrs old	583 (282-602)	737 (363-761)	1,332 (394-1,337)	1,675 (511-1,679)	68 (30-101)	127 (74-175)	494 (246-604)	631 (322-768)
3d. Routine 9 yrs old with MAC 9-18 yrs old [γ]	631 (293-631*)	797 (377-797*)	1,324 (380-1,324*)	1,665 (495-1,665*)	73 (35-103)	134 (80-179)	533 (265-656)	679 (345-831)
4d. Routine 9 yrs old with MAC 9-25 yrs old [γ]	749 (363-749*)	943 (464-943*)	1,656 (462-1,656*)	2,076 (596-2,076*)	98 (53-135)	170 (105-224)	650 (323-714)	824 (418-904)
**1 dose: Girls-only Routine vaccination with MAC, Vaccine efficacy = 85%**								
2c. Routine 9 yrs old with MAC 9-14 yrs old	237 (102-242)	309 (141-316)	508 (105-508)	655 (154-655*)	CS (CS-7)	18 (CS-43)	219 (89-289)	290 (127-377)
3c. Routine 9 yrs old with MAC 9-18 yrs old [γ]	317 (131-317*)	409 (177-409*)	573 (140-573*)	736 (198-736*)	1 (CS-28)	35 (8-74)	277 (127-326)	362 (173-423)
4c. Routine 9 yrs old with MAC 9-25 yrs old [γ]	440 (206-440*)	561 (269-561*)	779 (212-779*)	991 (288-991*)	29 (1-60)	74 (34-120)	402 (201-489)	517 (266-625)
**1 dose: Girls&Boys Routine vaccination with MAC[†], Vaccine efficacy = 85%**								
2d. Routine 9 yrs old with MAC 9-14 yrs old	452 (204-452*)	576 (267-576*)	821 (218-821*)	1,042 (294-1,042*)	32 (5-62)	78 (39-122)	415 (195-517)	533 (259-660)
3d. Routine 9 yrs old with MAC 9-18 yrs old [γ]	489 (225-489*)	621 (293-621*)	921 (245-940)	1,167 (328-1,189)	42 (10-70)	93 (46-133)	448 (222-506)	574 (292-647)
4d. Routine 9 yrs old with MAC 9-25 yrs old [γ]	594 (281-594*)	752 (363-752*)	1,124 (304-1,124*)	1,418 (402-1,418*)	60 (23-97)	119 (66-172)	547 (272-620)	696 (354-788)

MAC: Multiple age cohort vaccination

CS: Cost-saving

Predictions: Mean of the 10 best fit parameter sets to Globocan 2020 (80% UI: 10th and 90th percentiles of the 50 parameter sets)

Base case: 2 doses, Vaccine efficacy against HPV16/18/31/33/45/52/58 =100%, Vaccine duration=Lifetime, Vaccination coverage=80%

[α] Vaccination strategies are described in Table S1

* Maximum value is the mean of the 10 best fit parameter sets to Globocan 2020

[γ] Girls >14 years old always receive 3 doses

[¥] Extended interval: First dose at 9 years old and second dose at 14 years old

[†] Girls&Boys MAC: Girls age as indicated and boys always ≤ 14 yrs old

Low: Low vaccine price = $4.60/dose including administration costs; High: high vaccine price including administration costs = $7.50/dose;

Table S12. Sensitivity analysis: vaccine used

A) Relative reduction at equilibrium in the age-standardized cervical cancer incidence and prevalence of HPV16/18 infection (vs no vaccination)

Strategy vs no vaccination	India			Vietnam			Uganda			Nigeria		
	Females		Males	Females		Males	Females		Males	Females		Males
	Cervical cancer	HPV 16/18	HPV 16/18	Cervical cancer	HPV 16/18	HPV 16/18	Cervical cancer	HPV 16/18	HPV 16/18	Cervical cancer	HPV 16/18	HPV 16/18
	Mean	Mean	Mean	Mean	Mean	Mean	Mean	Mean	Mean	Mean	Mean	Mean
**2 doses: Girls-only Routine vaccination with MAC 9-14 yrs old**												
9valent	86%	87%	76%	85%	83%	59%	79%	84%	53%	79%	83%	50%
2/4valent no cross	81%	87%	77%	66%	82%	58%	68%	84%	53%	53%	83%	51%
2/4valent high cross†	84%	87%	76%	79%	83%	58%	76%	84%	54%	71%	83%	50%

MAC: Multiple age cohort vaccination
Predictions: Mean of the 10 best fit parameter sets to Globocan 2020
†High cross-protection: Vaccine efficacy against HPV31=89.4%, HPV33=82.3%, HPV45=100%, HPV52=30.4%, HPV58=36.1%

B) Number needed to vaccinate (NNV) to prevent 1 cervical cancer (vs no vaccination)

Vaccination Strategies	India	Vietnam	Uganda	Nigeria
	Mean (80% UI)	Mean (80% UI)	Mean (80% UI)	Mean (80% UI)
**2 doses: Girls-only Routine vaccination with MAC 9-14 yrs old**				
**NNV (doses)**				
9valent	196 (113-196*)	361 (132-361*)	80 (63-102)	160 (106-187)
2/4valent no cross	207 (117-207*)	469 (179-473)	93 (70-119)	224 (134-238)
2/4valent high cross†	201 (115-201*)	392 (144-392*)	84 (65-106)	176 (114-203)
**NNV (vaccinees)**				
9valent	98 (57-98*)	180 (66-180*)	40 (31-51)	80 (53-94)
2/4valent no cross	104 (58-104*)	234 (89-236)	46 (35-59)	112 (67-119)
2/4valent high cross†	101 (57-101*)	196 (72-196*)	42 (32-53)	88 (57-101)

MAC: Multiple age cohort vaccination
NNV: Number needed to vaccinate
Predictions: Mean of the 10 best fit parameter sets to Globocan 2020 (80% UI: 10th and 90th percentiles of the 50 parameter sets)
* Maximum value is the mean of the 10 best fit parameter sets to Globocan 2020
† High cross-protection: Vaccine efficacy against HPV31=89.4%, HPV33=82.3%, HPV45=100%, HPV52=30.4%, HPV58=36.1%

C) Cost-effectiveness ($/DALY-averted)[¥]

	India		Vietnam		Uganda		Nigeria	
**Vaccination**	**$4.60/dose**	**$7.50/dose**	**$4.60/dose**	**$7.50/dose**	**$4.60/dose**	**$7.50/dose**	**$4.60/dose**	**$7.50/dose**
**Strategies**	**Mean (80% UI)**	**Mean (80% UI)**	**Mean (80% UI)**	**Mean (80% UI)**	**Mean (80% UI)**	**Mean (80% UI)**	**Mean (80% UI)**	**Mean (80% UI)**
**GDP/0.5 GDP per capita**	**$7,056 / $3,528**		**$6,776 / $3,388**		**$1,864 / $932**		**$5,861 / $2,930**	
**2 doses: Girls-only Routine vaccination with MAC 9-14 yrs old**								
9valent	461 (219–465)	587 (286–593)	996 (266–996*)	1,259 (354–1,259*)	40 (8–71)	89 (43–134)	429 (207–534)	550 (274-680)
2/4valent no cross	479 (228–479*)	NA	1,260 (340–1,260*)	NA	54 (18–105)	NA	562 (261–613)	NA
2/4valent high cross†	480 (218–483)	NA	1,090 (273–1,161)	NA	46 (11–77)	NA	460 (216–566)	NA
9 valent vs 2/4valent no cross	CS (CS – CS)	3,875 (905–x)	CS (CS – CS)	1,254 (217–5,247)	CS (CS – CS)	430 (96-6,437)	CS (CS – CS)	503 (213-1,284)
9 valent vs 2/4valent high cross†	CS (CS – x)	3,515 (1,169–x)	CS (CS – x)	3,246 (594–x)	CS (CS – x)	1,075 (532–x)	CS (CS – CS)	2,008 (609–x)

CS: Cost-saving
NA: not applicable
x: > 1,000,000
MAC: Multiple age cohort vaccination
Predictions: Mean of the 10 best fit parameter sets to Globocan 2020 (80% UI: 10th and 90th percentiles of the 50 parameter sets)
* Maximal value is the mean of the 10 best fit parameter sets to Globocan 2020
† High cross-protection: Vaccine efficacy against HPV31=89.4%, HPV33=82.3%, HPV45=100%, HPV52=30.4%, HPV58=36.1%
¥ We assumed that the cost of the 2/4 valent vaccine was $4.60/dose and the cost of the 9valent vaccine could be $4.60/dose or $7.50/dose (including administration costs)

Table S13. Advantages of the different HPV vaccination strategies of girls aged between 9 to 14 years old

	Routine vaccination			Additional cohorts targeted			
Advantages	9 yrs old	14 yrs old	9 yrs old extended*	Routine 9 yrs old & MAC 9-14 yrs old	Routine 9 yrs old & 14-year-old catch-up	Routine 9 yrs old extended* & 14-year-old catch-up	Routine 14 yrs old, later switch to Routine 9 yrs old
Maximises health benefits							
• Vaccination prior to sex debut	x		x (1st dose)	x	x	x (1st dose)	x
• Higher coverage	x		x (1st dose)	x	x	x (1st dose)	x
• Opportunity to vaccinate girls missed at 9 yrs old			x			x	
Accelerates health benefits		x		x	x	x	x
Reduces number of doses needed in the 1st yrs of the program	x	x	x			x	x
Programmatic feasibility	x	x		x	x		

MAC: Multiple age cohort vaccination

* First dose at 9 years old, second dose at 14 years old

Figure S1. Framework used to examine optimal HPV vaccination strategies: Policy questions, goals, analyses and outcomes

Optimization issue	Elimination of Cervical cancer	Vaccine Supply constraints	Budget constraints
Goal	Maximise health benefits	Maximise health benefits for Minimal number of doses	Maximise health benefits for Minimal cost
Analysis	Population-level impact	Efficiency	Cost-effectiveness
Outcome	Absolute reduction in cervical cancer incidence over time	Number needed to vaccinate to prevent 1 cervical cancer (NNV)	Cost per DALY

Figure S2. Description of the different HPV vaccination strategies to optimize the vaccination of girls aged 9 to 14 years old

1) Routine 9 yrs old; No MACs

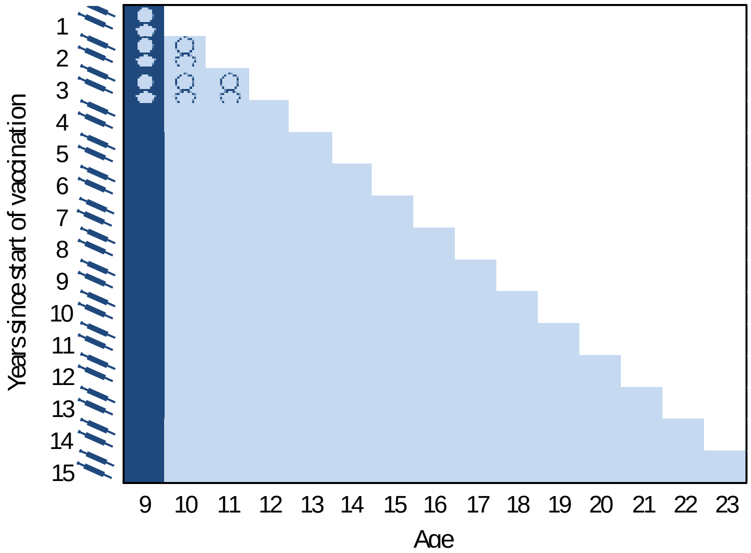

Routine vaccination of 9-year-old girls

- In the 1st year of the program:
  - girls are vaccinated at 9 years old
- In the 2nd year of the program:
  - girls vaccinated at 9 years old in the 1st year of the program are 10 years old in the 2nd year
  - a new cohort of 9-year-old girls is vaccinated
- In the 3rd year of the program:
  - girls vaccinated in the 1st and 2nd years of the program are respectively 11 and 10 years old in the 3rd year of the program
  - a new cohort of 9-year-old girls is vaccinated

2) Routine 9 years old, 5-yr extended interval, No MACs

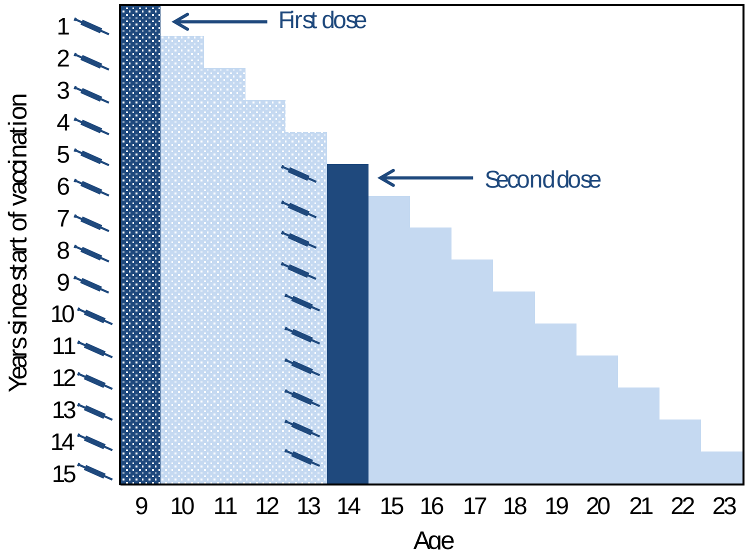

3) Routine 14 yrs old; No MACs

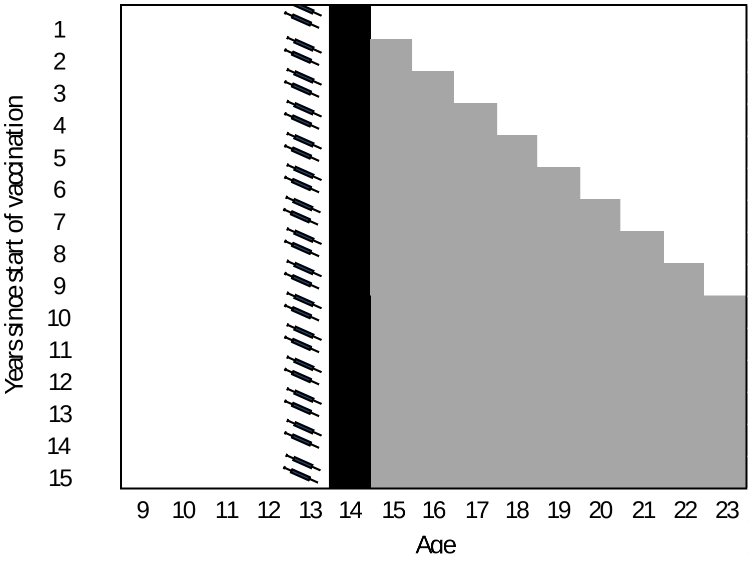

Vaccinated with 2 doses

Protected with 2 doses

Vaccinated with 2 doses between 10 and 14 yrs old

Protected with 2 doses

Vaccinated with 1st dose at 9 yrs old & 2nd dose at 14 yrs old

Protected with 1 dose

4) Routine 9 yrs old with MACs 9-14 yrs old

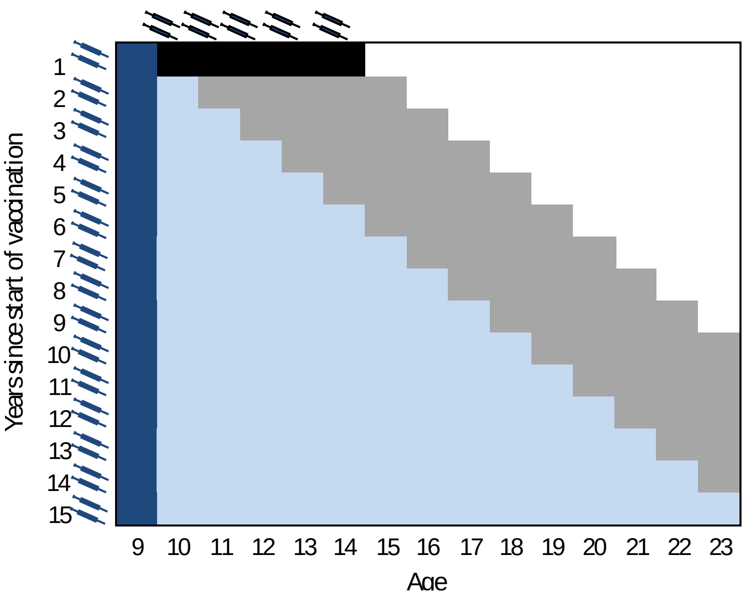

5) Routine 9 yrs old with 14-yr-old catch-up

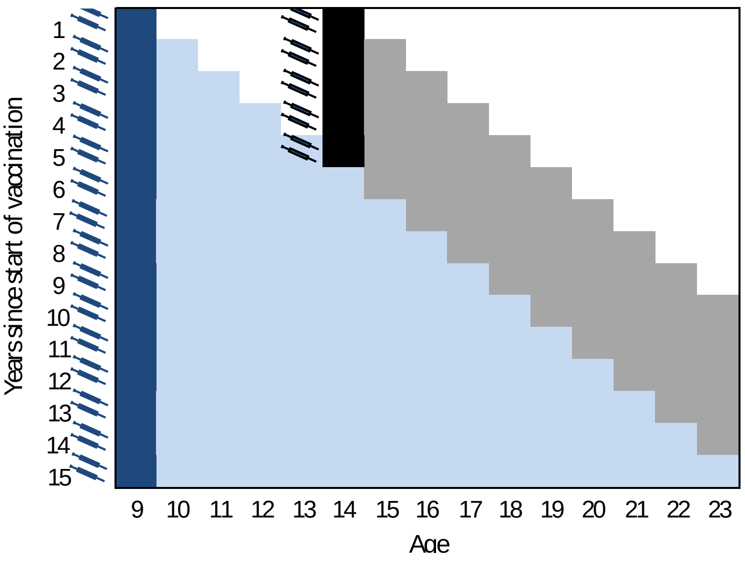

6) Routine 9 yrs old 5-yr extended interval with 14-yr-old catch-up

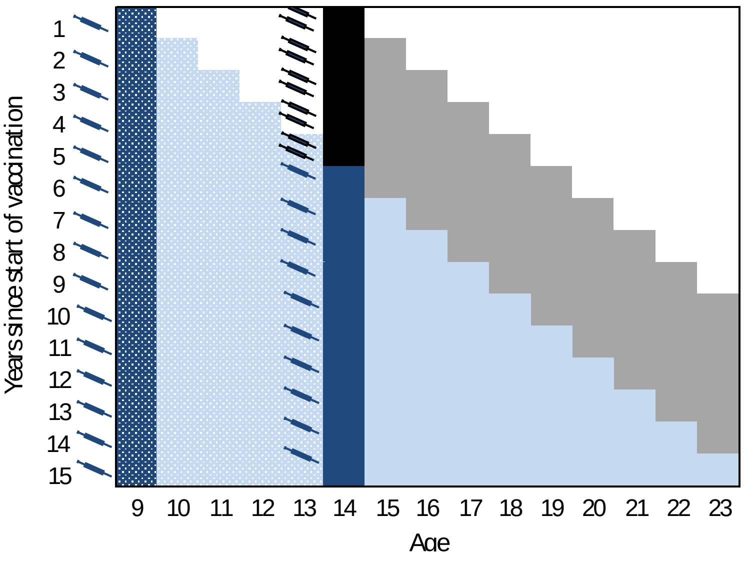

7) Routine 14 yrs old, later switch to Routine 9 yrs old

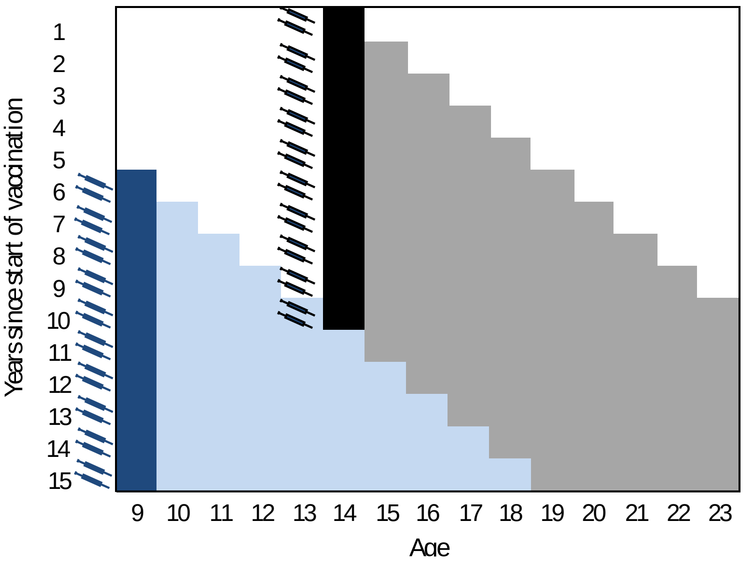

Vaccinated with 2 doses
Protected with 2 doses
Vaccinated with 2 doses between 10 and 14 yrs old
Protected with 2 doses
Vaccinated with 1st dose at 9 yrs old & 2nd dose at 14 yrs old
Protected with 1 dose

Figure S3. Examples of model fit to country-specific epidemiological data: Fit to proportion of sexually active women

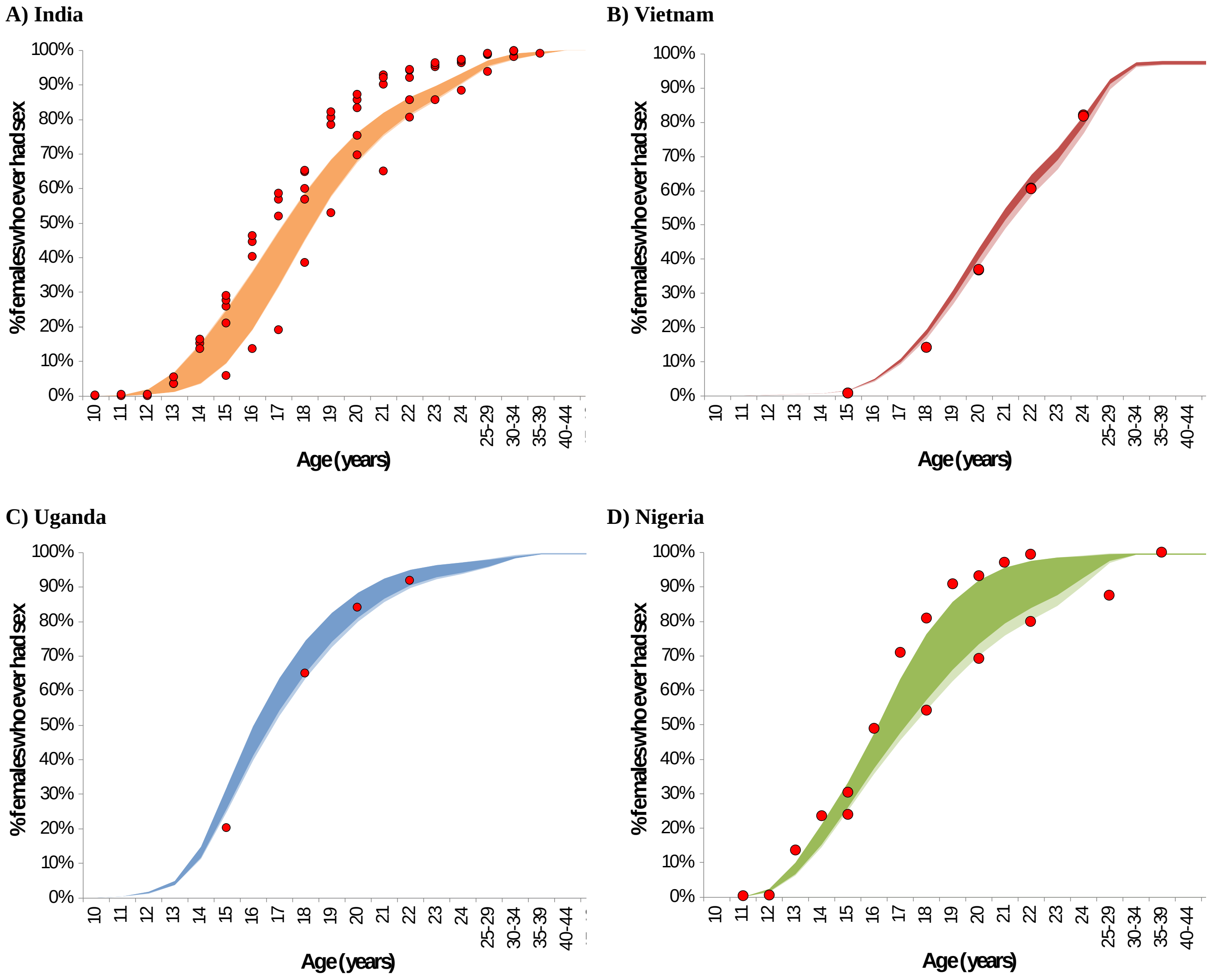

Shaded areas represent the min/max of model predictions generated by the 50 posterior parameter sets. Darker shaded areas represent min/max of 10 best fit parameter sets to Globocan 2020 cervical cancer incidence. Red dots represent observed data (India: GPS India 2008[1], NFHS-3 India 2005[2] ; Vietnam: VPAIS 2005[3]; Uganda: DHS Uganda 2016[4]; Nigeria: DHS Nigeria 2013[5]).

Figure S4. Examples of model fit to country-specific epidemiological data: Fit to HPV16/18 prevalence among sexually active females

A) India

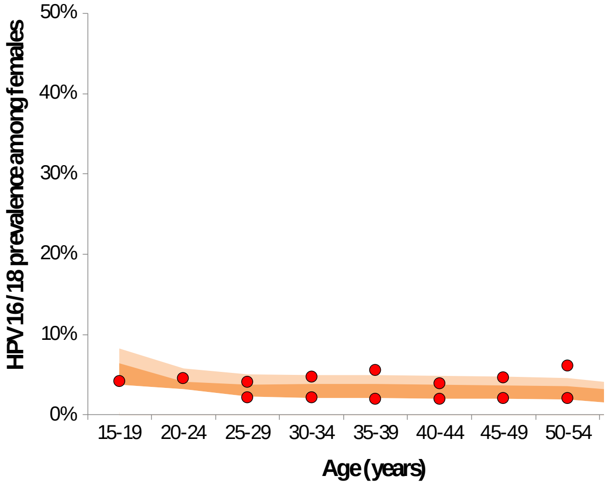

B) Vietnam

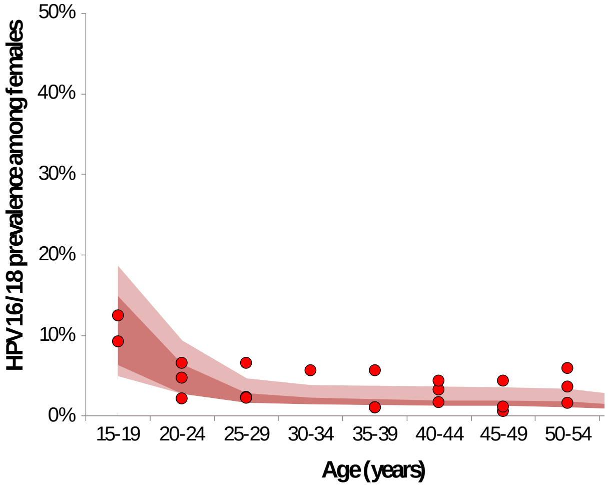

C) Uganda

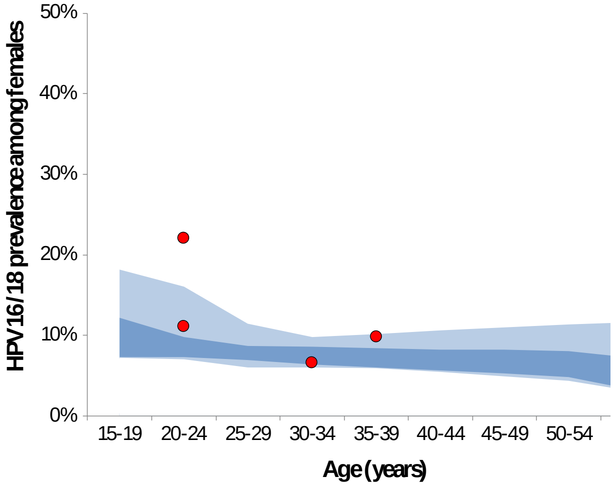

D) Nigeria

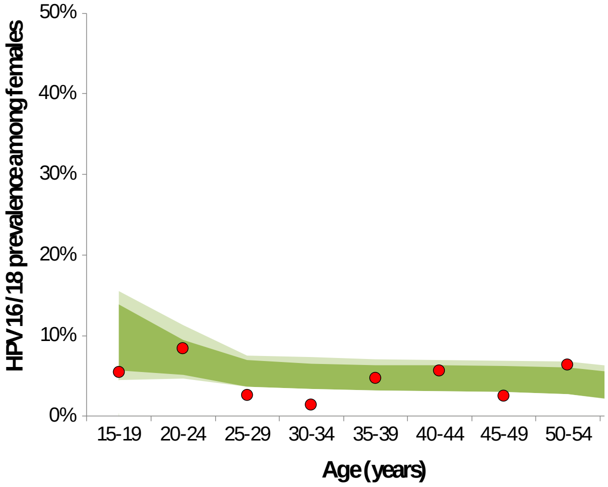

Shaded areas represent the min/max of model predictions generated by the 50 posterior parameter sets. Darker shaded areas represent min/max of 10 best fit parameter sets to Globocan 2020 cervical cancer incidence. For model predictions, we assumed a specificity of 99.7% for the HPV-test. Red dots represent the observed prevalence data (data sources for India: Dutta 2012[6], IARC prevalence data provided by Dr. Iacopo Baussano; Vietnam: Vu 2013[7] and IARC prevalence data provided by Dr. Iacopo Baussano; Uganda: Banura 2011[8], Moses 2015[9], Kumakech 2016[10]; Nigeria: IARC prevalence data for Nigeria provided by Dr. Iacopo Baussano)

Figure S5. Examples of model fit to country-specific epidemiological data: Fit to high-risk HPV prevalence among sexually active females

A) India

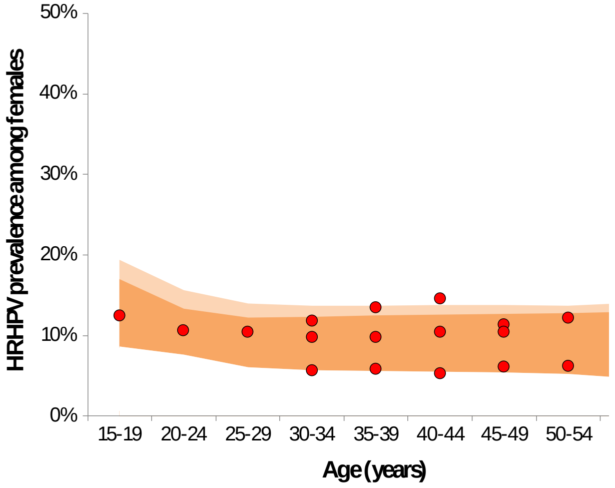

B) Vietnam

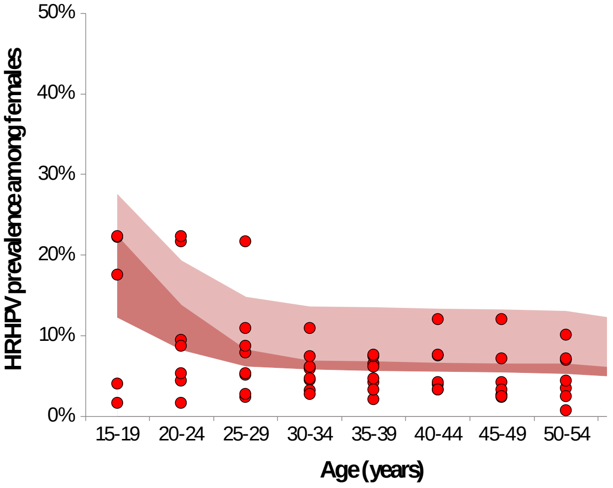

C) Uganda

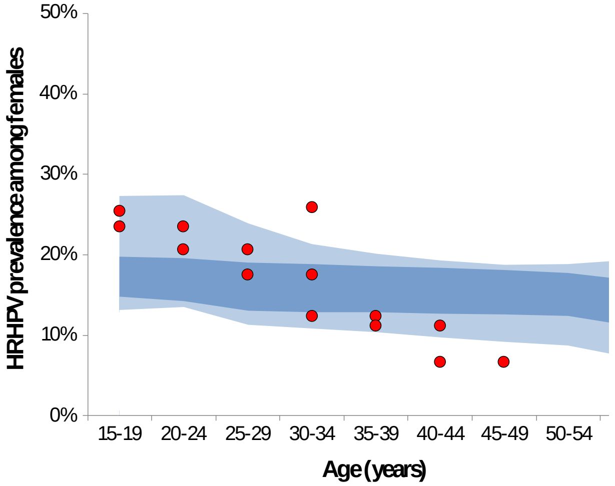

D) Nigeria

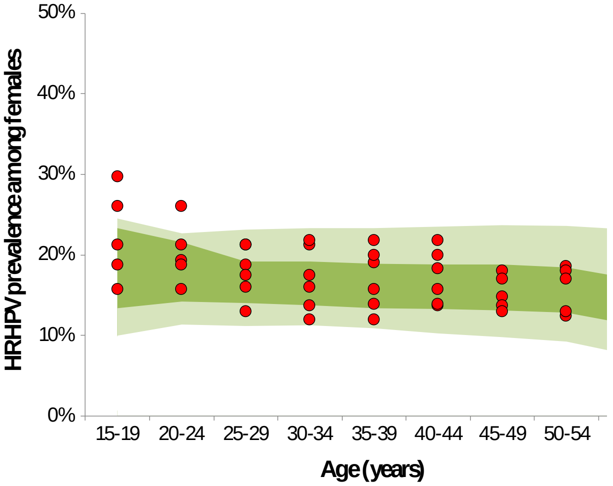

Shaded areas represent the min/max of model predictions generated by the 50 posterior parameter sets. Darker shaded areas represent min/max of 10 best fit parameter sets to Globocan 2020 cervical cancer incidence. For model predictions, we assumed a specificity of 99.7% for the HPV-test. Red dots represent the observed prevalence data (data sources for India: Sauvaget 2011[11], Basu 2013[12], and IARC prevalence data provided by Dr. Iacopo Baussano; Vietnam: Tran 2015[13], Vu 2012[14], Van 2017[15], Anh 2003[16], and IARC prevalence data provided by Dr. Iacopo Baussano; Uganda: Asiimwe 2008[17], Mitchell 2014[18], Serwadda 1999[19], Safaeian 2007[20], Safaeian 2008[21], and Moses 2015[9]; Nigeria: Ezechi 2014[22], Gage 2012[23], Thomas 2004[24], Clarke 2011[25], Adebamowo 2017[26], and IARC prevalence data for Nigeria provided by Dr. Iacopo Baussano)

Figure S6. Examples of model fit to country-specific epidemiological data: Fit to the age-specific incidence of cervical cancer

A) India

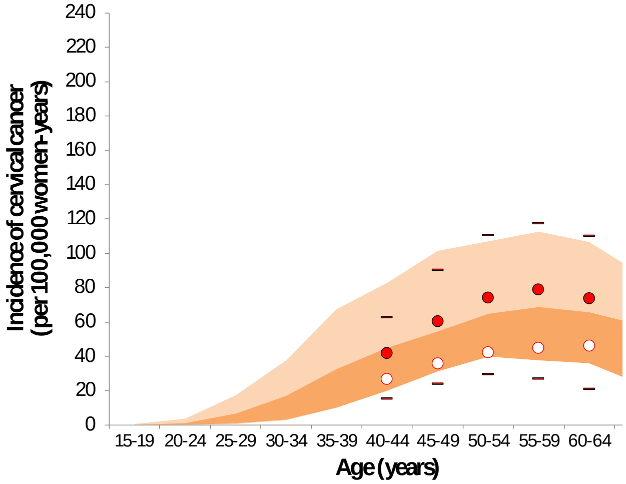

B) Vietnam

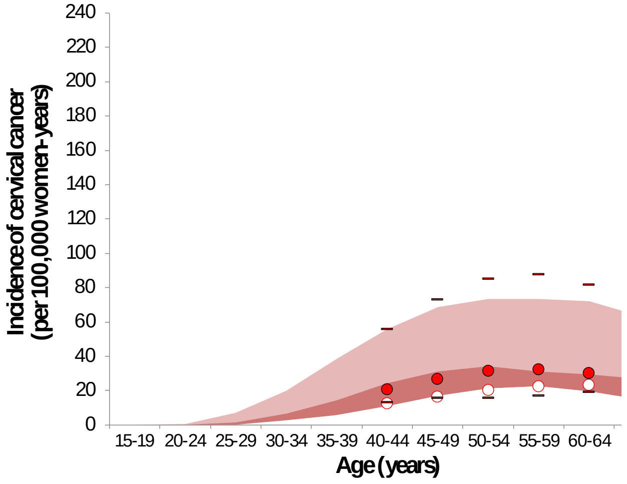

C) Uganda

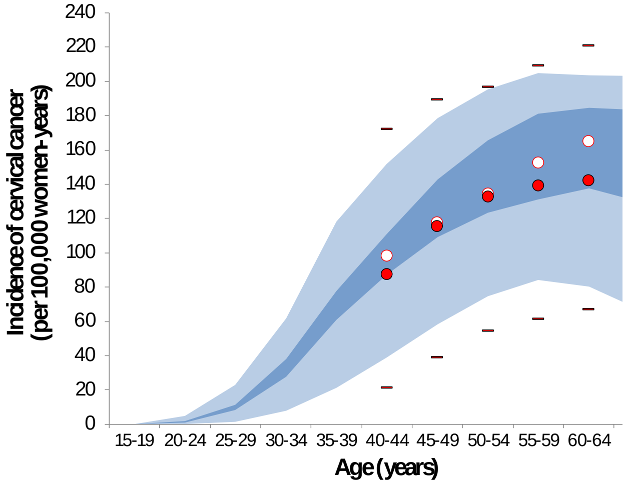

D) Nigeria

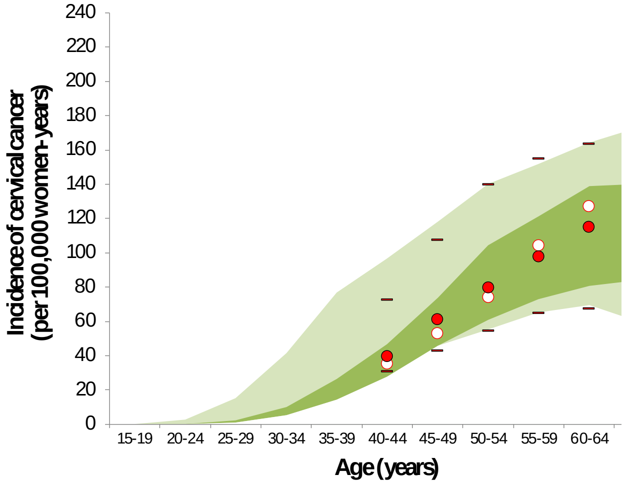

Shaded areas represent the min/max of model predictions generated by the 50 posterior parameter sets. Darker shaded areas represent min/max of 10 best fit parameter sets to Globocan 2020 cervical cancer incidence. Red dots represent the observed data for each country from Globocan 2012[27] and White dots represent the observed data for each country from Globocan 2020[28]. Red lines represent the variability of cervical cancer observed incidence for the world region or within each modeled country (India: Globocan 2012[27]; Parkin 2002[29]; Vietnam: Globocan 2012[27], Parkin 2002[29]; Uganda: Globocan 2012[27], Parkin 2002[29]; Nigeria: Globocan 2012[27])

Figure S7. Examples of model fit to country-specific epidemiological data: Fit to proportion of cervical cancers caused by high risk HPV types (HPV-16; HPV-16/18; cross-protective types HPV-31/33/45/52/58; and non-cross-protective types HPV-35/39/51/56/59/66/68/73/82)

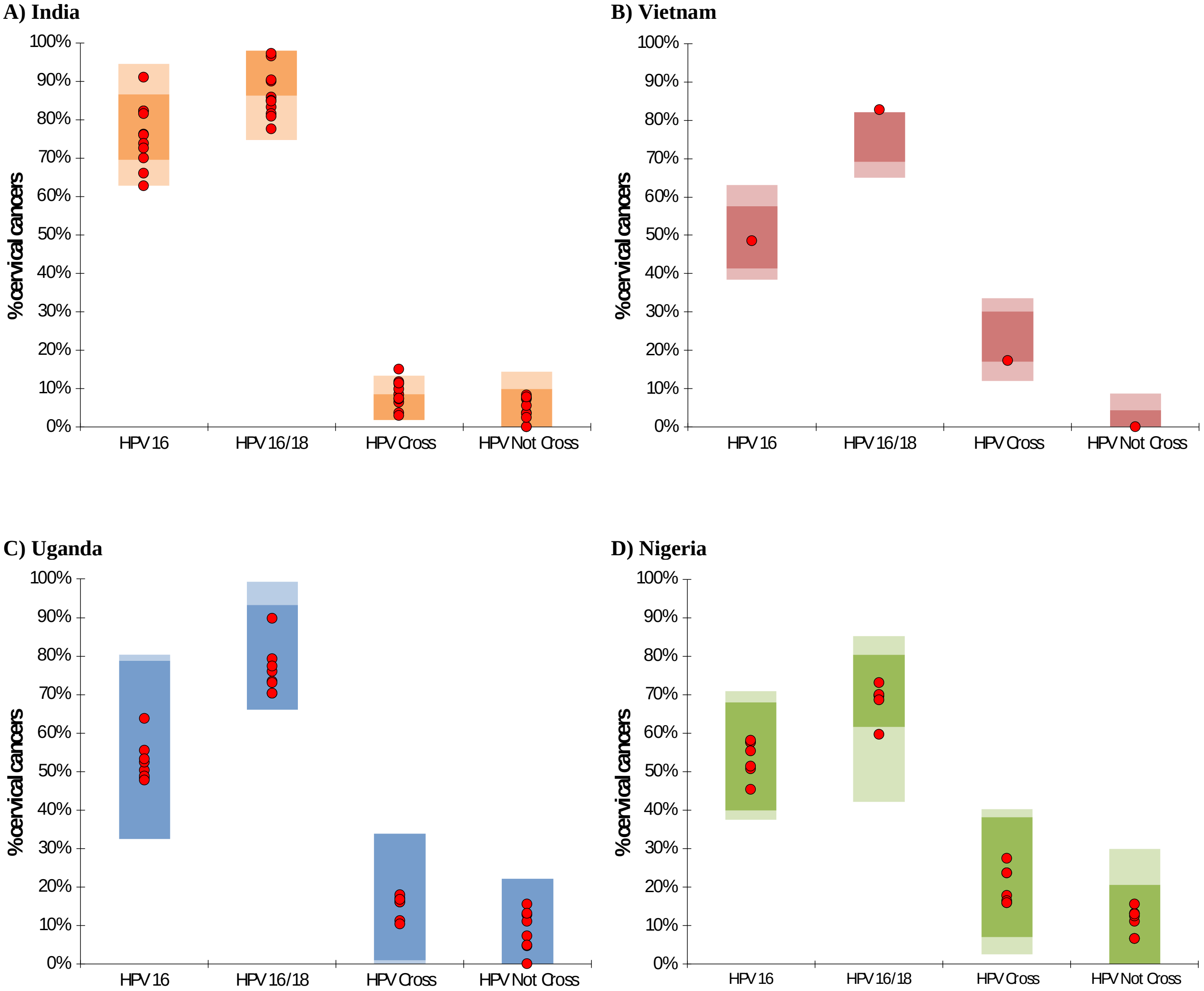

Shaded areas represent the min/max of model predictions generated by the 50 posterior parameter sets. Darker shaded areas represent min/max of 10 best fit parameter sets to Globocan 2020 cervical cancer incidence. Red dots represent the observed data (data sources for India: Serrano 2012[30], Franceschi 2003[31], Munirajan 1998[32], Sowjanya 2005[33], Pillai 2010[34], Deodhar 2012[35], Srivastava 2014[36]; Vietnam: ICO Vietnam 2017[37]; Uganda: Smith 2007[38], Ndiaye 2012[39], Odida 2008[40], Odida 2011[41], Guan 2012[42], Serrano 2012[30]; Nigeria: Lin 2001[43], Bayo 2002[44], Denny 2014[45], Ndiaye 2012[39]). Multiple HPV infections in cervical cancers were added to single types in accordance with their relative weights among single type infections in cervical cancers.

Figure S8. Impact of 1 dose duration of protection and vaccine efficacy on the population-level impact of routine vaccination of girls aged 9 years with multi-cohort vaccination (MAC)

A) Impact of 1 dose duration of protection

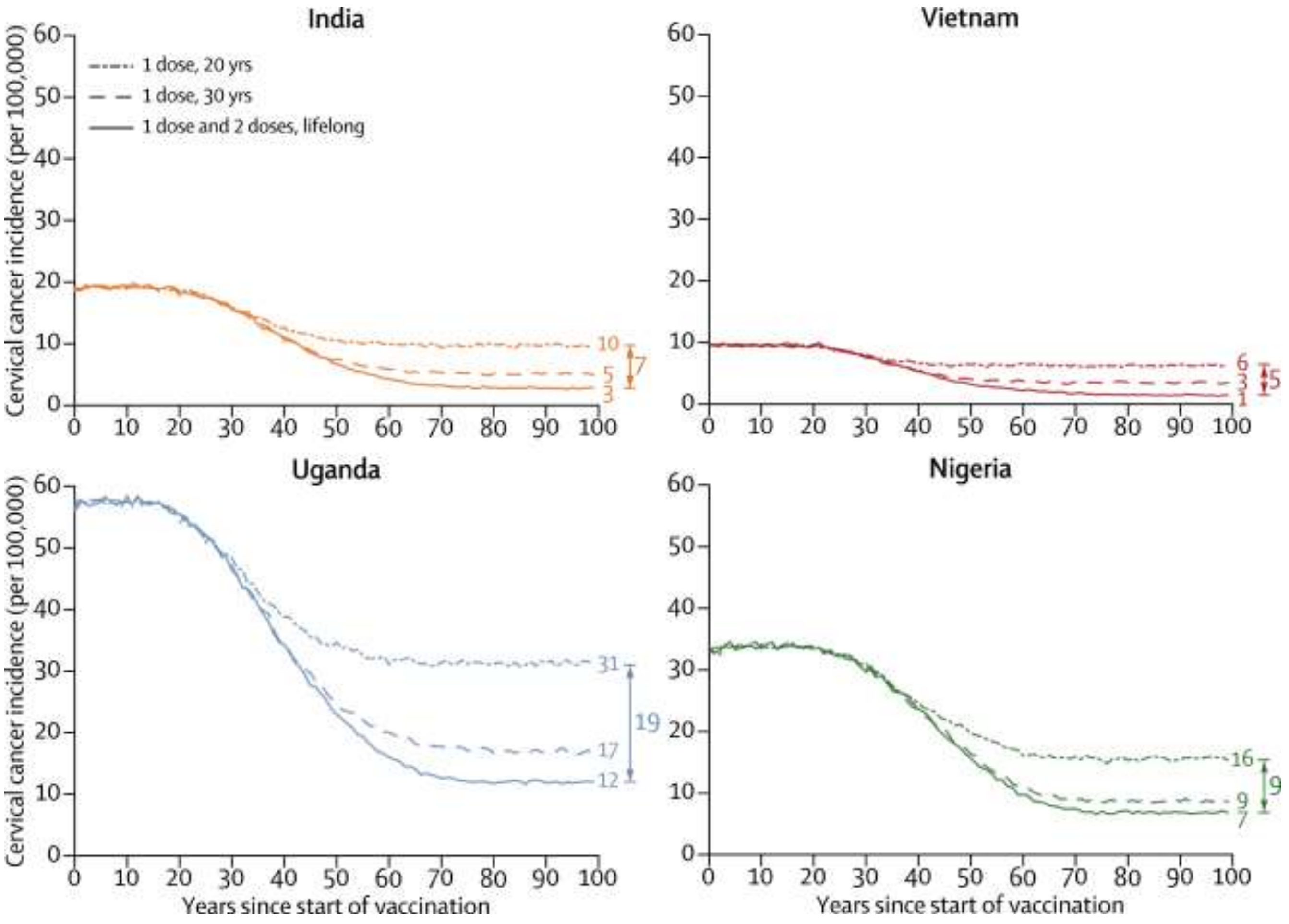

Predictions: Mean of the 10 best fit parameter sets to Globocan 2020
1 dose: Vaccine efficacy against HPV16/18/31/33/45/52/58 =100%, Vaccine duration=between 20 years and lifetime, Vaccination coverage=80%

B) Impact of 1 dose vaccine efficacy

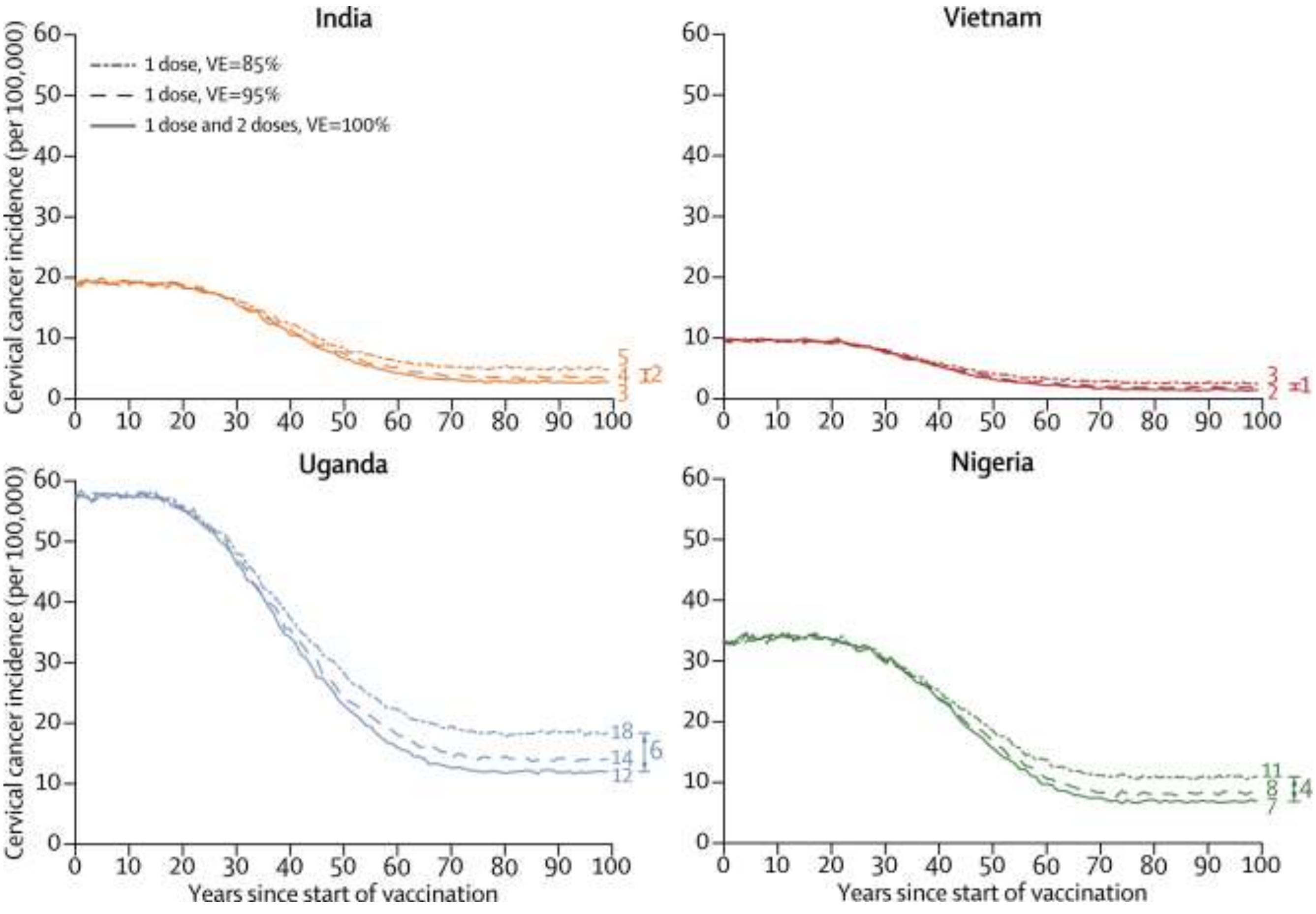

Predictions: Mean of the 10 best fit parameter sets to Globocan 2020

1 dose: Vaccine efficacy against HPV16/18/31/33/45/52/58 =varied between 85% and 100%, Vaccine duration= lifetime, Vaccination coverage=80%

Figure S9. Incremental efficiency and cost-effectiveness of different 1-dose HPV vaccination strategies varying the number of cohorts, age, and population (girls-only or girls & boys) targeted. Highlighted strategies are those that are incrementally most efficient and cost-effective. Strategies in the blue boxes are those that are incrementally most efficient and cost-effective.

A) Assuming vaccine duration = 20 years

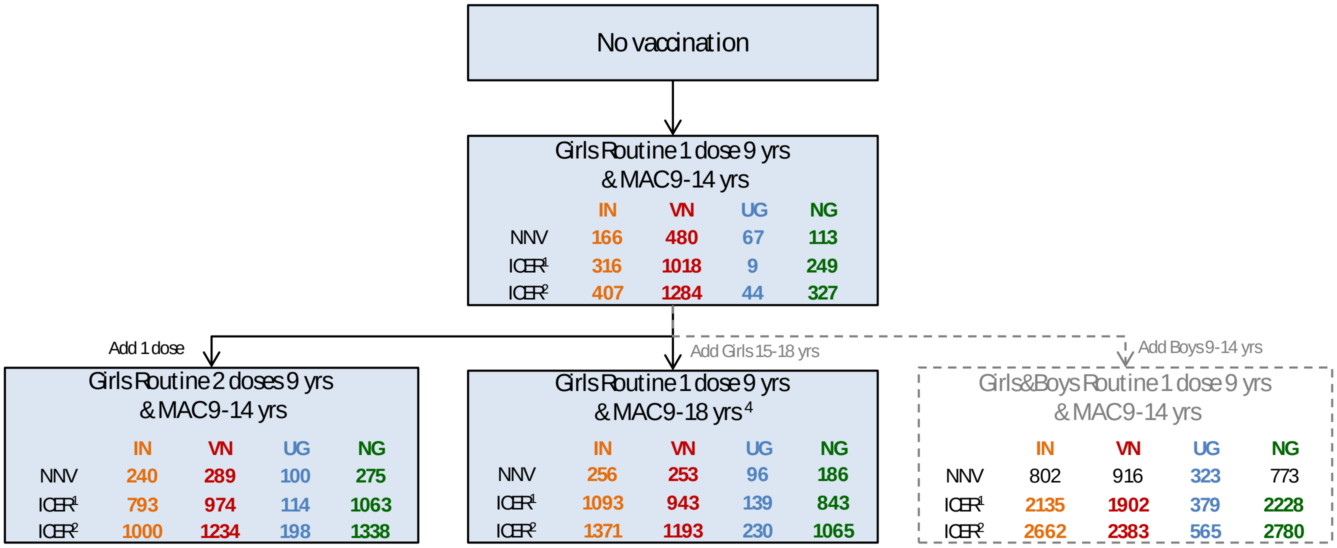

B) Assuming vaccine efficacy = 85%

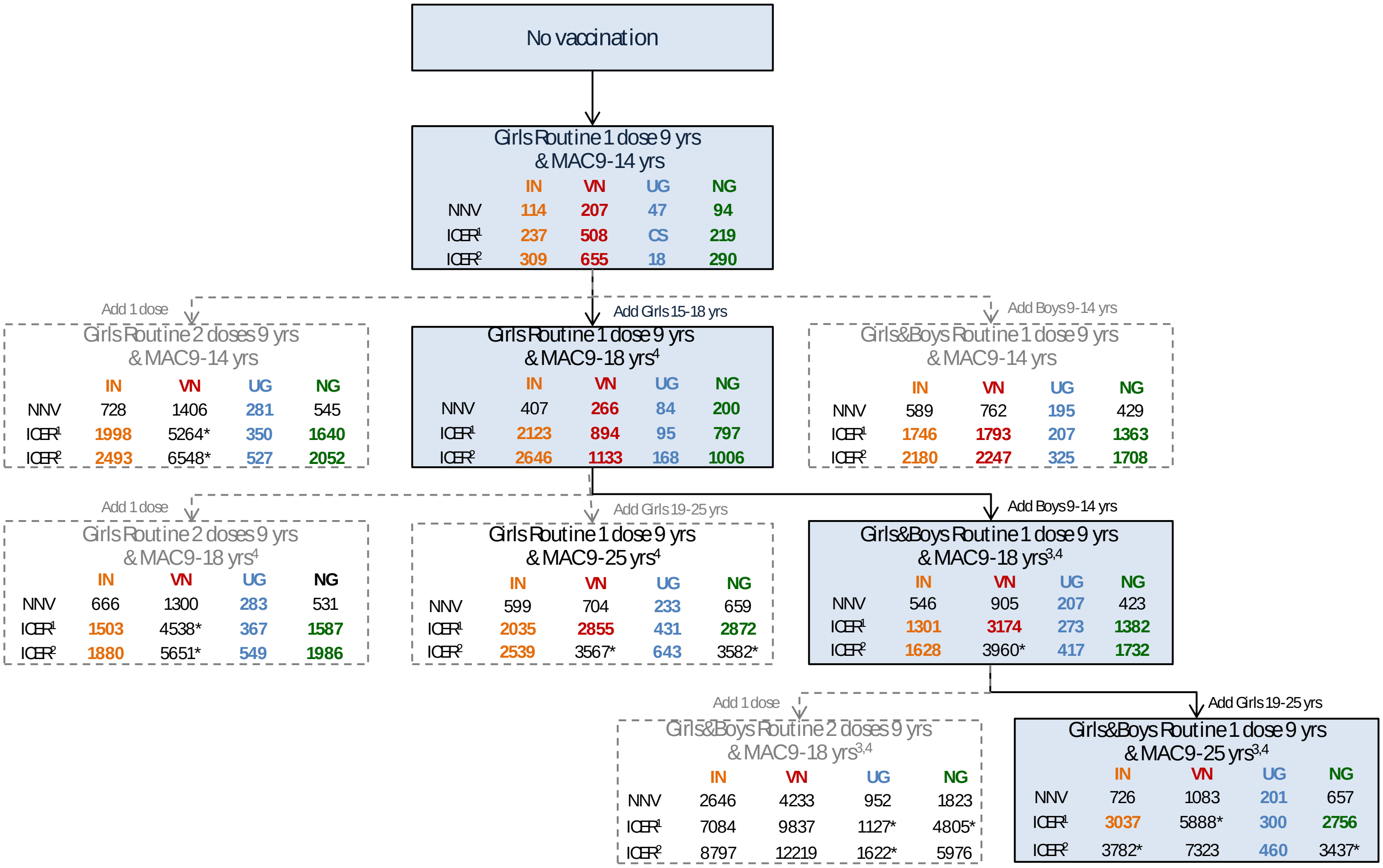

MAC: Multiple age cohort vaccination
NNV: Number of doses needed to prevent 1 cervical cancer
ICER: Incremental cost-effectiveness ratio
NNV in color are below 400; ICER in color are below 0.5 GDP per capita; ICER* are below the GDP per capita
Strategies in blue are the most efficient and cost-effective when vaccinating additional cohorts of individuals
Base case: 2 doses, Vaccine efficacy against HPV16/18/31/33/45/52/58 =100%, Vaccine duration=Lifetime, Vaccination coverage=80%
Predictions: Mean of the 10 best fit parameter sets to Globocan 2020
[1] Vaccine cost per dose including administration costs: $4.60
[2] Vaccine cost per dose including administration costs: $7.50
[3] Girls&Boys MAC: Girls age as indicated, boys always ≤14 yrs;
[4] Girls >14 years always have 3 doses

REFERENCES

1. Banandur P, Rajaram SP, Mahagaonkar SB, et al. Heterogeneity of the HIV epidemic in the general population of Karnataka state, south India. *BMC Public Health* 2011; 11 Suppl 6: S13.
2. International Institute for Population Sciences (IIPS) and Macro International. National Family Health Survey (NFHS-3), 2005–06: India: Volume I. Mumbai: IIPS., 2007.
3. General Statistics Office of Vietnam (GSO), National Institute of Hygiene and Epidemiology (NIHE) [Vietnam], ORC Macro. Vietnam Population and AIDS Indicator Survey (VPAIS) 2005. Calverton, Maryland, USA: GSO, NIHE, and ORC Macro, 2006.
4. Uganda Bureau of Statistics (UBOS) and ICF. Uganda Demographic and Health Survey 2016. Kampala, Uganda and Rockville, Maryland, USA: UBOS and ICF, 2018.
5. National Population Commission (NPC) [Nigeria] and ICF International. Nigeria Demographic and Health Survey 2013. Abuja, Nigeria, and Rockville, Maryland, USA: NPC and ICF International, 2014.
6. Dutta S, Begum R, Mazumder Indra D, et al. Prevalence of human papillomavirus in women without cervical cancer: a population-based study in Eastern India. *Int J Gynecol Pathol* 2012; 31(2): 178-83.
7. Vu LT, Bui D, Le HT. Prevalence of cervical infection with HPV type 16 and 18 in Vietnam: implications for vaccine campaign. *BMC Cancer* 2013; 13: 53.
8. Banura C, Mirembe FM, Katahoire AR, Namujju PB, Mbonye AK, Wabwire FM. Epidemiology of HPV genotypes in Uganda and the role of the current preventive vaccines: A systematic review. *Infect Agent Cancer* 2011; 6(1): 11.
9. Moses E, Pedersen HN, Mitchell SM, et al. Uptake of community-based, self-collected HPV testing vs. visual inspection with acetic acid for cervical cancer screening in Kampala, Uganda: preliminary results of a randomised controlled trial. *Trop Med Int Health* 2015; 20(10): 1355-67.
10. Kumakech E, Berggren V, Wabinga H, et al. Significantly Reduced Genoprevalence of Vaccine-Type HPV-16/18 Infections among Vaccinated Compared to Non-Vaccinated Young Women 5.5 Years after a Bivalent HPV-16/18 Vaccine (Cervarix(R)) Pilot Project in Uganda. *PLoS One* 2016; 11(8): e0160099.
11. Sauvaget C, Nene BM, Jayant K, et al. Prevalence and determinants of high-risk human papillomavirus infection in middle-aged Indian women. *Sex Transm Dis* 2011; 38(10): 902-6.
12. Basu P, Mittal S, Bhaumik S, et al. Prevalence of high-risk human papillomavirus and cervical intraepithelial neoplasias in a previously unscreened population--a pooled analysis from three studies. *Int J Cancer* 2013; 132(7): 1693-9.
13. Tran LT, Tran LT, Bui TC, et al. Risk factors for high-risk and multi-type Human Papillomavirus infections among women in Ho Chi Minh City, Vietnam: a cross-sectional study. *BMC Womens Health* 2015; 15: 16.
14. Vu LT, Bui D. Prevalence of cervical human papilloma virus infection among married women in Vietnam, 2011. *Asian Pac J Cancer Prev* 2012; 13(1): 37-40.
15. Van SN, Khac MN, Dimberg J, Matussek A, Henningsson AJ. Prevalence of Cervical Infection and Genotype Distribution of Human Papilloma Virus Among Females in Da Nang, Vietnam. *Anticancer Res* 2017; 37(3): 1243-7.
16. Pham TH, Nguyen TH, Herrero R, et al. Human papillomavirus infection among women in South and North Vietnam. *Int J Cancer* 2003; 104(2): 213-20.
17. Asiimwe S, Whalen CC, Tisch DJ, Tumwesigye E, Sethi AK. Prevalence and predictors of high-risk human papillomavirus infection in a population-based sample of women in rural Uganda. *Int J STD AIDS* 2008; 19(9): 605-10.
18. Mitchell SM, Sekikubo M, Biryabarema C, et al. Factors associated with high-risk HPV positivity in a low-resource setting in sub-Saharan Africa. *Am J Obstet Gynecol* 2014; 210(1): 81 e1-7.
19. Serwadda D, Wawer MJ, Shah KV, et al. Use of a hybrid capture assay of self-collected vaginal swabs in rural Uganda for detection of human papillomavirus. *J Infect Dis* 1999; 180(4): 1316-9.
20. Safaeian M, Kiddugavu M, Gravitt PE, et al. Comparability of self-collected vaginal swabs and physician-collected cervical swabs for detection of human papillomavirus infections in Rakai, Uganda. *Sex Transm Dis* 2007; 34(7): 429-36.
21. Safaeian M, Kiddugavu M, Gravitt PE, et al. Prevalence and risk factors for carcinogenic human papillomavirus infections in rural Rakai, Uganda. *Sex Transm Infect* 2008; 84(4): 306-11.
22. Ezechi OC, Ostergren PO, Nwaokorie FO, Ujah IA, Odberg Pettersson K. The burden, distribution and risk factors for cervical oncogenic human papilloma virus infection in HIV positive Nigerian women. *Virol J* 2014; 11: 5.
23. Gage JC, Ajenifuja KO, Wentzensen NA, et al. The age-specific prevalence of human papillomavirus and risk of cytologic abnormalities in rural Nigeria: implications for screen-and-treat strategies. *Int J Cancer* 2012; 130(9): 2111-7.
24. Thomas JO, Herrero R, Omigbodun AA, et al. Prevalence of papillomavirus infection in women in Ibadan, Nigeria: a population-based study. *Br J Cancer* 2004; 90(3): 638-45.

25. Clarke MA, Gage JC, Ajenifuja KO, et al. A population-based cross-sectional study of age-specific risk factors for high risk human papillomavirus prevalence in rural Nigeria. *Infect Agent Cancer* 2011; 6: 12.
26. Adebamowo SN, Olawande O, Famooto A, et al. Persistent Low-Risk and High-Risk Human Papillomavirus Infections of the Uterine Cervix in HIV-Negative and HIV-Positive Women. *Front Public Health* 2017; 5: 178.
27. International Agency for Research on Cancer (IARC). Incidence/Mortality, Age-specific tables, Cervix Uteri. Available at http://gco.iarc.fr/today/home. Accessed March 2 2017.
28. International Agency for Research on Cancer. Globocan 2020. Cervical cancer incidence and mortality worldwide 2020. Available at https://gco.iarc.fr/tomorrow/home. Accessed October 24 2019.
29. Parkin DM, Whelan SL, Ferlay J, Teppo L, Thomas DB, Editors. Cancer Incidence in Five Continents Vol. VIII, 2002.
30. Serrano B, Alemany L, Tous S, et al. Potential impact of a nine-valent vaccine in human papillomavirus related cervical disease. *Infect Agent Cancer* 2012; 7(1): 38.
31. Franceschi S, Rajkumar T, Vaccarella S, et al. Human papillomavirus and risk factors for cervical cancer in Chennai, India: a case-control study. *Int J Cancer* 2003; 107(1): 127-33.
32. Munirajan AK, Kannan K, Bhuvarahamurthy V, et al. The status of human papillomavirus and tumor suppressor genes p53 and p16 in carcinomas of uterine cervix from India. *Gynecol Oncol* 1998; 69(3): 205-9.
33. Sowjanya AP, Jain M, Poli UR, et al. Prevalence and distribution of high-risk human papilloma virus (HPV) types in invasive squamous cell carcinoma of the cervix and in normal women in Andhra Pradesh, India. *BMC Infect Dis* 2005; 5: 116.
34. Pillai RM, Babu JM, Jissa VT, et al. Region-wise distribution of high-risk human papillomavirus types in squamous cell carcinomas of the cervix in India. *Int J Gynecol Cancer* 2010; 20(6): 1046-51.
35. Deodhar K, Gheit T, Vaccarella S, et al. Prevalence of human papillomavirus types in cervical lesions from women in rural Western India. *J Med Virol* 2012; 84(7): 1054-60.
36. Srivastava S, Shahi UP, Dibya A, Gupta S, Roy JK. Distribution of HPV Genotypes and Involvement of Risk Factors in Cervical Lesions and Invasive Cervical Cancer: A Study in an Indian Population. *Int J Mol Cell Med* 2014; 3(2): 61-73.
37. Bruni L, Barrionuevo-Rosas L, Albero G, et al. ICO Information Centre on HPV and Cancer (HPV Information Centre). Human Papillomavirus and Related Diseases in Viet Nam. Summary Report 27 July 2017. Accessed May 2017.
38. Smith JS, Lindsay L, Hoots B, et al. Human papillomavirus type distribution in invasive cervical cancer and high-grade cervical lesions: a meta-analysis update. *Int J Cancer* 2007; 121(3): 621-32.
39. Ndiaye C, Alemany L, Ndiaye N, et al. Human papillomavirus distribution in invasive cervical carcinoma in sub-Saharan Africa: could HIV explain the differences? *Trop Med Int Health* 2012; 17(12): 1432-40.
40. Odida M, de Sanjose S, Quint W, Bosch XF, Klaustermeier J, Weiderpass E. Human Papillomavirus type distribution in invasive cervical cancer in Uganda. *BMC Infect Dis* 2008; 8: 85.
41. Odida M, Sandin S, Mirembe F, Kleter B, Quint W, Weiderpass E. HPV types, HIV and invasive cervical carcinoma risk in Kampala, Uganda: a case-control study. *Infect Agent Cancer* 2011; 6(1): 8.
42. Guan P, Howell-Jones R, Li N, et al. Human papillomavirus types in 115,789 HPV-positive women: A meta-analysis from cervical infection to cancer. *Int J Cancer* 2012; 131(10): 2349-59.
43. Lin P, Koutsky LA, Critchlow CW, et al. HLA class II DR-DQ and increased risk of cervical cancer among Senegalese women. *Cancer Epidemiol Biomarkers Prev* 2001; 10(10): 1037-45.
44. Bayo S, Bosch FX, de Sanjose S, et al. Risk factors of invasive cervical cancer in Mali. *Int J Epidemiol* 2002; 31(1): 202-9.
45. Denny L, Adewole I, Anorlu R, et al. Human papillomavirus prevalence and type distribution in invasive cervical cancer in sub-Saharan Africa. *Int J Cancer* 2014; 134(6): 1389-98.

CHEERS checklist

CHEERS Checklist

Items to include when reporting economic evaluations of health interventions

The **ISPOR CHEERS Task Force Report**, *Consolidated Health Economic Evaluation Reporting Standards (CHEERS)—Explanation and Elaboration: A Report of the ISPOR Health Economic Evaluations Publication Guidelines Good Reporting Practices Task Force*, provides examples and further discussion of the 24-item CHEERS Checklist and the CHEERS Statement. It may be accessed via the *Value in Health* or via the ISPOR Health Economic Evaluation Publication Guidelines – CHEERS: Good Reporting Practices webpage: http://www.ispor.org/TaskForces/EconomicPubGuidelines.asp

Section/item	Item No	Recommendation	Reported on page No/ line No
**Title and abstract**			
Title	1	Identify the study as an economic evaluation or use more specific terms such as "cost-effectiveness analysis", and describe the interventions compared.	Not applicable
Abstract	2	Provide a structured summary of objectives, perspective, setting, methods (including study design and inputs), results (including base case and uncertainty analyses), and conclusions.	P2:L1-35
**Introduction**			
Background and objectives	3	Provide an explicit statement of the broader context for the study.<br>Present the study question and its relevance for health policy or practice decisions.	P4:L1-39
**Methods**			
Target population and subgroups	4	Describe characteristics of the base case population and subgroups analysed, including why they were chosen.	P5:L2-19
Setting and location	5	State relevant aspects of the system(s) in which the decision(s) need(s) to be made.	P5:L2-11
Study perspective	6	Describe the perspective of the study and relate this to the costs being evaluated.	P7:L25-26
Comparators	7	Describe the interventions or strategies being compared and state why they were chosen.	P5:L12-20; Appendix Table S1, Fig. S2
Time horizon	8	State the time horizon(s) over which costs and consequences are being evaluated and say why appropriate.	P7:L27-28
Discount rate	9	Report the choice of discount rate(s) used for costs and outcomes and say why appropriate.	P7:L26-29
Choice of health outcomes	10	Describe what outcomes were used as the measure(s) of benefit in the evaluation and their relevance for the type of analysis performed.	P7:L4-22
Measurement of effectiveness	11a	*Single study-based estimates:* Describe fully the design features of the single effectiveness study and why the single study was a sufficient source of clinical effectiveness data.	Not applicable

	11b	*Synthesis-based estimates:* Describe fully the methods used for identification of included studies and synthesis of clinical effectiveness data.	P6:L14-36<br>Technical appendix*
Measurement and valuation of preference based outcomes	12	If applicable, describe the population and methods used to elicit preferences for outcomes.	Not applicable
Estimating resources and costs	13a	*Single study-based economic evaluation:* Describe approaches used to estimate resource use associated with the alternative interventions. Describe primary or secondary research methods for valuing each resource item in terms of its unit cost. Describe any adjustments made to approximate to opportunity costs.	Not applicable
	13b	*Model-based economic evaluation:* Describe approaches and data sources used to estimate resource use associated with model health states. Describe primary or secondary research methods for valuing each resource item in terms of its unit cost. Describe any adjustments made to approximate to opportunity costs.	P6:L37-41<br>Appendix Table S2
Currency, price date, and conversion	14	Report the dates of the estimated resource quantities and unit costs. Describe methods for adjusting estimated unit costs to the year of reported costs if necessary. Describe methods for converting costs into a common currency base and the exchange rate.	P6:L41<br>P7:L1-2<br>Appendix Table S2
Choice of model	15	Describe and give reasons for the specific type of decision-analytical model used. Providing a figure to show model structure is strongly recommended.	P5:L21-38<br>P6:L1-13<br>Technical appendix*
Assumptions	16	Describe all structural or other assumptions underpinning the decision-analytical model.	P5:L21-38;P6:L1-13, L30-41; Technical app.*
Analytical methods	17	Describe all analytical methods supporting the evaluation. This could include methods for dealing with skewed, missing, or censored data; extrapolation methods; methods for pooling data; approaches to validate or make adjustments (such as half cycle corrections) to a model; and methods for handling population heterogeneity and uncertainty.	P5:L21-38<br>P6: L1-41<br>P7:1-2<br>Appendix Fig. S3-7<br>Technical appendix*
**Results**			
Study parameters	18	Report the values, ranges, references, and, if used, probability distributions for all parameters. Report reasons or sources for distributions used to represent uncertainty where appropriate. Providing a table to show the input values is strongly recommended.	P6:L14-41<br>Appendix Table S2<br>Technical appendix*
Incremental costs and outcomes	19	For each intervention, report mean values for the main categories of estimated costs and outcomes of interest, as well as mean differences between the comparator groups. If applicable, report incremental cost-effectiveness ratios.	P8:L1-39;<br>P9:L1-38<br>P17-22:Fig.1-6<br>Appendix Table S5-6
Characterising uncertainty	20a	*Single study-based economic evaluation:* Describe the effects of sampling uncertainty for the estimated incremental cost and incremental effectiveness parameters, together with the impact	Not applicable

*Technical appendix available at: http://www.marc-brisson.net/HPVadvise-LMIC.pdf

		of methodological assumptions (such as discount rate, study perspective).	
	20b	*Model-based economic evaluation:* Describe the effects on the results of uncertainty for all input parameters, and uncertainty related to the structure of the model and assumptions.	P10:L1-17; P13:15-19; Appendix Tables S5-12
Characterising heterogeneity	21	If applicable, report differences in costs, outcomes, or cost-effectiveness that can be explained by variations between subgroups of patients with different baseline characteristics or other observed variability in effects that are not reducible by more information.	Not applicable
**Discussion**			
Study findings, limitations, generalisability, and current knowledge	22	Summarise key study findings and describe how they support the conclusions reached. Discuss limitations and the generalisability of the findings and how the findings fit with current knowledge.	P10:L19-39; P11:L1-41; P12:L1-42; P13:L1-40 P14:L1-5
**Other**			
Source of funding	23	Describe how the study was funded and the role of the funder in the identification, design, conduct, and reporting of the analysis. Describe other non-monetary sources of support.	P7:L34-39 P14:L9-12
Conflicts of interest	24	Describe any potential for conflict of interest of study contributors in accordance with journal policy. In the absence of a journal policy, we recommend authors comply with International Committee of Medical Journal Editors recommendations.	P14:L7

For consistency, the CHEERS Statement checklist format is based on the format of the CONSORT statement checklist

The **ISPOR CHEERS Task Force Report** provides examples and further discussion of the 24-item CHEERS Checklist and the CHEERS Statement. It may be accessed via the *Value in Health* link or via the ISPOR Health Economic Evaluation Publication Guidelines – CHEERS: Good Reporting Practices webpage: http://www.ispor.org/TaskForces/EconomicPubGuidelines.asp

The citation for the CHEERS Task Force Report is:
Husereau D, Drummond M, Petrou S, et al. Consolidated health economic evaluation reporting standards (CHEERS)—Explanation and elaboration: A report of the ISPOR health economic evaluations publication guidelines good reporting practices task force. Value Health 2013;16:231-50.
